# Measuring re-identification risk using a synthetic estimator to enable data sharing

**DOI:** 10.1371/journal.pone.0269097

**Published:** 2022-06-17

**Authors:** Yangdi Jiang, Lucy Mosquera, Bei Jiang, Linglong Kong, Khaled El Emam

**Affiliations:** 1 Department of Mathematical and Statistical Sciences, University of Alberta, Edmonton, Canada; 2 Replica Analytics Ltd., Ottawa, Ontario, Canada; 3 School of Epidemiology and Public Health, University of Ottawa, Ottawa, Ontario, Canada; 4 Childrens Hospital of Eastern Ontario Research Institute, Ottawa, Ontario, Canada; UNITED STATES

## Abstract

**Background:**

One common way to share health data for secondary analysis while meeting increasingly strict privacy regulations is to de-identify it. To demonstrate that the risk of re-identification is acceptably low, re-identification risk metrics are used. There is a dearth of good risk estimators modeling the attack scenario where an adversary selects a record from the microdata sample and attempts to match it with individuals in the population.

**Objectives:**

Develop an accurate risk estimator for the sample-to-population attack.

**Methods:**

A type of estimator based on creating a synthetic variant of a population dataset was developed to estimate the re-identification risk for an adversary performing a sample-to-population attack. The accuracy of the estimator was evaluated through a simulation on four different datasets in terms of estimation error. Two estimators were considered, a Gaussian copula and a d-vine copula. They were compared against three other estimators proposed in the literature.

**Results:**

Taking the average of the two copula estimates consistently had a median error below 0.05 across all sampling fractions and true risk values. This was significantly more accurate than existing methods. A sensitivity analysis of the estimator accuracy based on variation in input parameter accuracy provides further application guidance. The estimator was then used to assess re-identification risk and de-identify a large Ontario COVID-19 behavioral survey dataset.

**Conclusions:**

The average of two copula estimators consistently provides the most accurate re-identification risk estimate and can serve as a good basis for managing privacy risks when data are de-identified and shared.

## 1. Introduction

It has been argued that access to data is key to fight the COVID-19 pandemic [1–4], especially since AI methods that are being applied to analyze COVID-19 data require larger volumes of data [5,6]. However, privacy concerns are growing [7–10], and some governments have begun to reduce the amount of information being shared about COVID-19 cases [7,11–16].

More broadly, access to health data for secondary research remains a challenge [17–20]. Assessments of procuring individual-level data for research projects from authors have had success rates vary significantly and were generally low at 58% [21], 46% [22], 14% [23], and 0% [24]. Some researchers note that getting access to datasets from authors can take from 4 months to 4 years [24].

To be able to manage privacy risks when sharing health data, in the context of the COVID-19 pandemic and more broadly to share such data with the research community, it is necessary to be able to measure the re-identification risk of the dataset being shared and ensure that it is small. For example, under the US HIPAA Privacy Rule Expert Determination method, the risk needs to be “very small” that the information could be used, alone or in combination with other reasonably available information, by an anticipated recipient, to identify an individual [25]. Recent guidance from the Ontario Information and Privacy Commissioner’s Office indicates that the risk of re-identification of individuals in data should be determined to be “very low” or “very small” prior to the data being released [26].

In Canada and the European Union, the reasonableness standard is widely used to judge whether or not information is identifiable [27–30]. For instance, in the Federal Court of Canada case of *Gordon v*. *Canada (Health)* [31], the judge adopted the “serious possibility” test proposed by the Federal Privacy Commissioner to determine if information constitutes personal information as defined in the *Privacy Act* (i.e., whether it is information about an identifiable individual).

Recital 26 of the European General Data Protection Regulation states, “To determine whether a natural person is identifiable, account should be taken of all the means reasonably likely to be used, such as singling out, either by the controller or by another person to identify the natural person directly or indirectly.” [29] The EU Article 29 Working Party Opinion on Anonymization Techniques indicates that anonymized data must meet three specific criteria (must not allow for singling out, linkability or inference) or a re-identification risk analysis must be performed to demonstrate that the risk is “acceptably small” [32].

To enable the precise and repeatable assessment of terms such as “reasonable”, “reasonably likely”, “serious possibility”, “very low”, “very small”, or “acceptably small”, quantitative measures of risk are necessary. By measuring re-identification risk, a data custodian can apply various algorithms to reduce that risk to an acceptable value. Absent reliable measurement, it will be difficult to know whether the re-identification risk meets any of these standards, and whether methods for the de-identification of health data are adequate or not.

Re-identification risk is defined as the probability of an adversary correctly matching a record in the dataset with a real person. A large body of work has been developed in the disclosure control literature to estimate this parameter [33–39]. Our main focus is the estimation of identity disclosure risk for datasets rather than for individual records in a dataset. If a dataset is deemed to have a low risk, then it can be treated as non-personal information and can be used and disclosed without additional patient consent.

Many existing estimators in the literature do not model actual adversarial attacks, and therefore provide only a proxy for risk. One of the more common ways to assess re-identification risk is to measure population uniqueness [40]. For example, an accurate estimator of uniqueness uses a log-linear model [41]. However, managing population uniqueness in a dataset does not provide sufficient assurances. Population uniqueness does not model a specific adversary attack scenario, plus it is quite easy to have data with low population uniqueness but still have a quite high probability of re-identification. For example, a dataset with zero population uniqueness value could have a mean or maximum probability of re-identifying a record as high as 0.5, which is much higher than the 0.09 commonly recommended threshold by data custodians [42–46], and used by the European Medicines Agency [47,48] and Health Canada [49] (also see the literature review of thresholds in [50]). Therefore, it is important to move beyond uniqueness as a risk measure and employ models that are more aligned with attack scenarios: managing re-identification risk based on uniqueness will not ensure that the overall data risk is acceptably small.

In this paper we present a new estimator of re-identification probability that is based on synthetic data generation methods, and that models a specific adversarial attack scenario. The method is an average of estimates from a Gaussian copula and a d-vine copula. Our average estimator gives the probability that a random record selected from a microdata sample can be correctly matched to a record (or individual) in the population from which the sample comes from.

We empirically evaluate the copula methods on four datasets and compare their overall accuracy to three other estimators that have already appeared in the literature. Our results demonstrate very high re-identification risk estimation accuracy for the average of the two copula methods, and that it is better than alternative estimators in the literature. A post-hoc sensitivity analysis was also performed to evaluate its performance when there are errors in its input parameters, and this provides additional specific application heuristics. We then applied this estimator to assess and de-identify a behavioral COVID-19 survey dataset of Ontario residents, which is now available through Physionet.

Our contribution is that this new average copula method provides a very accurate estimate of the risk of re-identification by matching a microdata sample record with the population, and its performance is better than other methods described in the literature. It can be used to enable sharing of COVID-19 and other datasets while providing reasonable privacy assurances.

## 2. Methods

Our ultimate objective is to be able to estimate re-identification risk for a dataset that is being shared. We will refer to this dataset as the microdata sample. The general assumption we make is that the data to be shared is a sample from some population, which is going to be the case in the majority of situations in practice.

In this section we first describe our estimation method. We then describe the empirical evaluation where we perform a simulation to compare six methods for the estimation of re-identification risk. Three of these are the ones we propose and three are the baseline methods proposed in the literature.

### 2.1 Context

Having an accurate estimator of re-identification risk will ensure that data is not excessively perturbed during de-identification, and that when a data controller claims that the risk of re-identification is low then it is indeed low. The former affects the ability of organizations to use health data for secondary purposes and the latter affects the ability of organizations to meet their regulatory obligations and avoid potentially large penalties for inappropriately processing personal health information.

For example, consider the situation where the actual re-identification risk of a dataset is 0.2 (i.e., the probability of a microdata sample record being correctly matched to a real person in the population is 0.2). And let the acceptable risk threshold be 0.05. If an estimator estimated that the risk is 0.02 (an underestimation of the true risk) then the data controller would incorrectly treat the data as de-identified. The obligations of the data controller when processing de-identified data are quite limited under, for example, the GDPR [51]. This means that the data controller would be in breach of the regulation and potentially subject to significant fines.

On the other hand, if the true risk was 0.02 and the estimated risk was 0.2 (an overestimation of the true risk), then the data controller would unnecessarily perturb the dataset to reduce its risk to a value below the threshold. This would result in lower utility data that would reduce the ability to derive meaningful results from the data. If the utility is too low due to excessive perturbation the data controller may not be able to use the data at all for secondary analysis.

Therefore, accurate risk estimation is important to ensure the responsible use of health data for secondary purposes using de-identification.

### 2.2 Assumptions and notation

When an adversary is attempting to match records in a dataset to individuals in the population, she does so using a subset of the variables in the dataset that are knowable. This subset if called the *quasi-identifiers* [33]. Examples of quasi-identifiers are date of birth, gender, race, main language spoken, and level of education. These are knowable because they are likely to be known by an acquaintance of someone in the dataset or they exist in public registries, such as voter registration lists [52].

All the records that have the same values on the quasi-identifiers are called an equivalence class. If an equivalence class size is equal to one, then it is a unique record. Equivalence classes sizes can be computed from the microdata sample or from the population.

In defining our risk measure and its estimators, we will use the notation below:

**Table:** 

Notation	Interpretation
*D*_*r*_	Microdata sample dataset
*D*_*p*_	Synthetic population dataset
*D*_*s*_	Synthetic microdata dataset, sampled from the synthetic population dataset *D*_*p*_
*k*	Index for records in the microdata sample
*n*	Number of records in the microdata sample
*N*	Number of records in the population
*f*_*k*_	Size of the equivalence class (in the microdata sample) that record *k* belongs to
*F*_*k*_	Size of the equivalence class (in the population) that record *k* belongs to
*A*	Match rate for population-to-sample attacks
*B*	Match rate for sample-to-population attacks

### 2.3 Measuring re-identification risk

We consider two risk metrics that are modeled after specific attacks [33]. We will refer to these as population-to-sample attacks and sample-to-population attacks.

In the first, the attacker selects an individual from the population and tries to match that individual on the quasi-identifiers with records in the dataset. The match rate for such population-to-sample attacks is given by [33] (the derivation of formula (1) is provided in the appendix):

$$A=\frac{1}{N}\sum_{k=1}^{n}\frac{1}{f_{k}}$$ (1)

This gives the probability that a random individual selected from the population can be correctly matched with their record in the microdata sample. A selected individual from the population may not be in the microdata sample, and therefore the fact that the microdata is a sample does have a protective effect.

With the second method of attack, the sample-to-population attack, the adversary randomly selects a record from the sample and matches it to individuals in the population. The match rate is given by [33] (the derivation of formula (2) is provided in the appendix):

$$B=\frac{1}{n}\sum_{k=1}^{n}\frac{1}{F_{k}}$$ (2)

This gives the probability that a random individual selected from the microdata sample can be correctly matched with their record (or person) in the population.

These risk values are not conditional on whether an equivalence class is unique in the real dataset or not.

The parameters in Eq (1) can be easily computed from the microdata sample directly and using knowledge of the population size (i.e., the value of *N*, which we assume is known). However, the parameters in Eq (2) must be estimated because *F*_*k*_ is unknown. Our objective in this study is to estimate $\hat{B}$ in Eq (2) using microdata sample information only.

### 2.4 Previous estimators

A number of approaches have been proposed to estimate the population equivalence group size value, ${\hat{F}}_{k}$ or 1F^k.

One approach that can be used to approximate $\hat{B}$ uses microdata sample entropy [53]. This method assumes that the quasi-identifiers are independent, and computes the probability that a specific set of quasi-identifier values appear in the population.

The Benedetti-Franconi estimator assesses the risk following a Bayesian approach [37]. The method provides an estimate of risk for individual records and can be aggregated across all records to obtain an expected dataset risk value.

An earlier estimator of sample dataset risk was proposed by El Emam and Dankar [54]. This method uses a hypothesis testing approach assuming that equivalence group sizes in the population follow a truncated-at-zero Poisson distribution.

These three estimators are used as a baseline to compare our proposed methods against.

### 2.5 Proposed estimator

Our proposed estimator is an average of two copula methods: each of them is used to make a separate estimate and then we take the average.

Let the microdata sample be *D*_*r*_. We use this microdata sample to create a synthetic variant which is the same size as the population (i.e., with *N* records). The synthesis is only performed on the quasi-identifiers. The synthetic population dataset is denoted by *D*_*p*_. We then sample from the synthetic population *D*_*p*_ a dataset of size *n* using simple random sampling, which we denote as *D*_*s*_. This is a synthetic sample from the synthetic population. We then compute the $\hat{B}$ value in Eq (2) using *D*_*p*_ and *D*_*s*_.

In this process we are simulating the population using data synthesis, and then drawing a synthetic microdata sample from that synthetic population to get a simulated microdata sample. These two synthetic datasets are then used to compute our estimator.

### 2.6 Synthesis using copulas

We use copulas to synthesize the population [55]. Copulas are flexible models that link univariate marginal distributions to form a multivariate distribution. This multivariate distribution captures the dependence structure among variables [56]. These models can be used to simplify the modelling and sampling of multivariate data. Copula models have been used to model financial time series [57–59], survival data [60,61], as well as for synthetic data generation [62–65]. We examine two different copulas, a Gaussian copula and a vine copula. The step-by-step procedure for fitting each of the two copulas is described in detail in the appendices.

A Gaussian copula models a series of variables as an m-dimensional multivariate Gaussian distribution. One benefit of using a Gaussian copula rather than a traditional multivariate normal distribution is that each variable does not need to be normally distributed initially. In our work, each variable has an empirical cumulative density function fit using the sample data which is then transformed to the standard normal using the inverse quantile function for the standard normal distribution. This means that this model does not make a strong distributional assumption about the data it models. Synthetic population samples are generated from sampling from the m-dimensional multivariate Gaussian distribution, then applying the standard normal quantile function and the inverse of the empirical cumulative density function to each dimension, yielding a synthetic sample in the original data space. The dependence between two variables *i* and *j* is estimated by the correlation coefficient *ρ*_*ij*_ where the optimal value for *ρ*_*ij*_ is the value which minimizes the difference between the mutual information in the real data sample and the mutual information in a synthetic data sample.

The d-vine copula models m variables using a series of pairwise bivariate Gaussian copulas where the pattern of bivariate copulas is described by a vine structure. A vine structure is a hierarchy of trees that describe conditional dependence between variables. This modelling strategy is particularly effective at modelling more complicated dependence relationships than the Gaussian copula.

### 2.7 Datasets used

The four datasets that we used in our simulations are summarized in Table 1. These datasets were selected to represent common contemporary health data and a common benchmark dataset. The specific quasi-identifiers that were used are summarized in the appendix, as well as links to the sources of these datasets.

**Table 1:** A summary of the datasets that were used in our simulation. These datasets represent the population that were used in our simulation.

Name	Description	Number of Records
Adult dataset	UCI Machine Learning Repository Adults dataset; this dataset is included as a reference point since it is often used in the machine learning and disclosure control community	48,842
Texas hospitals 2007 dataset	The Texas hospital discharge dataset (public dataset from the Texas Department of Health and Social Services)	50,000 records selected from the original 2,244,997 records
Washington 2007 hospitals dataset	The Washington state hospital discharge dataset	50,000 records selected from the original 644,902 records
Nexoid dataset	An on-line survey on COVID-19 exposure	50,000 records selected from the original 968,408 records

In total we evaluated six estimators as noted below as well as the names given to them in the results: the entropy estimator [53] (entropy), the hypothesis testing estimator [54] (hypothesis test), the Benedetti-Franconi estimator [37] (Italian), the Gaussian copula estimator (gaussian copula), the d-vine copula (d-vine copula), and the average of these two latter copula estimators (average).

### 2.8 Simulation

The simulation varied the selected quasi-identifiers and the sampling fraction. The number of quasi-identifiers was varied and a subset of variables of that number was selected as the quasi-identifiers. For example, if there were m quasi-identifiers, then a random number of quasi-identifiers from 1 to m was selected for each simulation run. The variation in the number of quasi-identifiers allows us to model datasets of different complexity.

Within the same simulation run a sampling fraction between 0.01 and 0.99 was selected (drawn from a uniform distribution). The variation in sampling fraction captures different situations where datasets may cover most of the population (e.g., datasets under single payor systems), and cases where datasets are for a subset of patients from a specific clinic where the population sampling fraction can be small. That way a more complete assessment of the behavior of the estimators will be possible.

For each subset of quasi-identifiers randomly selected we create a new sub-sample and generate a new synthetic dataset to compute the risk from. The synthetic datasets were generated using each of the two copula methods. A total of 1000 study points were generated for each data synthesis method.

The full dataset size (as noted in Table 1) that was sampled from was used as the population size (the *N* value). The estimate error was computed as $\hat{B}-B$. This allowed us to evaluate the extent of over and under estimation. We then plotted the error against the true risk value *B*. Given that there were multiple simulations per true risk value *B*, a box-and-whisker plot was used to represent the error median and variation at each true risk value. Deviation from zero indicates the extent of estimate error.

The descriptive visualization for presenting the results was not accompanied by formal statistical tests to compare the error at different true risk values *B* because in a simulation context such testing is not informative. One can just increase the number of simulation study points to get statistically significant differences across the board. Therefore, the useful information is the median error and the variation of the error (in the form of the inter-quartile range).

It is noted that the Texas, Washington, and Nexoid datasets are larger than what we used in our analysis. We therefore evaluated the impact of using a population larger than 50,000 in our simulations. We found that increasing the size of the population did not have a material impact on our results and conclusions across all the estimation methods. Therefore, we only present the results with the 50,000 population.

### 2.9 Ethics

This project was approved by the CHEO Research Institute Research Ethics Board, protocol number CHEOREB# 20/96X.

## 3. Results

In the main body of the paper, we present the results for the Texas dataset and the Nexoid dataset for a low (0.05), medium (0.3), and high (0.7) sampling fraction. These are two quite different datasets and illustrate the range of results we obtained. All of the remaining results for the other sampling fractions and datasets are included in the supplementary appendices. These show the same patterns as the results included in the main body of the paper.

We plot the error against the true risk in 0.1 true risk increments. Some of the datasets do not have a high true risk because of the nature of the quasi-identifiers that are considered (i.e., the true risk does not necessarily go all the way up to 1), and therefore we present their values to the highest true risk achievable.

**Fig 1:**
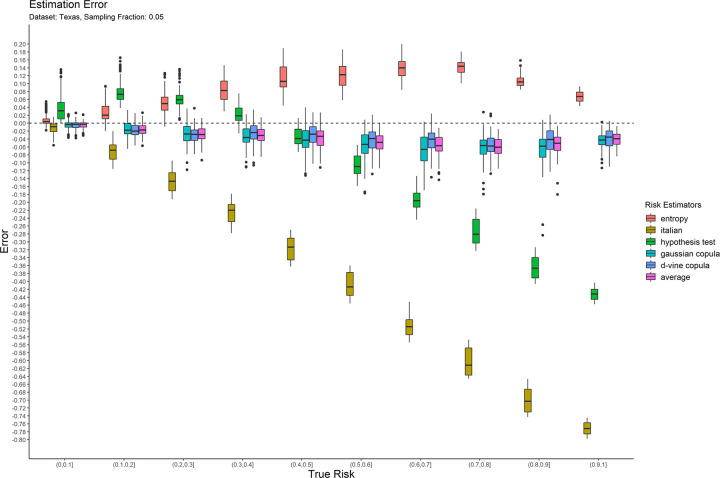
The estimation error for the Texas hospitals 2007 dataset at the 0.05 sampling fraction.

**Fig 2:**
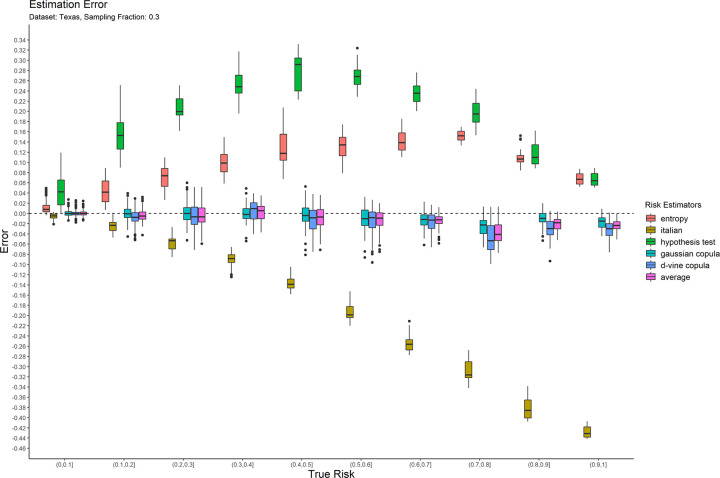
The estimation error for the Texas hospitals 2007 dataset at the 0.3 sampling fraction.

**Fig 3:**
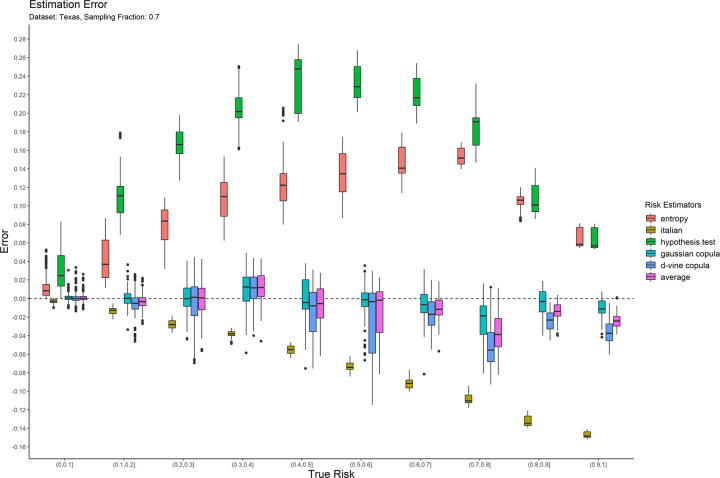
The estimation error for the Texas hospitals 2007 dataset at the 0.7 sampling fraction.

**Fig 4:**
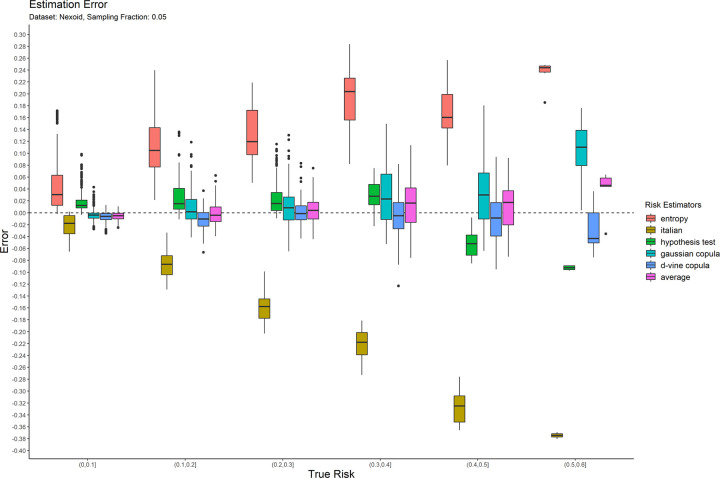
The estimation error for the Nexoid dataset at the 0.05 sampling fraction.

**Fig 5:**
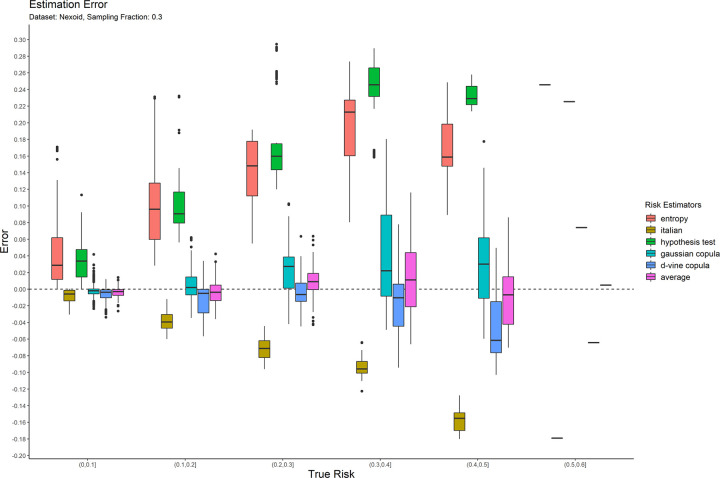
The estimation error for the Nexoid dataset at the 0.3 sampling fraction.

**Fig 6:**
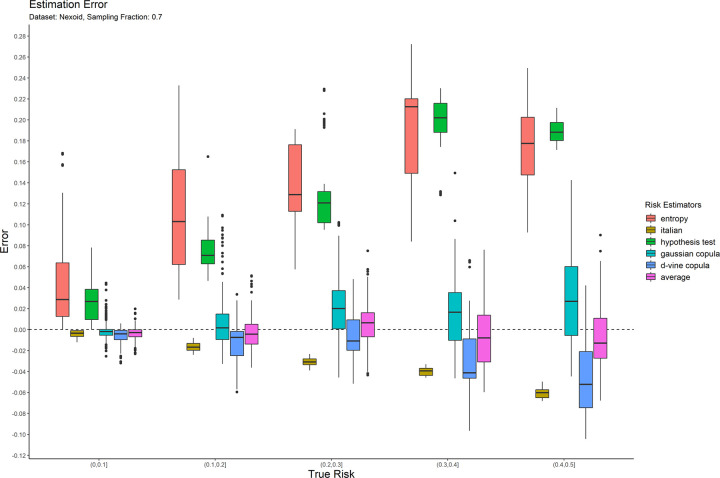
The estimation error for the Nexoid dataset at the 0.7 sampling fraction.

The error results for the 0.05 sampling fraction are shown in Fig 1. These are represented as box-and-whisker plots at each category of the true risk (on the x-axis). Similarly, the results are shown for the 0.3 and 0.7 sampling fractions in Figs 2 and 3 respectively. Equivalent results for the Nexoid dataset are shown in Figs 4–6 for the three sampling fractions respectively.

The main patterns are consistent across all of plots. We can make the following observations.

The entropy method consistently and significantly overestimates the true risk. This means that applications of that estimator in a de-identification context will be conservative resulting in more data transformations than necessary. Conservatism has a negative impact on data utility.

The Italian method consistently and significantly underestimates the true risk. This means that applications of that estimator in a de-identification context will be permissive resulting in datasets that have a higher re-identification risk than is assumed.

The hypothesis testing method underestimates the true risk for low sampling fractions and overestimates the risk for high sampling fractions. Even though its error is generally better than the previous two estimators, it is still quite high in practice when the full range of true risk is considered.

The Gaussian and d-vine copula estimators tend to perform well with quite small errors across the range of true risk values and sampling fractions. The average is better than either of the copula estimators in terms of maintaining an error less than 5% across the different datasets and sampling fractions. It is by far the best performing estimator out of the six that we have evaluated.

The average copula estimator provides an accurate estimate of re-identification risk that it would be practically useful for managing re-identification risk and being used as a reliable optimization criterion for de-identification algorithms. This estimator also performs better than the other methods in the literature. In particular, the average copula estimator provides the most accurate results around the commonly used 0.09 threshold, which is where such accuracy can make an important difference between whether a dataset is considered personal information or not.

## 4. Discussion and conclusions

The objective of this study was to develop and evaluate an estimator of re-identification risk that can be used to capture the match rate or the probability of re-identification success that is aligned with an actual attack (rather than a proxy). The specific attack we were modeling is the adversary selecting a record from the microdata sample and attempting to match that with a person in the real world using the quasi-identifiers.

### 4.1 Summary

Our general approach was to create a synthetic population from the microdata and then sample from the synthetic population to create a synthetic sample. Using the synthetic population and synthetic sample, we were able to compute the probability of a correct match under the sample-to-population attack. While the overall scheme we have proposed is relatively simple, it has not been applied before for re-identification risk estimation and the results demonstrate very good estimation accuracy. Therefore, there is incremental value from this result in terms of improving our ability to manage re-identification risk and enabling responsible sharing of health data for research and broader secondary analysis.

To synthesize the population, we evaluated two copula methods, a Gaussian copula and a d-vine copula, and an average of these two copula estimates. These were compared to three estimators in the literature: an entropy-based estimator, a Bayesian estimator, and a hypothesis testing estimator.

Our results show that the average of two copula methods produced the best results, with high accuracy of the estimated re-identification probability under the considered attack. This estimator performed better than all of the other methods in the literature that we considered. Furthermore, it had a consistently low error rate below 5% and small variation in the error on the datasets we included in our evaluation. This is a low error rate in an absolute sense and ensures reliable behavior across sampling fraction and true risk conditions.

Therefore, we propose the use of this average copula risk estimator as the basis for computing the re-identification risk for a sample-to-population attack.

### 4.2 Sensitivity analysis

The main parameter for the copula estimators is the population size, *N*. We performed a post-hoc sensitivity analysis to determine the extent to which the accuracy of the average copula method risk estimate is affected by errors in that value. This allows us to provide practical guidance on the application of the average copula estimator.

We only performed this sensitivity analysis on the average copula estimator because results on the other estimation methods would not affect our conclusions. If the other methods had a high sensitivity to the population size parameter, then there would be no reason to use them for that reason. Even if they had low sensitivity, their accuracy was still poor that we would not recommend using them. And further evaluation of these other estimators would not contribute to developing practical guidance for using the average copula estimator.

In practice, the exact population value may not be known with precision. We therefore computed the risk estimation error as the *N* value used in the synthesis is increased and decreased by up to 30%. This will tell us how much errors as large as 30% in the value of *N* influence accuracy. While the direction of the impact of changes in *N* can be anticipated in advance, the magnitude of that change is not known, and the objective was to empirically evaluate the magnitude of change.

**Fig 7:**
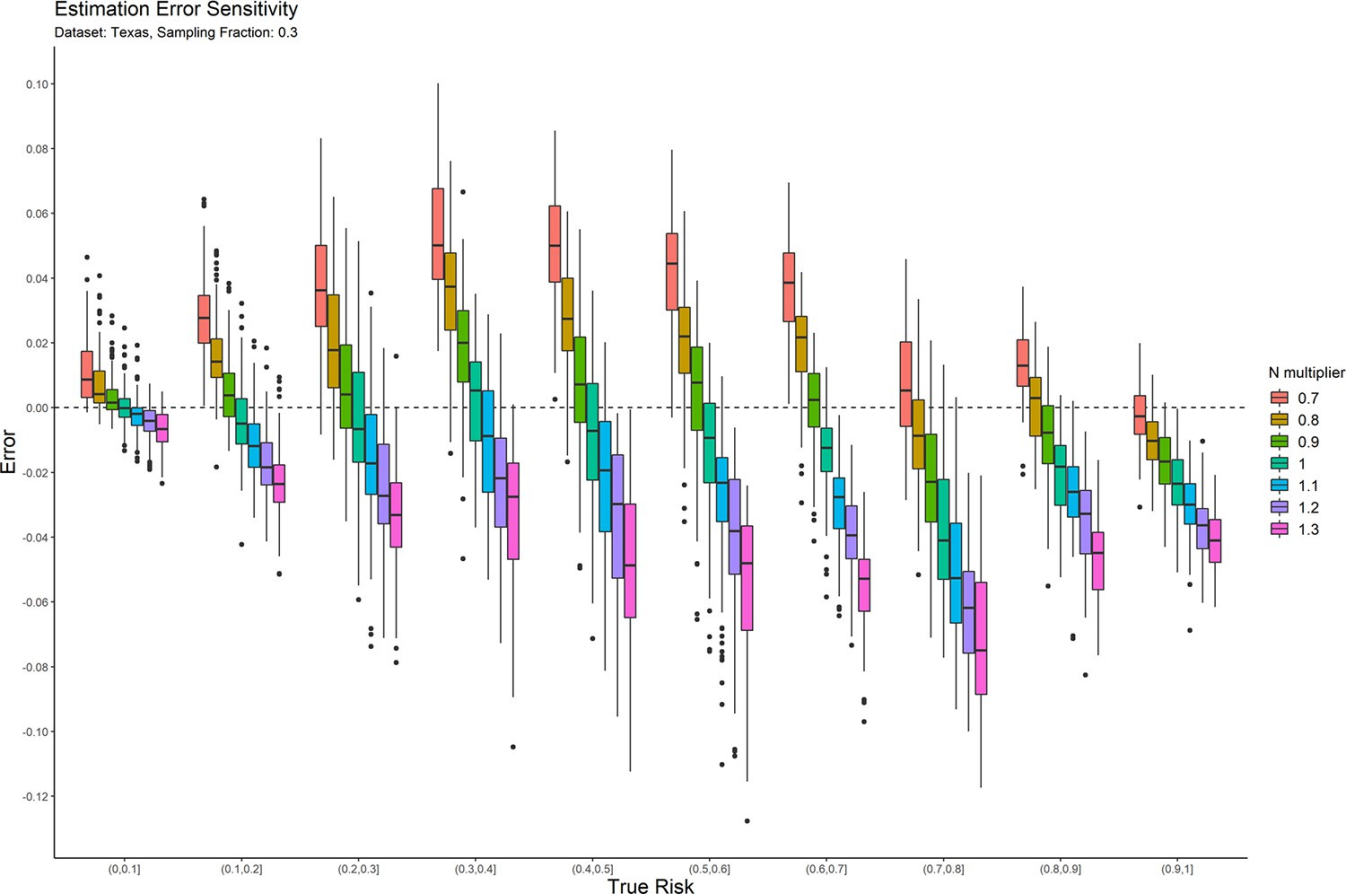
The sensitivity of the estimation error for the Texas dataset at the 0.3 sampling fraction.

**Fig 8:**
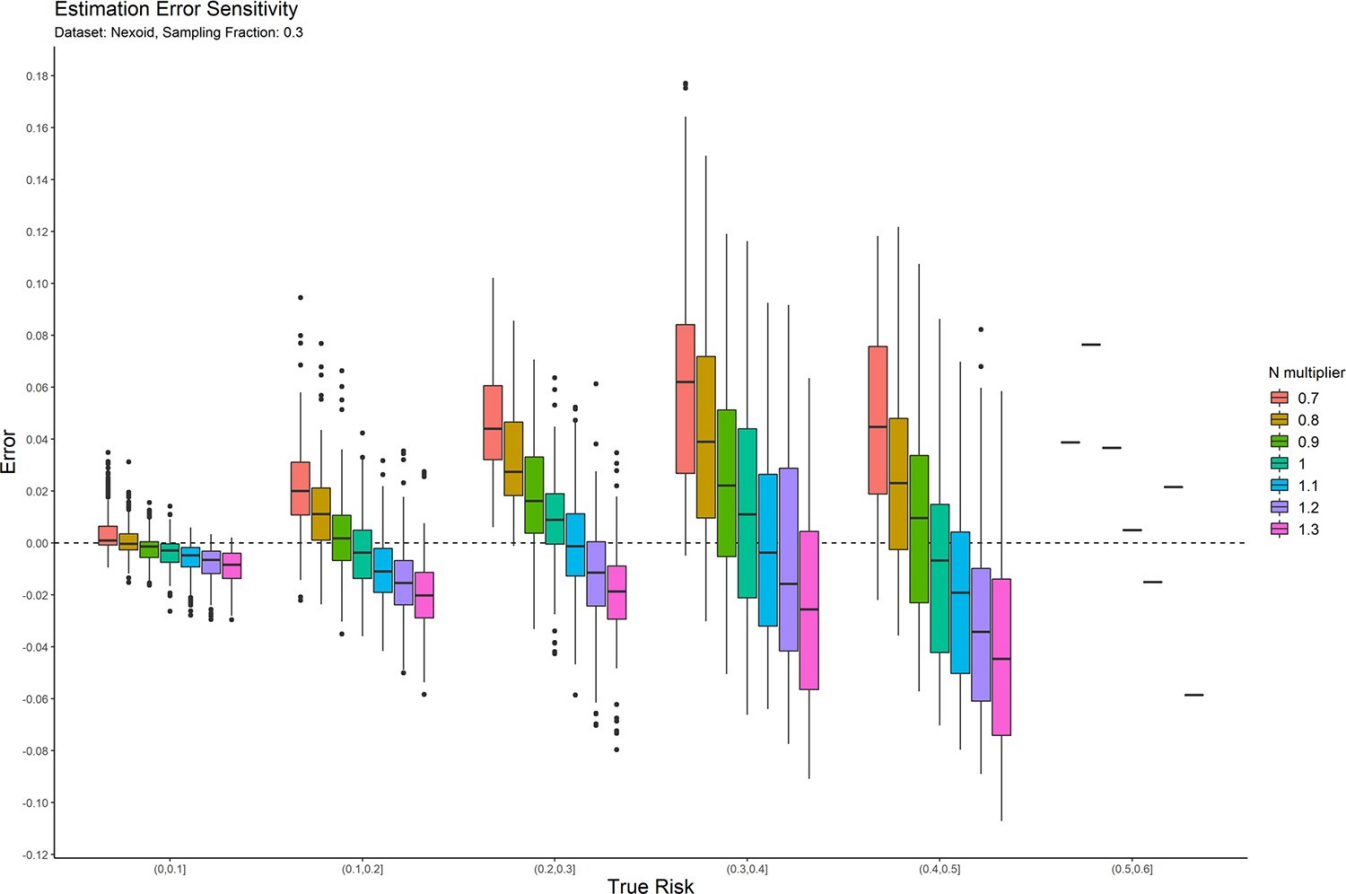
The sensitivity of the estimation error for the Nexoid dataset at the 0.3 sampling fraction.

The result plots of the sensitivity analysis for the average of two copulas are presented in Figs 7 and 8 for the 0.3 sampling fraction on the Texas and Nexoid datasets. The remaining plots are in the appendix. Having a value of N that is larger than the real value tends to result in underestimation of the risk and having a value of N that is smaller than the real value tends to result in overestimation of the risk. The over-/ under-estimation is within the 10% range. In general, when there is uncertainty around the true value of N, it is better to err on the side of using a value of N that may be smaller than the true value. This will result in a more conservative risk estimate and will reduce the likelihood of treating a dataset as a having a lower re-identification risk than the true risk, and therefore gives a little more privacy protection than what is necessary.

### 4.3 Application: Risk estimation for a COVID-19 dataset

The average copula estimator described in this paper was used to de-identify the flatten.ca dataset, which is currently available on Physionet [66]. Access to the data requires agreeing to certain terms-of-use.

This was data collected online from Ontario residents pertaining to their experiences with COVID-19. The Ontario dataset contained 18,903 observations, and the simulated population consisted of 13,448,494 individuals (the population of Ontario at the time). The quasi-identifiers are listed in Table 2. The sample-to-population risk was measured and the quasi-identifiers were generalized until the estimated risk was below the commonly used 0.09 threshold as noted in the introduction. The estimated sample-to-population risk after the adjustments described in Table 2 was 0.0723. The population-to-sample risk (see Eq (1)) was 0.0009 for the dataset generalized as described in Table 2.

**Table 2:** The quasi-identifiers and how they were modified to ensure a low risk of re-identification.

Variable	Generalizations
Date	Converted to month format
FSA	Forward Sortation Area, which is the first three characters of the postal code
Conditions	Medical conditions diagnosed
age_1	Age categories: <26, 26–44, 45–64, >65
travel_outside_canada	Travel outside Canada in the last 14 days (binary)
Ethnicity	
Sex	
tobacco_usage	
travel_work_school	
covid_results_date	Converted to month format
people_in_household	Removed

Beyond ensuring that the population-to-sample risk estimates were small, the dataset was mapped to Statistics Canada census data on the Forward Sortation Area [67]. Individuals in FSAs with small populations on the demographic quasi-identifiers (age, sex, and ethnicity) below a threshold of 11 were removed from the dataset. This provides additional conservative assurance for matches against population registries.

### 4.4 Future work

Other types of generative models can be used to improve on the average copula estimator in future work, or more complex copula designs can be used. The basic scheme of synthesizing a population and deriving a sample from it, to create both a synthetic population and synthetic sample, is a general recipe that has produced good results.

### 4.5 Limitations

In this analysis we made the assumption that the adversary would use exact matching to attack a microdata sample. This is a common assumption in the disclosure control literature when evaluating re-identification risk estimators and when modeling re-identification risk. Other forms of matching, such as approximate or probabilistic, may yield different results, and should be investigated further in future studies.

## Supporting information

S1 File (PDF)

S2 File (ZIP)

S3 File (ZIP)

## References

[1] Layne S., Hyman J., Morens D., and Taubenberger J., “New coronavirus outbreak: Framing questions for pandemic prevention,” *Science Translational Medicine*, vol. 12, no. 534, Mar. 2020, doi: 10.1126/scitranslmed.abb1469 32161107 PMC11000442

[2] Downey M., “Sharing data and research in a time of global pandemic,” *Duke University Libraries*, Mar. 17, 2020. https://blogs.library.duke.edu/bitstreams/2020/03/17/sharing-data-and-research-in-a-time-of-global-pandemic/ (accessed Apr. 08, 2020).

[3] Ng A., “Coronavirus pandemic changes how your privacy is protected,” *CNET*, Mar. 21, 2020. https://www.cnet.com/news/coronavirus-pandemic-changes-how-your-privacy-is-protected/ (accessed Apr. 08, 2020).

[4] Cosgriff C. V., Ebner D. K., and Celi L. A., “Data sharing in the era of COVID-19,” *The Lancet Digital Health*, vol. 2, no. 5, p. e224, May 2020, doi: 10.1016/S2589-7500(20)30082-0 32373785 PMC7194831

[5] Beam A. L. and Kohane I. S., “Big Data and Machine Learning in Health Care,” *JAMA*, vol. 319, no. 13, pp. 1317–1318, Apr. 2018, doi: 10.1001/jama.2017.18391 29532063

[6] Adly A. S., Adly A. S., and Adly M. S., “Approaches Based on Artificial Intelligence and the Internet of Intelligent Things to Prevent the Spread of COVID-19: Scoping Review,” *Journal of Medical Internet Research*, vol. 22, no. 8, p. e19104, 2020, doi: 10.2196/19104 32584780 PMC7423390

[7] Park S., Choi G. J., and Ko H., “Information Technology–Based Tracing Strategy in Response to COVID-19 in South Korea—Privacy Controversies,” *JAMA*, Apr. 2020, doi: 10.1001/jama.2020.6602 32324202

[8] Ienca M. and Vayena E., “On the responsible use of digital data to tackle the COVID-19 pandemic,” *Nature Medicine*, vol. 26, no. 4, Art. no. 4, Apr. 2020, doi: 10.1038/s41591-020-0832-5 32284619 PMC7100462

[9] Lewis P., Conn D., and Pegg D., “UK government using confidential patient data in coronavirus response,” *The Guardian*, Apr. 12, 2020. Accessed: May 08, 2020. [Online]. Available: https://www.theguardian.com/world/2020/apr/12/uk-government-using-confidential-patient-data-in-coronavirus-response

[10] Zastrow M., “South Korea is reporting intimate details of COVID-19 cases: has it helped?,” *Nature*, Mar. 2020, doi: 10.1038/d41586-020-00740-y 32203363

[11] Rocha R., “The data-driven pandemic: Information sharing with COVID-19 is ‘unprecedented,’” *CBC News*, Canada, Mar. 17, 2020. Accessed: Apr. 08, 2020. [Online]. Available: https://www.cbc.ca/news/canada/coronavirus-date-information-sharing-1.5500709.

[12] Rackley K., “DHEC, state authorities address privacy issues, information about coronavirus case specifics,” *Aiken Standard*, Aiken, SC, USA, Apr. 04, 2020. Accessed: Apr. 08, 2020. [Online]. Available: https://www.aikenstandard.com/coronavirus/dhec-state-authorities-address-privacy-issues-information-about-coronavirus-case-specifics/article_1358c0b6-7359-11ea-a5dc-d7fc40deaf22.html.

[13] Hinkle J., “Framingham one of several cities and towns told by DPH to limit information about residents who test positive for coronavirus,” *Wicked Local—News*, Mar. 28, 2020. https://www.wickedlocal.com/news/20200328/framingham-one-of-several-cities-and-towns-told-by-dph-to-limit-information-about-residents-who-test-positive-for-coronavirus (accessed Apr. 08, 2020).

[14] McCallum A., “Janesville and Rock County officials clash over sharing of COVID-19 information,” *GazetteXtra*, Janesville, Wisconsin, Apr. 05, 2020. Accessed: Apr. 08, 2020. [Online]. Available: https://www.gazettextra.com/news/health_care/coronavirus/janesville-and-rock-county-officials-clash-over-sharing-of-covid-19-information/article_b401c479-b9d2-51e0-b60d-8d0b6d667a28.html.

[15] Hancock L., “Ohio health director cites privacy concerns as local health departments withhold coronavirus details,” *cleveland.com*, Apr. 03, 2020. https://www.msn.com/en-us/news/us/ohio-health-director-cites-privacy-concerns-as-local-health-departments-withhold-coronavirus-details/ar-BB128Ztr (accessed Apr. 08, 2020).

[16] Hill K., “Spokane health officials providing more information about COVID-19 patients, but it remains unclear where they’re being treated,” *The Spokesman-Review*, Spokan, WA. Accessed: Apr. 08, 2020. [Online]. Available: https://www.spokesman.com/stories/2020/apr/05/spokane-health-officials-providing-more-informatio/.

[17] U. S. G. A. Office, “Artificial Intelligence in Health Care: Benefits and Challenges of Machine Learning in Drug Development [Reissued with revisions on Jan. 31, 2020.].” https://www.gao.gov/products/gao-20-215sp (accessed Jun. 21, 2021).

[18] Howe B., Stoyanovich J., Ping H., Herman B., and Gee M., “Synthetic Data for Social Good,” *arXiv:1710.08874 [cs]*, Oct. 2017, Accessed: Mar. 01, 2020. [Online]. Available: http://arxiv.org/abs/1710.08874.

[19] Rabes T. and ratana, “European data law is impeding studies on diabetes and Alzheimer’s, researchers warn,” *Science | AAAS*, Nov. 20, 2019. https://www.sciencemag.org/news/2019/11/european-data-law-impeding-studies-diabetes-and-alzheimer-s-researchers-warn (accessed Jun. 21, 2021).

[20] Lugg-Widger F. V. et al., “Challenges in accessing routinely collected data from multiple providers in the UK for primary studies: Managing the morass.,” *IJPDS*, vol. 3, no. 3, Art. no. 3, Sep. 2018, doi: 10.23889/ijpds.v3i3.432 34095522 PMC8142952

[21] Polanin J. R., “Efforts to retrieve individual participant data sets for use in a meta-analysis result in moderate data sharing but many data sets remain missing,” *Journal of Clinical Epidemiology*, vol. 98, pp. 157–159, Jun. 2018, doi: 10.1016/j.jclinepi.2017.12.014 29288135

[22] Naudet F. et al., “Data sharing and reanalysis of randomized controlled trials in leading biomedical journals with a full data sharing policy: survey of studies published in The BMJ and PLOS Medicine,” *BMJ*, vol. 360, Feb. 2018, doi: 10.1136/bmj.k400 29440066 PMC5809812

[23] Villain B., Dechartres A., Boyer P., and Ravaud P., “Feasibility of individual patient data meta-analyses in orthopaedic surgery,” *BMC Med*, vol. 13, no. 1, p. 131, Jun. 2015, doi: 10.1186/s12916-015-0376-6 26040278 PMC4464630

[24] Ventresca M. et al., “Obtaining and managing data sets for individual participant data meta-analysis: scoping review and practical guide,” *BMC Medical Research Methodology*, vol. 20, no. 1, p. 113, May 2020, doi: 10.1186/s12874-020-00964-6 32398016 PMC7218569

[25] Congress US, “The Health Insurance Portability and Accountability Act of 1996; 42 U.S. Code § 1320d - Definitions.” 1996. [Online]. Available: http://bit.ly/2zmpuRs.

[26] Information and Privacy Commissioner of Ontario, “De-identification Guidelines for Structured Data,” Jun. 2016. [Online]. Available: http://bit.ly/1PkrnMF.

[27] Government of Ontario, *Personal Health Information Protection Act*. 2004.

[28] Province of Alberta, *Health Information Act*. 2016, p. Chapter H-5. Accessed: Oct. 12, 2017. [Online]. Available: http://www.qp.alberta.ca/documents/Acts/H05.pdf.

[29] European Parliament and the Council of the European Union, *REGULATION (EU) NO 2016/679 OF THE EUROPEAN PARLIAMENT AND OF THE COUNCIL OF APRIL 27*, *2016*, *on the protection of individuals with regard to the processing of personal data and on the free movement of such data*, *and repealing Directive 95/46/EC (General Data Protection Regulation)*., vol. NO 2016/679. 2016. [Online]. Available: http://ec.europa.eu/justice/dataprotection/reform/files/regulation_oj_en.pdf.

[30] Province of New Brunswick, *Personal Health Information Privacy and Access Act*, *SNB* 2009, *c P-7*.*05*. Accessed: Oct. 12, 2017. [Online]. Available: https://www.canlii.org/en/nb/laws/stat/snb-2009-c-p-7.05/latest/snb-2009-c-p-7.05.html.

[31] Gibson J., *Mike Gordon v*. *The Minister of Health and Privacy Commissioner of Canada*. 2008.

[32] Article 29 Data Protection Working Party, “Opinion 05/2014 on Anonymization Techniques,” Apr. 2014.

[33] El Emam K., *Guide to the De-Identification of Personal Health Information*. CRC Press (Auerbach), 2013.

[34] Hundepool Anco et al., *Statistical Disclosure Control*. Wiley, 2012. Accessed: May 10, 2015. [Online]. Available: http://ca.wiley.com/WileyCDA/WileyTitle/productCd-1119978157.html.

[35] Hundepool Anco et al., “Handbook on Statistical Disclosure Control,” ESSNet SDC, 2010.

[36] Duncan G., Elliot M., and Salazar G., *Statistical Confidentiality—Principles and Practice*. Springer, 2011. Accessed: Oct. 05, 2014. [Online]. Available: http://www.springer.com/statistics/social+sciences+%26+law/book/978-1-4419-7801-1.

[37] Templ Matthias, “Statistical Disclosure Control for Microdata—Methods and Applications in R,” Aug. 24, 2018. https://www.springer.com/us/book/9783319502700 (accessed Aug. 24, 2018).

[38] Willenborg L. and de Waal T., *Statistical Disclosure Control in Practice*. New York: Springer-Verlag, 1996.

[39] Willenborg L. and de Waal T., *Elements of Statistical Disclosure Control*. New York: Springer-Verlag, 2001.

[40] Dankar F., El Emam K., Neisa A., and Roffey T., “Estimating the Re-identification Risk of Clinical Data Sets,” *BMC Medical Informatics and Decision Making*, vol. 12:66, 2012. doi: 10.1186/1472-6947-12-66 22776564 PMC3583146

[41] Skinner C. and Shlomo N., “Assessing Identification Risk in Survey Microdata Using Log-Linear Models,” *Journal of the American Statistical Association*, vol. 103, no. 483, pp. 989–1001, 2008, doi: 10.1198/016214507000001328

[42] CMS, “2008 Basic Stand Alone Medicare Claims Public Use Files.” http://go.cms.gov/2itDh2o.

[43] Erdem E. and Prada S. I., “Creation of public use files: lessons learned from the comparative effectiveness research public use files data pilot project,” Sep. 13, 2011. http://bit.ly/2xZKfyb (accessed Nov. 09, 2012).

[44] “Instructions for Completing the Limited Data Set ATA use Agreement (DUA) (CMS-R-0235L).” Department of Health & Human Services. [Online]. Available: http://go.cms.gov/2yJ1KX4.

[45] California Department of Health Care Services, “Public Reporting Guidelines.” https://www.dhcs.ca.gov/dataandstats/Pages/PublicReportingGuidelines.aspx (accessed May 23, 2020).

[46] State of Vermont Agency of Education, “Data Governance.” https://education.vermont.gov/data-and-reporting/data-governance (accessed May 23, 2020).

[47] European Medicines Agency, “External guidance on the implementation of the European Medicines Agency policy on the publication of clinical data for medicinal products for human use (v1.4),” Oct. 2018.

[48] European Medicines Agency, “European Medicines Agency policy on publication of data for medicinal products for human use: Policy 0070.” Oct. 02, 2014. [Online]. Available: http://www.ema.europa.eu/docs/en_GB/document_library/Other/2014/10/WC500174796.pdf.

[49] Health Canada, “Guidance document on Public Release of Clinical Information,” Apr. 01, 2019. https://www.canada.ca/en/health-canada/services/drug-health-product-review-approval/profile-public-release-clinical-information-guidance.html.

[50] El Emam K., Mosquera L., and Bass J., “Evaluating Identity Disclosure Risk in Fully Synthetic Health Data: Model Development and Validation,” *JMIR*, vol. 22, no. 11, Nov. 2020, Accessed: Oct. 13, 2020. [Online]. Available: https://www.jmir.org/2020/11/e23139. doi: 10.2196/23139 33196453 PMC7704280

[51] Hintze M. and El Emam K., “Comparing the benefits of pseudonymisation and anonymisation under the GDPR,” *Journal of Data Protection & Privacy*, vol. 2, no. 1, pp. 145–158, Dec. 2018.

[52] Benitez K. and Malin B., “Evaluating Re-Identification Risks with Respect to the HIPAA Privacy Rule,” *J Am Med Inform Assoc*, vol. 17, no. 2, pp. 169–177, Mar. 2010, doi: 10.1136/jamia.2009.000026 20190059 PMC3000773

[53] Erlich Y. and Narayanan A., “Routes for Breaching and Protecting Genetic Privacy,” *Nat Rev Genet*, vol. 15, no. 6, pp. 409–421, Jun. 2014, doi: 10.1038/nrg3723 24805122 PMC4151119

[54] El Emam K. and Dankar F., “Protecting Privacy Using k-Anonymity,” *Journal of the American Medical Informatics Association*, vol. 15, pp. 627–637, 2008. doi: 10.1197/jamia.M2716 18579830 PMC2528029

[55] Joe Harry, *Dependence Modeling with Copulas*. CRC Press, 2015.

[56] Nelsen R. B., Ed., *An Introduction to Copulas*. New York, NY: Springer, 2006. doi: 10.1007/0-387-28678-0_1

[57] Patton A. J., “A review of copula models for economic time series,” *Journal of Multivariate Analysis*, vol. 110, pp. 4–18, Sep. 2012, doi: 10.1016/j.jmva.2012.02.021

[58] Krupskii P. and Joe H., “Flexible copula models with dynamic dependence and application to financial data,” *Econometrics and Statistics*, vol. 16, pp. 148–167, Oct. 2020, doi: 10.1016/j.ecosta.2020.01.005

[59] Kayalar D. E., Küçüközmen C. C., and Selcuk-Kestel A. S., “The impact of crude oil prices on financial market indicators: copula approach,” *Energy Economics*, vol. 61, pp. 162–173, Jan. 2017, doi: 10.1016/j.eneco.2016.11.016

[60] Shih J. H. and Louis T. A., “Inferences on the Association Parameter in Copula Models for Bivariate Survival Data,” *Biometrics*, vol. 51, no. 4, pp. 1384–1399, 1995, doi: 10.2307/2533269 8589230

[61] Prenen L., Braekers R., and Duchateau L., “Extending the Archimedean copula methodology to model multivariate survival data grouped in clusters of variable size,” *Journal of the Royal Statistical Society*: *Series B (Statistical Methodology)*, vol. 79, no. 2, pp. 483–505, 2017, doi: 10.1111/rssb.12174

[62] Benali F., Bodénès D., Labroche N., and De Runz C., “MTCopula: Synthetic Complex Data Generation Using Copula,” in 23rd International Workshop on Design, Optimization, Languages and Analytical Processing of Big Data (DOLAP), Nicosia, Cyprus, 2021, pp. 51–60. Accessed: Jun. 21, 2021. [Online]. Available: https://hal.archives-ouvertes.fr/hal-03188317.

[63] Sun Y., Cuesta-Infante A., and Veeramachaneni K., “Learning Vine Copula Models for Synthetic Data Generation,” *AAAI*, vol. 33, no. 01, Art. no. 01, Jul. 2019, doi: 10.1609/aaai.v33i01.33015049

[64] Meyer D., Nagler T., and Hogan R. J., “Copula-based synthetic data generation for machine learning emulators in weather and climate: application to a simple radiation model,” *Geoscientific Model Development Discussions*, pp. 1–21, Jan. 2021, doi: 10.5194/gmd-2020-427

[65] Li H., Xiong L., Zhang L., and Jiang X., “DPSynthesizer: Differentially Private Data Synthesizer for Privacy Preserving Data Sharing,” *Proceedings VLDB Endowment*, vol. 7, no. 13, pp. 1677–1680, Aug. 2014, doi: 10.14778/2733004.2733059 26167358 PMC4496798

[66] Jain Shrey et al., “Flatten: COVID-19 Survey Data on Symptoms, Demographics and Mental Health in Canada.” PhysioNet. doi: 10.13026/V8EQ-8V80

[67] “DemoStats 2019,” Environics Analytics, 2019.

