# Supplementary material for: Measuring re-identification risk using a synthetic estimator to enable data sharing
Source: PLoS One. 2022 Jun 17;17(6):e0269097. doi: 10.1371/journal.pone.0269097 (PMC9205507; doi:10.1371/journal.pone.0269097)
Supplement: S1 File — (PDF) [file pone.0269097.s001.pdf]

Appendix A: Risk Formulas

(a) Derivation for formula (1)

The formula:

$$A = \frac{1}{N}\sum_{k=1}^{n}\frac{1}{f_k}$$

The Derivation:

A natural metric for measuring population-to-sample risk is the expected value of the probability that a population record is successfully matched to the corresponding record in the sample dataset.

Suppose the adversary chooses a record k in the population that belongs to an equivalence class of size F_k, and the record k belongs to the equivalence class of size f_k in the sample, with $0 \le f_k \le F_k$.

To archive a successful match, there are 2 necessary conditions:

1. $0 < f_k$

2. The record k must be inside of the f_k records in the sample.

Assuming equal probability of selection, the probability of conditions 2 can be computed as follow:

$$\frac{\binom{F_k - 1}{f_k - 1}}{\binom{F_k}{f_k}} = \frac{f_k}{F_k}$$

Given the 2 conditions have been satisfied, the probability of a successful match is $\frac{1}{f_k}$, again assuming equal probability of selection. Therefore, we come to the following formula:

$$
\begin{aligned}
A &= \frac{1}{N}\sum_{k=1, f_k \neq 0}^{N} \frac{f_k}{F_k} \times \frac{1}{f_k} \\
&= \frac{1}{N}\sum_{k=1, f_k \neq 0}^{N} \frac{1}{F_k} \\
&= \frac{1}{N}\sum_{\{k|f_k \neq 0\}} \frac{1}{F_k} \\
&= \frac{L - L_1}{N} \\
&= \frac{K}{N} \\
&= \frac{1}{N}\sum_{k=1}^{n} \frac{1}{f_k}
\end{aligned}
$$

where L is the number of equivalence classes in the population, L_1 is the number of equivalence classes in the population that do not have a corresponding equivalence class in the sample, and K is the number of equivalence classes in the sample.

(b): Derivation for formula (2)

The formula:

$$B = \frac{1}{n}\sum_{k=1}^{n} \frac{1}{F_k}$$

The Derivation:

A natural metric for measuring sample-to-population risk is the expected value of the probability that a sample record is successfully matched to the corresponding record in the population.

The derivation is simpler than for A since every sample record must exist in the population.

1. Assuming a sample record is uniformly randomly selected, the probability of selecting a sample record is $\frac{1}{n}$.
2. Suppose a sample record k is selected, assuming no additional information available, the probability of successful match is $\frac{1}{F_k}$

Therefore,

$$B = \sum_{k=1}^{n} \frac{1}{n} \times \frac{1}{F_k}$$
$$= \frac{1}{n} \sum_{k=1}^{n} \frac{1}{F_k}$$

Appendix B: Description of Copula Estimators

(a) Gaussian Copula Estimator

1. For each variable X_i, a marginal empirical distribution $\hat{F}_i$ was fitted.
2. Estimate the correlation matrix across all variables fitted in the first step as follows:
    a. The fitted marginal empirical cdf fitted in step 1 was applied to each variable $\hat{F}_i(X_i)$, and then the quantile function for the standard normal was applied, $\Phi^{-1}(\hat{F}_i(X_i))$.
    b. For each pair of variables X_i and X_j, we estimated the correlation parameter between these two variables using the following procedure:
        i. We choose the correlation parameter ρ_{ij} such that the following quantity is minimized.
        ii. Given a correlation parameter ρ_{ij}, we draw a sample of size n from the bivariate Gaussian distribution with mean vector $\mathbf{0}$ and covariance matrix

$$\begin{bmatrix} 1 & \rho_{ij} \\ \rho_{ij} & 1 \end{bmatrix}$$

        iii. Denote the sample by $(\hat{X}_i, \hat{X}_j)$, then we apply Φ and $\hat{F}_i^{-1} / \hat{F}_j^{-1}$ to the sample, $\left(\hat{F}_i^{-1}(\Phi(\hat{X}_i)), \hat{F}_j^{-1}(\Phi(\hat{X}_j))\right)$. We compute the empirical mutual information for these transformed quantities, $\hat{I}_{ij}$.
        iv. Compute the empirical mutual information for the original data (X_i, X_j), denoted by I_{ij}.
        v. We choose the parameter ρ_{ij} such that $(\hat{I}_{ij} - I_{ij})^2$ is minimized. This can be accomplished using an optimization method. In particular, the method we used is a combination of golden section search and successive parabolic interpolation [32].
    c. Repeat step (2.b) for every pair of variables.
    d. Once the $\frac{m(m-1)}{2}$ correlation parameters have been estimated (where m is the number of quasi-identifiers), we need make sure the correlation matrix we constructed is positive semi-definite. If the matrix is not positive semi-definite, the nearest (w.r.t sup norm) positive semi-definite matrix is chosen [33].
3. Once the correlation matrix is fitted from step (2). We can sample from the fitted gaussian copula distribution as follow,
    a. First, sample from a multivariate Gaussian distribution with mean vector $\mathbf{0}$ and the correlation matrix fitted in step (2), denote this sample by $(Y_i, \dots, Y_m)$

b. Then, apply standard normal cdf to the sample, $\left(\Phi(Y_i),\ldots,\Phi(Y_m)\right)$.

c. Lastly, we apply the fitted marginal quantile function, $\left(\hat{F}_i^{-1}(\Phi(Y_i)),\ldots,\hat{F}_m^{-1}(\Phi(Y_m))\right)$, to obtain the synthetic data values.

(b) D-vine Copula Estimator

Fitting the vine copula proceeds as follows:

1. For each variable X_i, a marginal empirical distribution $\hat{F}_i$ was fitted.
2. To model the dependence relations between variables, a vine copula approach is used. Instead of modeling a multivariate copula directly, a vine copula approach decomposes the multivariate copula into a sequence of bivariate copulas by conditioning on different variables. To fit a vine copula, it requires the specification of a vine structure and one bivariate distribution fitted for each edge of the vine structure.
   a. A vine structure consists of a collection of trees. The edges in the previous tree become the nodes for the next tree. A vine structure specifies how each pair of variables depends on other variables. To specify the vine structure, a regular vine (that is, a vine that satisfies certain regularity conditions) is required. There are many possible such vines. In our approach, the d-vine is used due to its simplicity. The diagram in Figure 1 is an example of a d-vine for 5 variables. Each edge in the diagram represents a bivariate relation. For example, the edge "12" in the first tree T_1 indicates we should model the dependence relation between variable 1 and variable 2 without conditioning on other variables. On the other hand, the edge "15|234" in the last tree T_4 indicates that we should model the dependence relation between variable 1 and variable 5 while conditioning on variable 2, 3 & 4.
   b. Once the vine structure is specified, a bivariate gaussian copula (which is characterized by its correlation parameter) is fitted for each edge as follow. For instance, consider the edge "13|2" in tree T_2,
      i. Applying the empirical CDF $\hat{F}_1, \hat{F}_3$ to variable X_1, X_3, we have $\hat{F}_1(X_1), \hat{F}_3(X_3)$.
      ii. We choose the correlation parameter $\rho_{13|2}$ such that the following quantity is minimized.
      iii. Given a correlation parameter $\rho_{13|2}$, we draw a sample of size n from the bivariate Gaussian distribution with mean vector $\mathbf{0}$ and covariance matrix

$$\begin{bmatrix} 1 & \rho_{13|2} \\ \rho_{13|2} & 1 \end{bmatrix}$$

$(\hat{X}_1, \hat{X}_3)$, then we apply Φ and $\hat{F}_1^{-1} / \hat{F}_3^{-1}$

$\left(\hat{F}_i^{-1}(\Phi(\hat{X}_i)), \hat{F}_j^{-1}(\Phi(\hat{X}_j))\right)$. We compute the empirical conditional mutual

information for these transformed quantities given the original variable X_2, denoted by $\hat{I}_{13|2}$.

v. Compute the empirical conditional mutual information for the original data (X_1, X_3) given X_2, denoted by $I_{13|2}$.

vi. We choose the parameter $\rho_{13|2}$ such that $(\hat{I}_{13|2} - I_{13|2})^2$ is minimized. This can be accomplished using an optimization method. In particular, the method we used is a combination of golden section search and successive parabolic interpolation [32].

c. After each $\frac{m(m-1)}{2}$ edge is fitted with a bivariate gaussian copula, our d-vine copula distribution is fitted.

3. We sample from the fitted multivariate distribution as follow,
   a. Draw a sample from the d-vine copula distribution fitted from step (2) using the d-vine sampling algorithm described in [34]. Denote this sample by $(U_1, \ldots, U_m)$.
   b. Apply the empirical marginal quantile function in step 1 to the sample, $\left(\hat{F}_1^{-1}(U_1), \ldots, \hat{F}_m^{-1}(U_m)\right)$, to obtain the synthetic data values.

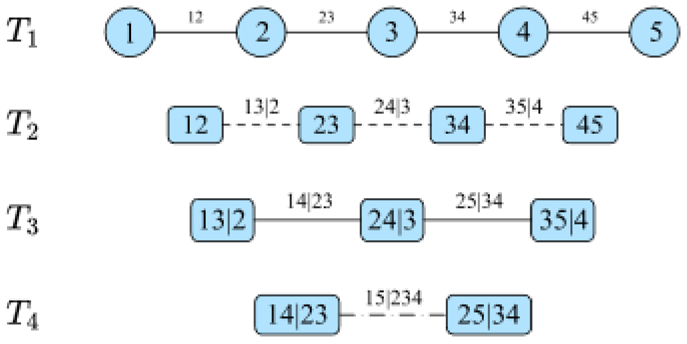

Figure 1: An illustration of the structure of a d-vine copula.

Appendix C: Dataset Summaries

The following are the quasi-identifiers that were used for each dataset, as well as the links to obtain the datasets that were used in our study.

1. For Adult dataset, all 11 variables were included.

Variables	Description
age	Age of the individual
workclass	Work status of the individual
education	Education level of the individual
marital_status	Marital status of the individual
occupation	Occupation of the individual
relationship	Type of relationship
race	Race of the individual
sex	Gender of the individual
native_country	Country of origin of the individual
capital	Capital gain obtained
income	Income level

This dataset is available from: https://archive.ics.uci.edu/ml/index.php

2. For the Texas 2007 hospitals dataset, 9 variables were used.

Variables	Description
DISCHARGE	Year and quarter of discharge.
PAT_STATE	State of the patient's mailing address in Texas and contiguous states.
PAT_COUNTRY	Country of patient's residential address.
COUNTY	FIPS code of patient's county.
SEX_CODE	Gender of the patient as recorded at date of admission or start of care.
ADMIT_WEEKDAY	Code indicating day of week patient is admitted
LENGTH_OF_STAY	Length of stay in days
PAT_AGE	Code indicating age of patient in days or years on date of discharge.
RACE	Code indicating the patient's race

This dataset is available from: https://www.dshs.texas.gov/thcic/hospitals/Inpatientpudf.shtm

3. For the Washington 2007 hospitals dataset, 9 variables were used.

Variables	Description
AGE	Age in years at admission
AGEDAY	Age in days (when age < 1 year)
AGEMONTH	Age in months (when age < 11 year)
PSTCO2	Patient state/country code, possibly derived from ZIP Code
ZIP	Patient ZIP Code
FEMALE	Indicator of sex
AYEAR	Admission year
AMOMTH	Admission month
AWEEKEND	Admission day is a weekend

This dataset is available from: https://www.hcup-us.ahrq.gov/sidoverview.jsp

4. For the Nexiod dataset, 8 variables were used.

**Variable**	**Description**
country	Country of origin of the individual
sex	Gender of the individual
age	Age of the individual
height	Height of the individual
weight	Weight of the individual
income	Income level of the individual
race	Race of the individual
immigrant	Immigrant status of the individual

This dataset is available from: https://www.covid19survivalcalculator.com/en/download

Appendix D: Complete Results from Simulations - Plots

The resulting plots from simulations are stored in the *results.zip* file. Inside the zip file, there are 4 folders storing the results for each datasets. These cover all of the sampling fractions that were included in the simulation.

1. “adults” folder: results for the Adult dataset.
    a. File named “comparison.adults.#.png” stores the sampling fraction 0.05*# comparison results between different risk estimators for the Adult dataset.
    b. File named “sensitivity.adults.#.png” stores the sampling fraction 0.05*# sensitivity results for the Adult dataset.
2. “tx” folder: results for the Texas hospitals 2007 dataset.
    a. File named “comparison.tx.#.png” stores the sampling fraction 0.05*# comparison results between different risk estimators for the Texas hospitals 2007 dataset.
    b. File named “sensitivity.tx.#.png” stores the sampling fraction 0.05*# sensitivity results for the Texas hospitals 2007 dataset.
3. “wa” folder: results for the Washington 2007 hospitals dataset.
    a. File named “comparison.wa.#.png” stores the sampling fraction 0.05*# comparison results between different risk estimators for the Washington 2007 hospitals dataset.
    b. File named “sensitivity.wa.#.png” stores the sampling fraction 0.05*# sensitivity results for the Washington 2007 hospitals dataset.
4. “nexoid” folder: results for the nexoid dataset.
    a. File named “comparison.nexoid.#.png” stores the sampling fraction 0.05*# comparison results between different risk estimators for the Nexoid dataset.
    b. File named “sensitivity.nexoid.#.png” stores the sampling fraction 0.05*# sensitivity results for the Nexoid dataset.

Appendix E: Complete Results from Simulations - Raw Data

The raw results from simulations are stored in the raw_*results.zip* file. Inside the zip file, there are 4 folders storing the results for each datasets.

1. "adults" folder: results for the Adult dataset.
2. "tx" folder: results for the Texas hospitals 2007 dataset.
3. "wa" folder: results for the Washington 2007 hospitals dataset.
4. "nexoid" folder: results for the nexoid dataset.

The different results for the average estimator in these tables pertain to the sensitivity analysis.
