# Supplementary figures and images for: Measuring re-identification risk using a synthetic estimator to enable data sharing (part 2 of 2)
Source: PLoS One. 2022 Jun 17;17(6):e0269097. doi: 10.1371/journal.pone.0269097 (PMC9205507; doi:10.1371/journal.pone.0269097)

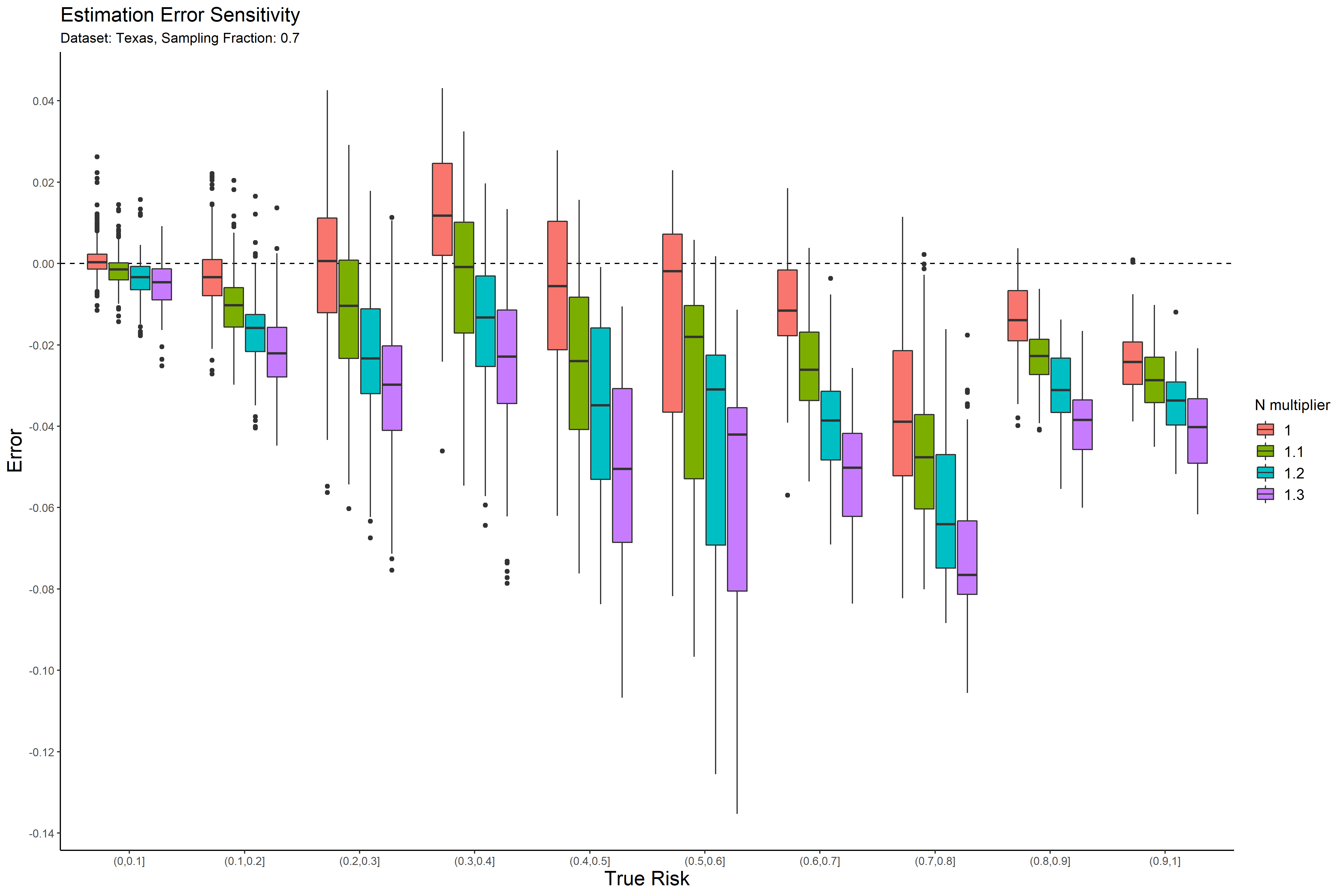

Supplement: S2 File — (ZIP) [file pone.0269097.s002.zip › tx/sensitivity.tx.14.png]

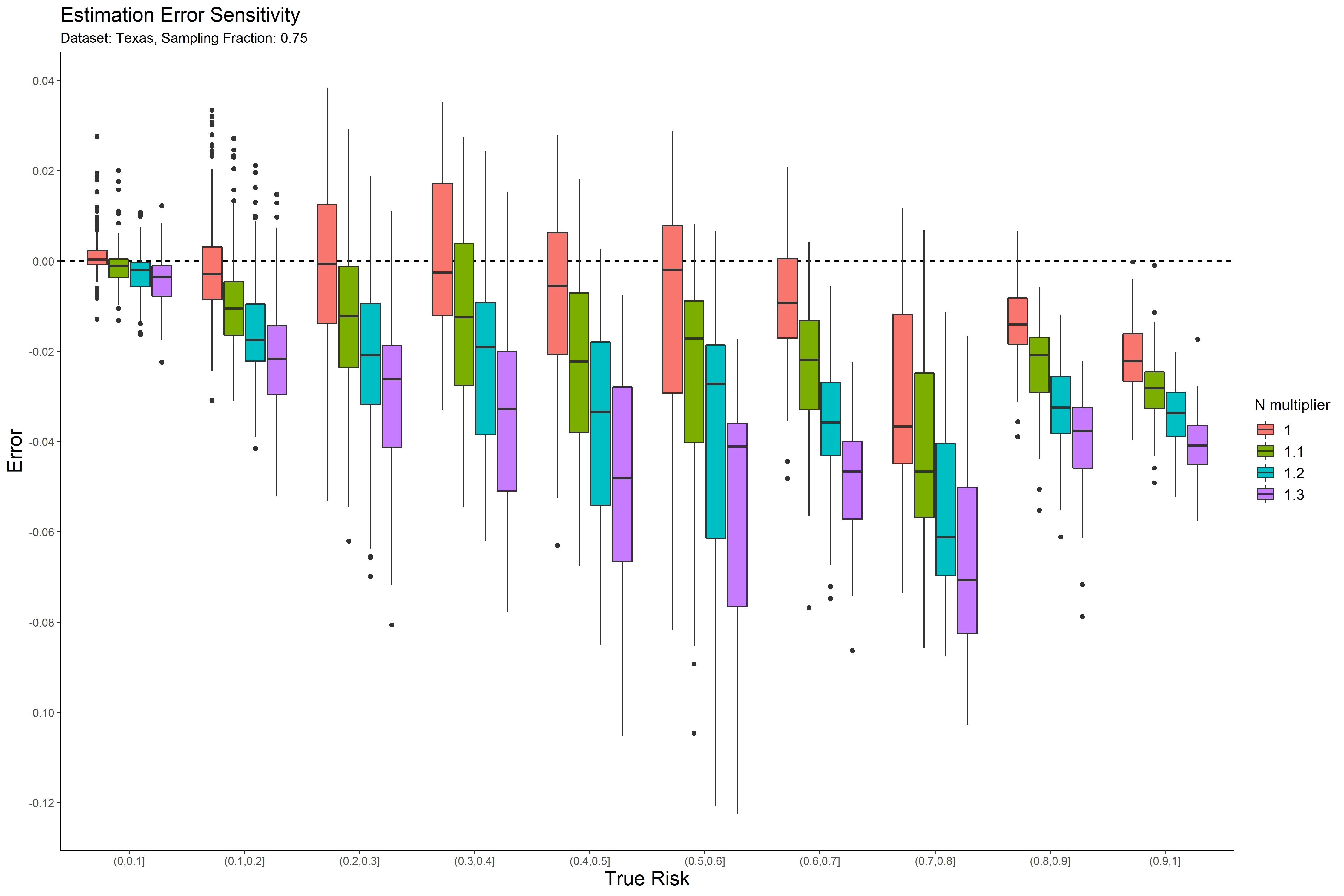

Supplement: S2 File — (ZIP) [file pone.0269097.s002.zip › tx/sensitivity.tx.15.png]

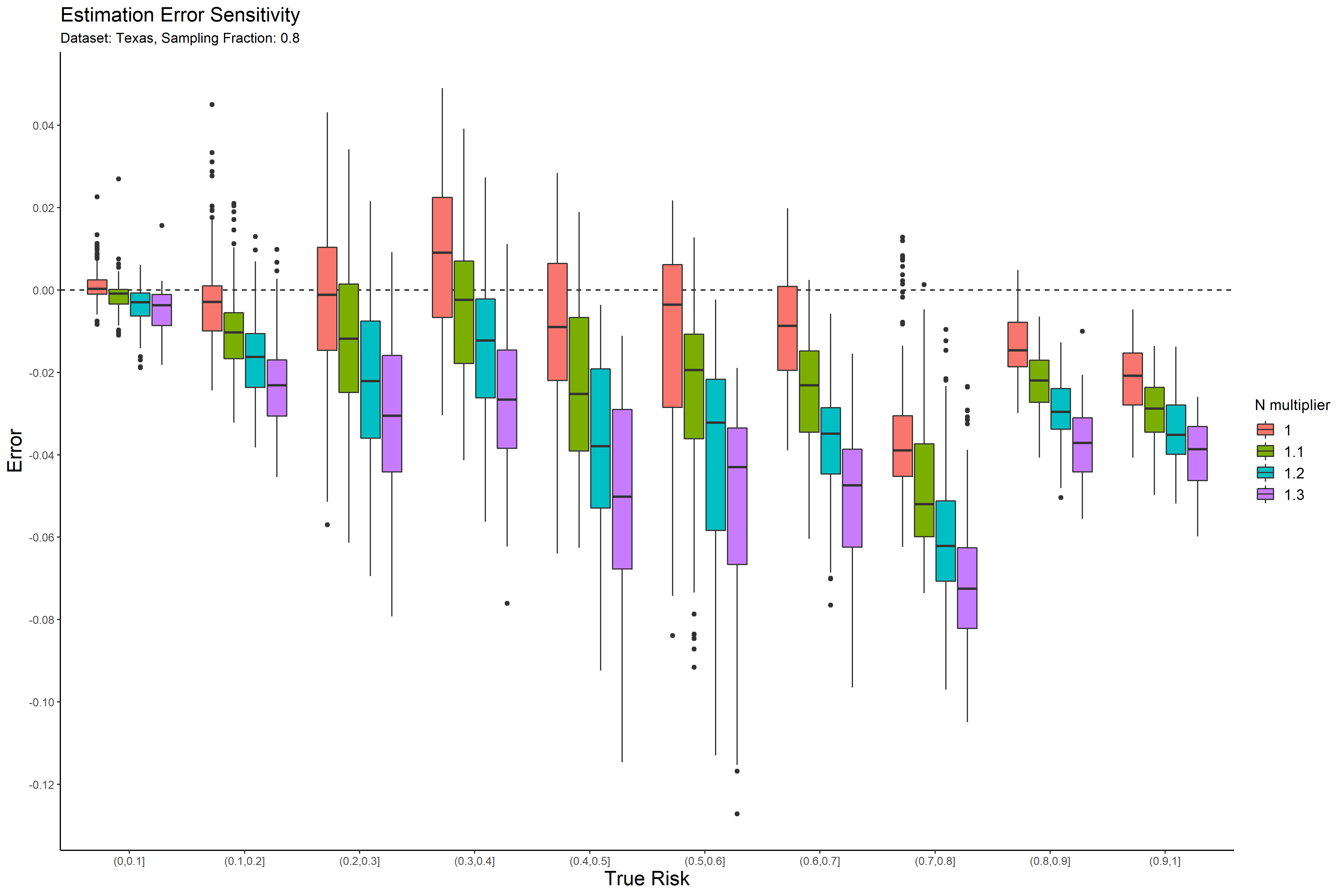

Supplement: S2 File — (ZIP) [file pone.0269097.s002.zip › tx/sensitivity.tx.16.png]

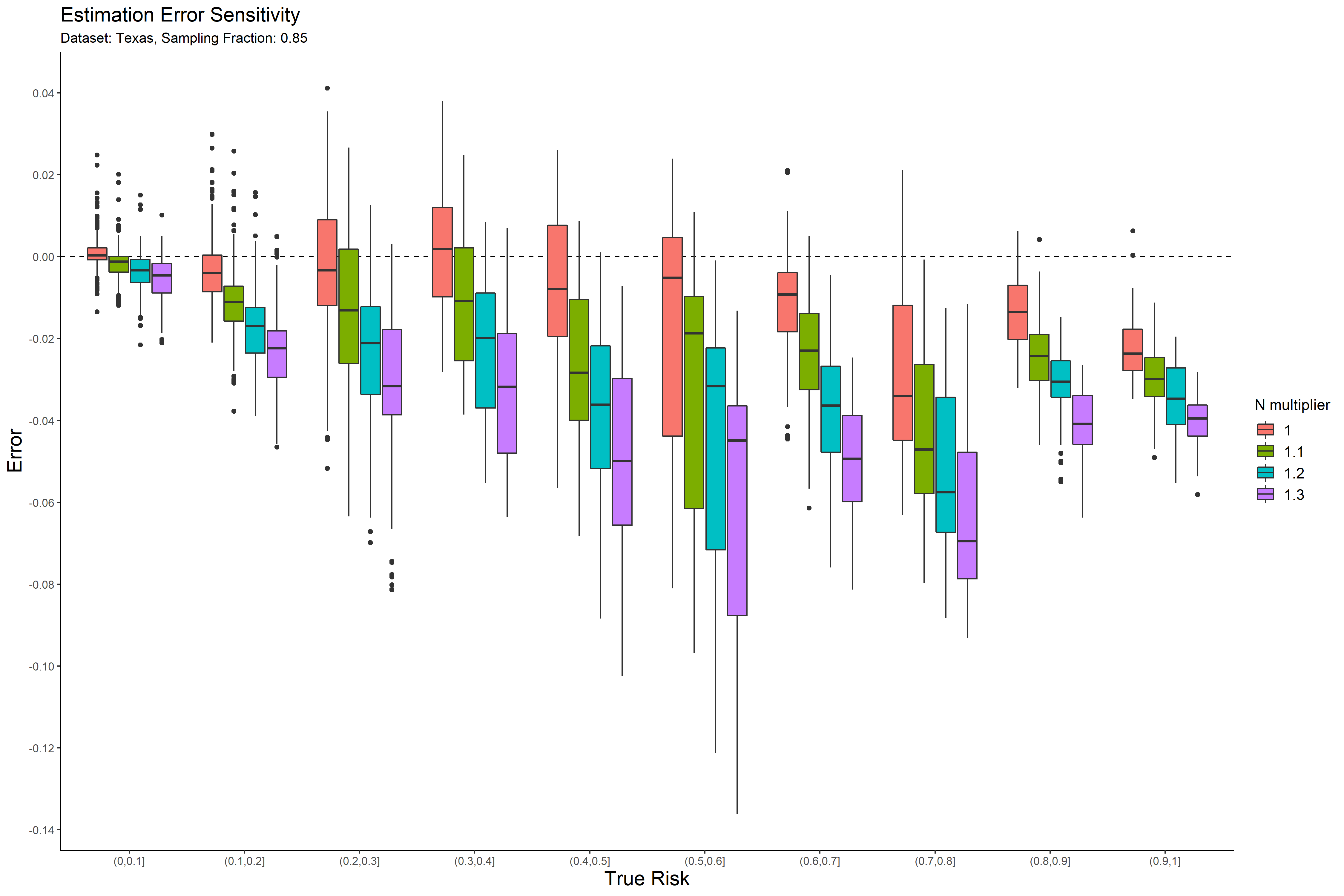

Supplement: S2 File — (ZIP) [file pone.0269097.s002.zip › tx/sensitivity.tx.17.png]

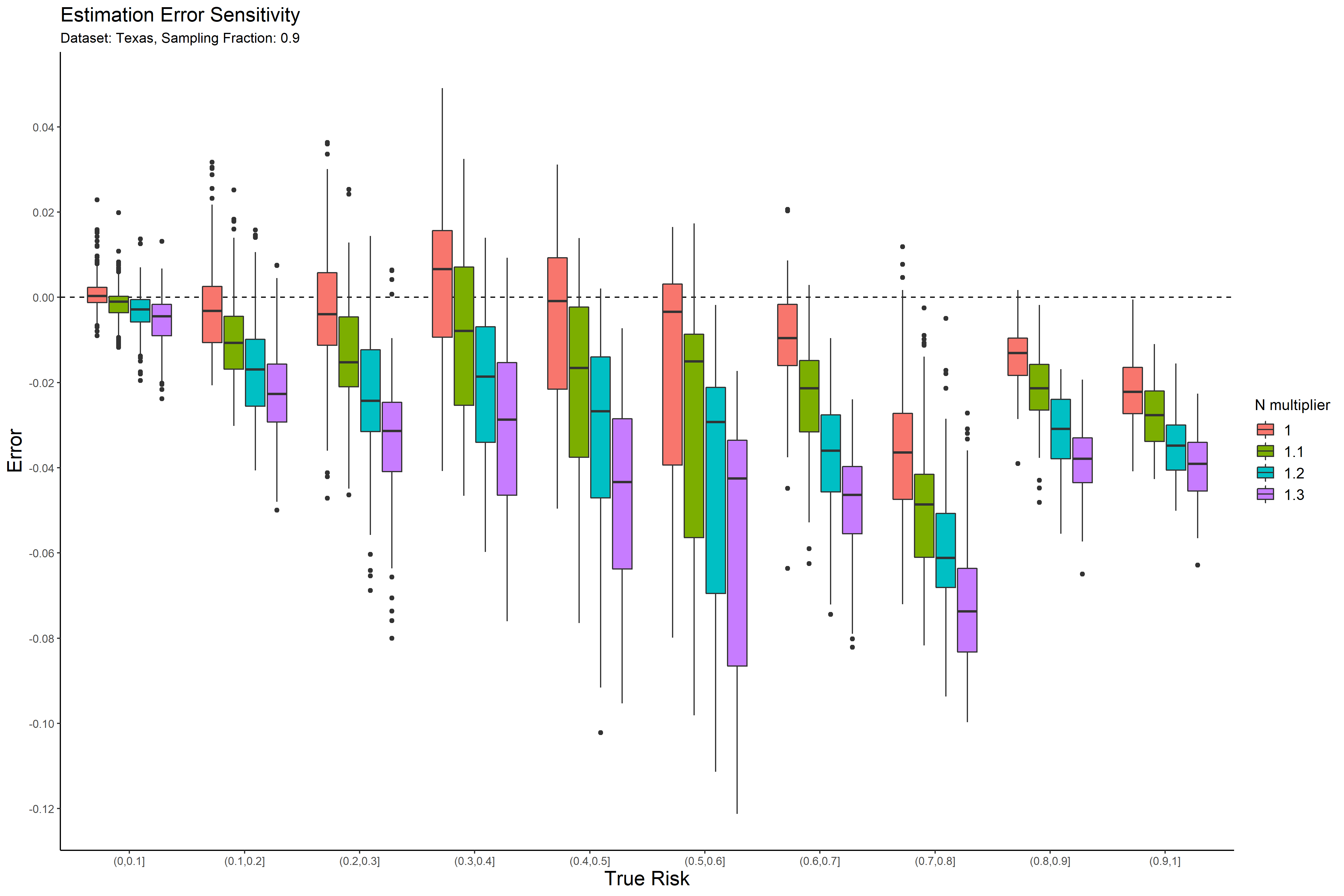

Supplement: S2 File — (ZIP) [file pone.0269097.s002.zip › tx/sensitivity.tx.18.png]

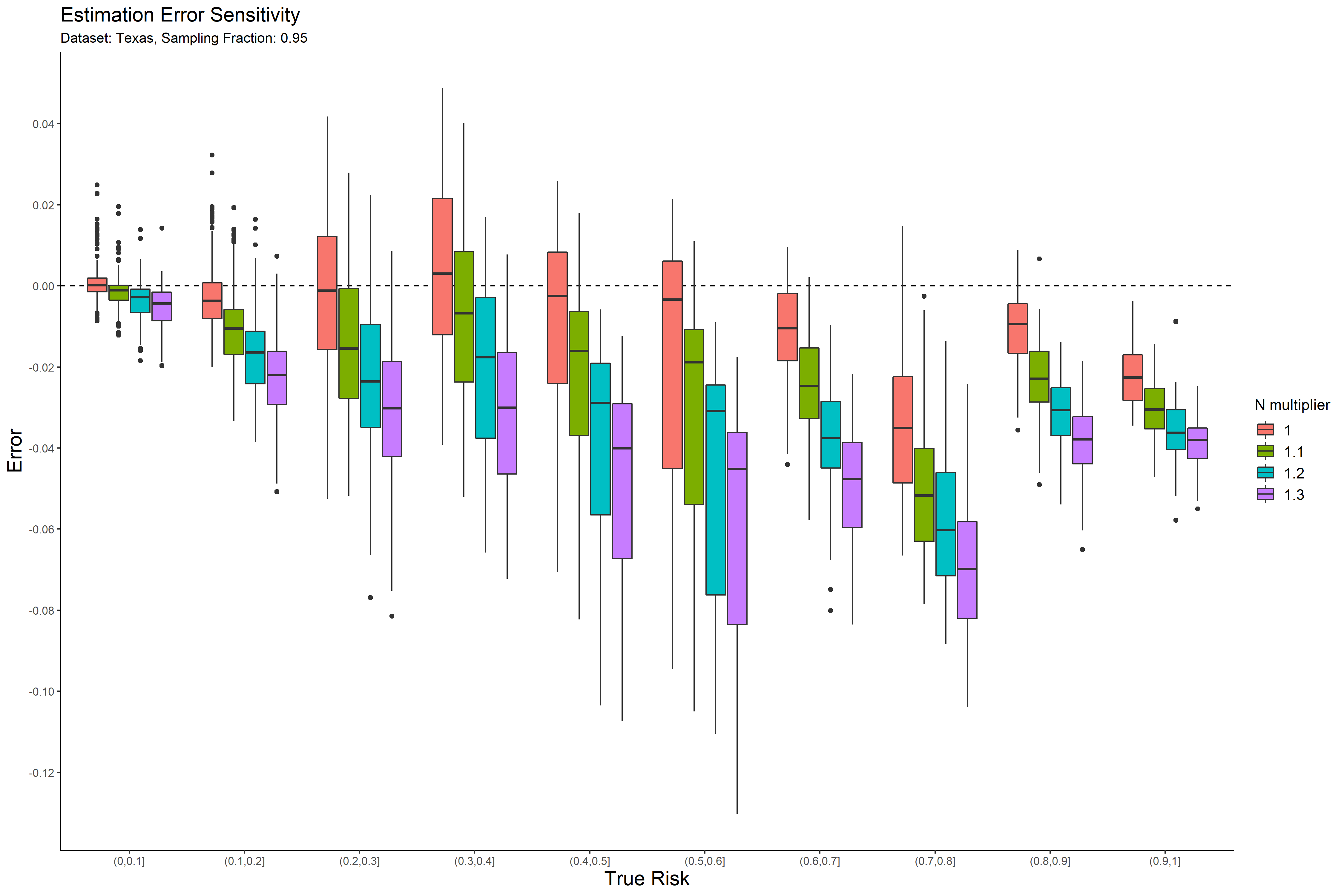

Supplement: S2 File — (ZIP) [file pone.0269097.s002.zip › tx/sensitivity.tx.19.png]

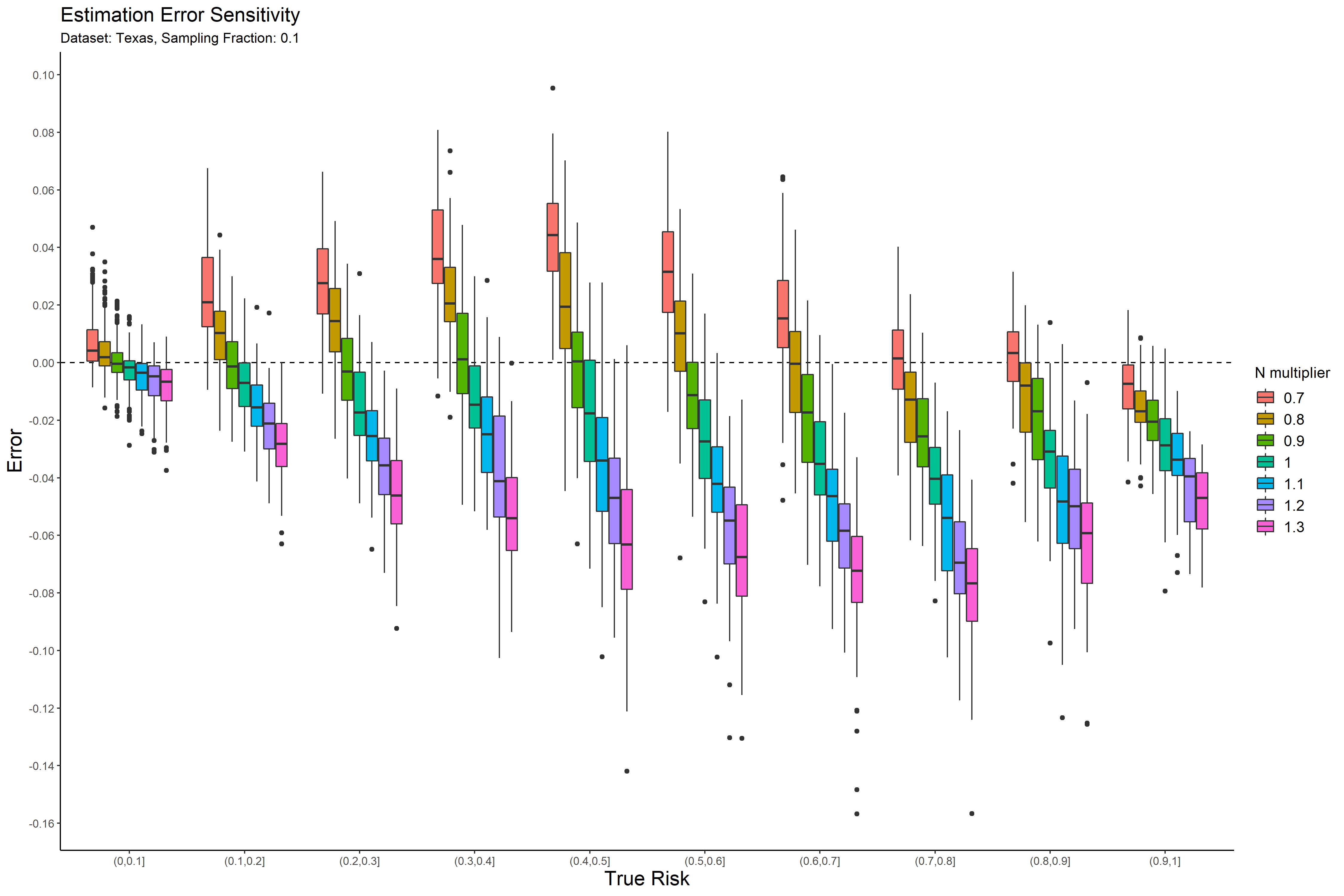

Supplement: S2 File — (ZIP) [file pone.0269097.s002.zip › tx/sensitivity.tx.2.png]

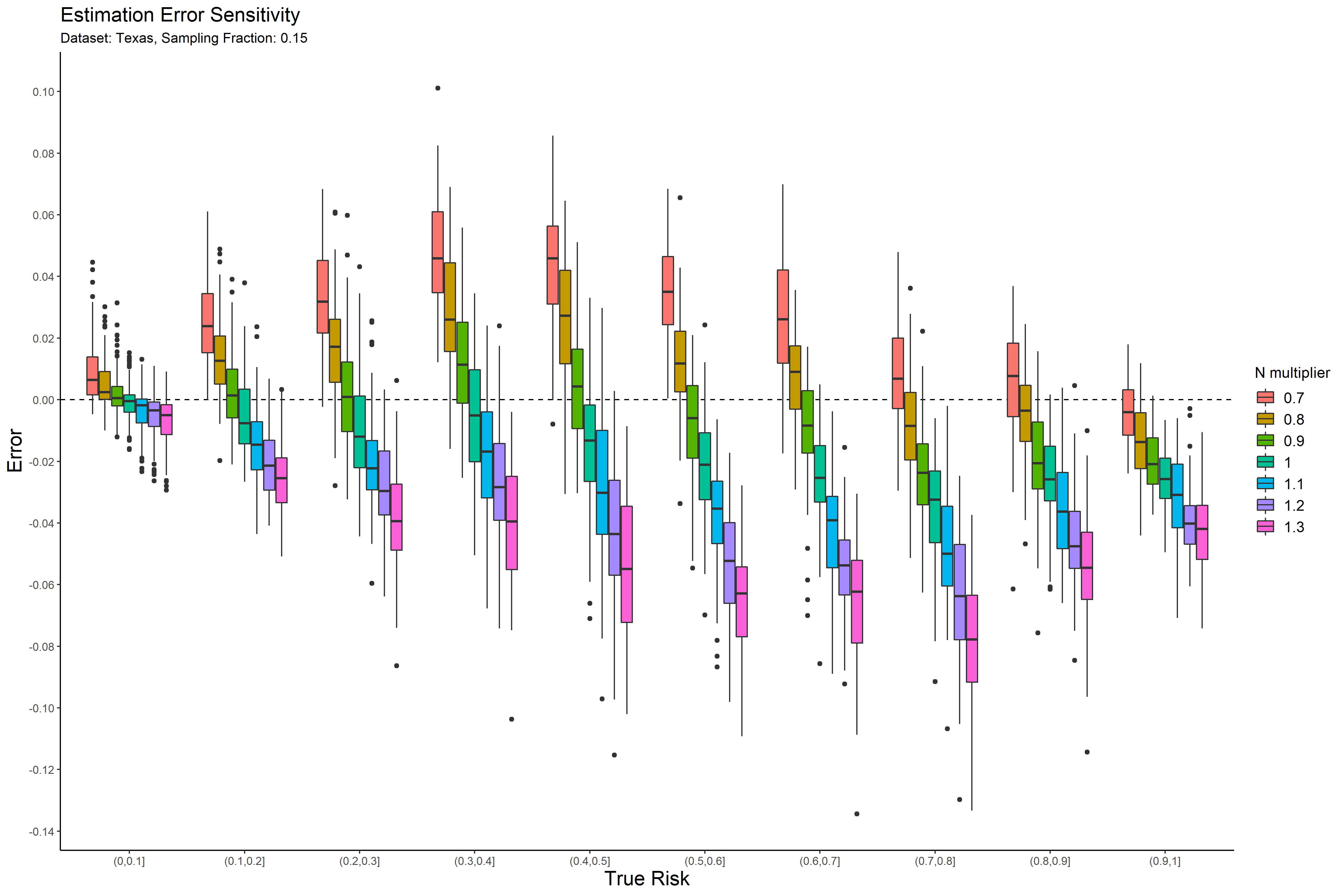

Supplement: S2 File — (ZIP) [file pone.0269097.s002.zip › tx/sensitivity.tx.3.png]

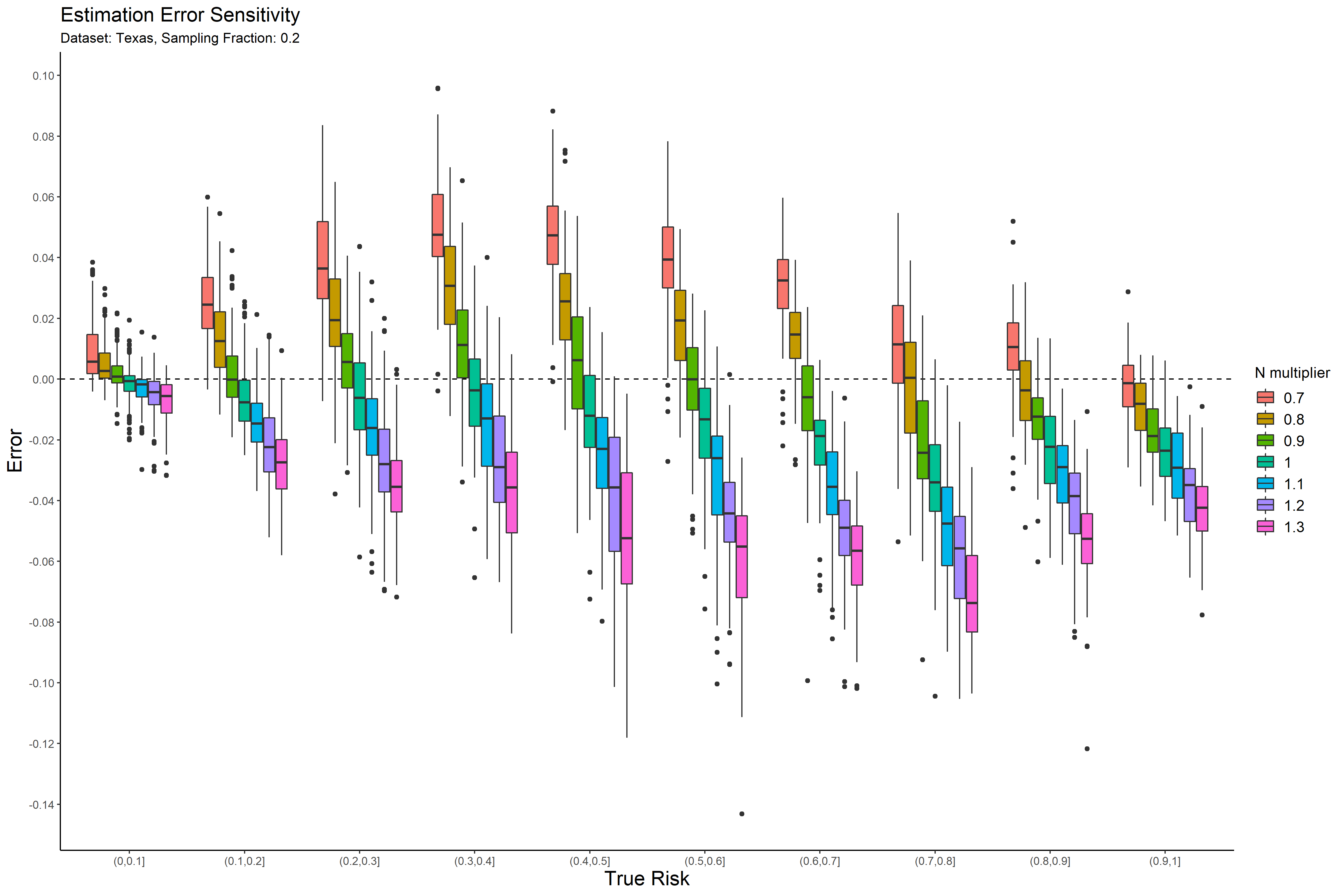

Supplement: S2 File — (ZIP) [file pone.0269097.s002.zip › tx/sensitivity.tx.4.png]

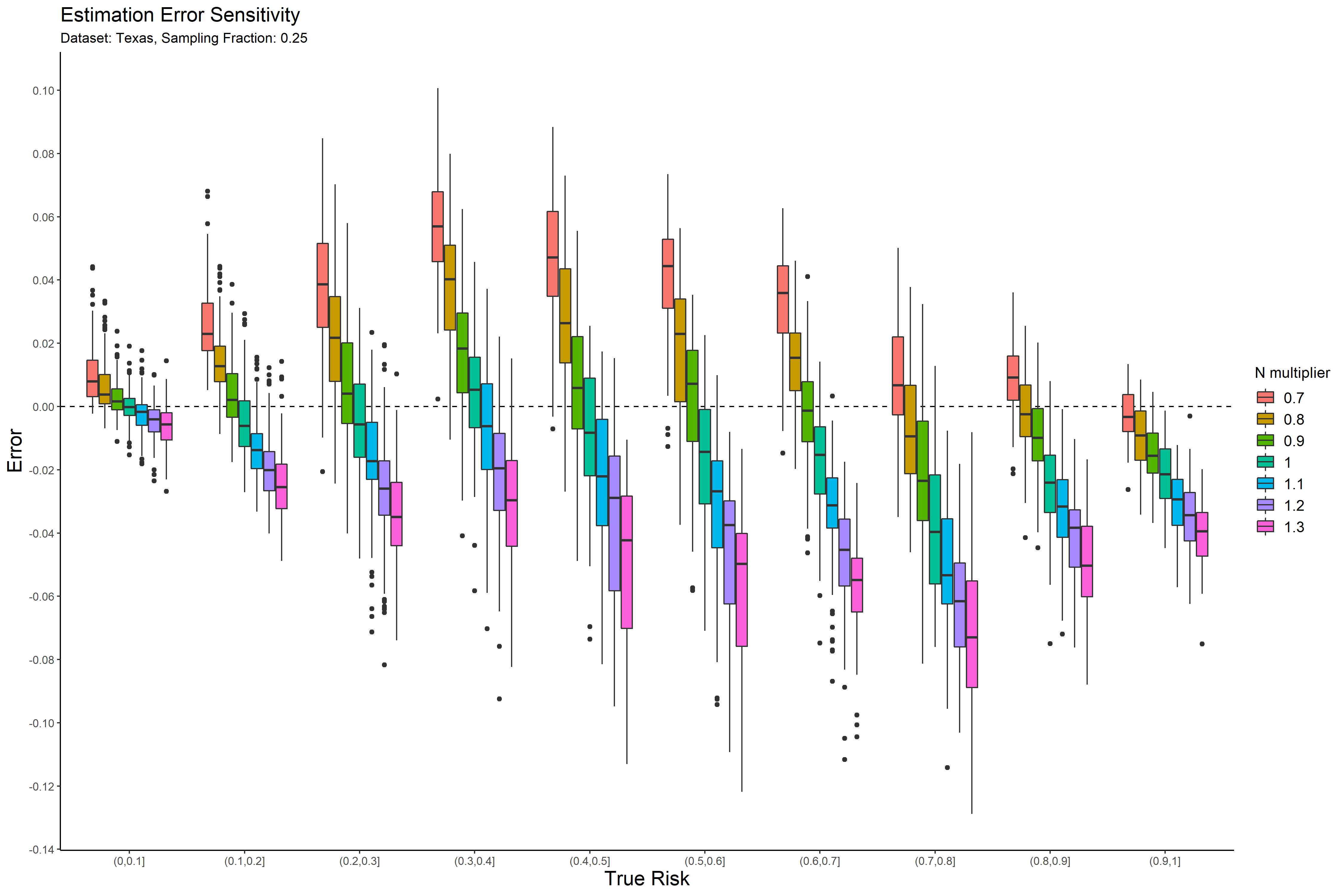

Supplement: S2 File — (ZIP) [file pone.0269097.s002.zip › tx/sensitivity.tx.5.png]

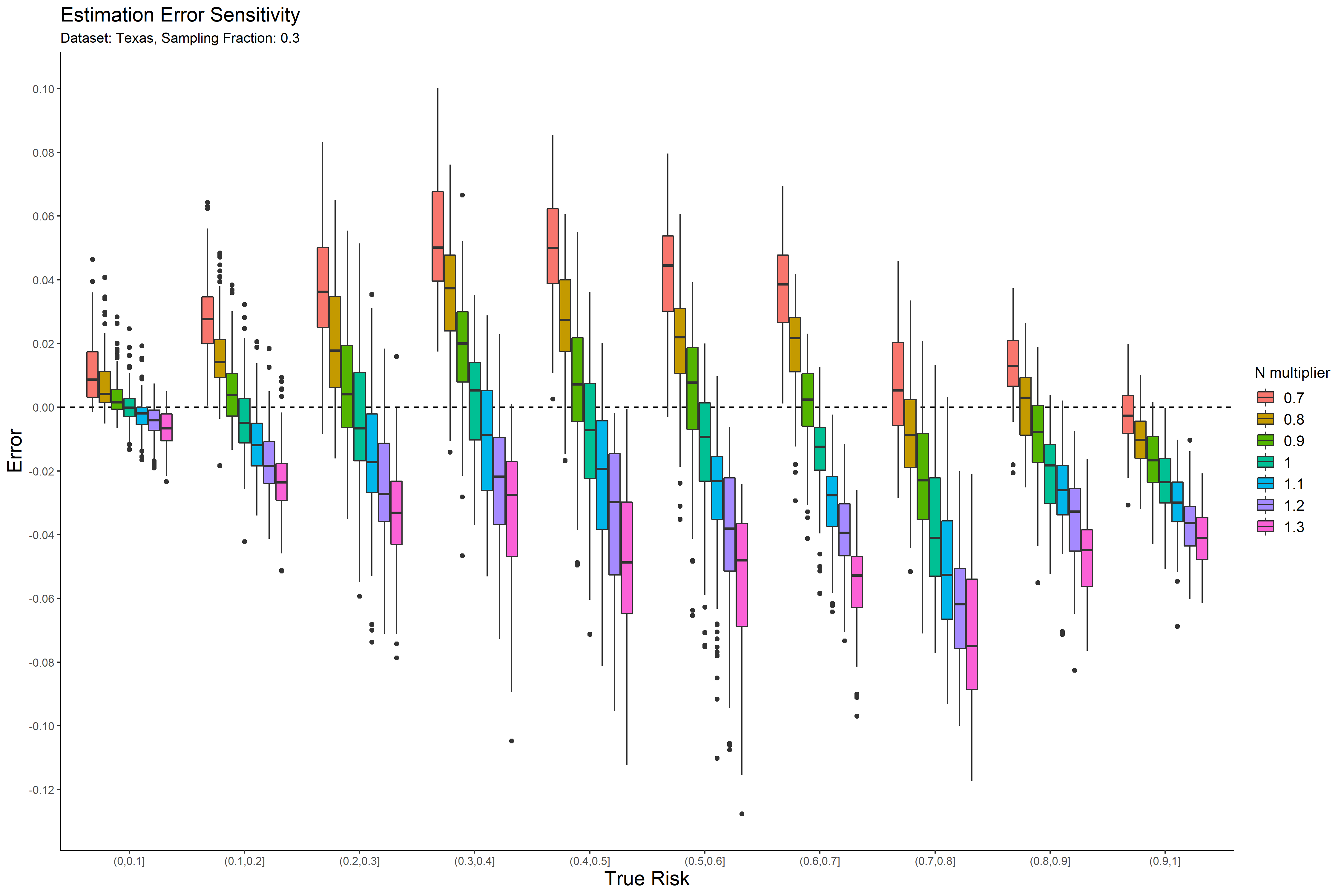

Supplement: S2 File — (ZIP) [file pone.0269097.s002.zip › tx/sensitivity.tx.6.png]

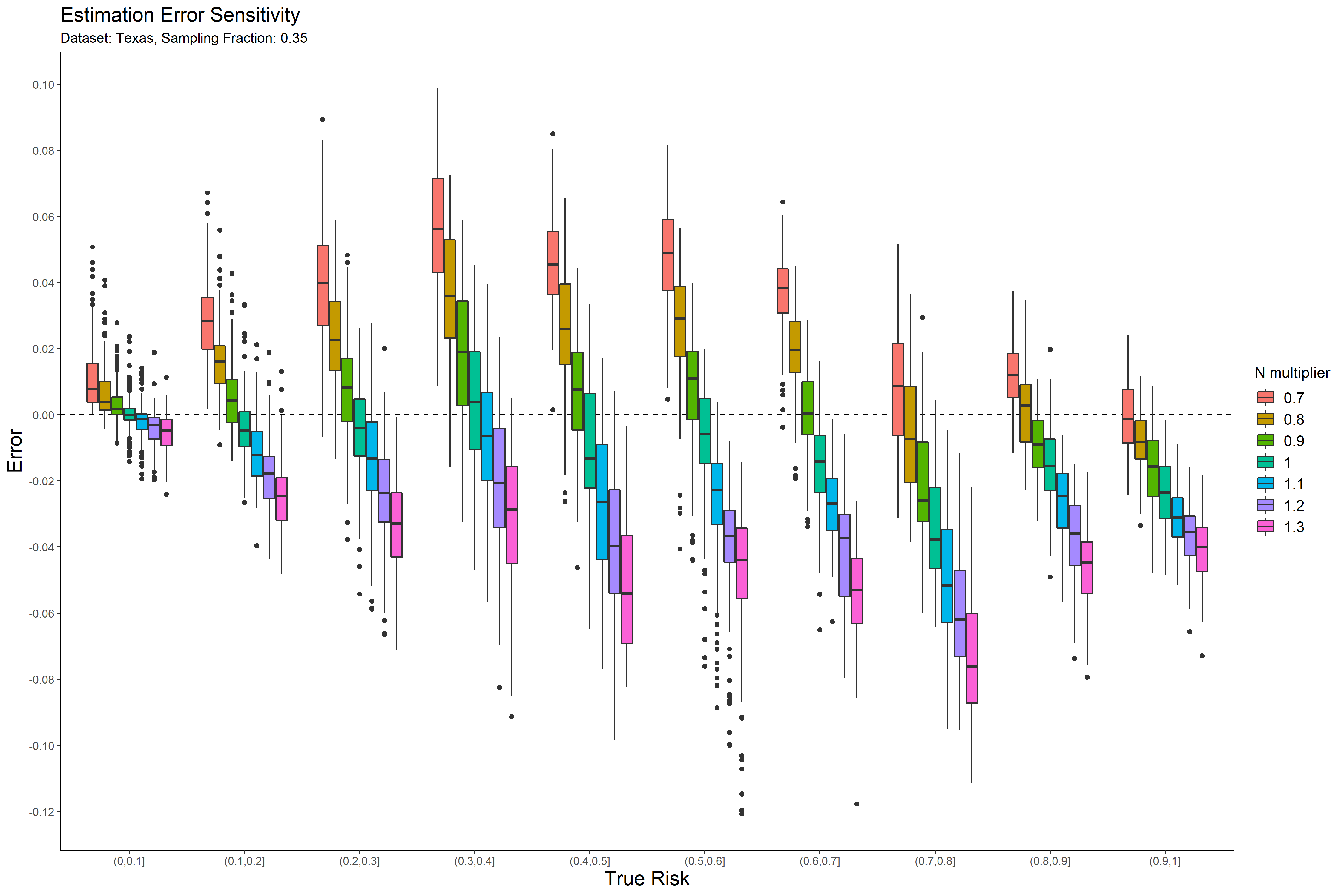

Supplement: S2 File — (ZIP) [file pone.0269097.s002.zip › tx/sensitivity.tx.7.png]

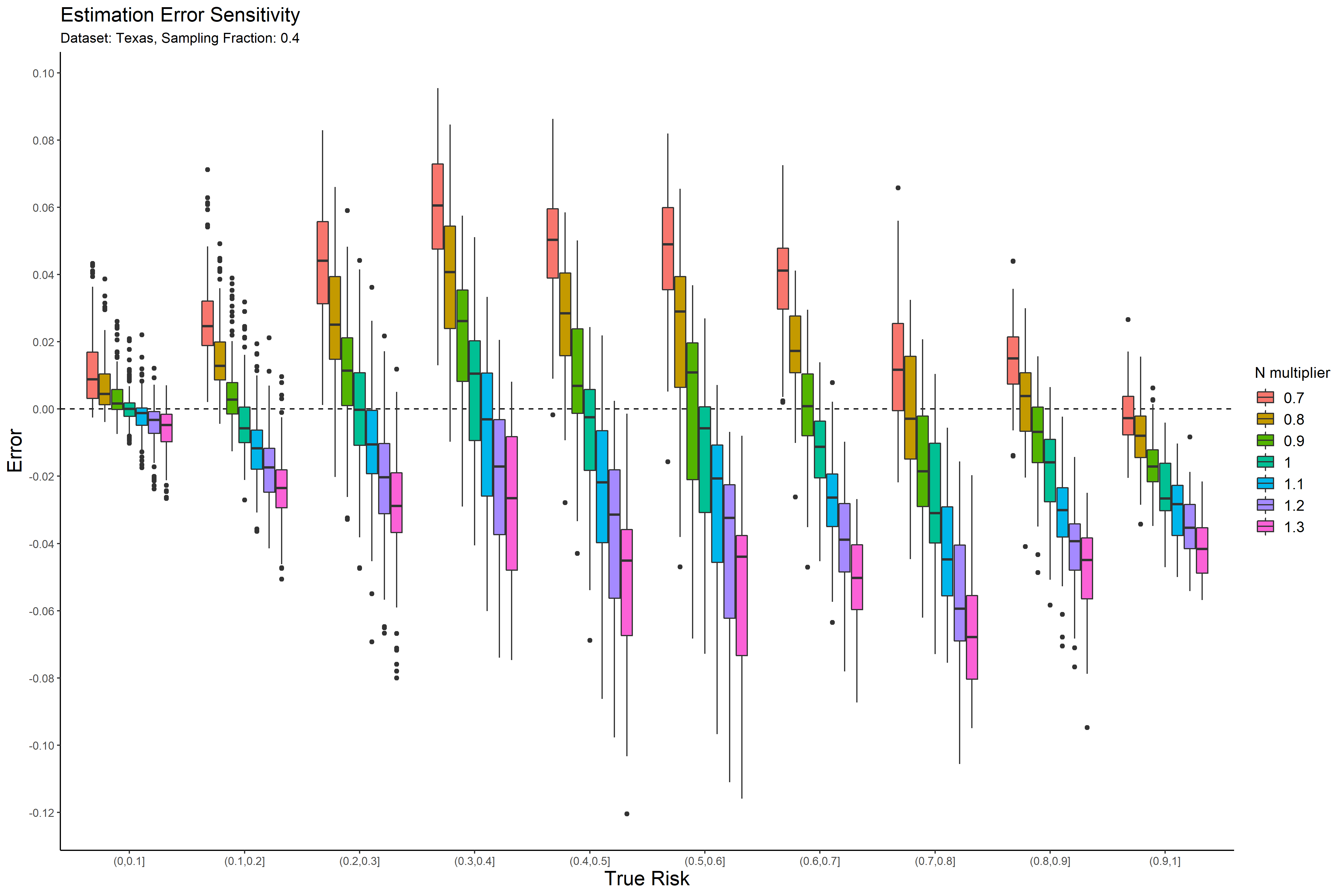

Supplement: S2 File — (ZIP) [file pone.0269097.s002.zip › tx/sensitivity.tx.8.png]

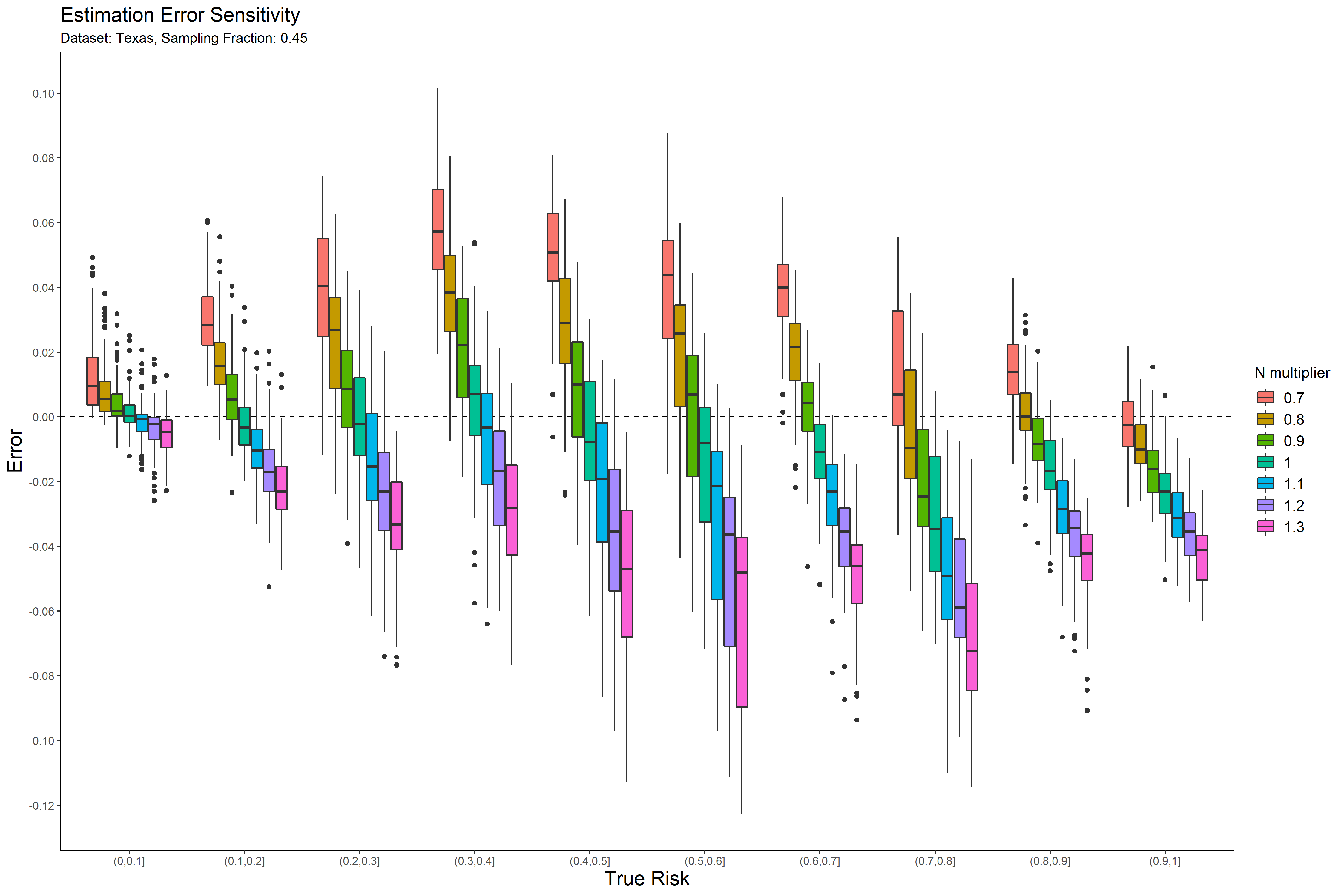

Supplement: S2 File — (ZIP) [file pone.0269097.s002.zip › tx/sensitivity.tx.9.png]

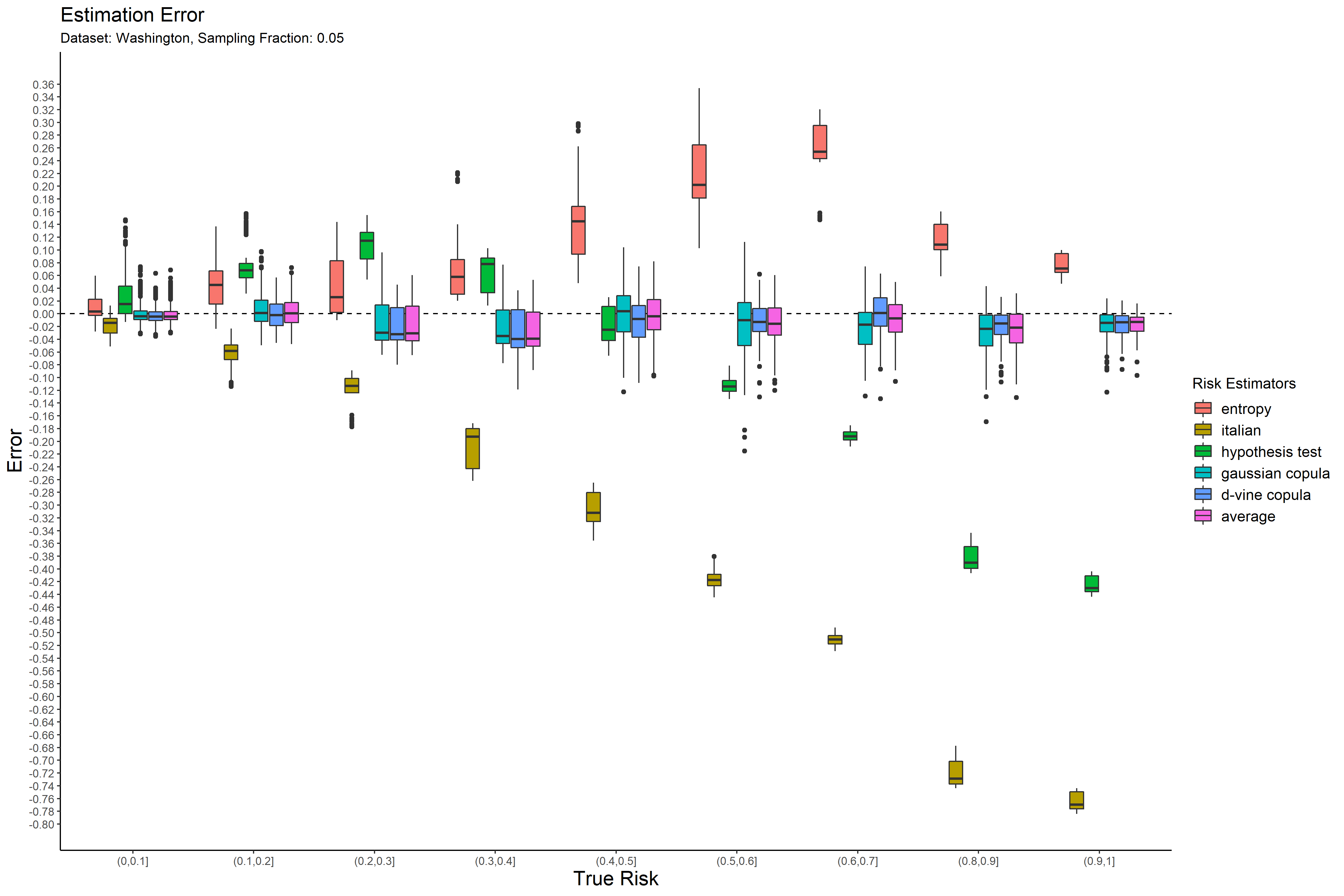

Supplement: S2 File — (ZIP) [file pone.0269097.s002.zip › wa/comparison.wa.1.png]

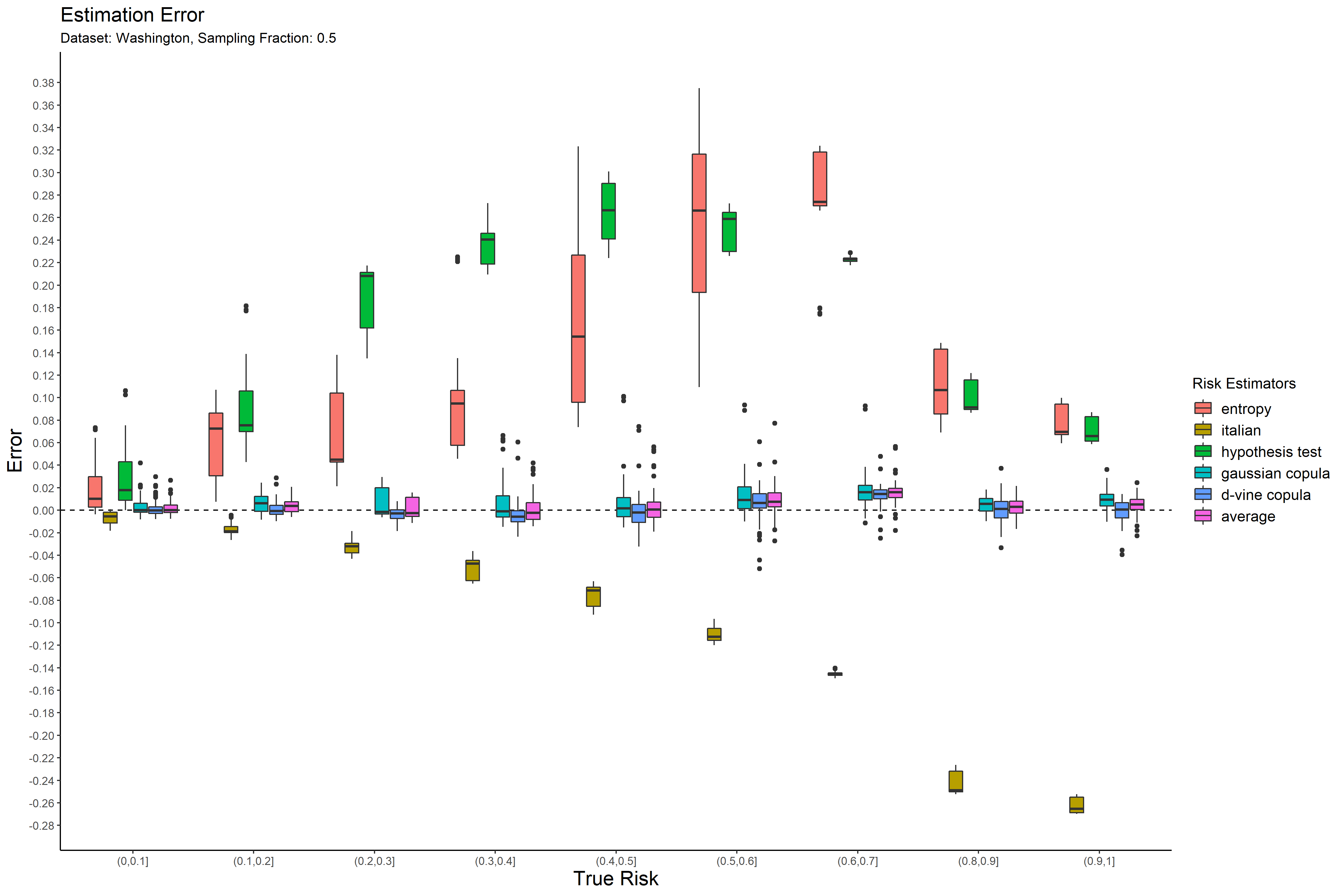

Supplement: S2 File — (ZIP) [file pone.0269097.s002.zip › wa/comparison.wa.10.png]

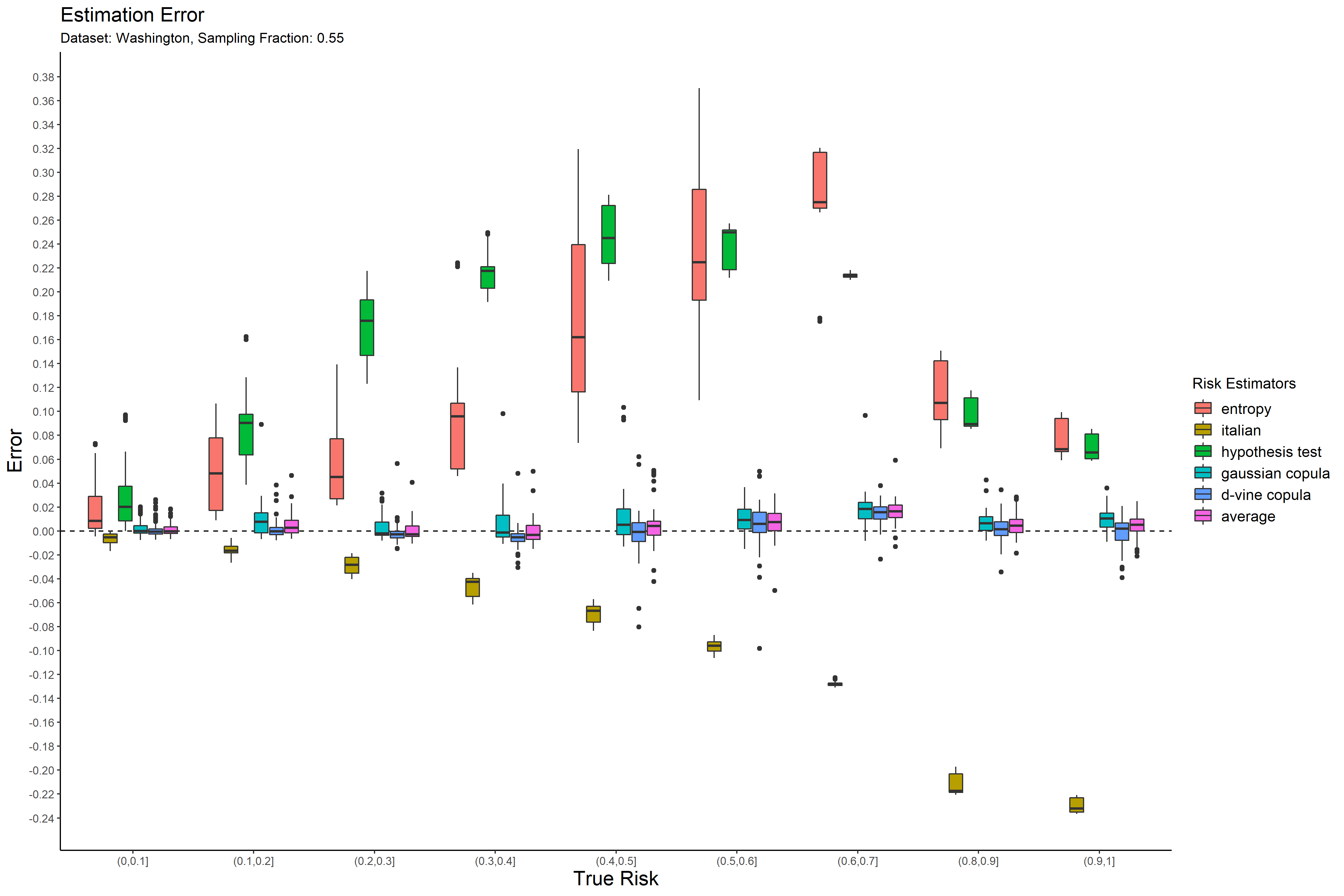

Supplement: S2 File — (ZIP) [file pone.0269097.s002.zip › wa/comparison.wa.11.png]

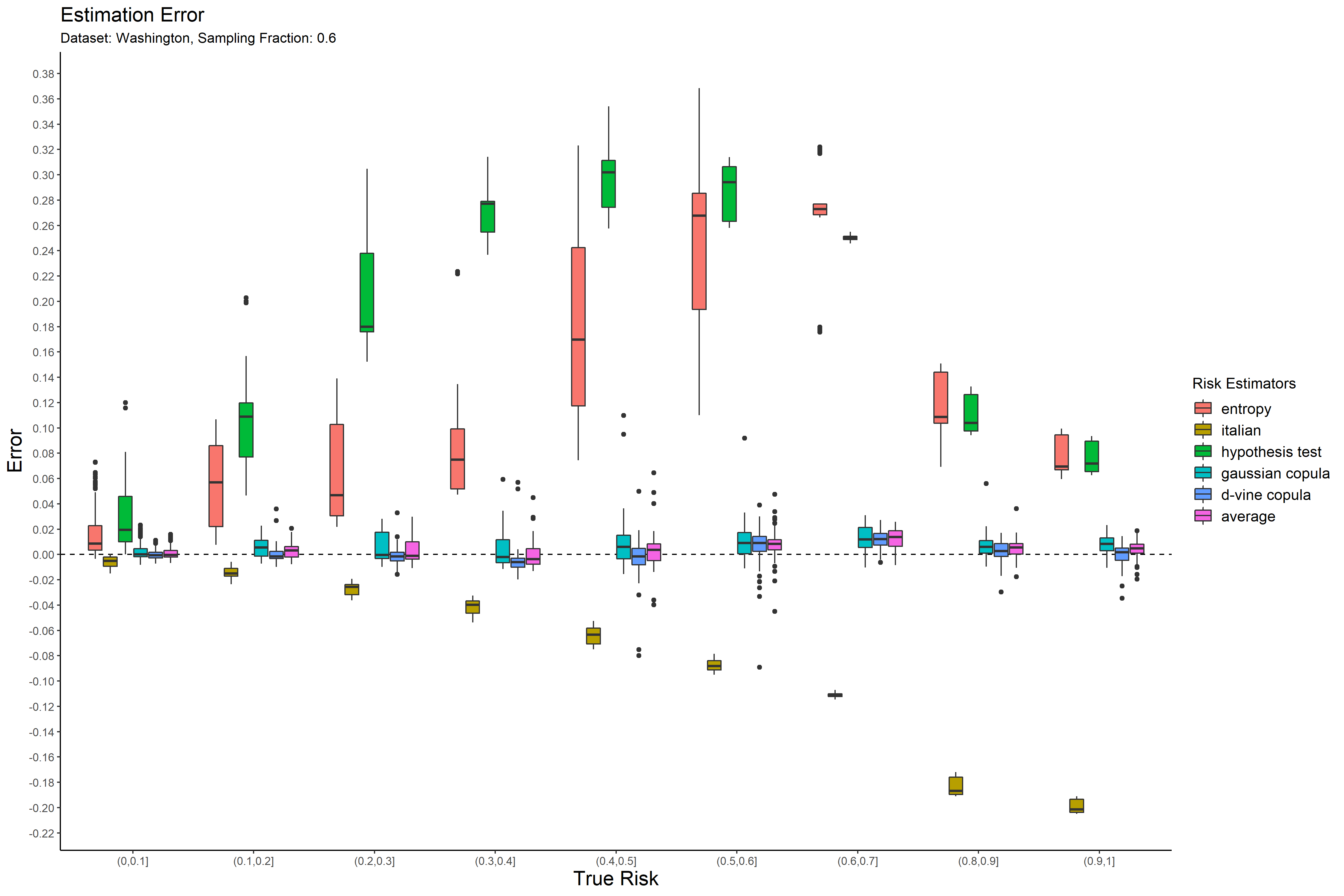

Supplement: S2 File — (ZIP) [file pone.0269097.s002.zip › wa/comparison.wa.12.png]

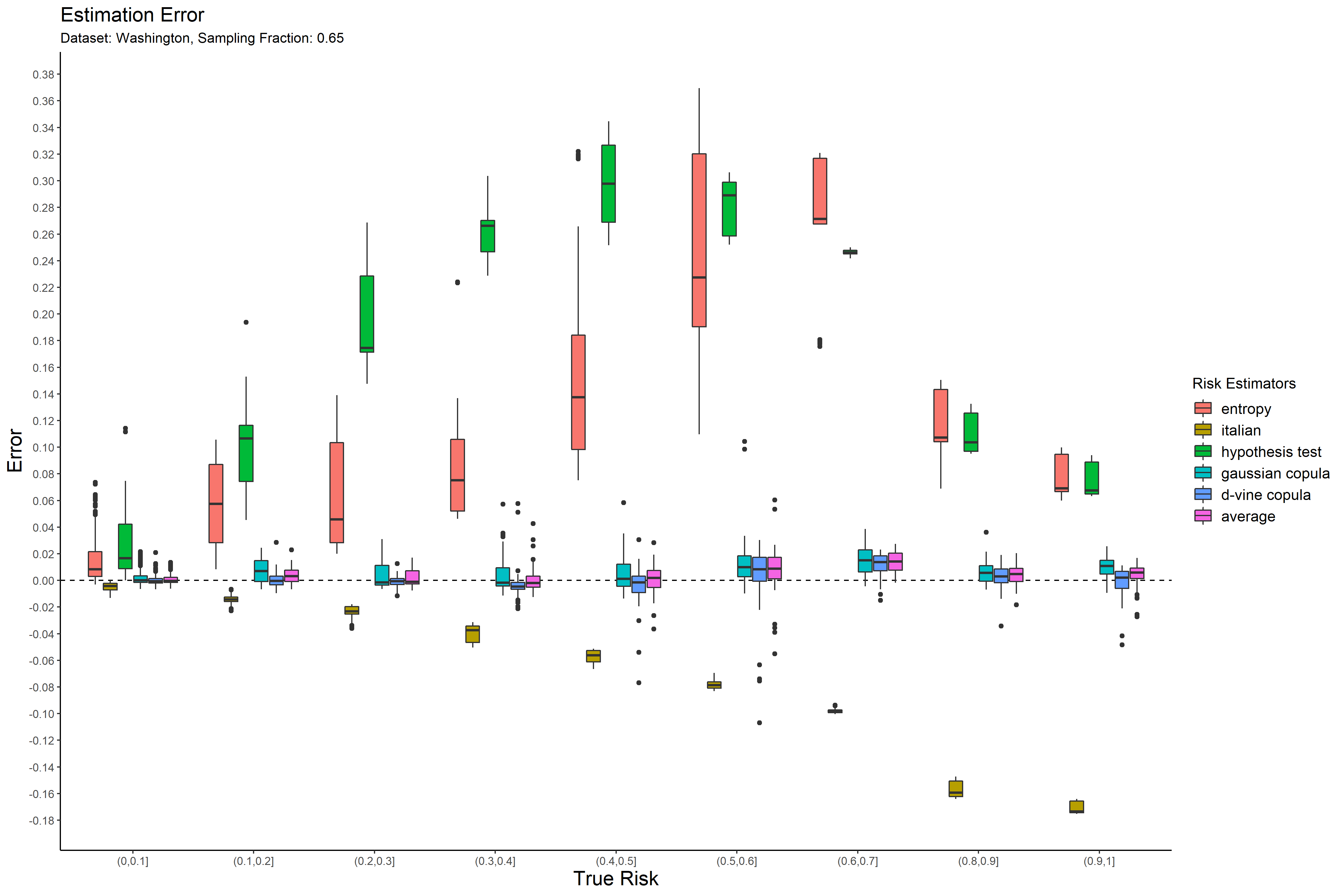

Supplement: S2 File — (ZIP) [file pone.0269097.s002.zip › wa/comparison.wa.13.png]

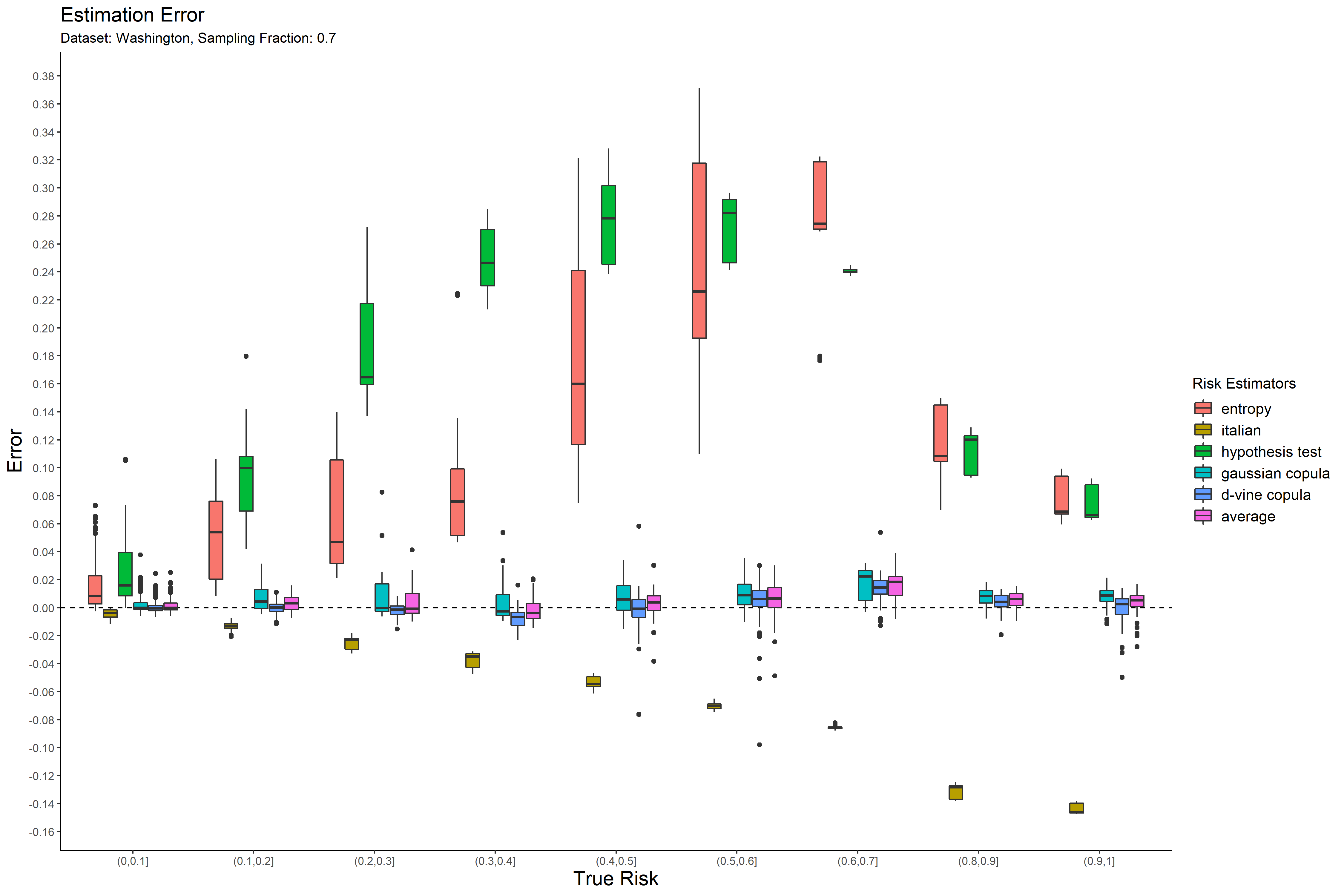

Supplement: S2 File — (ZIP) [file pone.0269097.s002.zip › wa/comparison.wa.14.png]

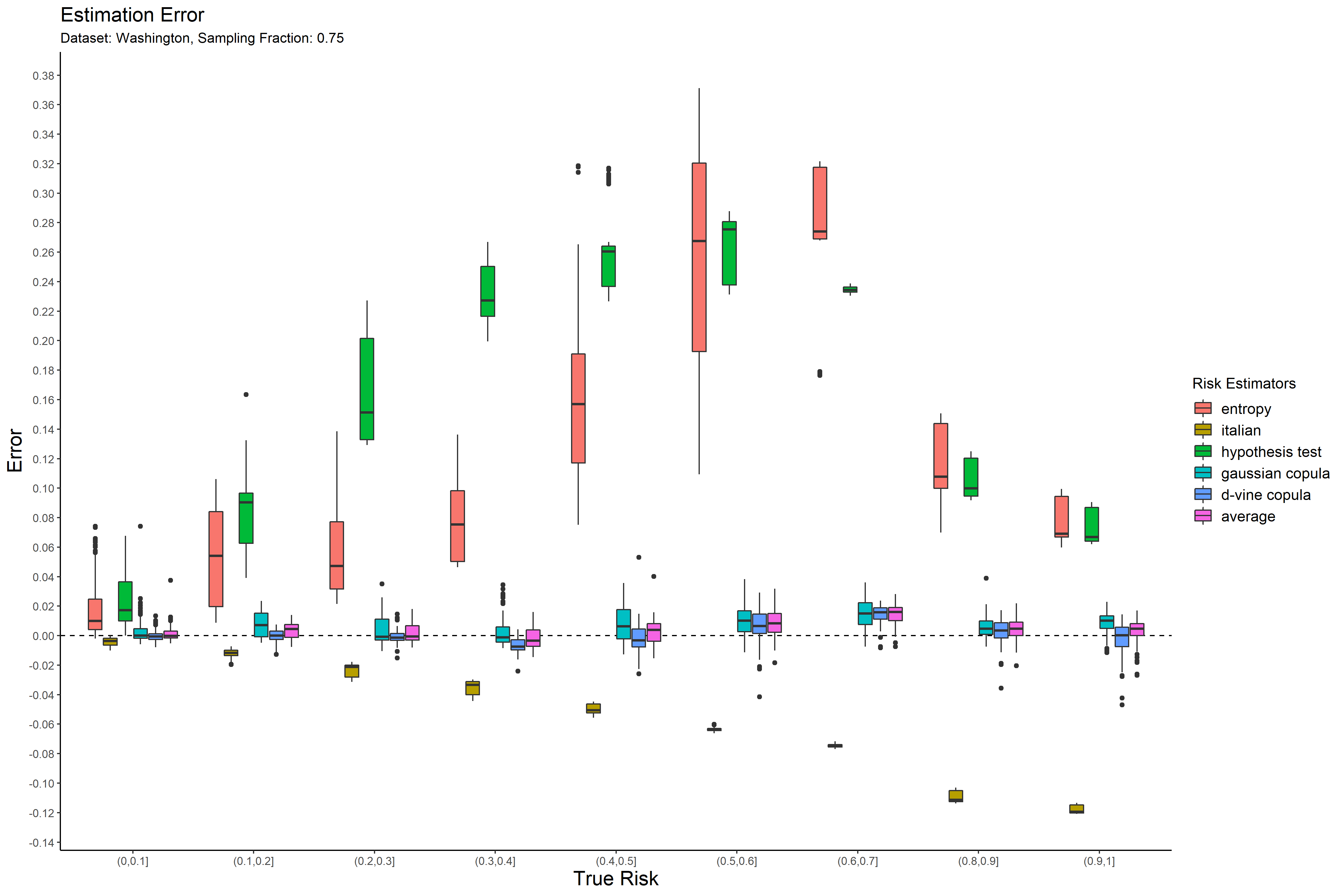

Supplement: S2 File — (ZIP) [file pone.0269097.s002.zip › wa/comparison.wa.15.png]

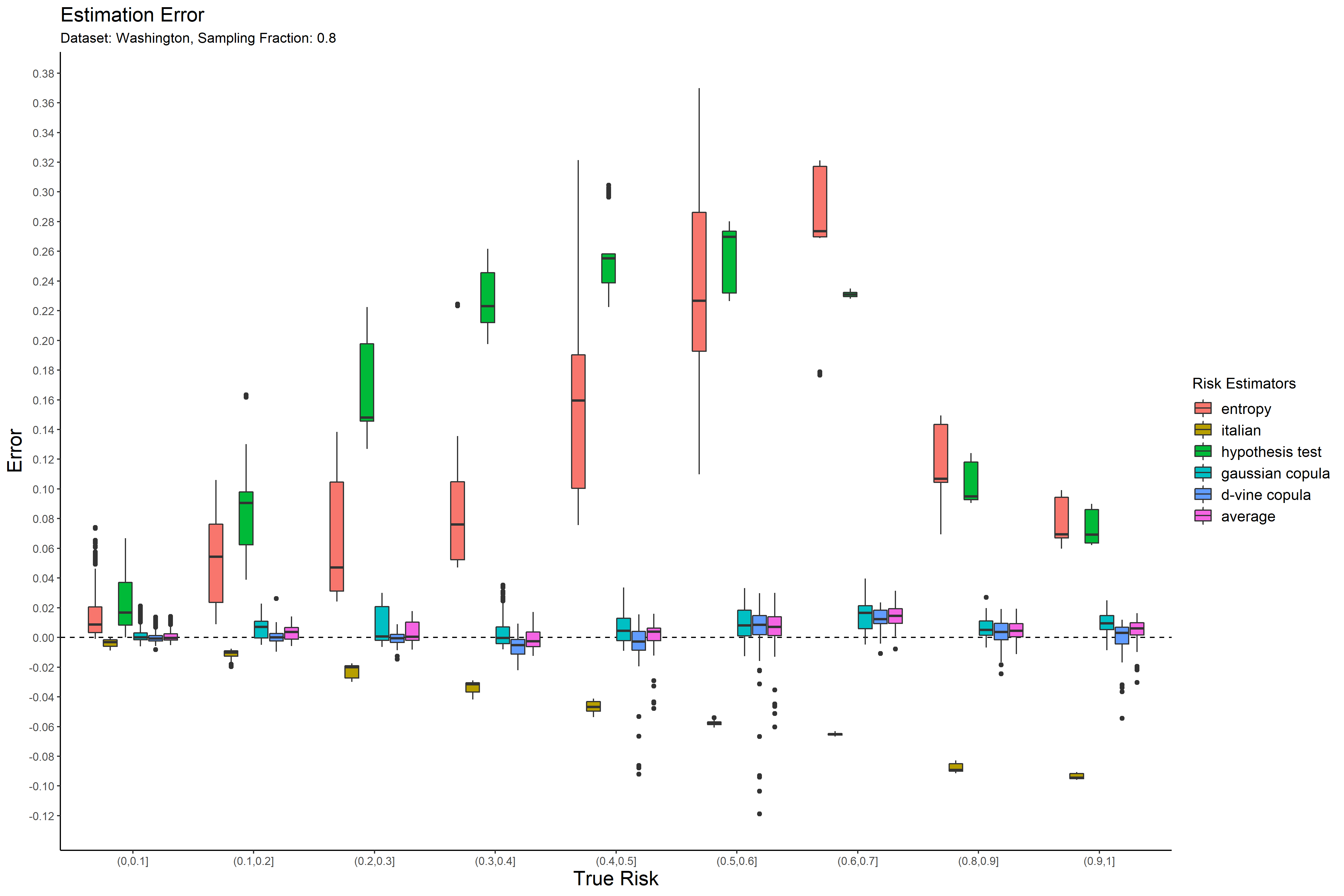

Supplement: S2 File — (ZIP) [file pone.0269097.s002.zip › wa/comparison.wa.16.png]

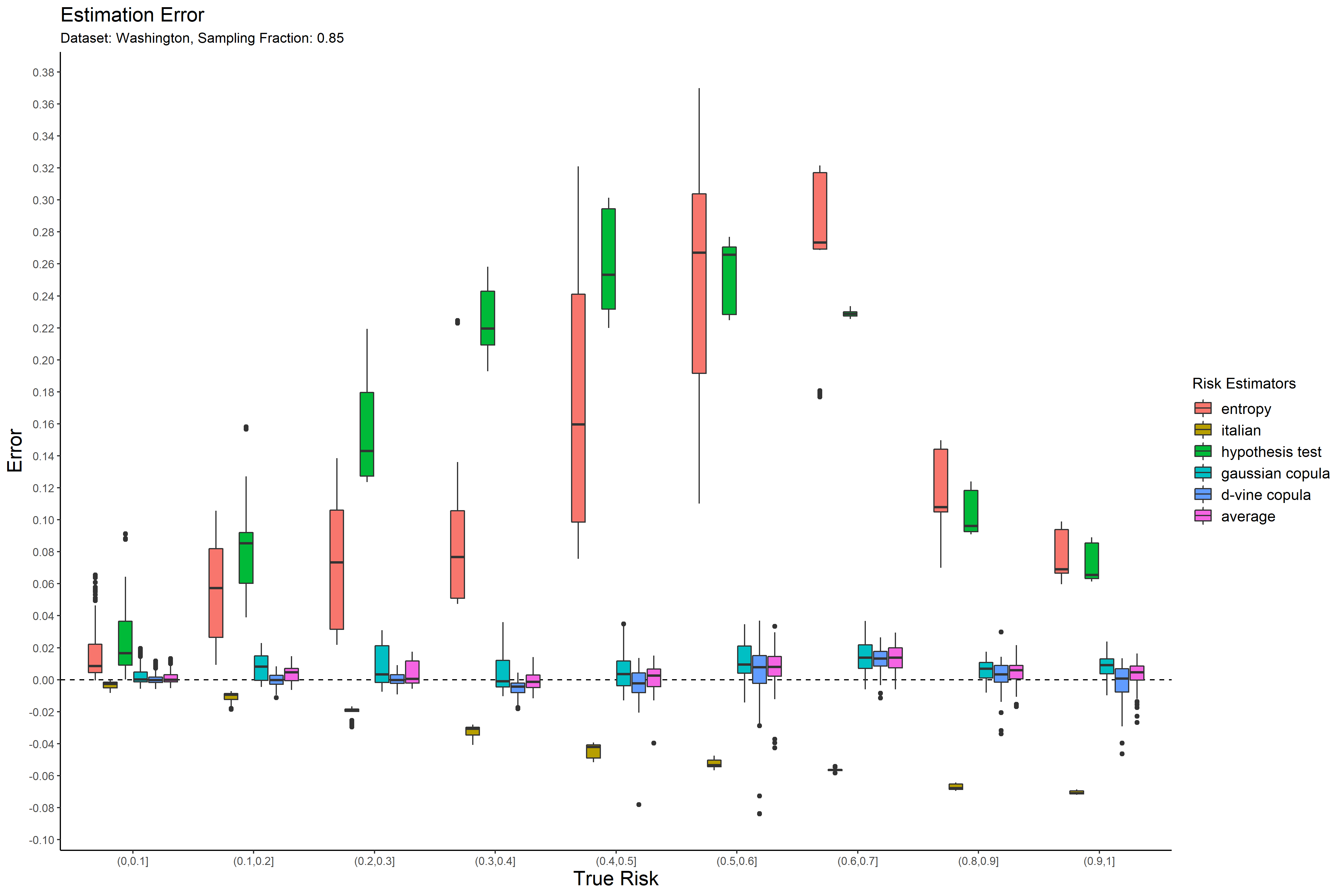

Supplement: S2 File — (ZIP) [file pone.0269097.s002.zip › wa/comparison.wa.17.png]

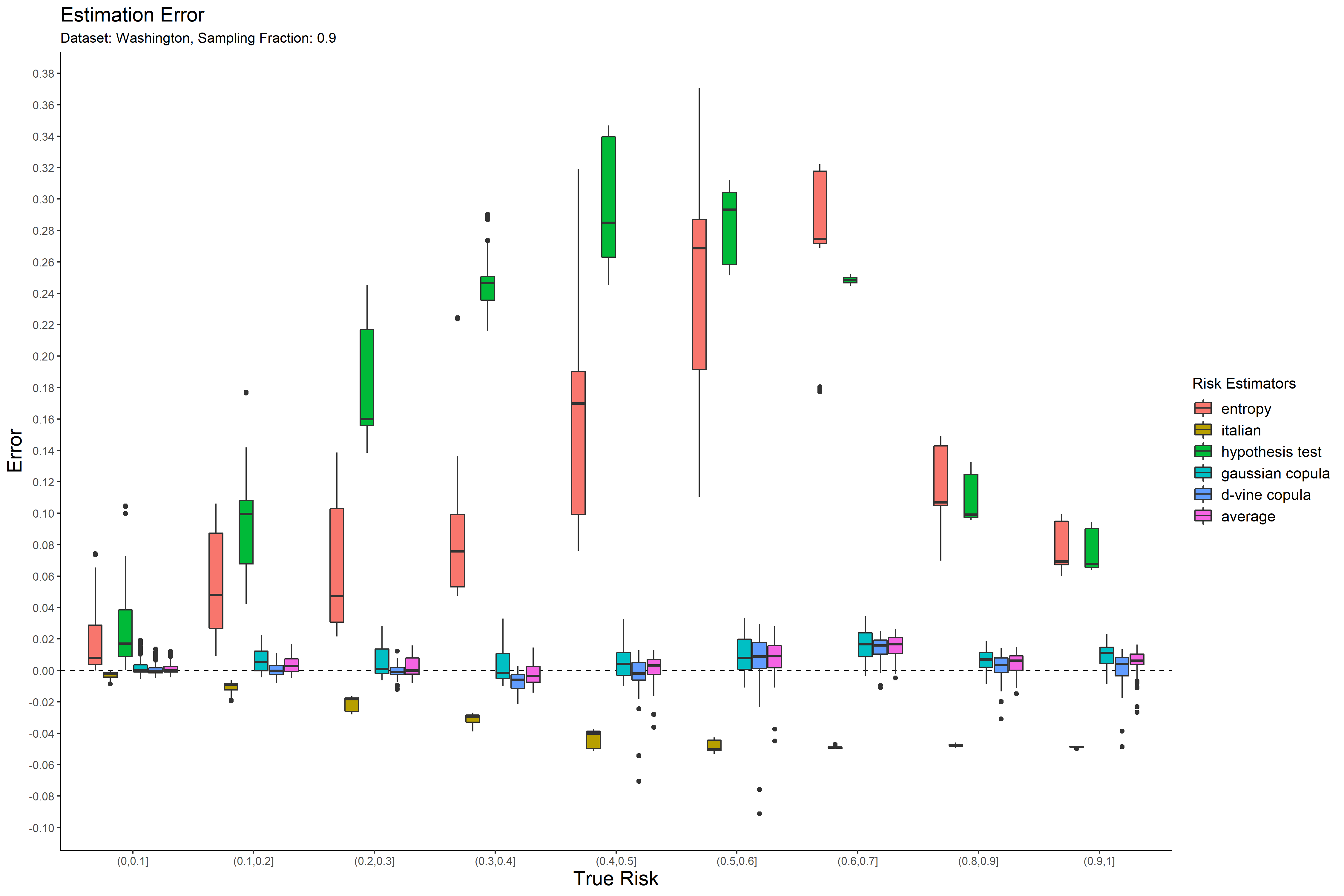

Supplement: S2 File — (ZIP) [file pone.0269097.s002.zip › wa/comparison.wa.18.png]

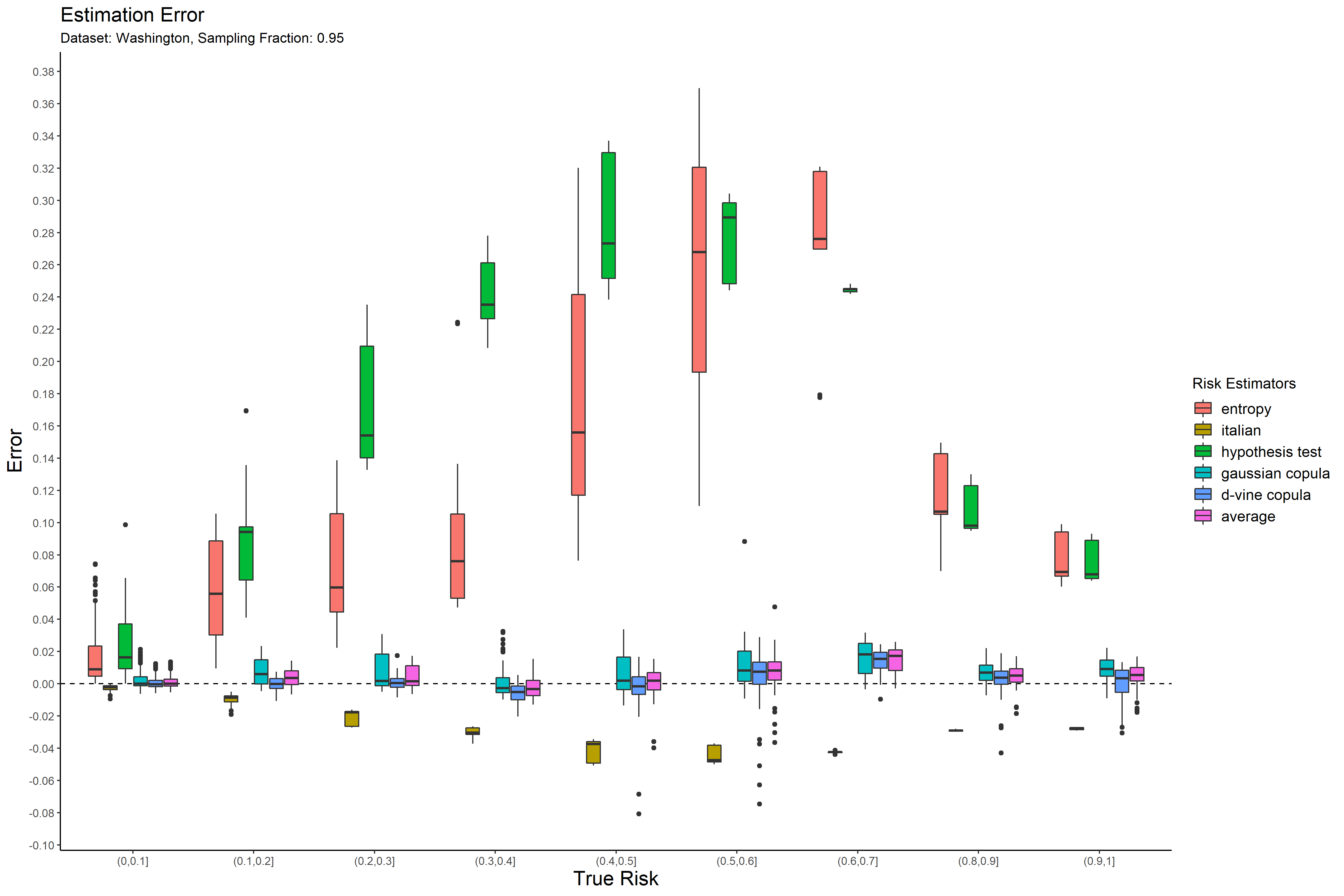

Supplement: S2 File — (ZIP) [file pone.0269097.s002.zip › wa/comparison.wa.19.png]

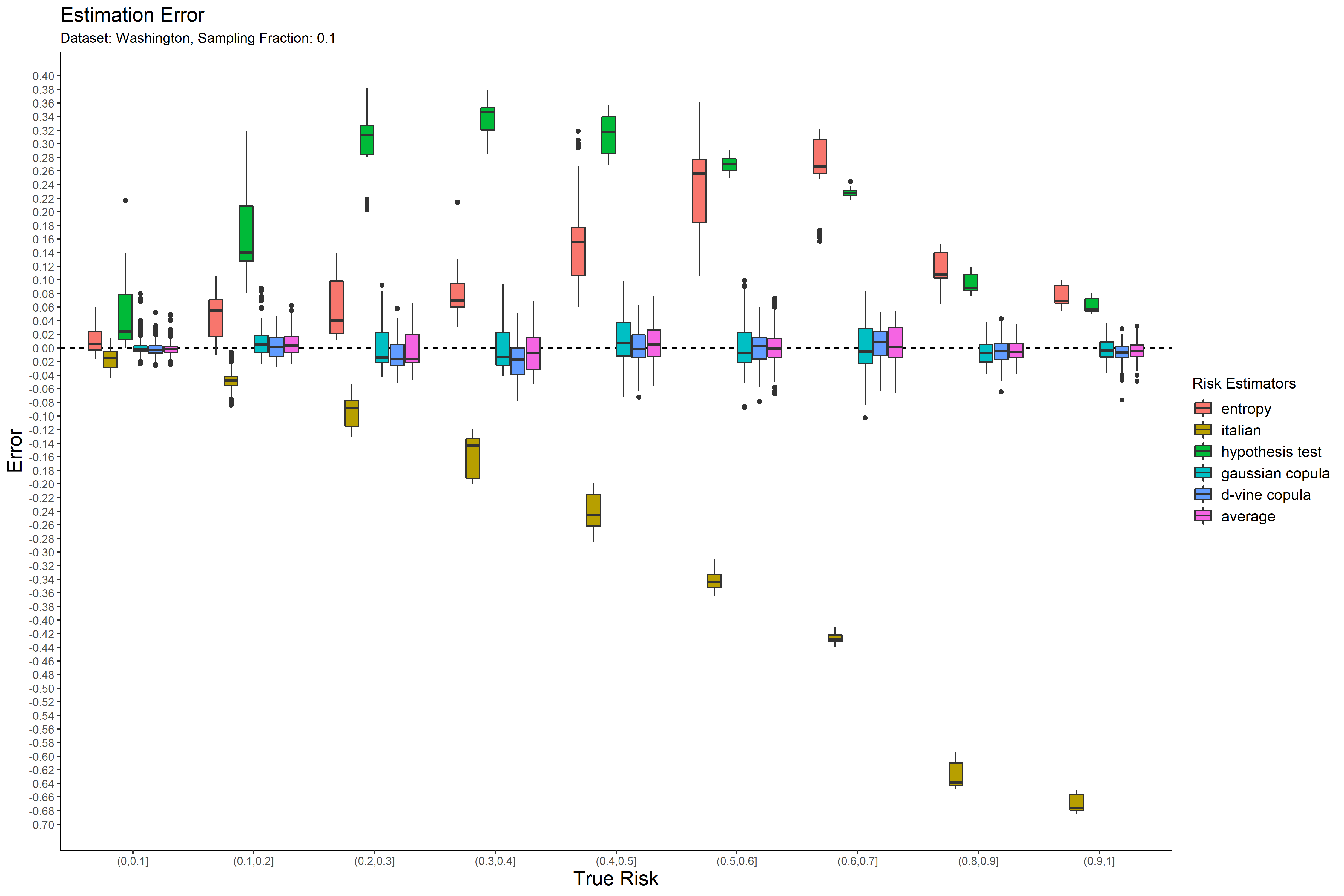

Supplement: S2 File — (ZIP) [file pone.0269097.s002.zip › wa/comparison.wa.2.png]

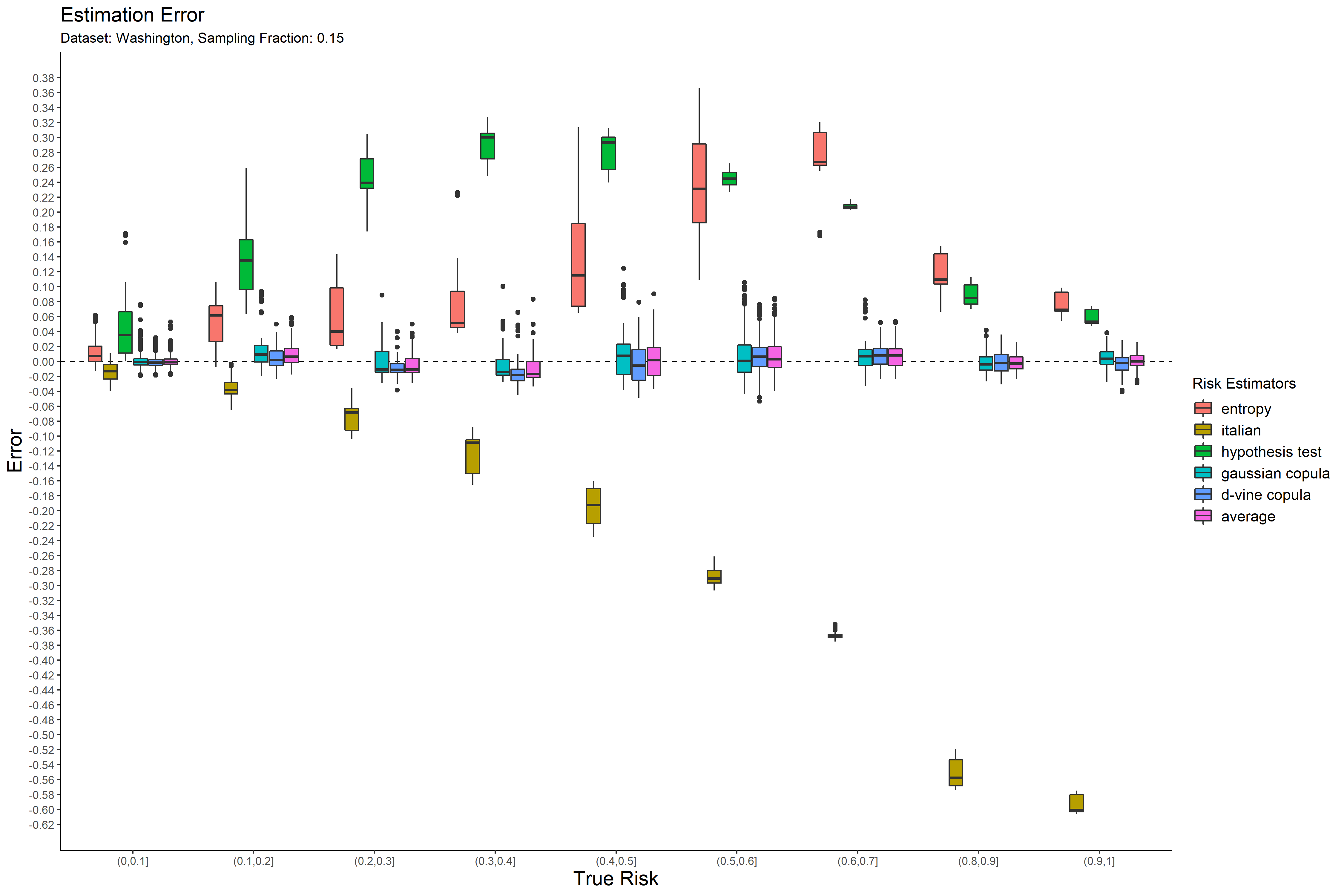

Supplement: S2 File — (ZIP) [file pone.0269097.s002.zip › wa/comparison.wa.3.png]

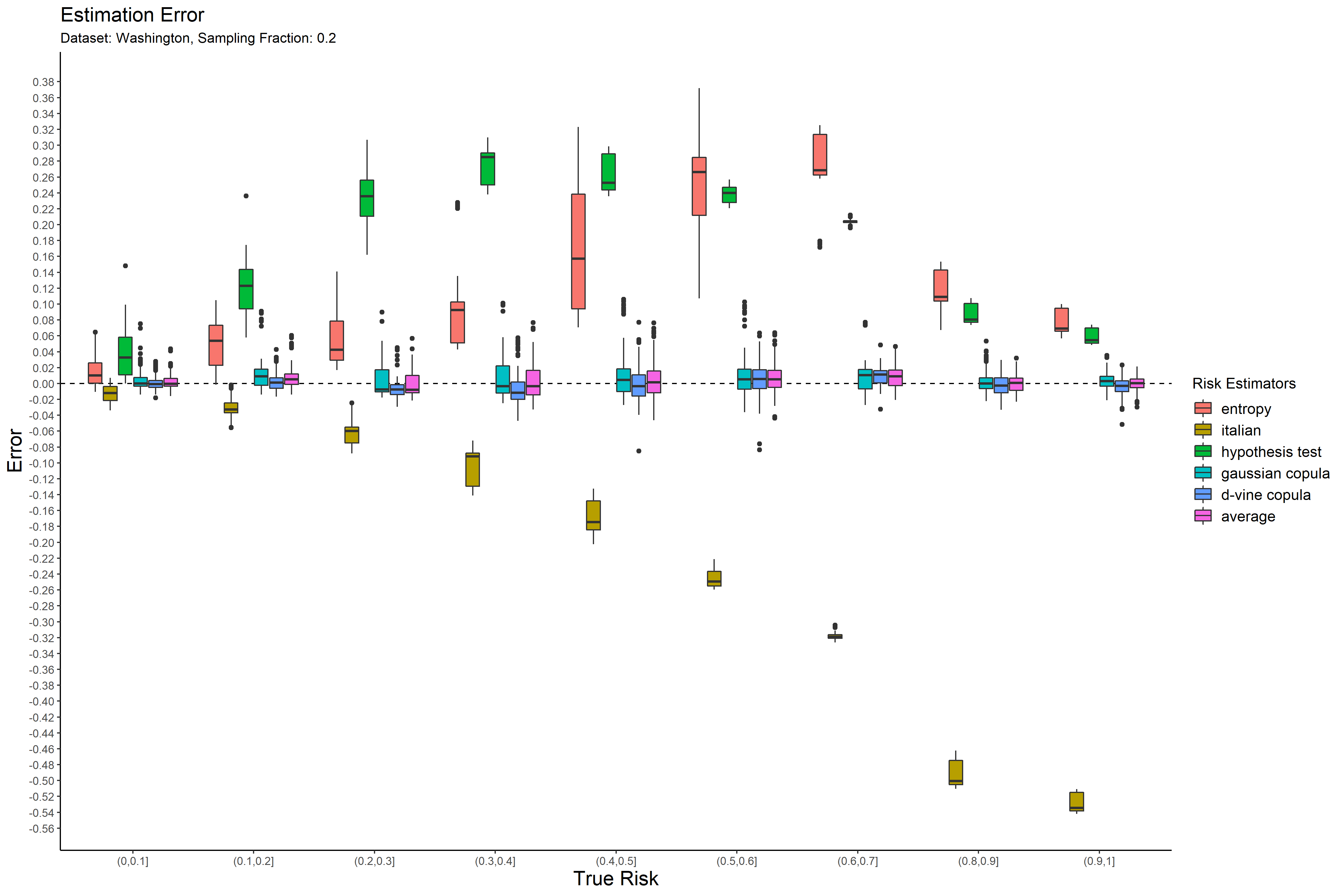

Supplement: S2 File — (ZIP) [file pone.0269097.s002.zip › wa/comparison.wa.4.png]

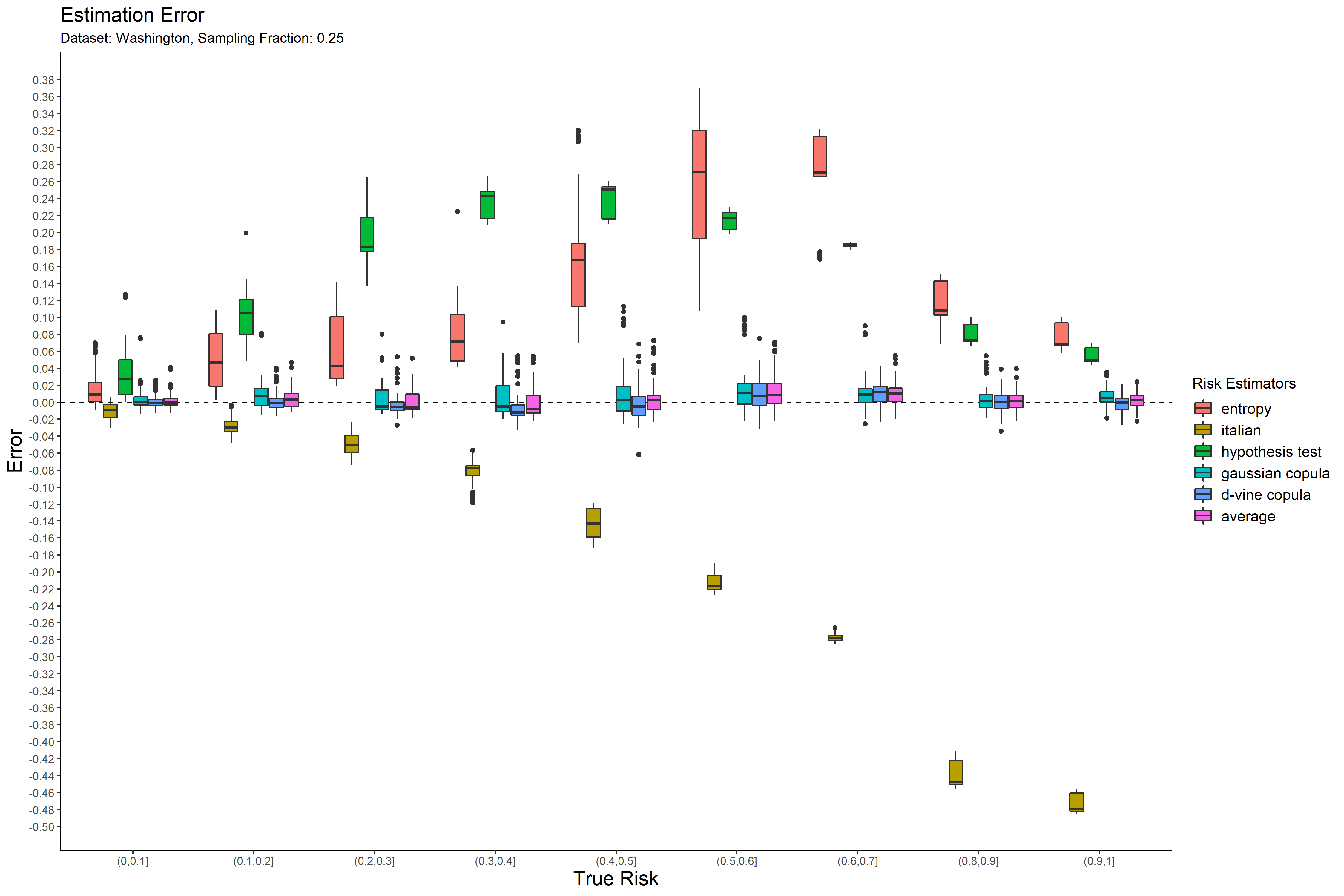

Supplement: S2 File — (ZIP) [file pone.0269097.s002.zip › wa/comparison.wa.5.png]

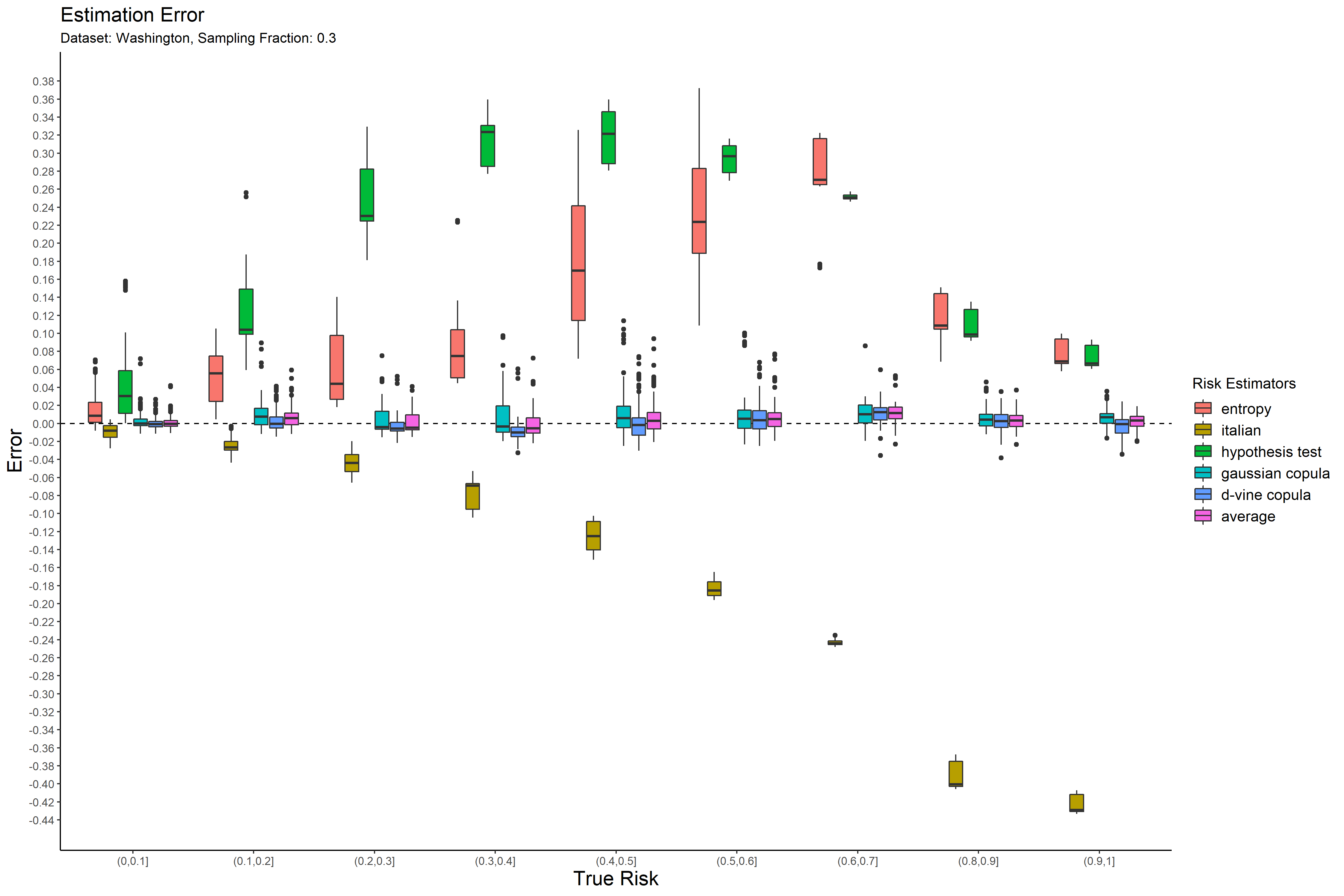

Supplement: S2 File — (ZIP) [file pone.0269097.s002.zip › wa/comparison.wa.6.png]

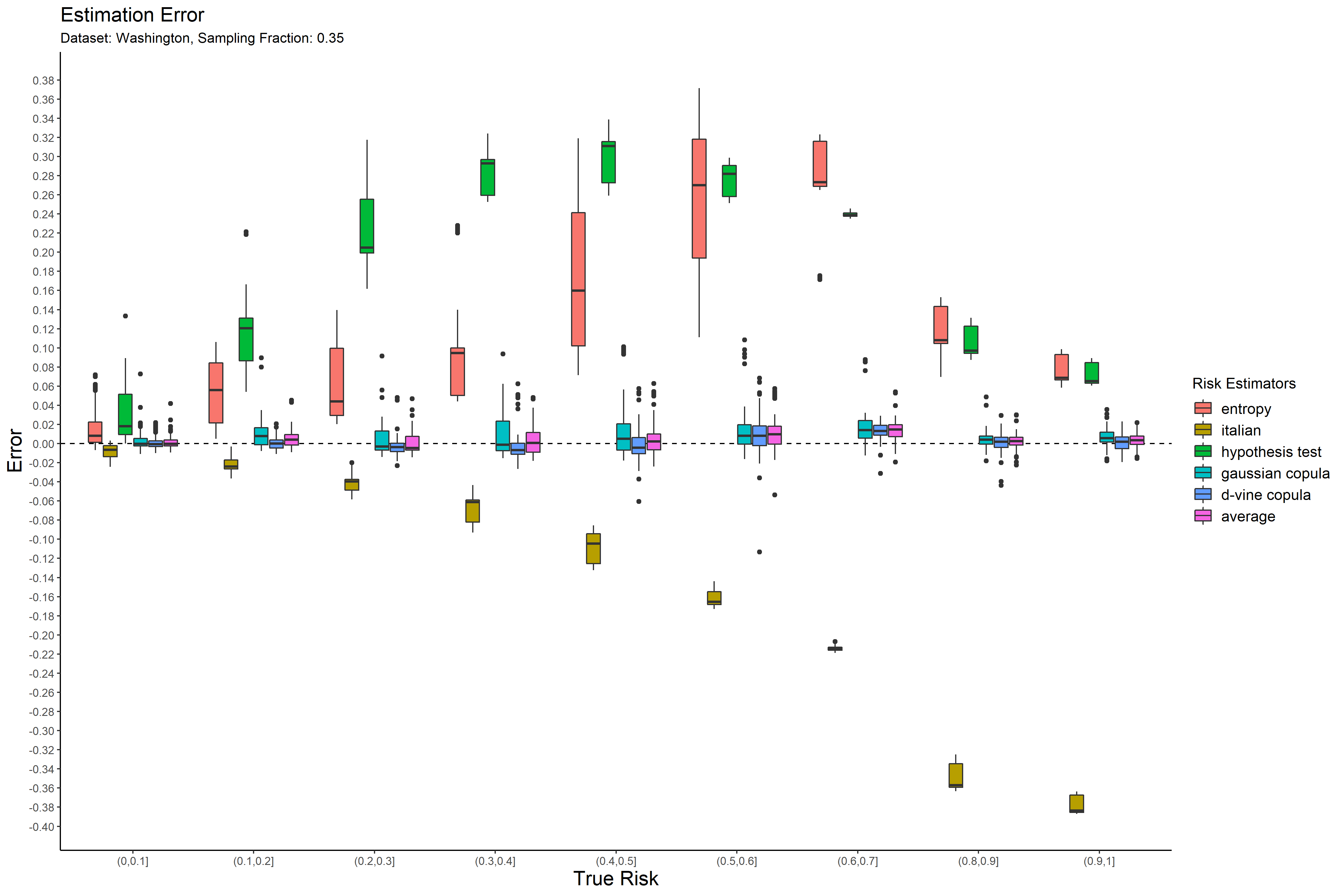

Supplement: S2 File — (ZIP) [file pone.0269097.s002.zip › wa/comparison.wa.7.png]

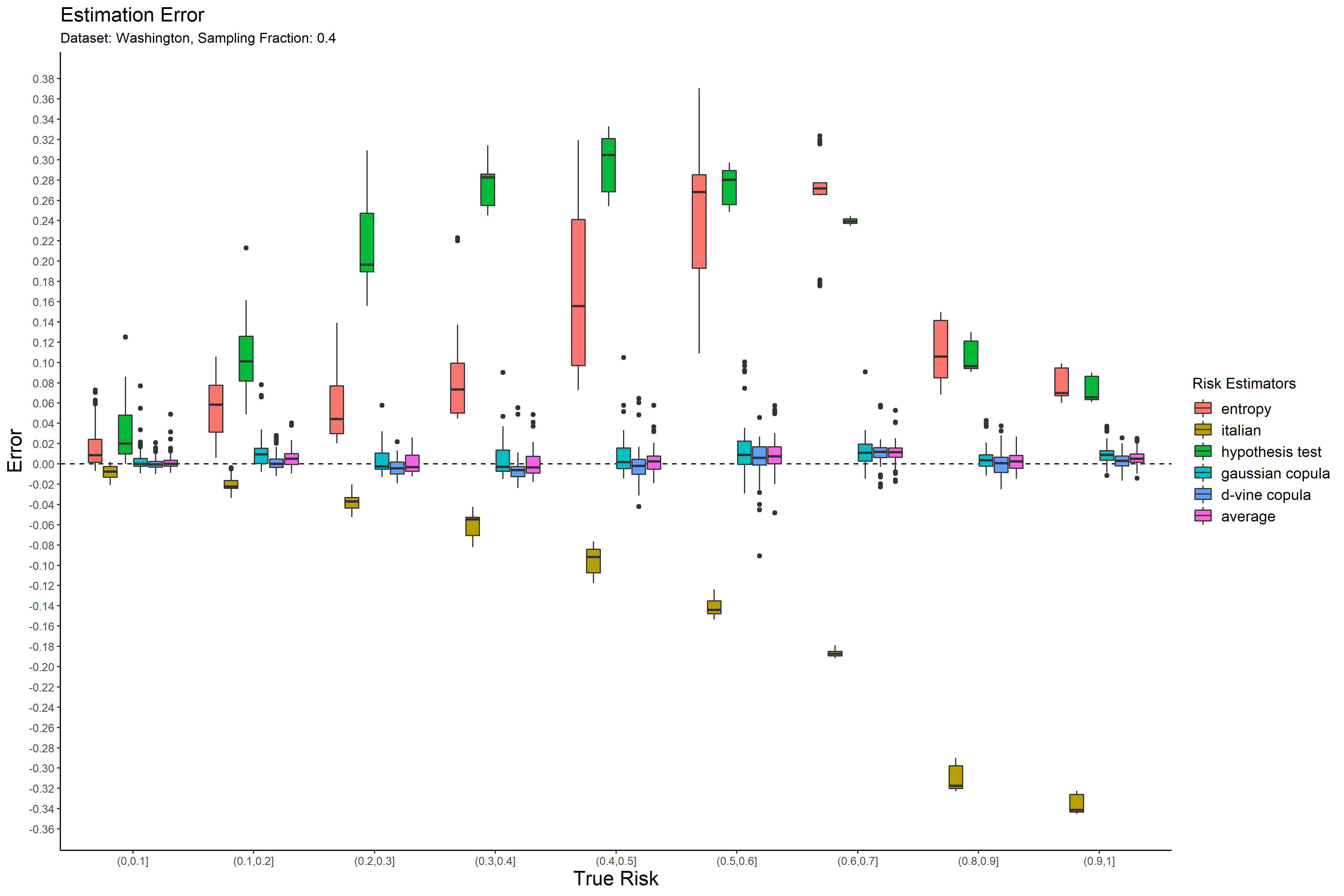

Supplement: S2 File — (ZIP) [file pone.0269097.s002.zip › wa/comparison.wa.8.png]

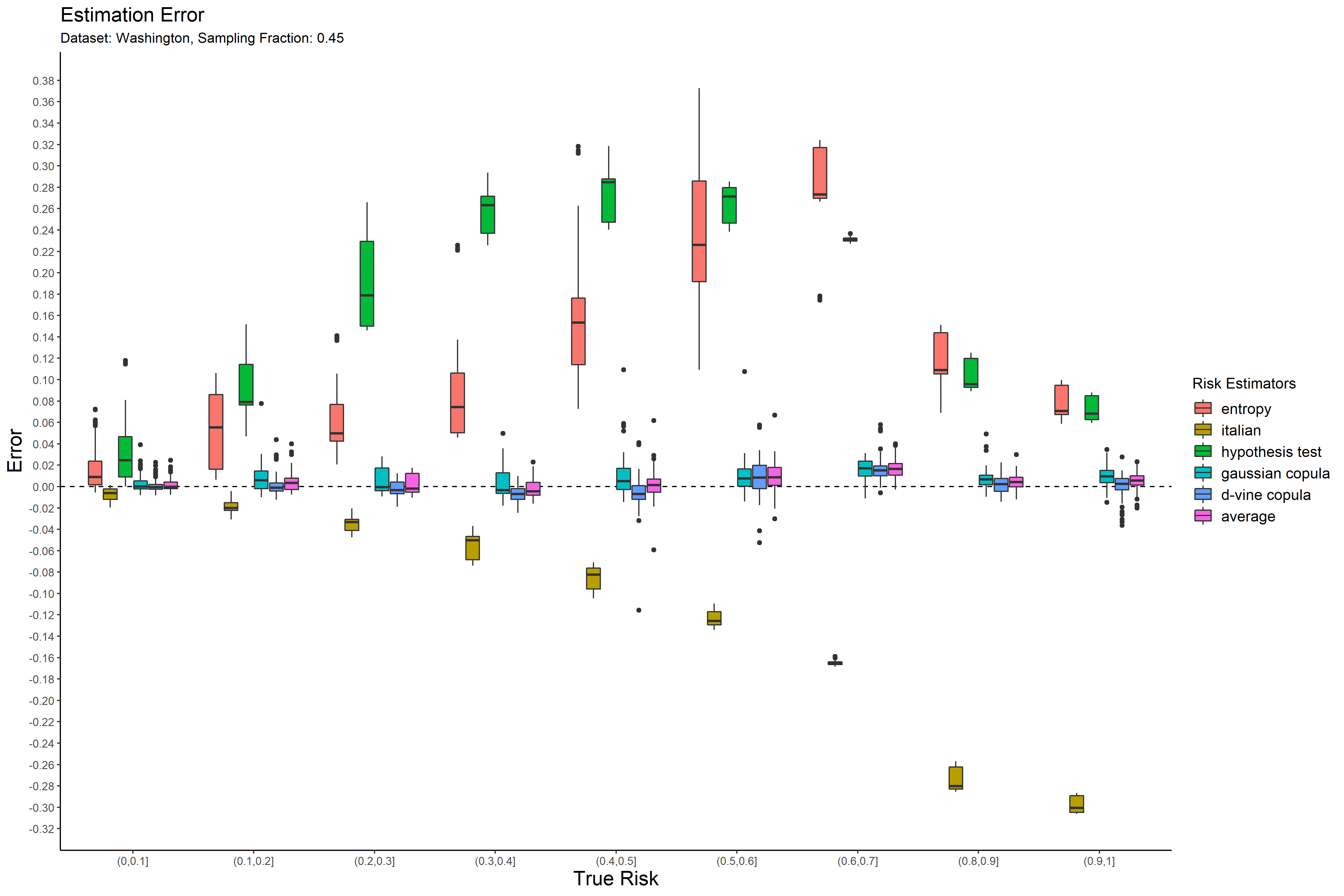

Supplement: S2 File — (ZIP) [file pone.0269097.s002.zip › wa/comparison.wa.9.png]

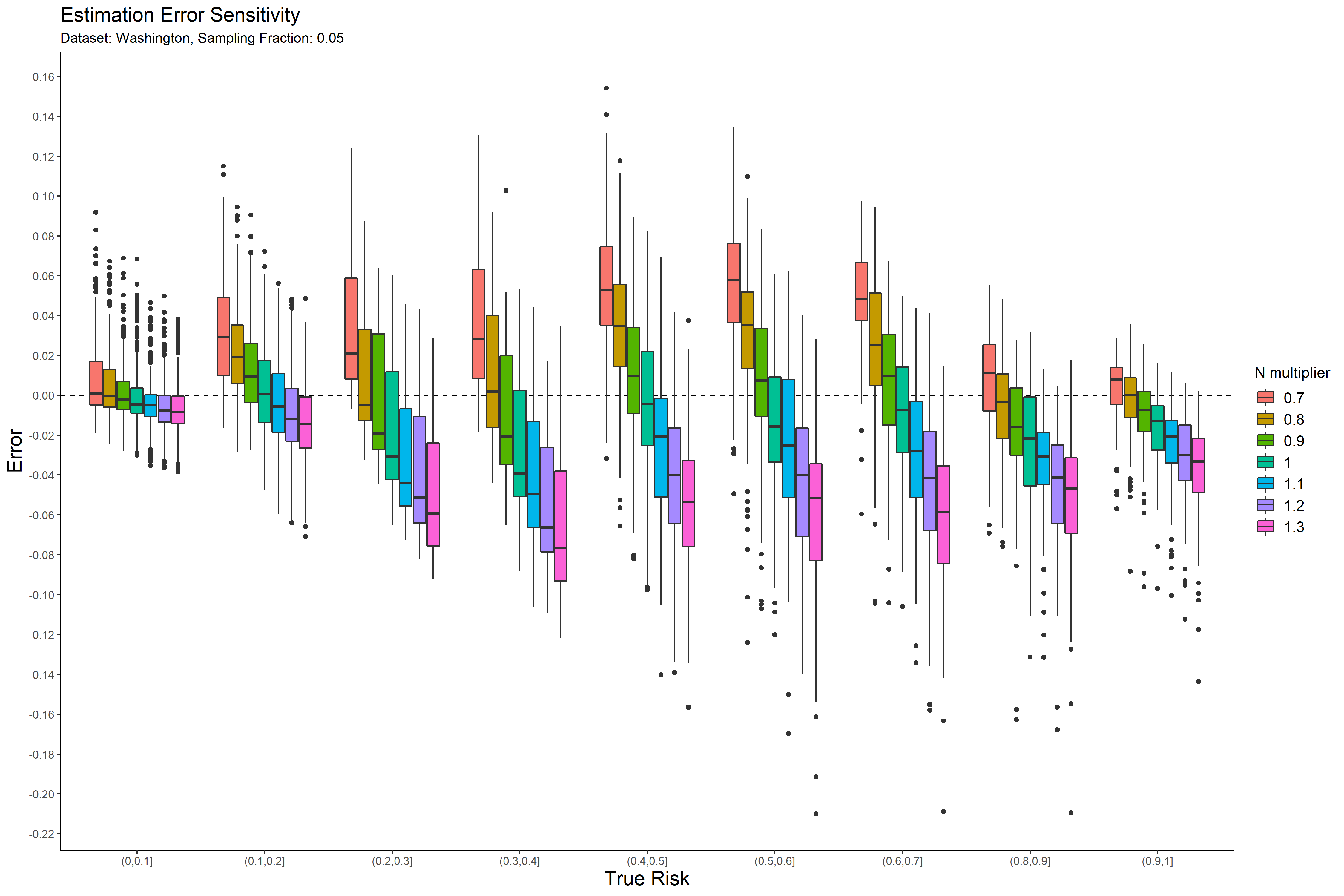

Supplement: S2 File — (ZIP) [file pone.0269097.s002.zip › wa/sensitivity.wa.1.png]

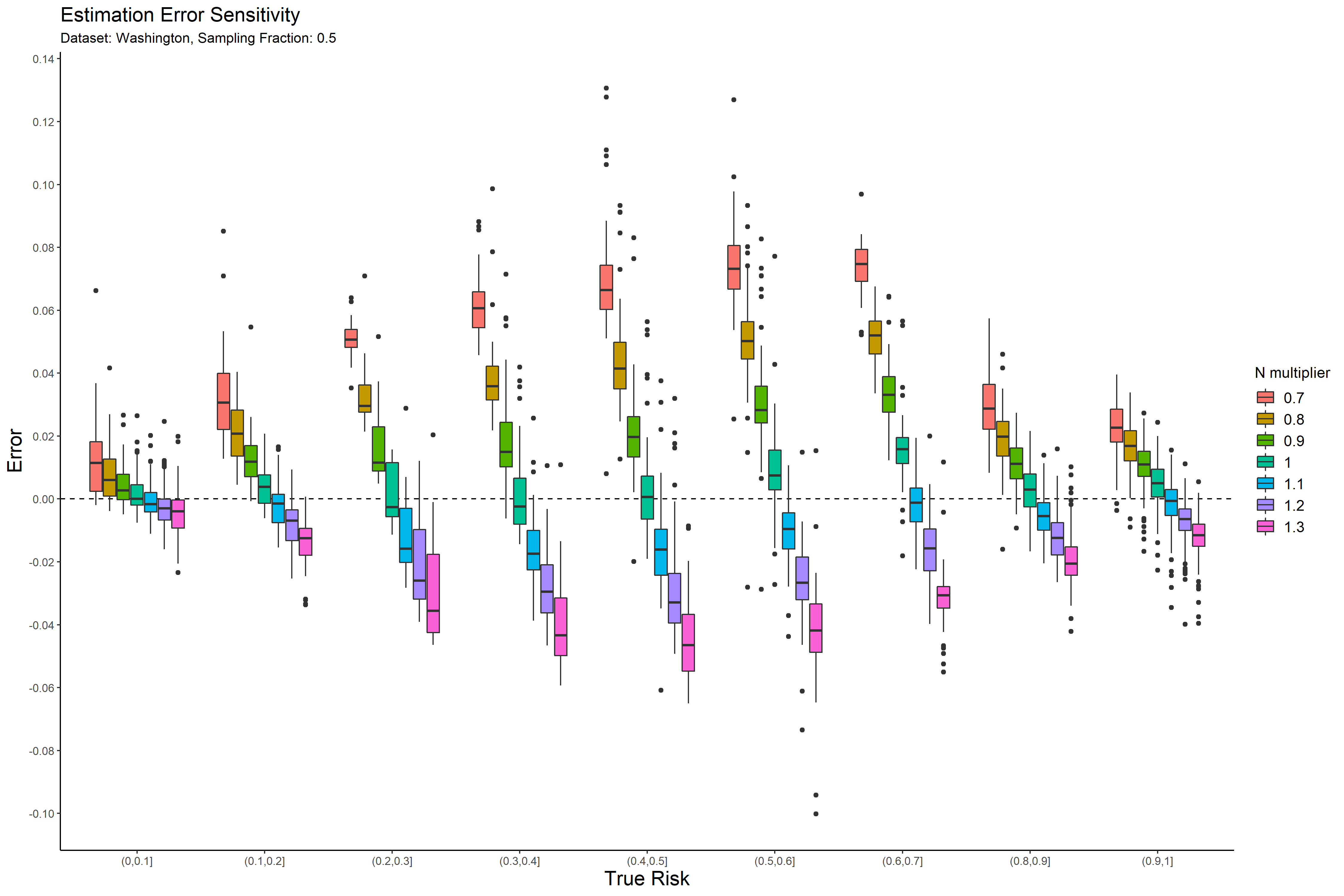

Supplement: S2 File — (ZIP) [file pone.0269097.s002.zip › wa/sensitivity.wa.10.png]

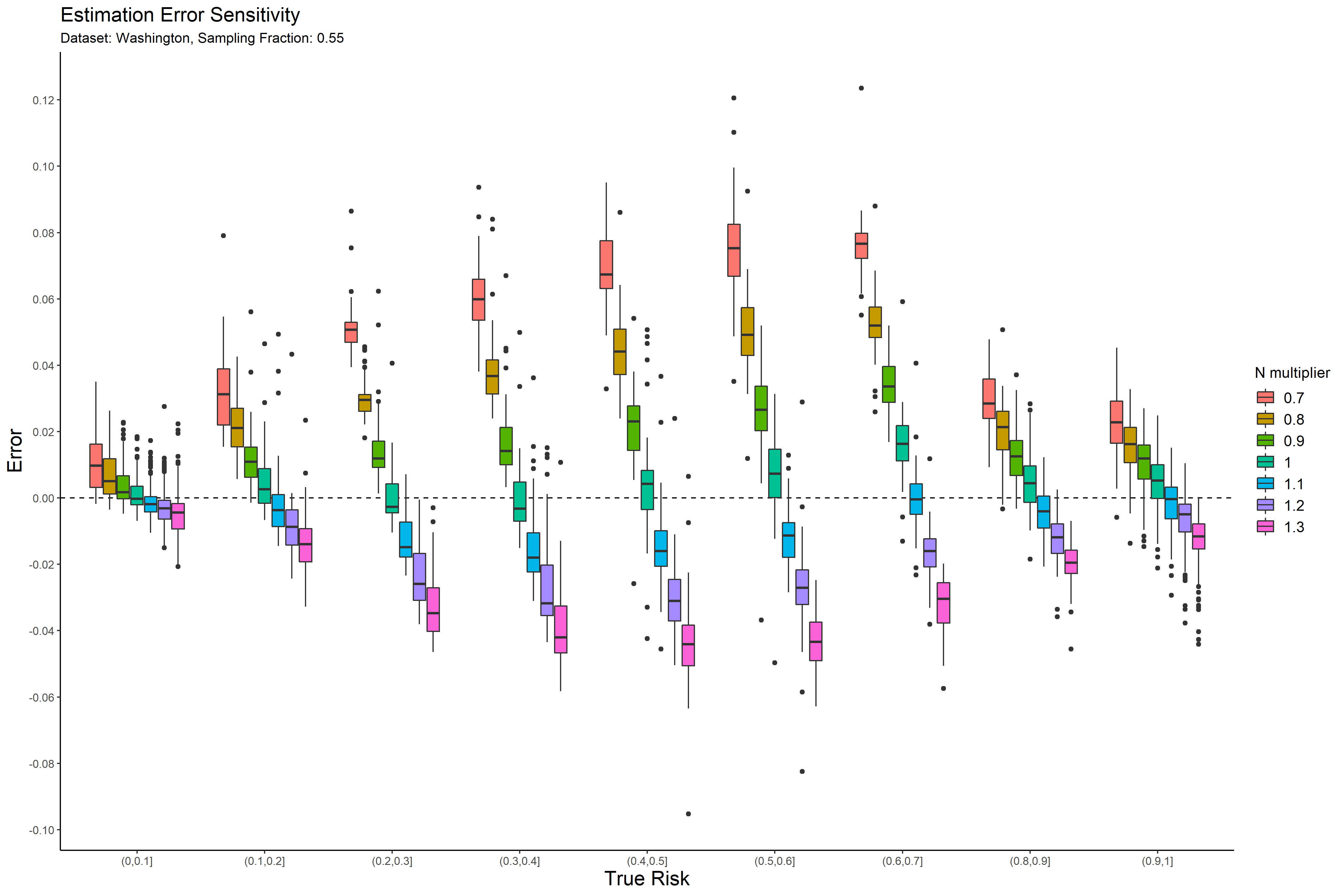

Supplement: S2 File — (ZIP) [file pone.0269097.s002.zip › wa/sensitivity.wa.11.png]

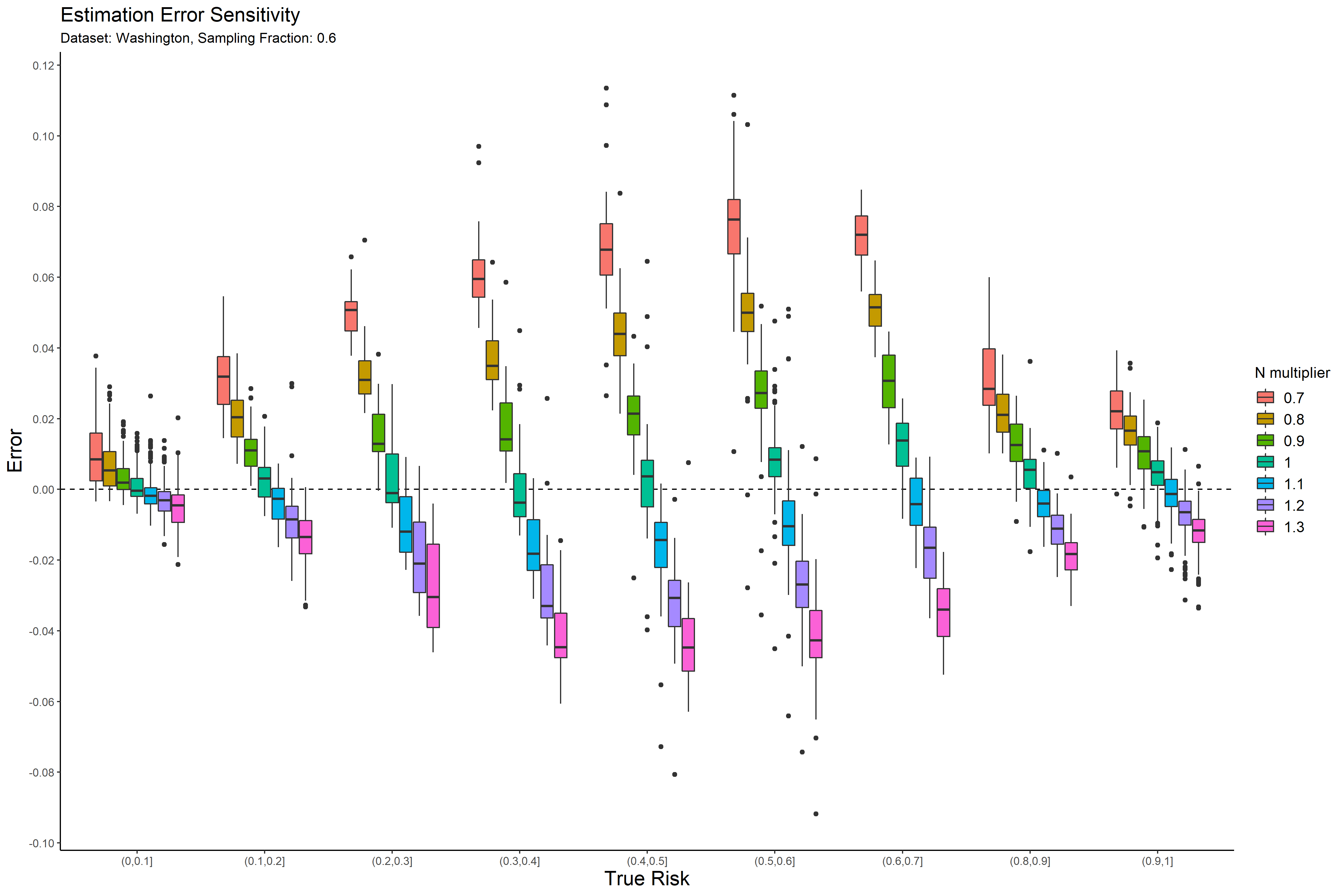

Supplement: S2 File — (ZIP) [file pone.0269097.s002.zip › wa/sensitivity.wa.12.png]

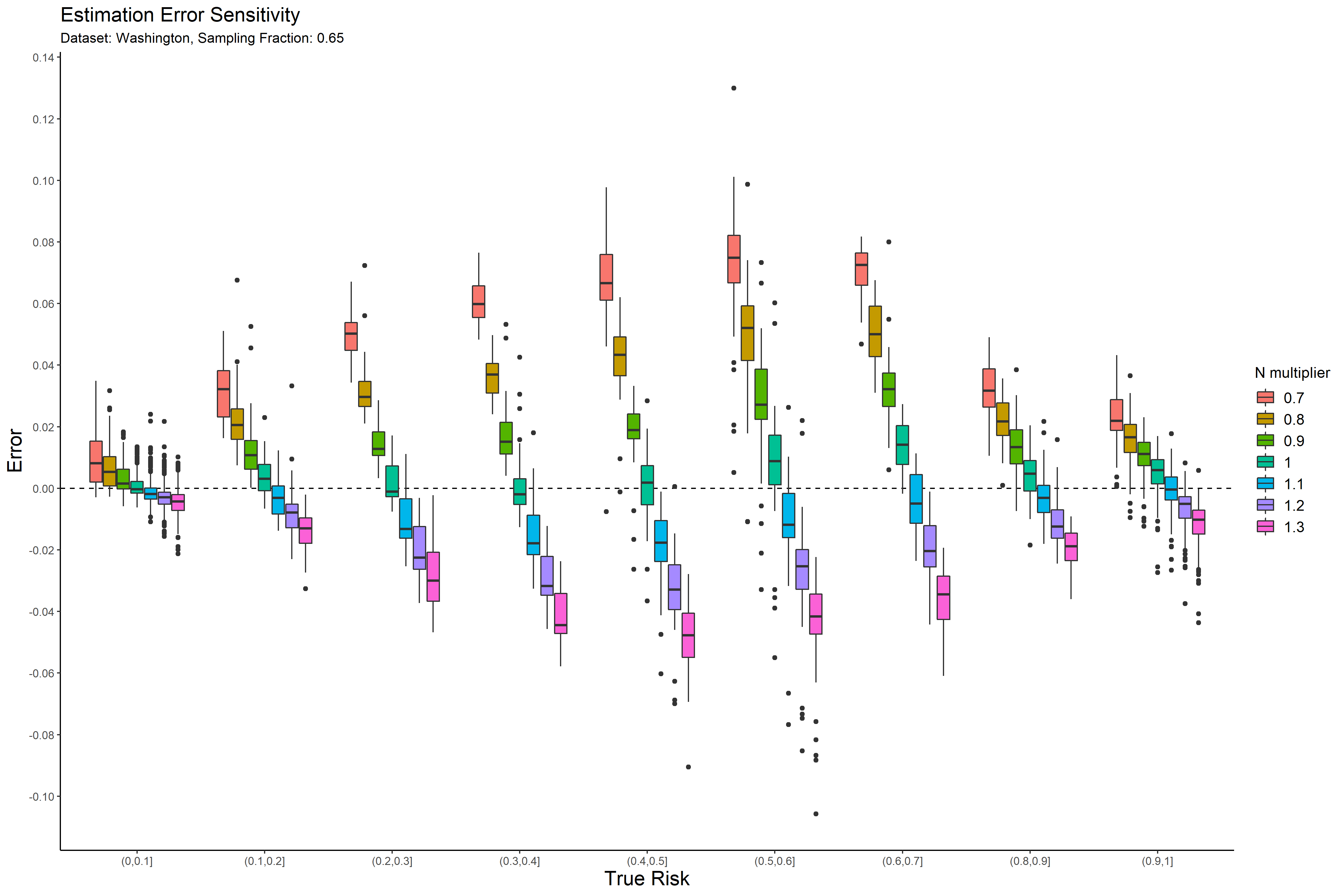

Supplement: S2 File — (ZIP) [file pone.0269097.s002.zip › wa/sensitivity.wa.13.png]

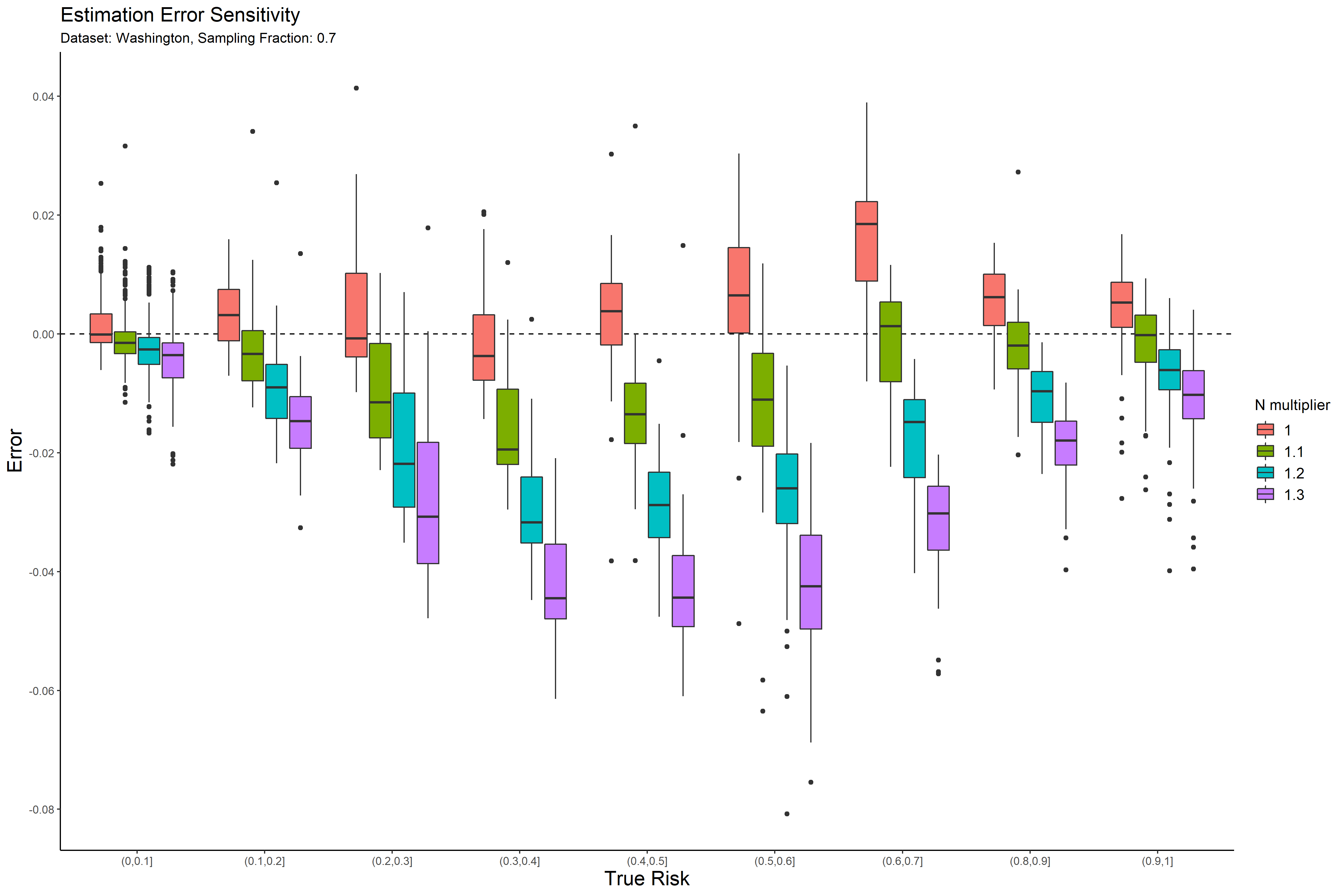

Supplement: S2 File — (ZIP) [file pone.0269097.s002.zip › wa/sensitivity.wa.14.png]

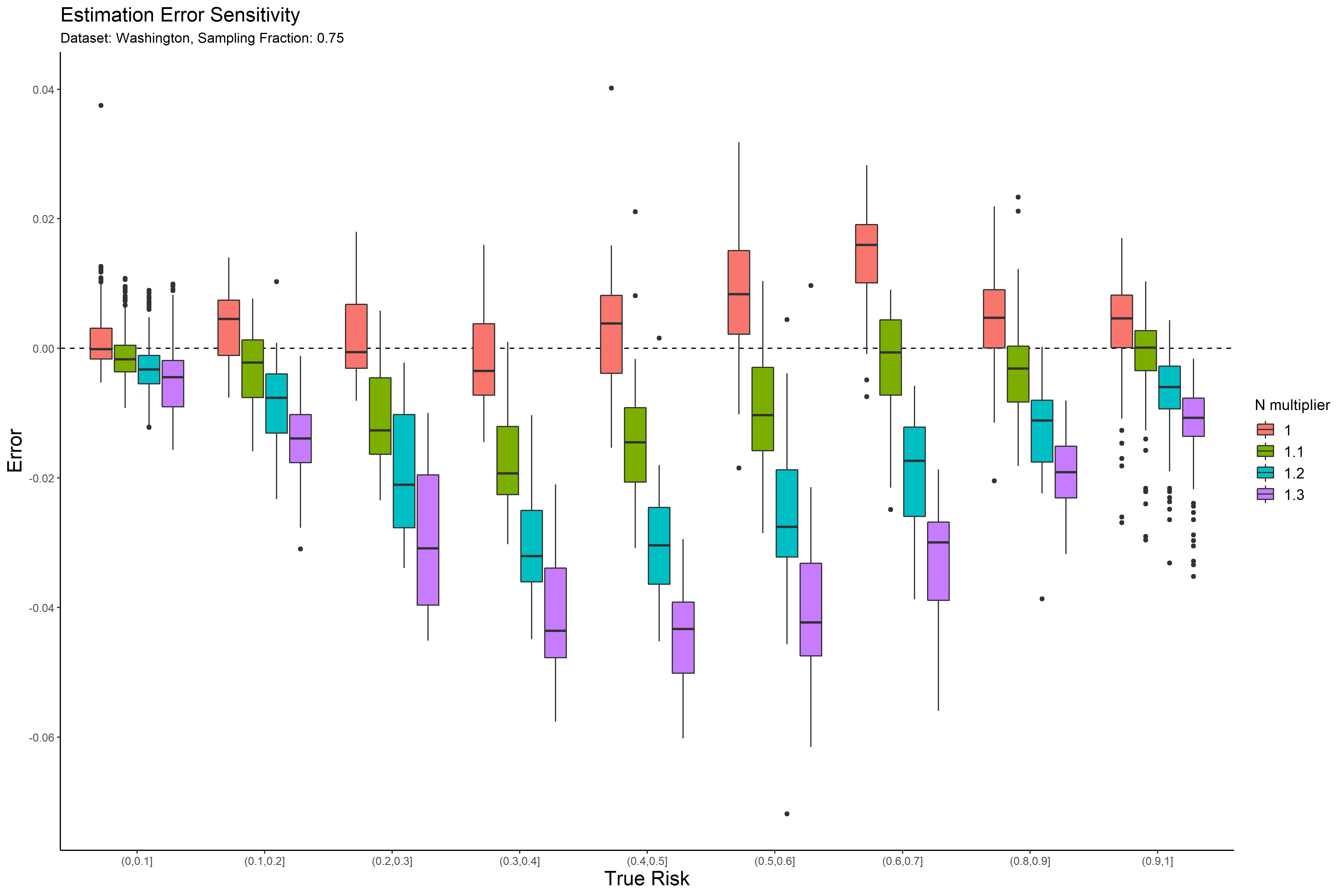

Supplement: S2 File — (ZIP) [file pone.0269097.s002.zip › wa/sensitivity.wa.15.png]

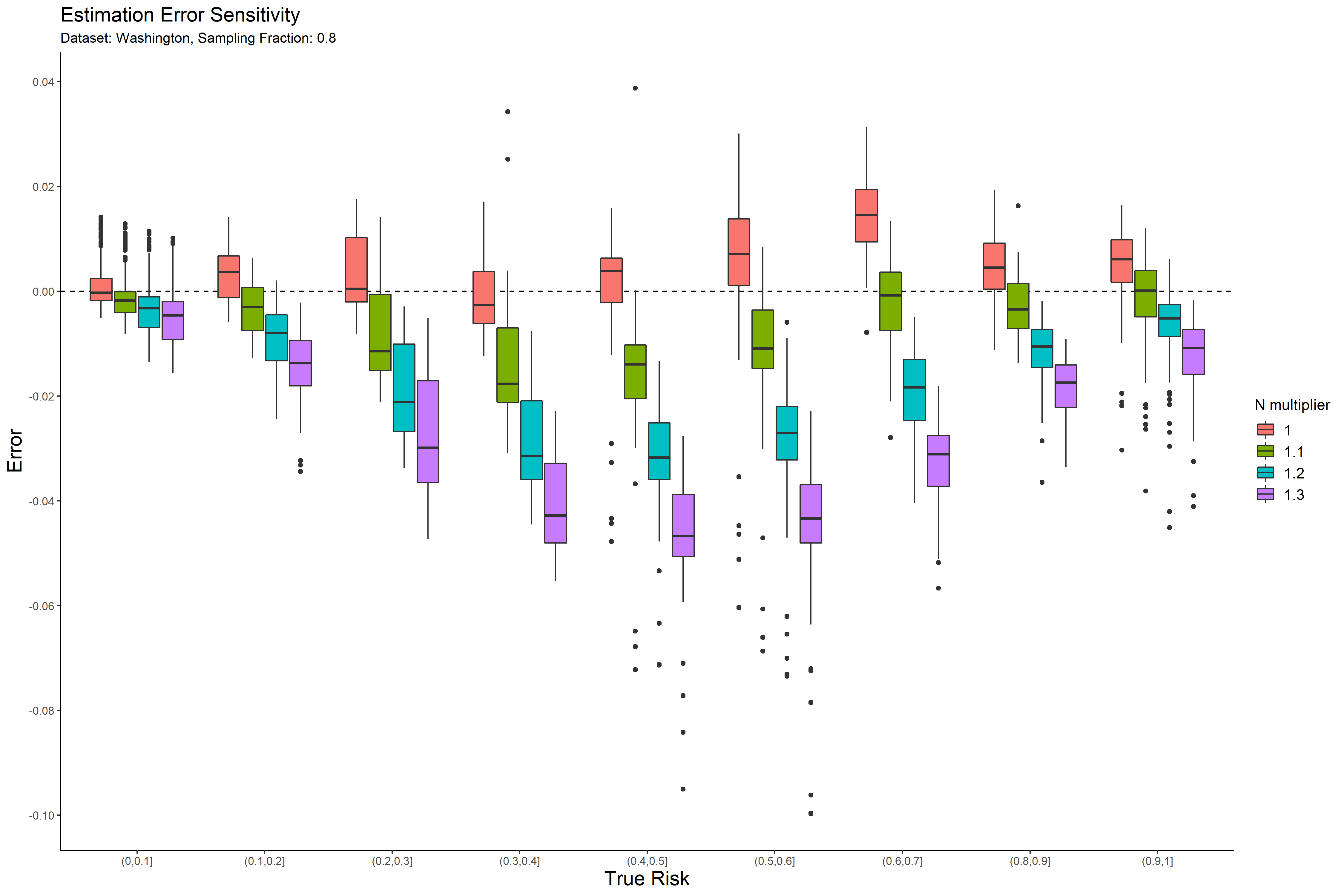

Supplement: S2 File — (ZIP) [file pone.0269097.s002.zip › wa/sensitivity.wa.16.png]

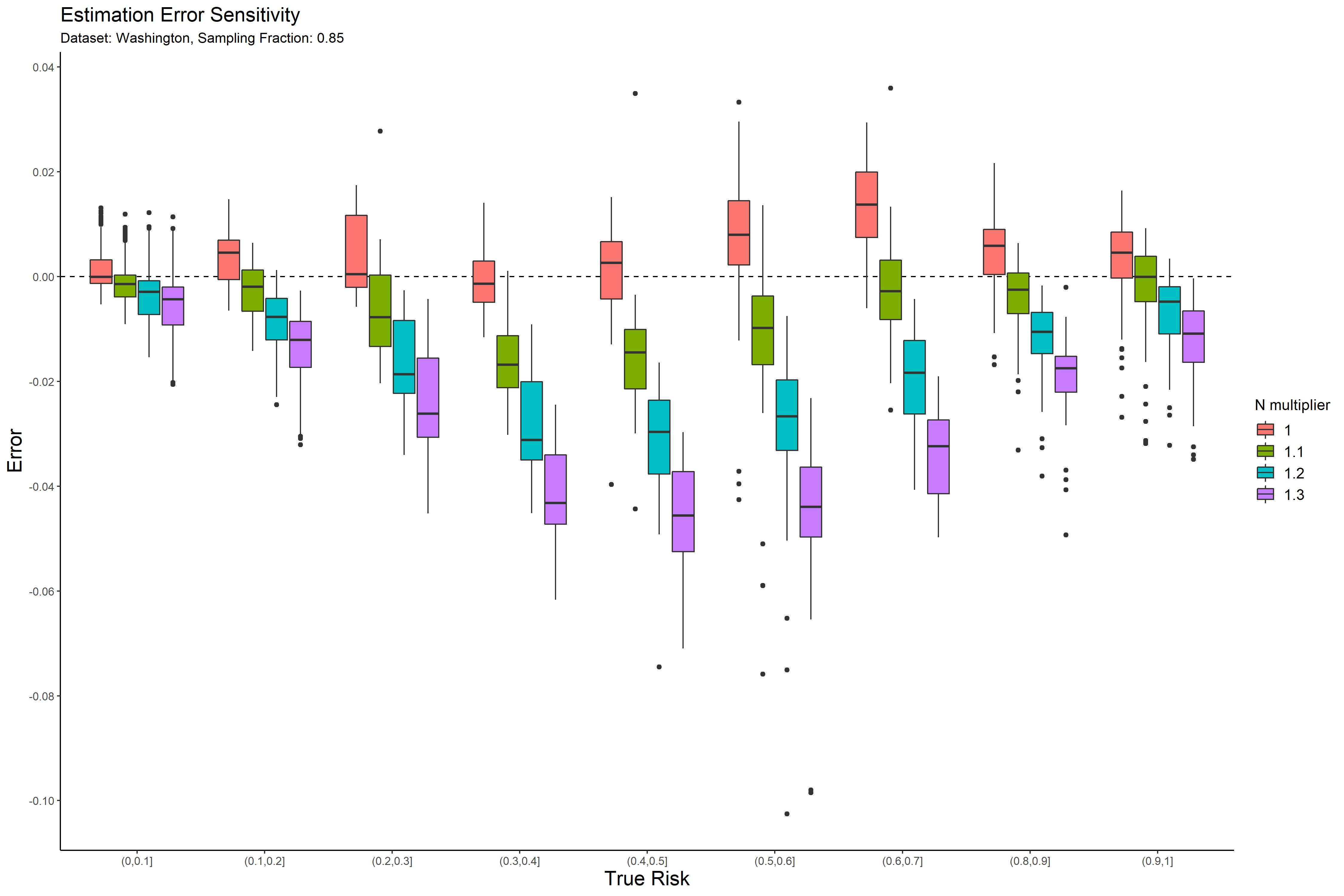

Supplement: S2 File — (ZIP) [file pone.0269097.s002.zip › wa/sensitivity.wa.17.png]

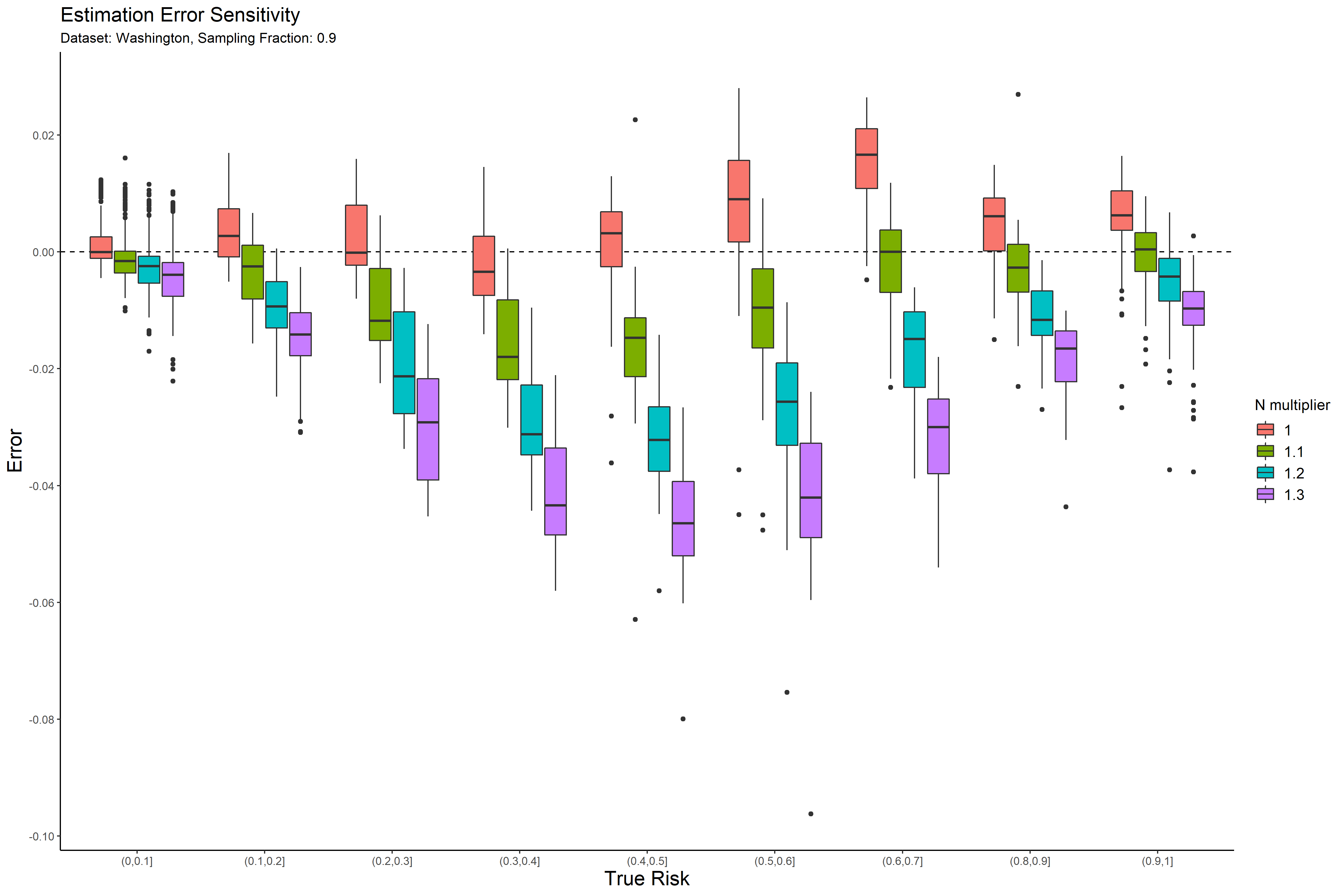

Supplement: S2 File — (ZIP) [file pone.0269097.s002.zip › wa/sensitivity.wa.18.png]

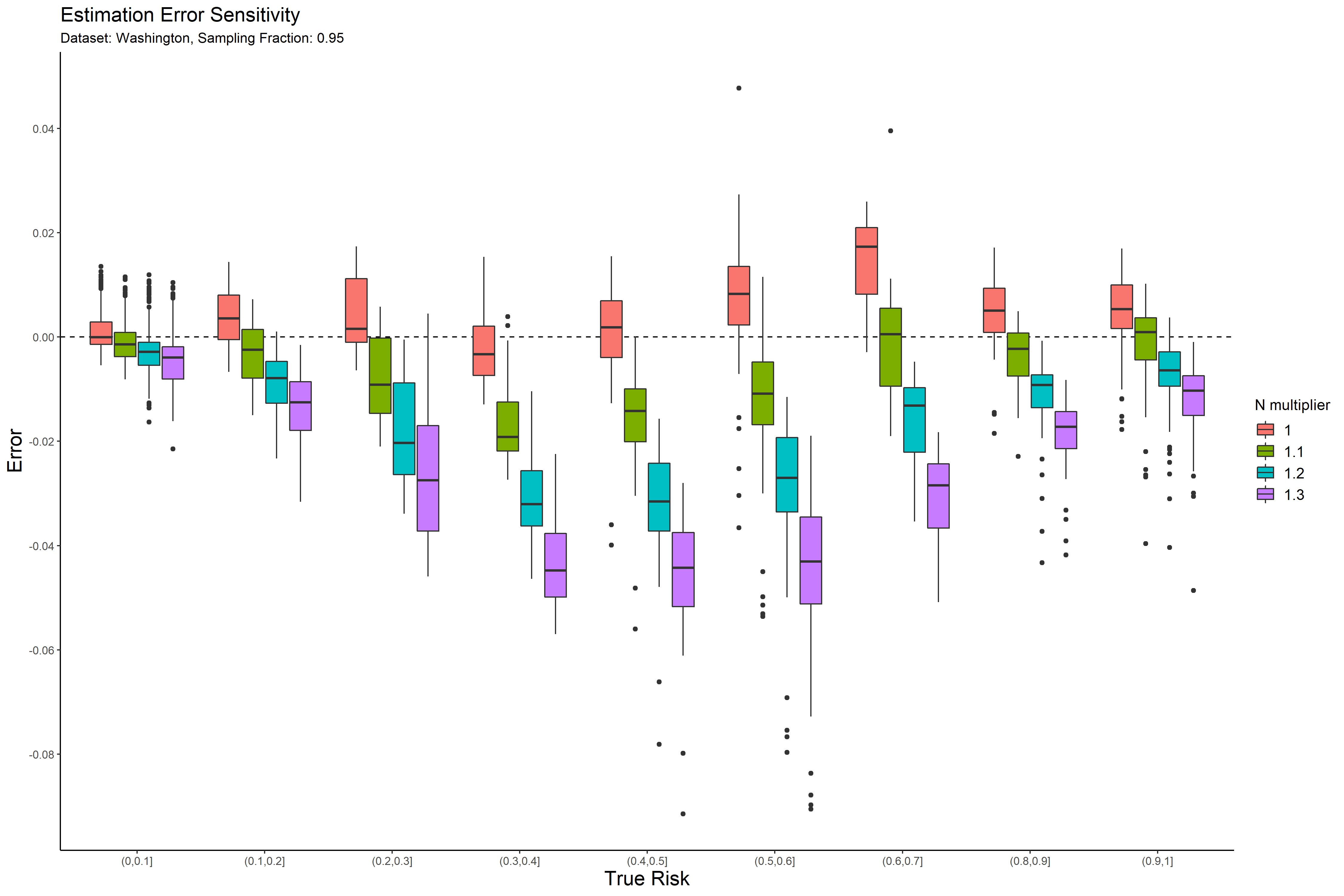

Supplement: S2 File — (ZIP) [file pone.0269097.s002.zip › wa/sensitivity.wa.19.png]

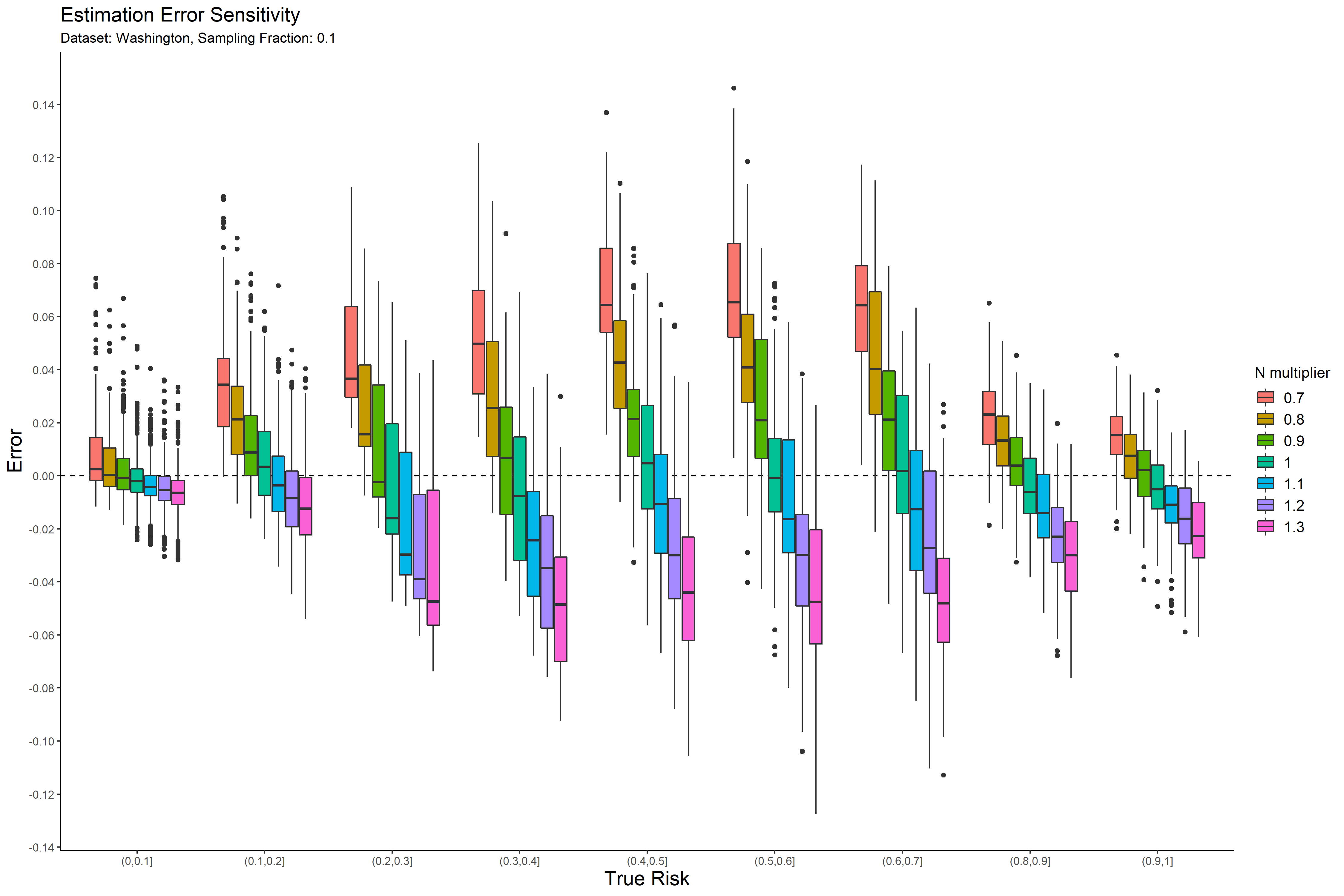

Supplement: S2 File — (ZIP) [file pone.0269097.s002.zip › wa/sensitivity.wa.2.png]

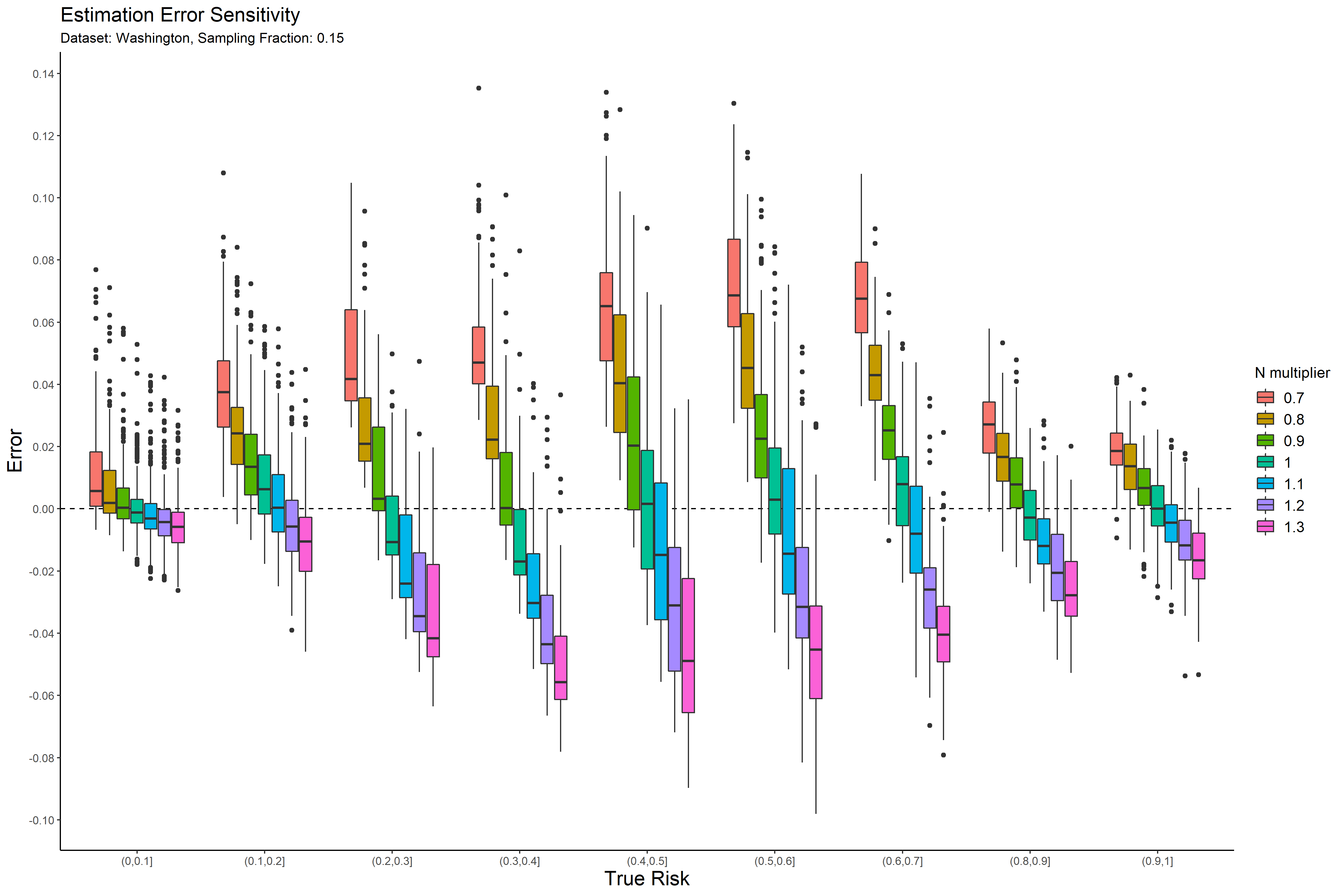

Supplement: S2 File — (ZIP) [file pone.0269097.s002.zip › wa/sensitivity.wa.3.png]

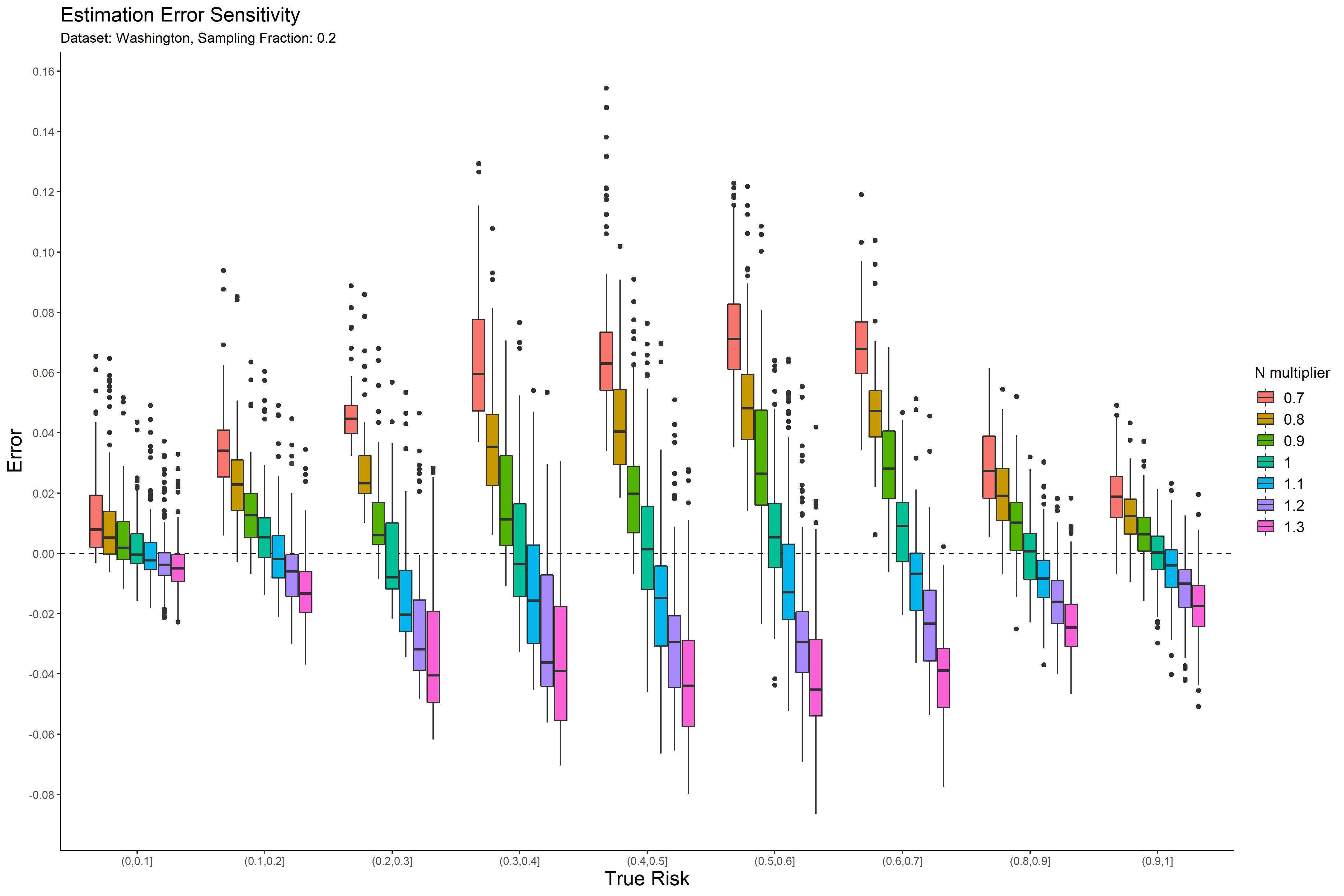

Supplement: S2 File — (ZIP) [file pone.0269097.s002.zip › wa/sensitivity.wa.4.png]

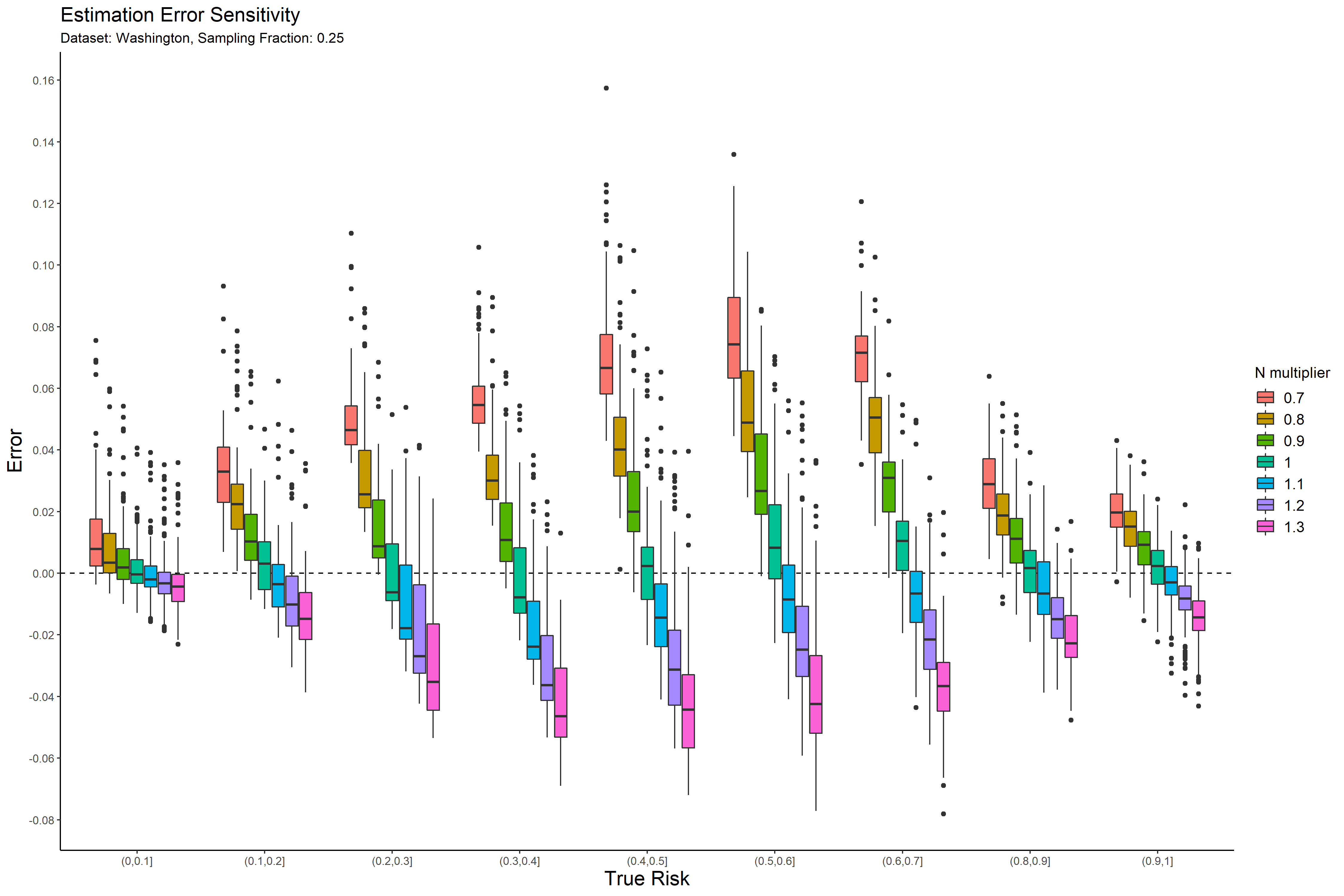

Supplement: S2 File — (ZIP) [file pone.0269097.s002.zip › wa/sensitivity.wa.5.png]

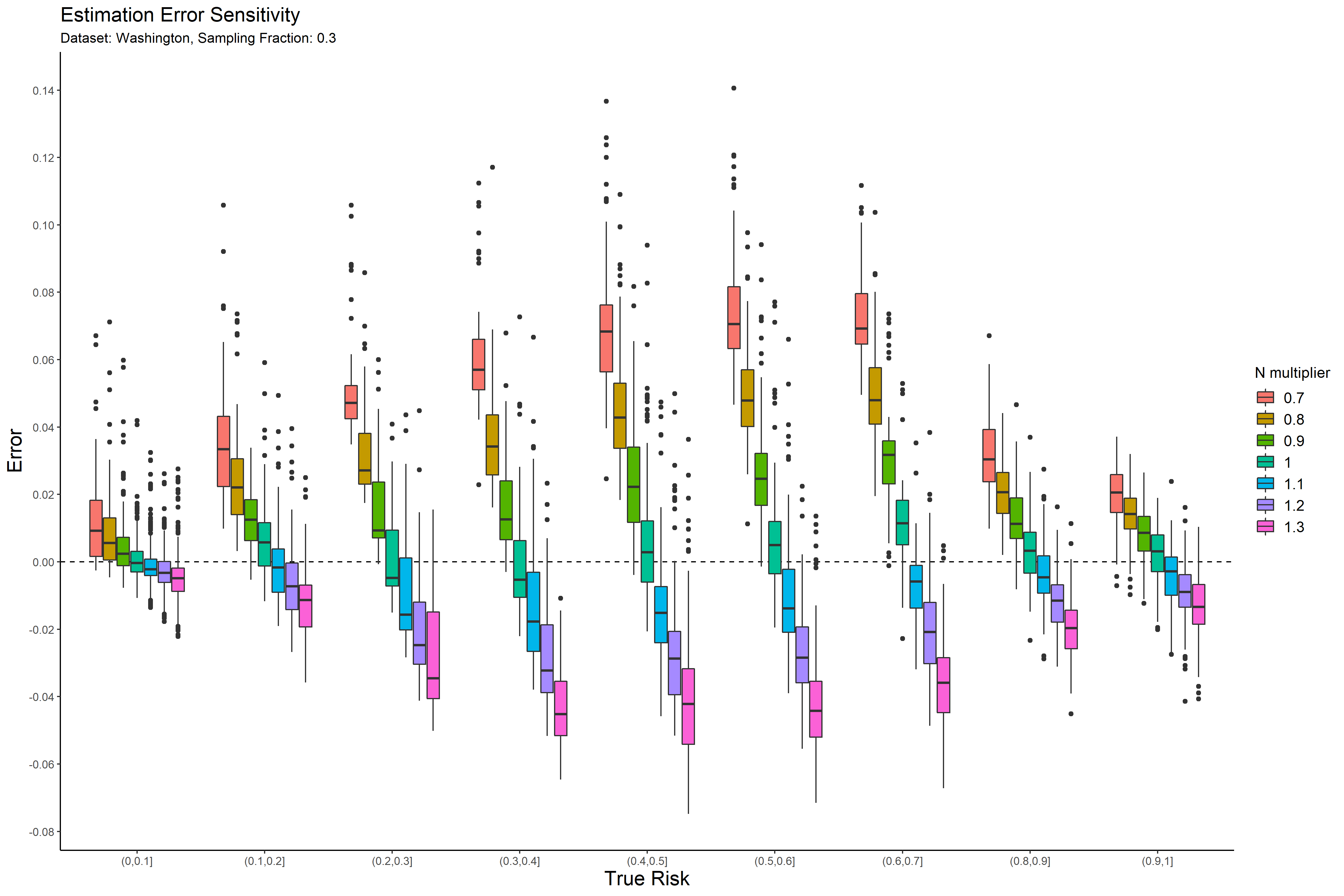

Supplement: S2 File — (ZIP) [file pone.0269097.s002.zip › wa/sensitivity.wa.6.png]

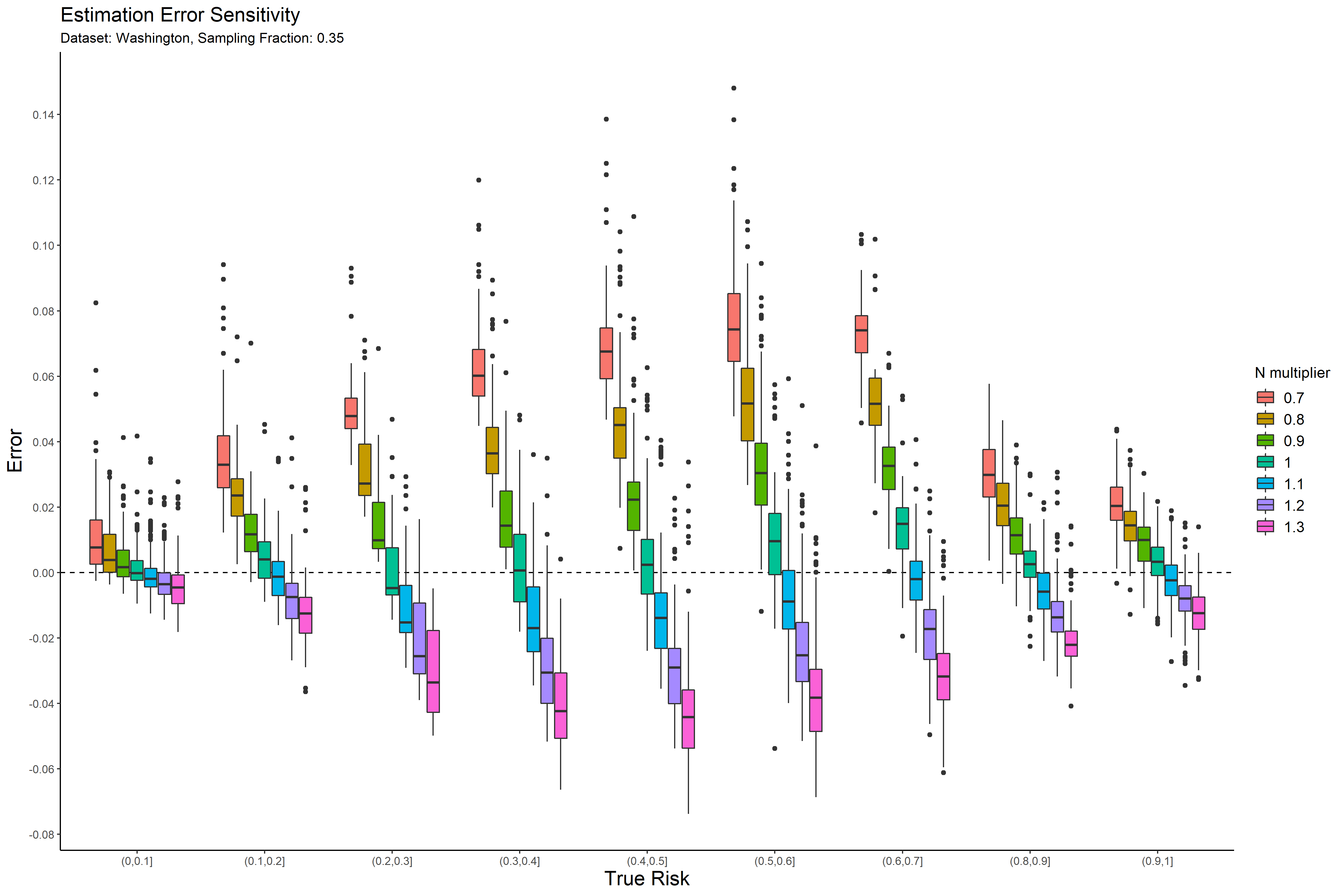

Supplement: S2 File — (ZIP) [file pone.0269097.s002.zip › wa/sensitivity.wa.7.png]

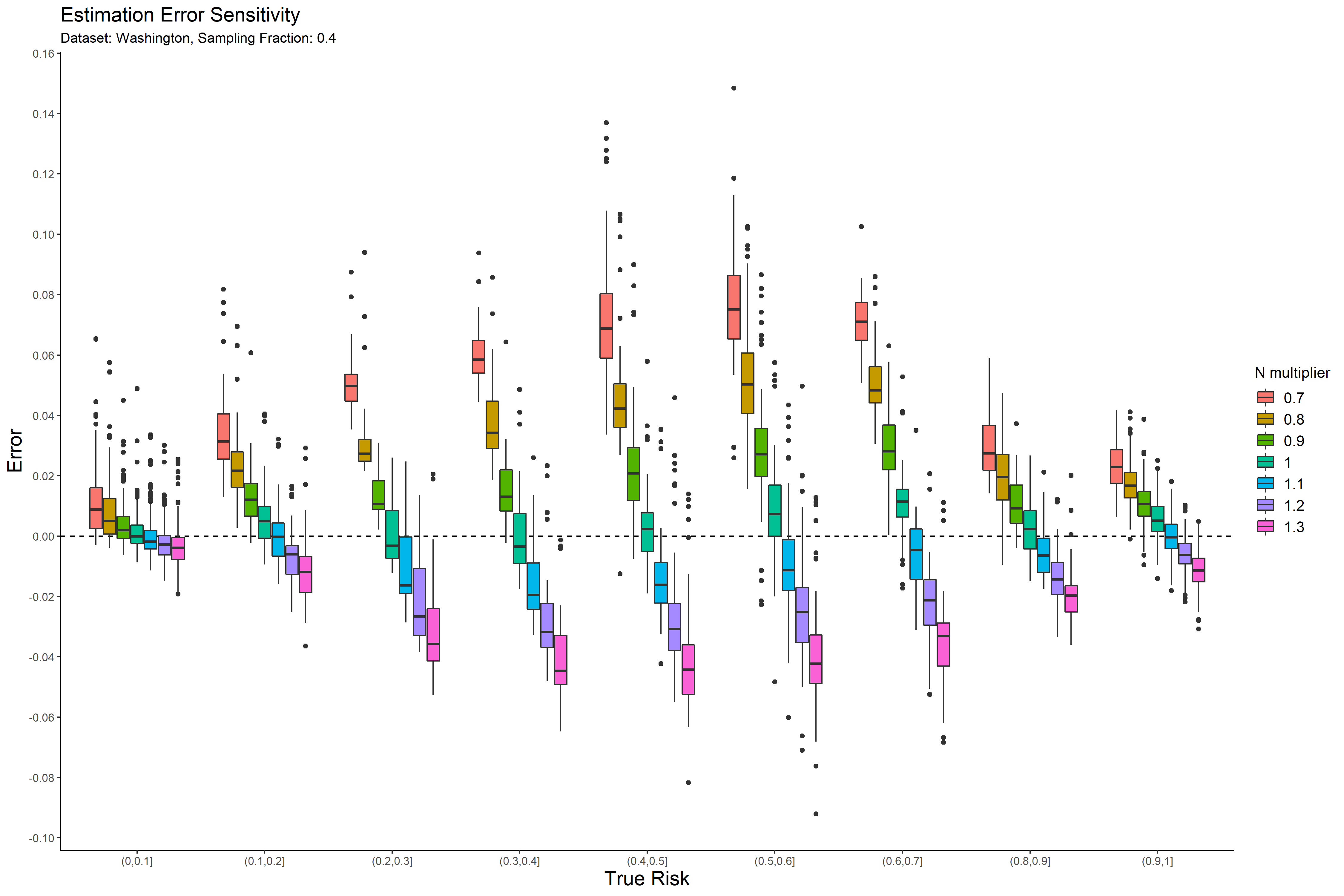

Supplement: S2 File — (ZIP) [file pone.0269097.s002.zip › wa/sensitivity.wa.8.png]

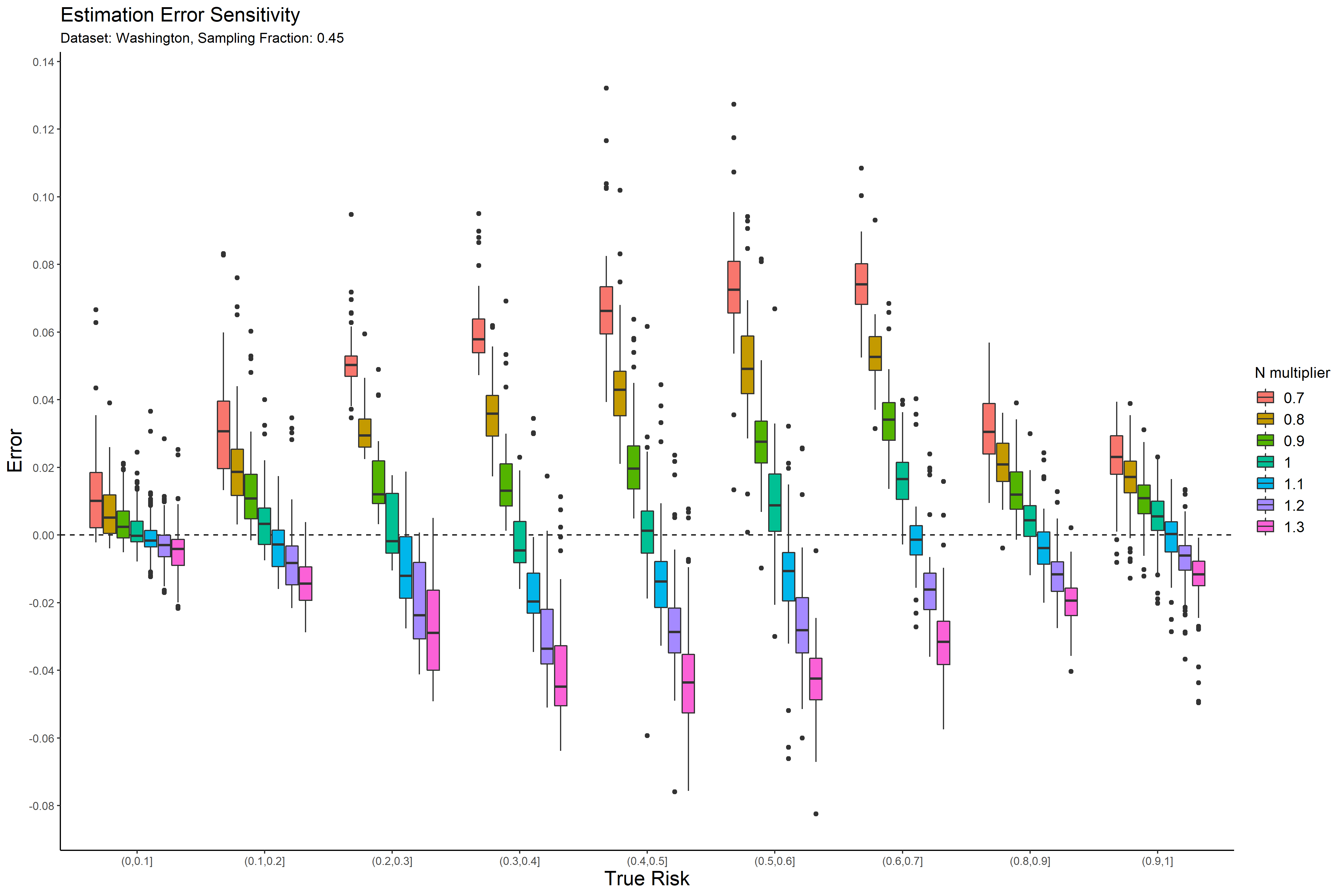

Supplement: S2 File — (ZIP) [file pone.0269097.s002.zip › wa/sensitivity.wa.9.png]
