# Supplementary figures and images for: Measuring re-identification risk using a synthetic estimator to enable data sharing (part 1 of 2)
Source: PLoS One. 2022 Jun 17;17(6):e0269097. doi: 10.1371/journal.pone.0269097 (PMC9205507; doi:10.1371/journal.pone.0269097)

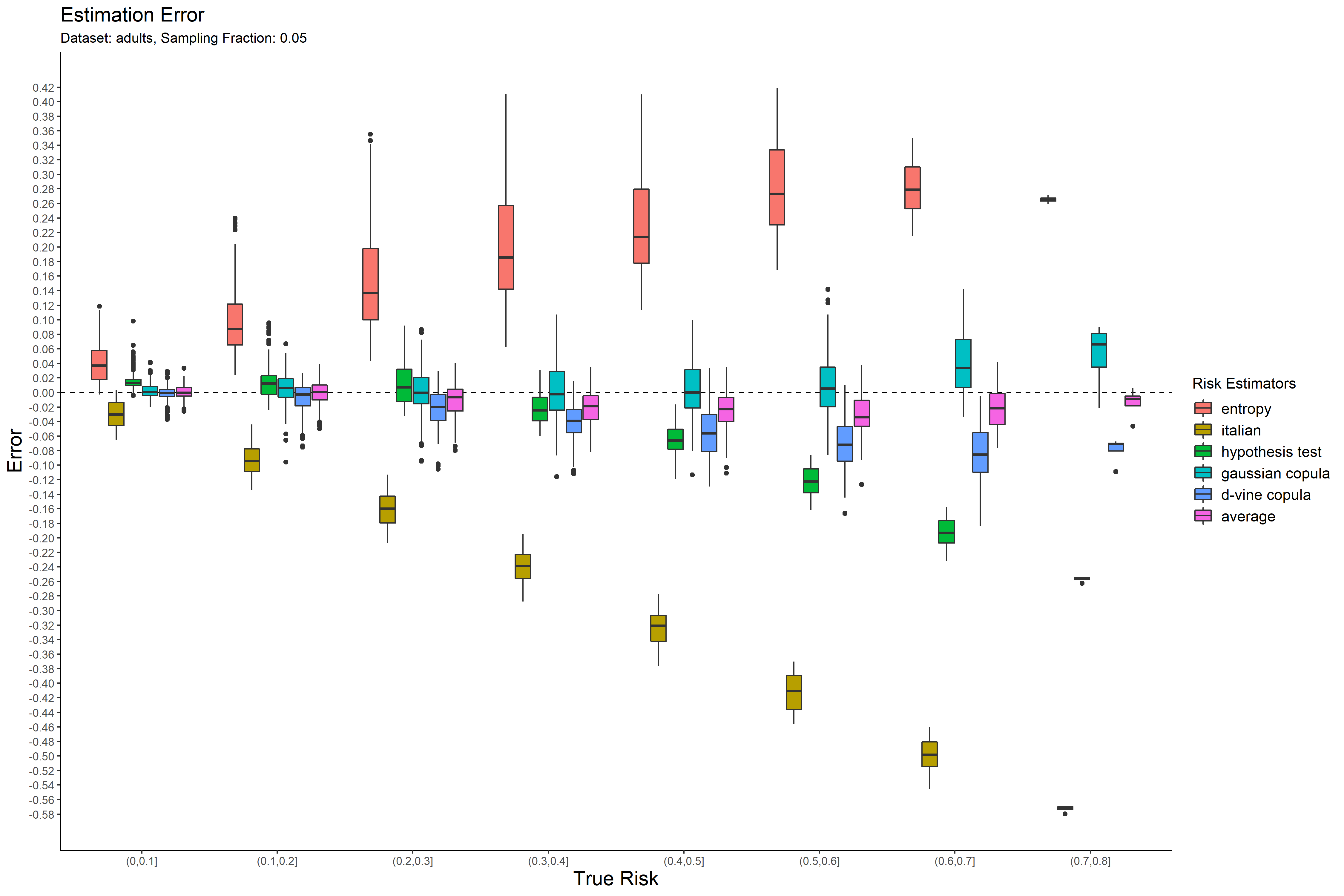

Supplement: S2 File — (ZIP) [file pone.0269097.s002.zip › adults/comparison.adults.1.png]

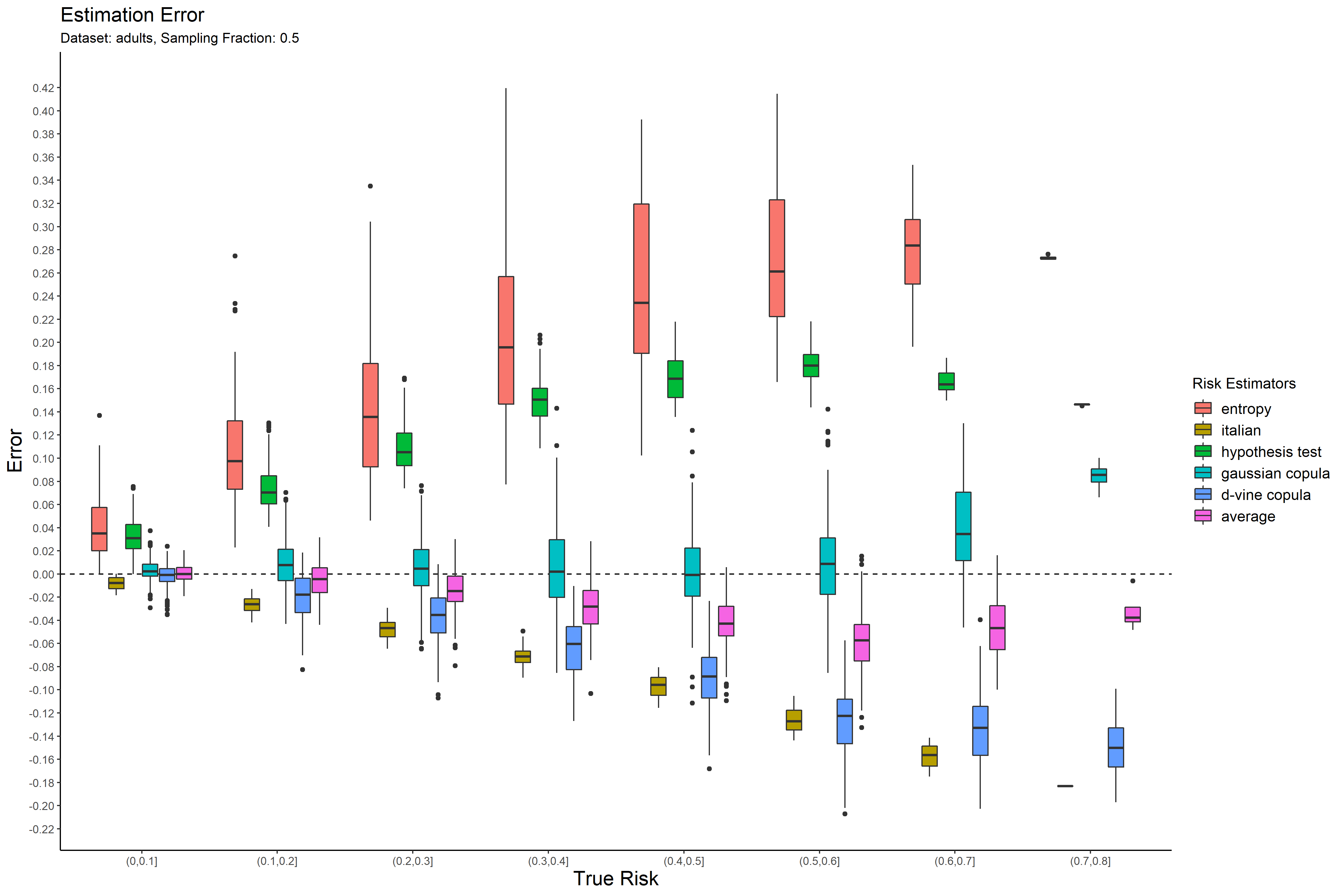

Supplement: S2 File — (ZIP) [file pone.0269097.s002.zip › adults/comparison.adults.10.png]

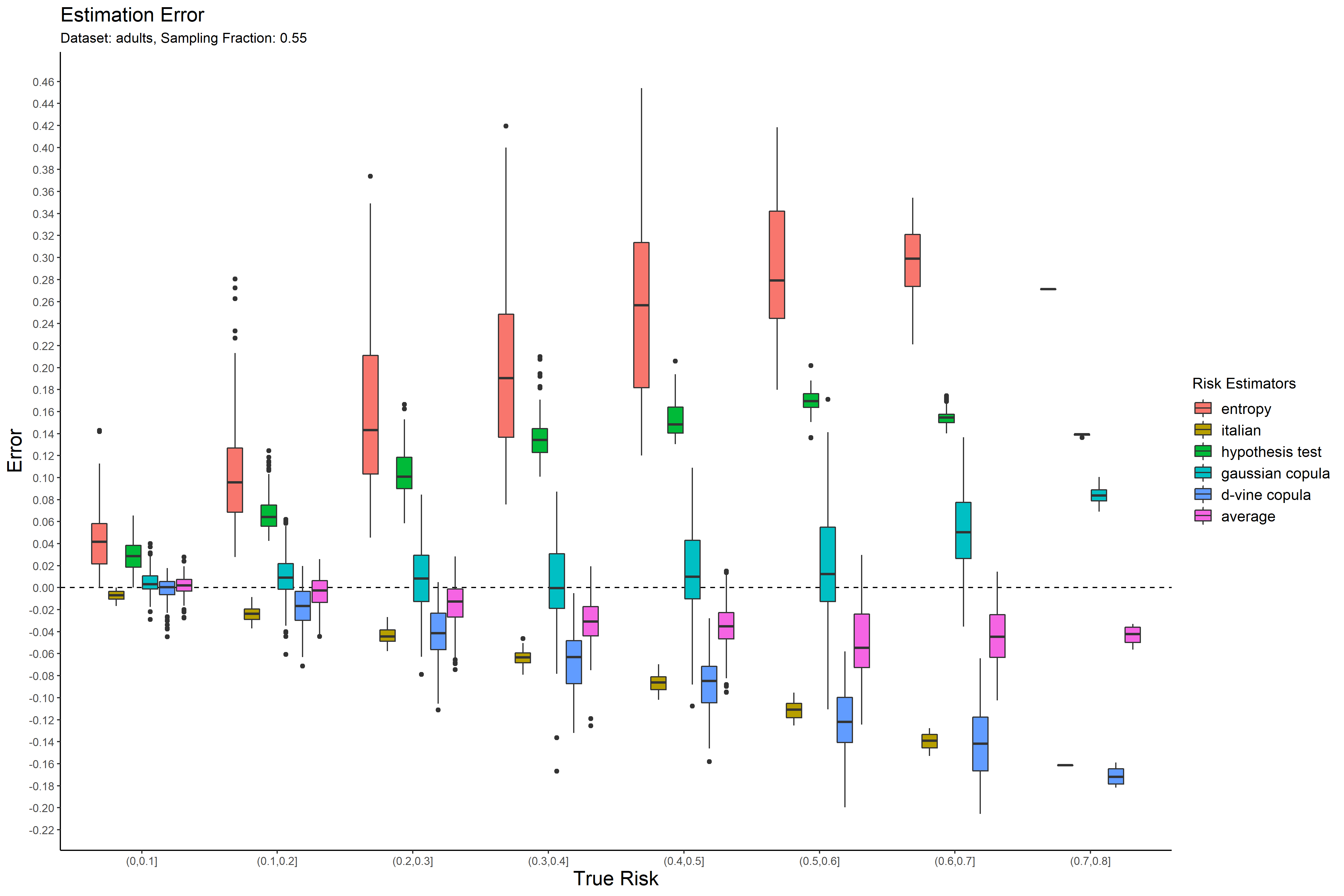

Supplement: S2 File — (ZIP) [file pone.0269097.s002.zip › adults/comparison.adults.11.png]

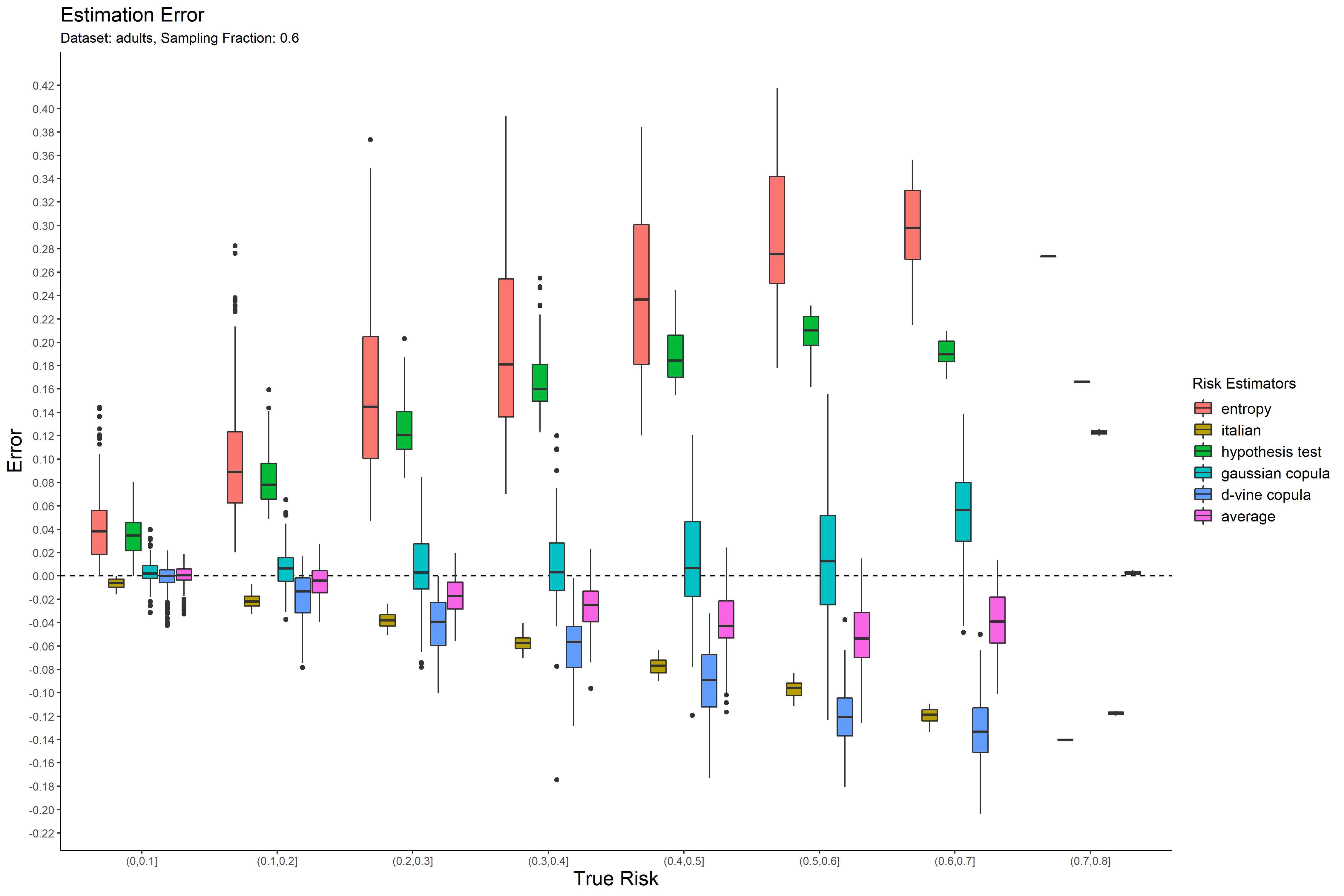

Supplement: S2 File — (ZIP) [file pone.0269097.s002.zip › adults/comparison.adults.12.png]

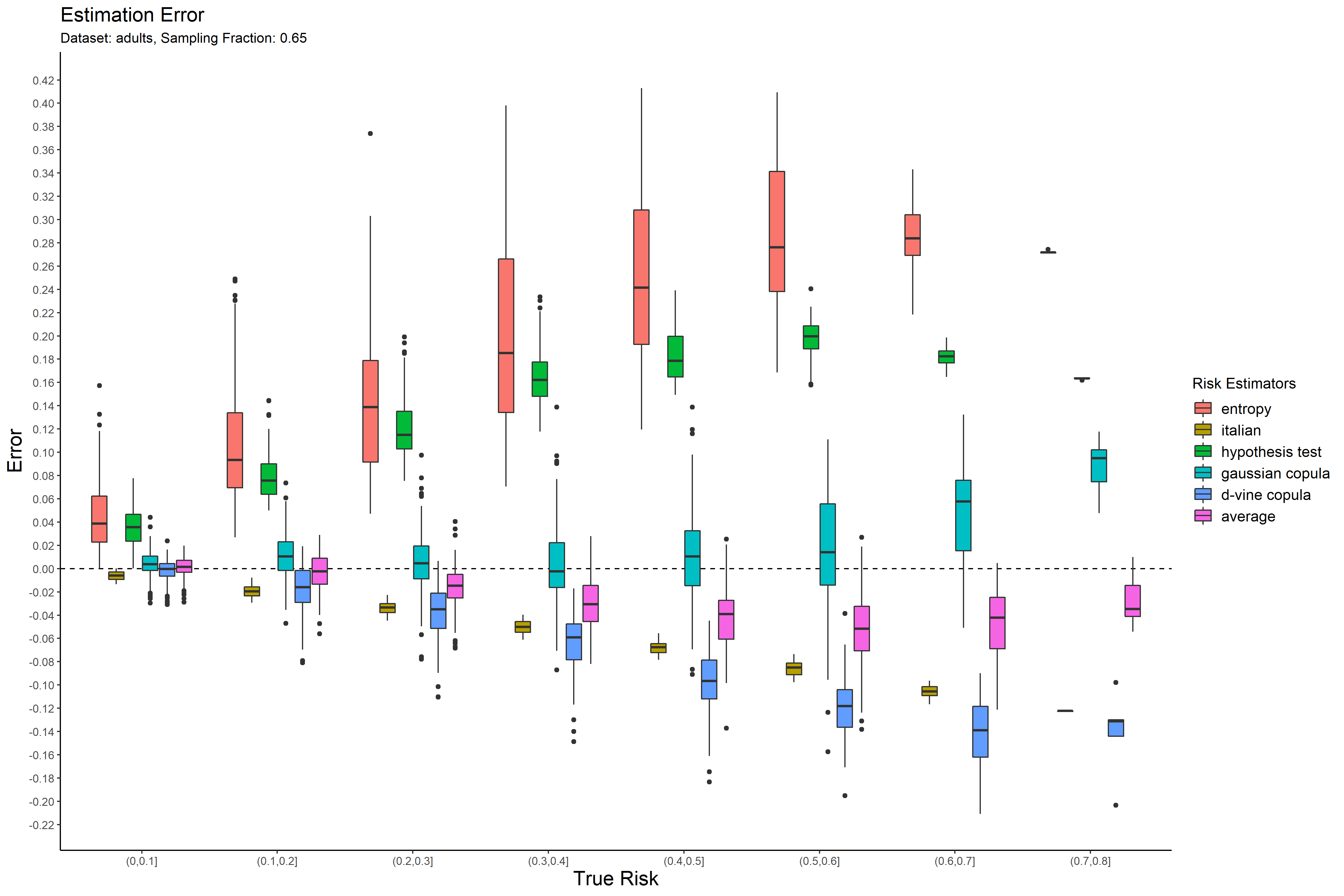

Supplement: S2 File — (ZIP) [file pone.0269097.s002.zip › adults/comparison.adults.13.png]

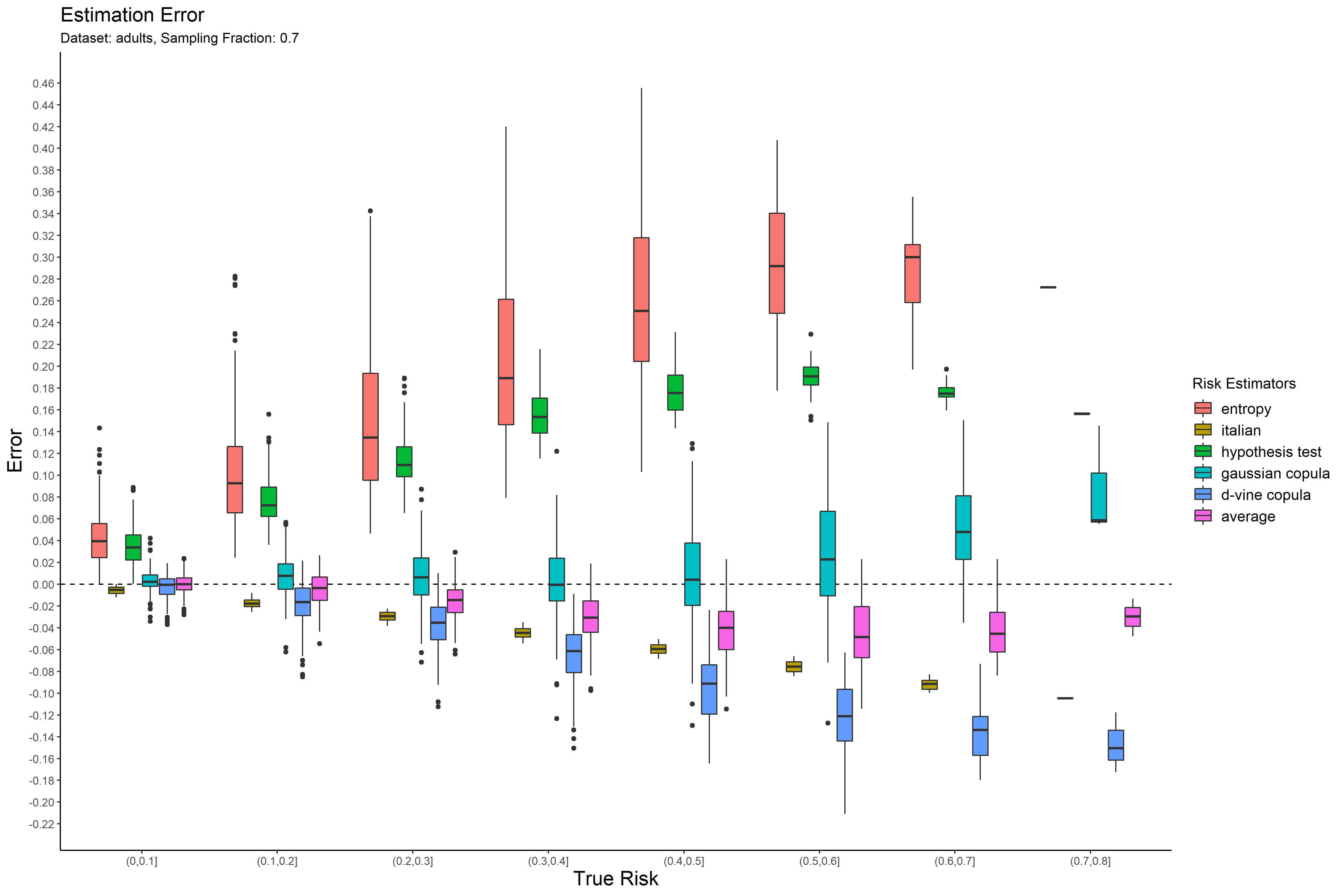

Supplement: S2 File — (ZIP) [file pone.0269097.s002.zip › adults/comparison.adults.14.png]

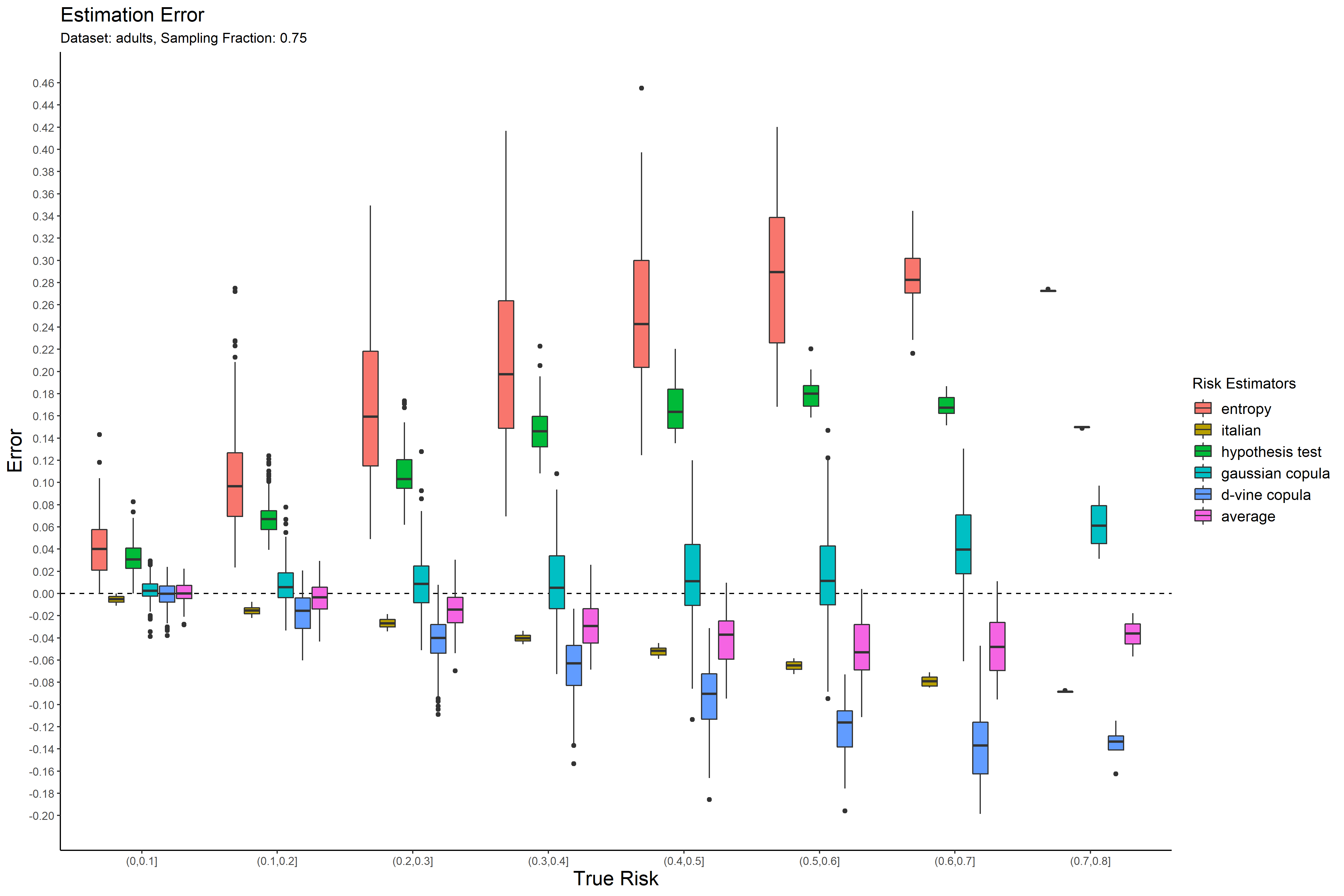

Supplement: S2 File — (ZIP) [file pone.0269097.s002.zip › adults/comparison.adults.15.png]

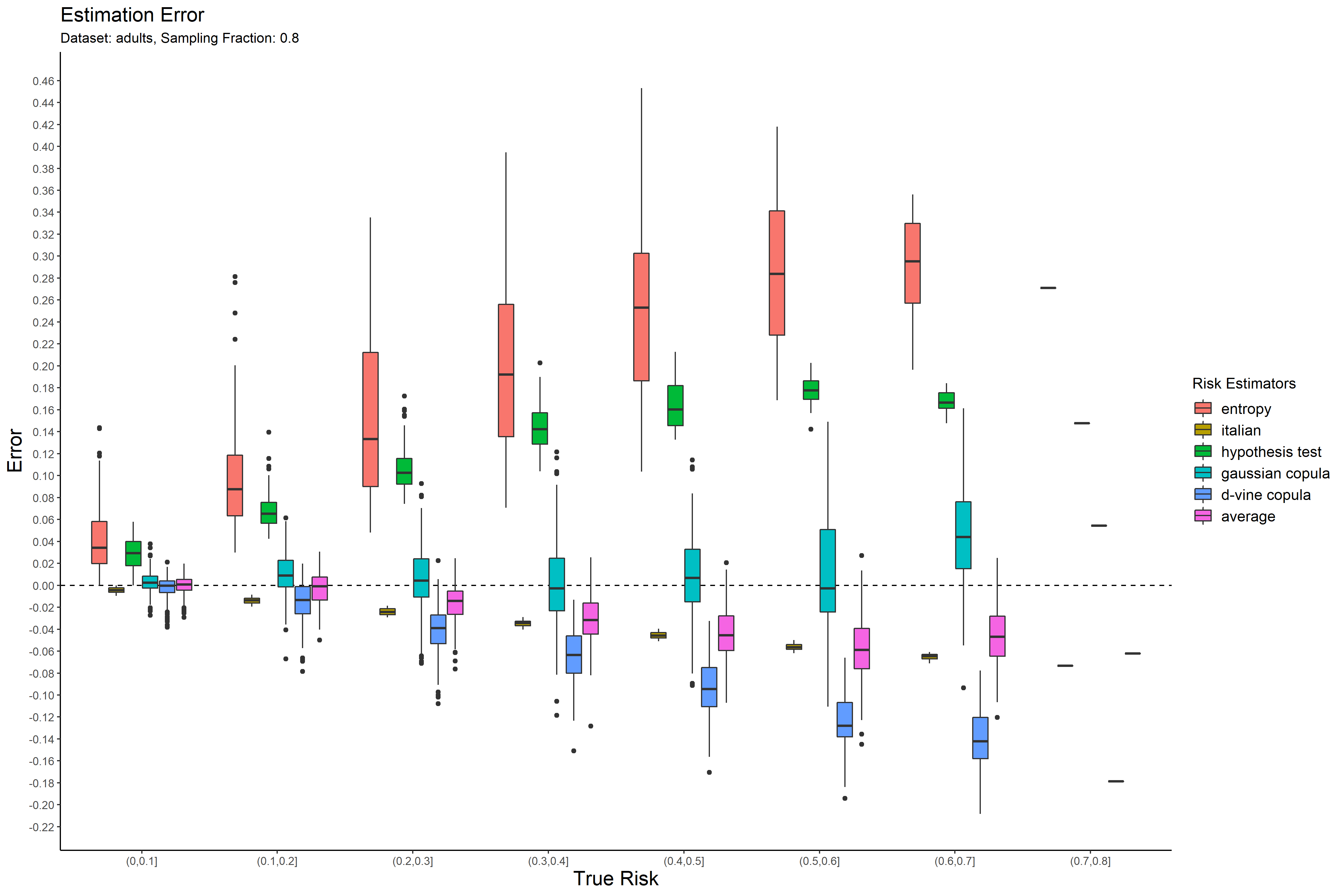

Supplement: S2 File — (ZIP) [file pone.0269097.s002.zip › adults/comparison.adults.16.png]

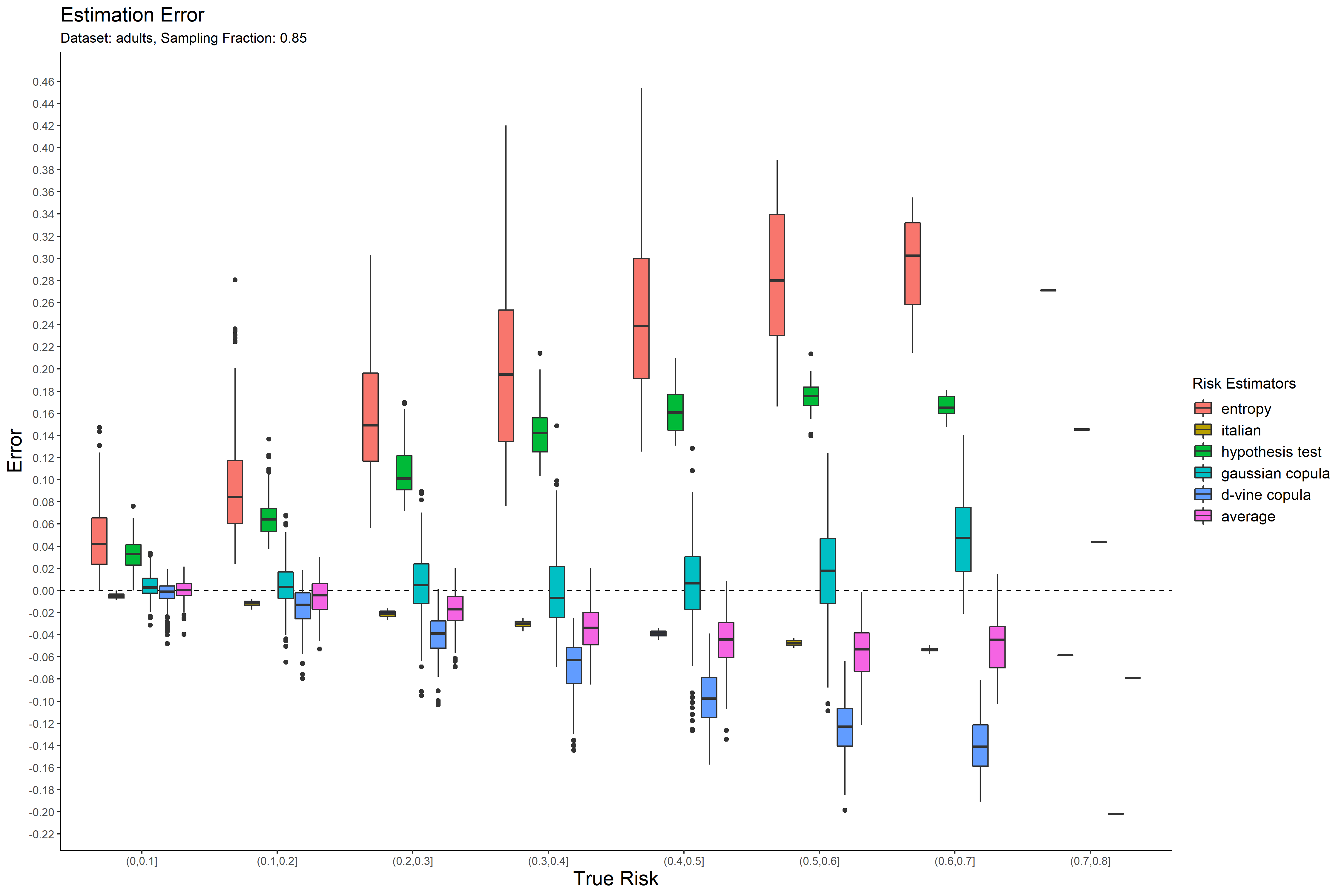

Supplement: S2 File — (ZIP) [file pone.0269097.s002.zip › adults/comparison.adults.17.png]

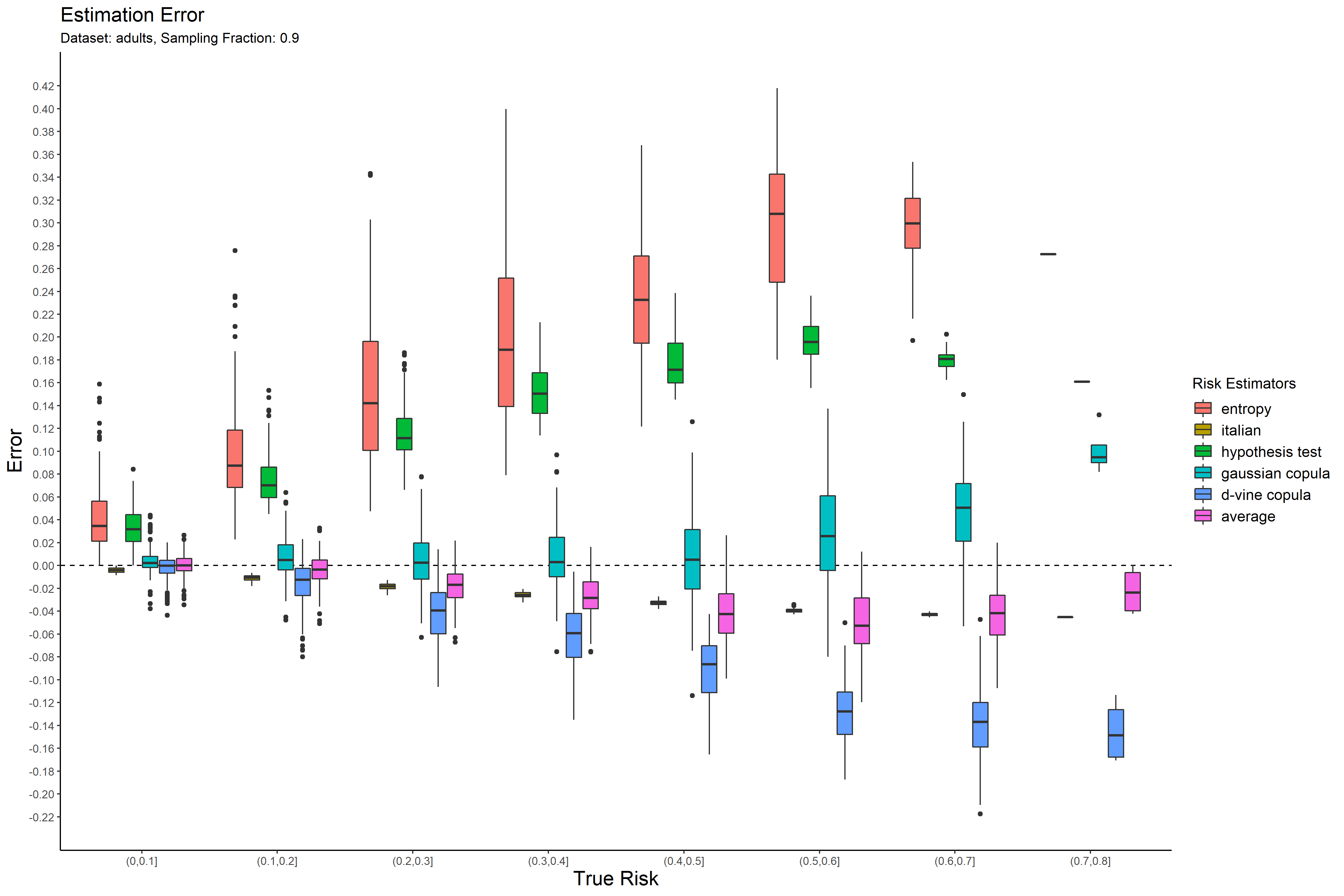

Supplement: S2 File — (ZIP) [file pone.0269097.s002.zip › adults/comparison.adults.18.png]

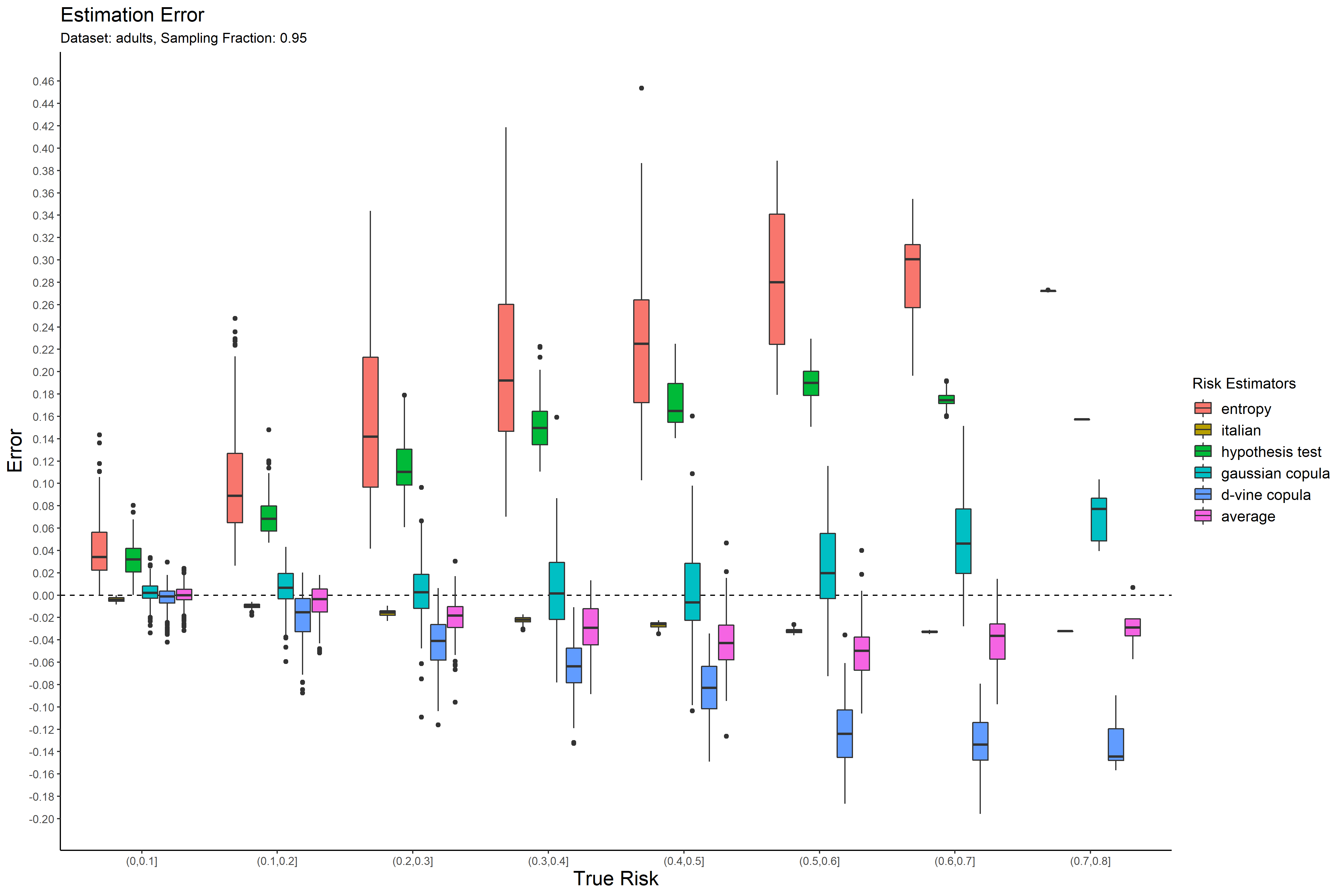

Supplement: S2 File — (ZIP) [file pone.0269097.s002.zip › adults/comparison.adults.19.png]

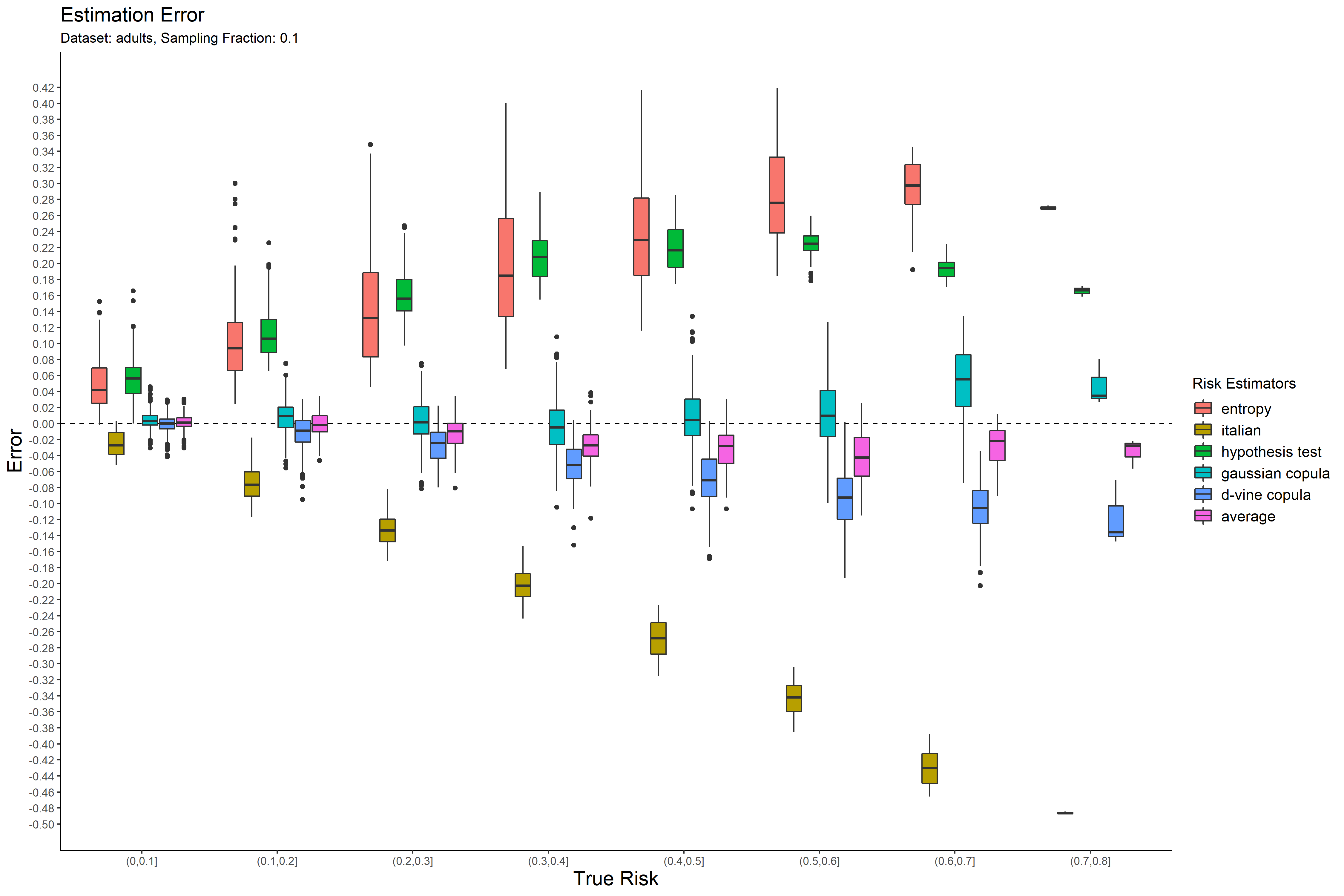

Supplement: S2 File — (ZIP) [file pone.0269097.s002.zip › adults/comparison.adults.2.png]

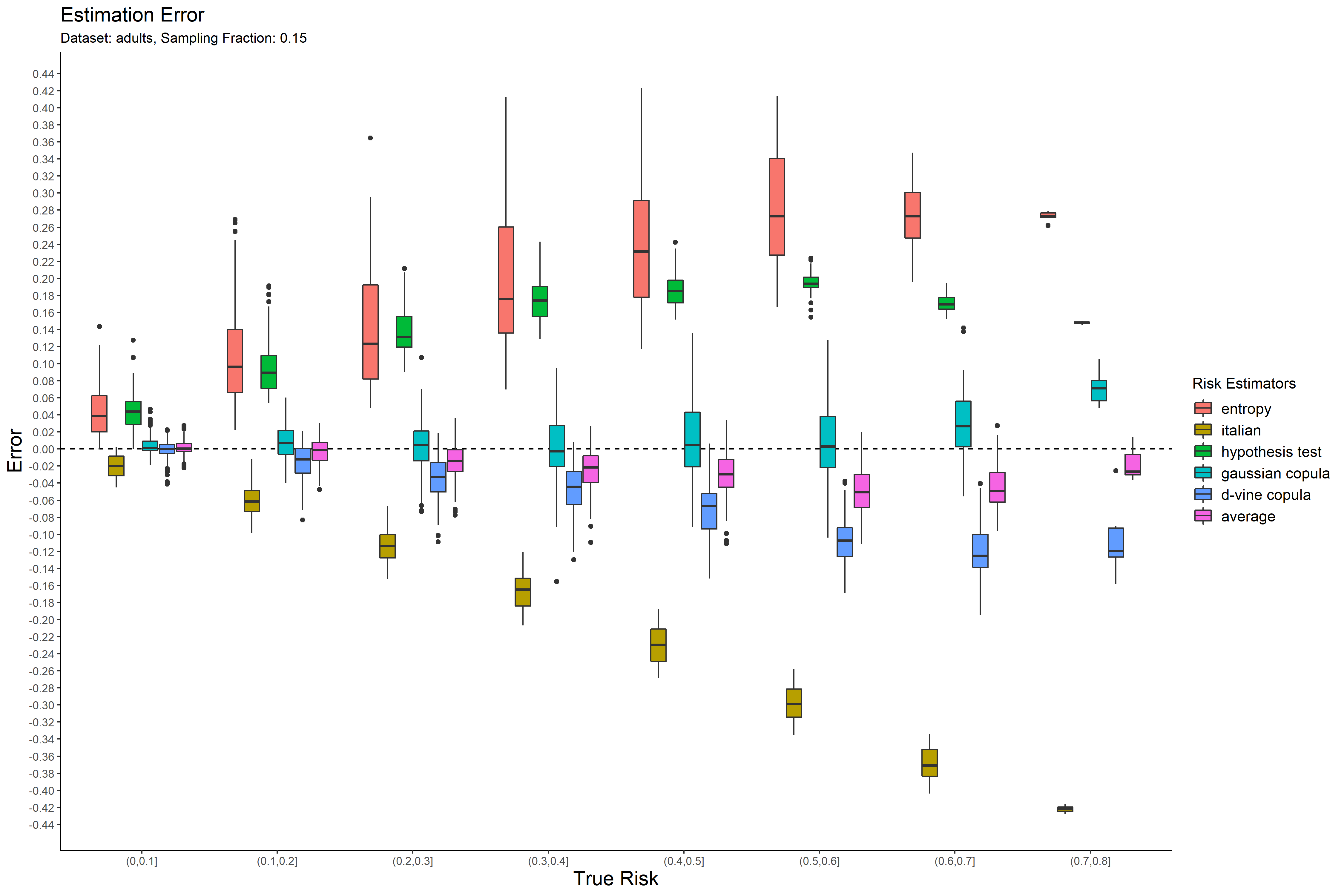

Supplement: S2 File — (ZIP) [file pone.0269097.s002.zip › adults/comparison.adults.3.png]

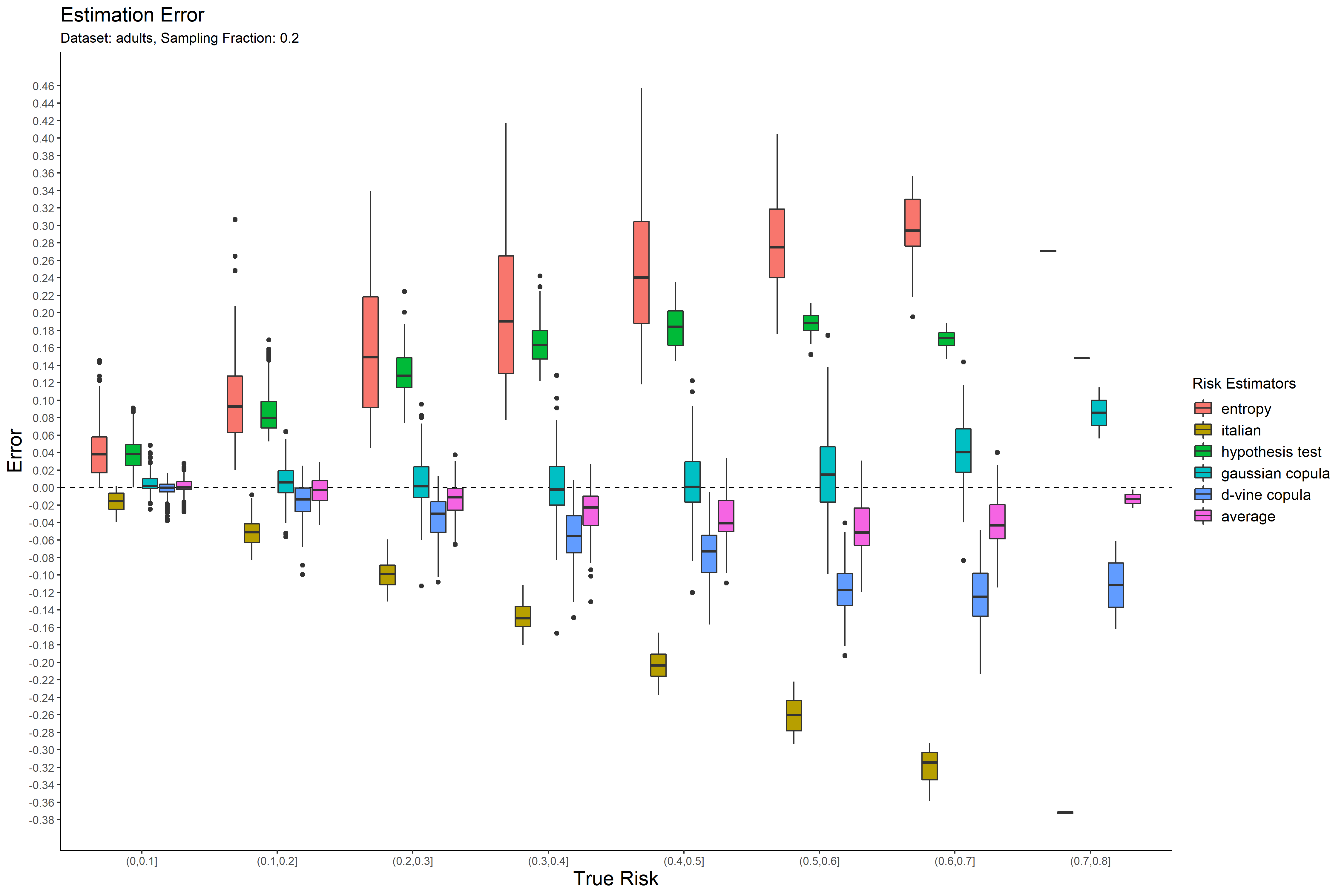

Supplement: S2 File — (ZIP) [file pone.0269097.s002.zip › adults/comparison.adults.4.png]

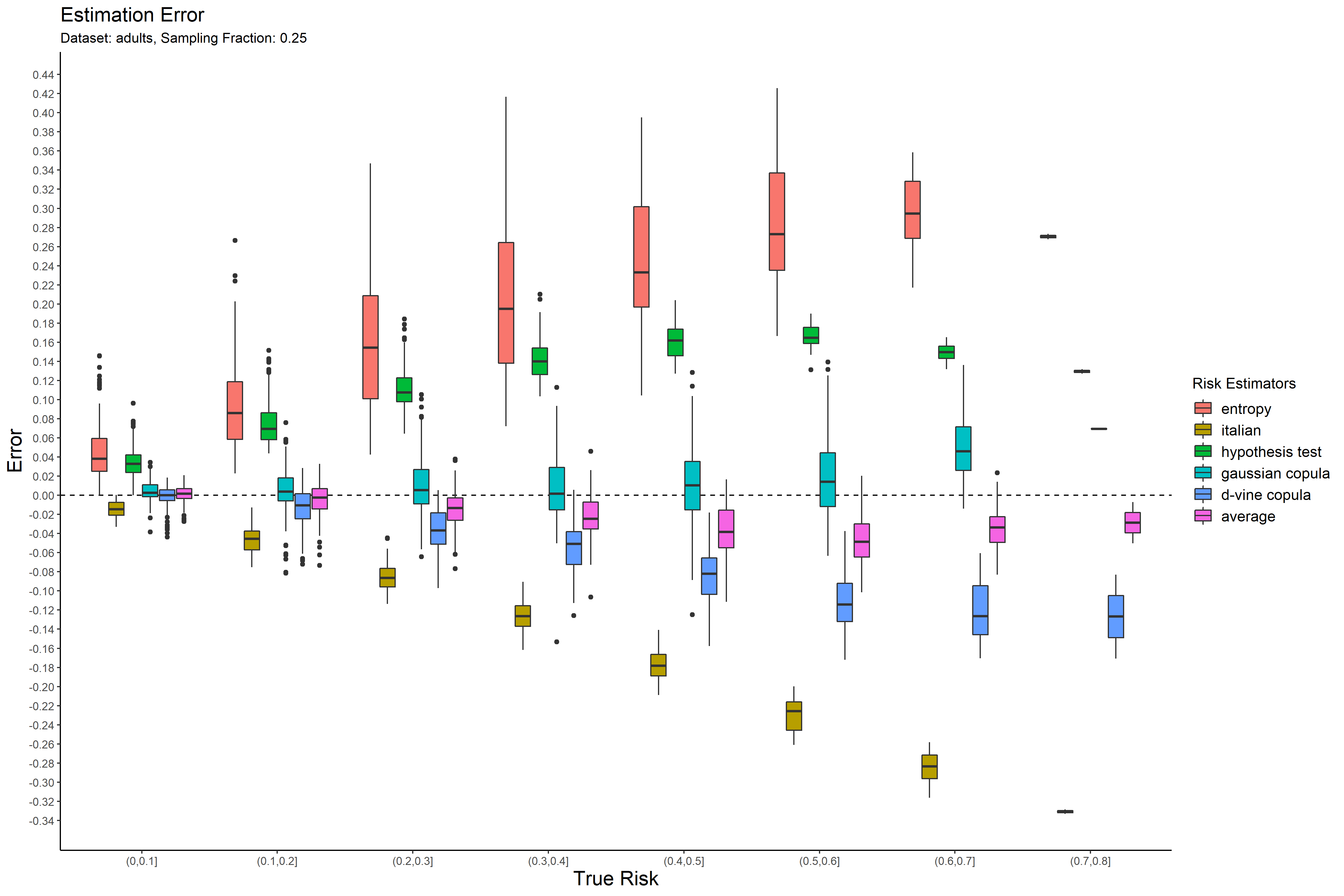

Supplement: S2 File — (ZIP) [file pone.0269097.s002.zip › adults/comparison.adults.5.png]

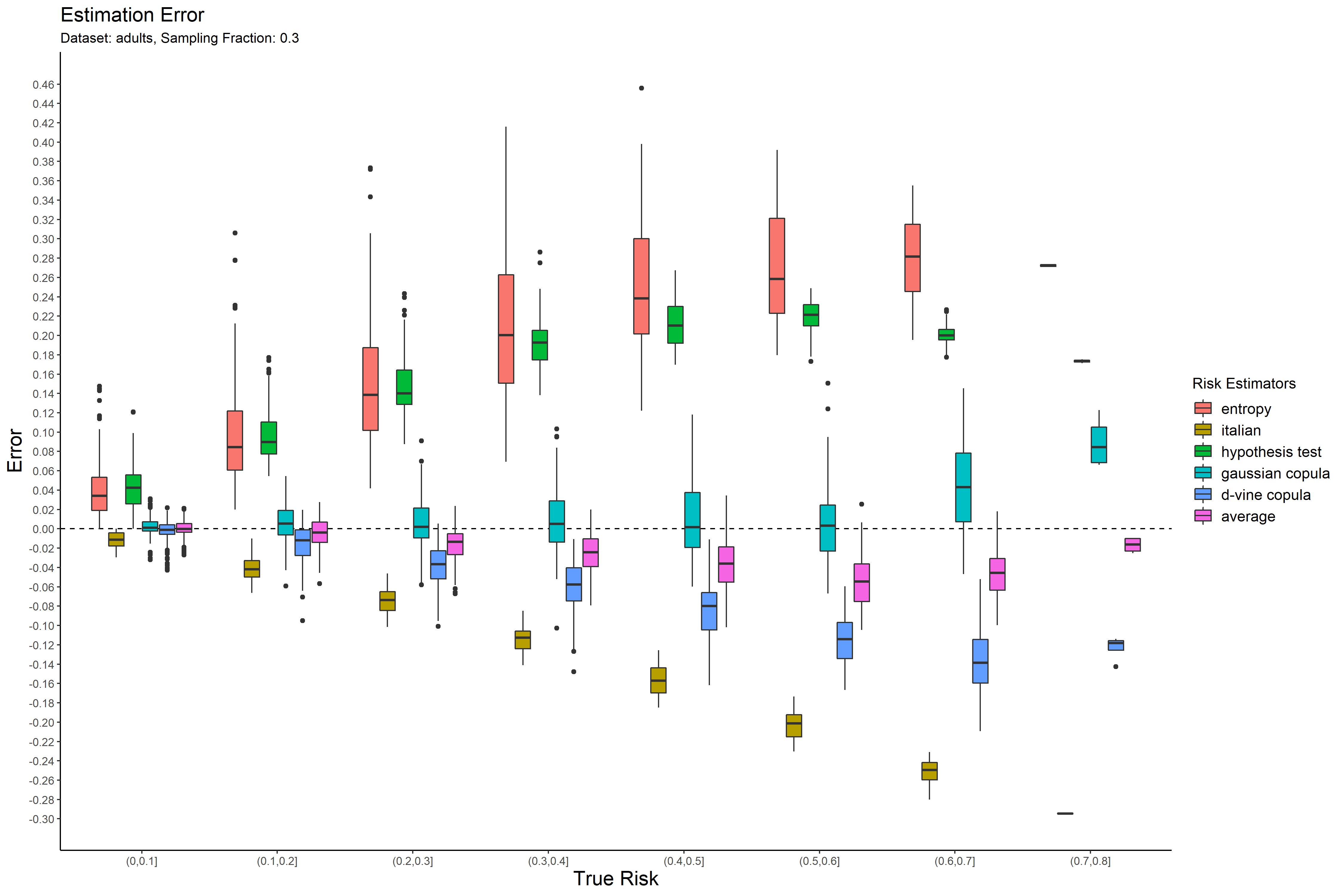

Supplement: S2 File — (ZIP) [file pone.0269097.s002.zip › adults/comparison.adults.6.png]

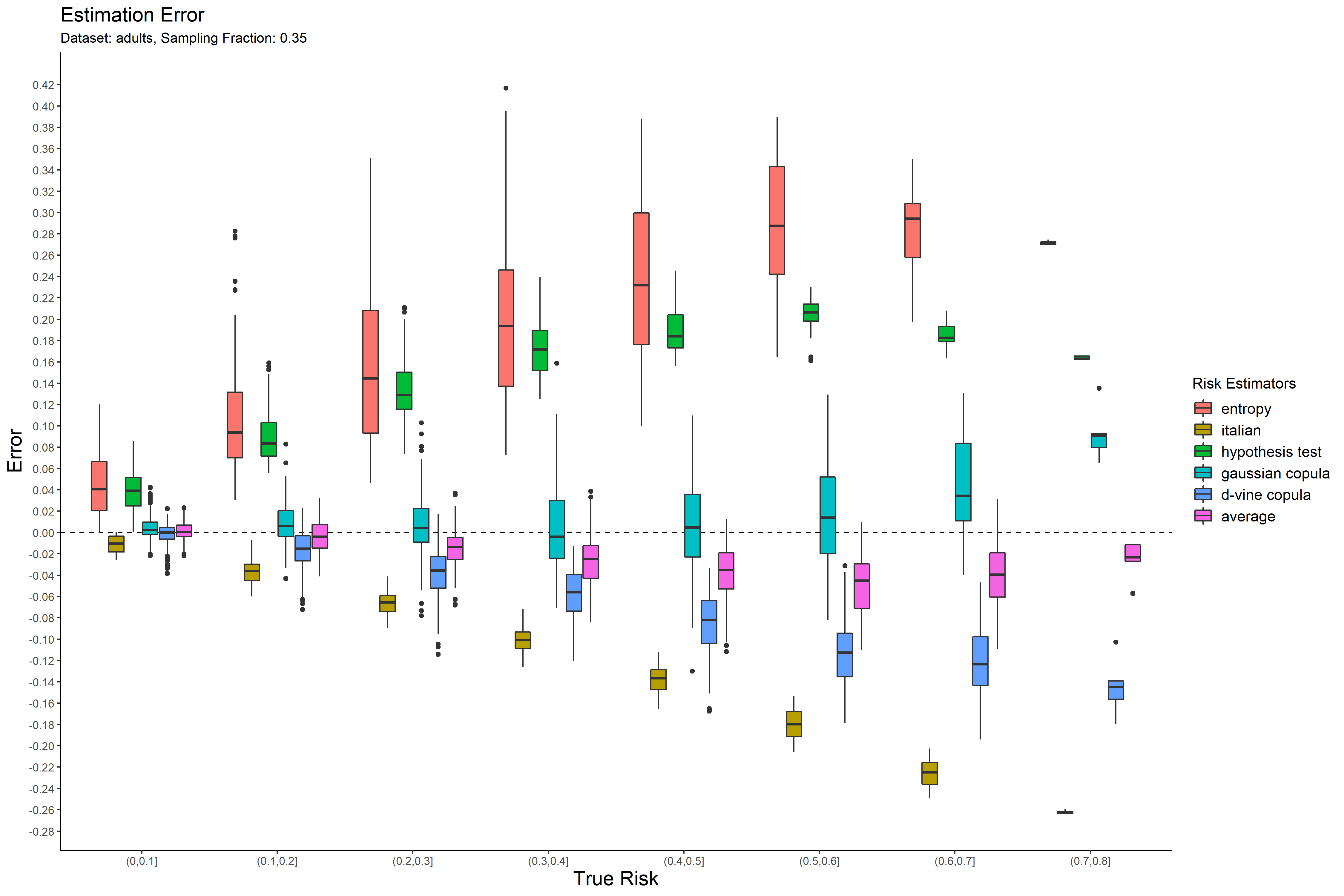

Supplement: S2 File — (ZIP) [file pone.0269097.s002.zip › adults/comparison.adults.7.png]

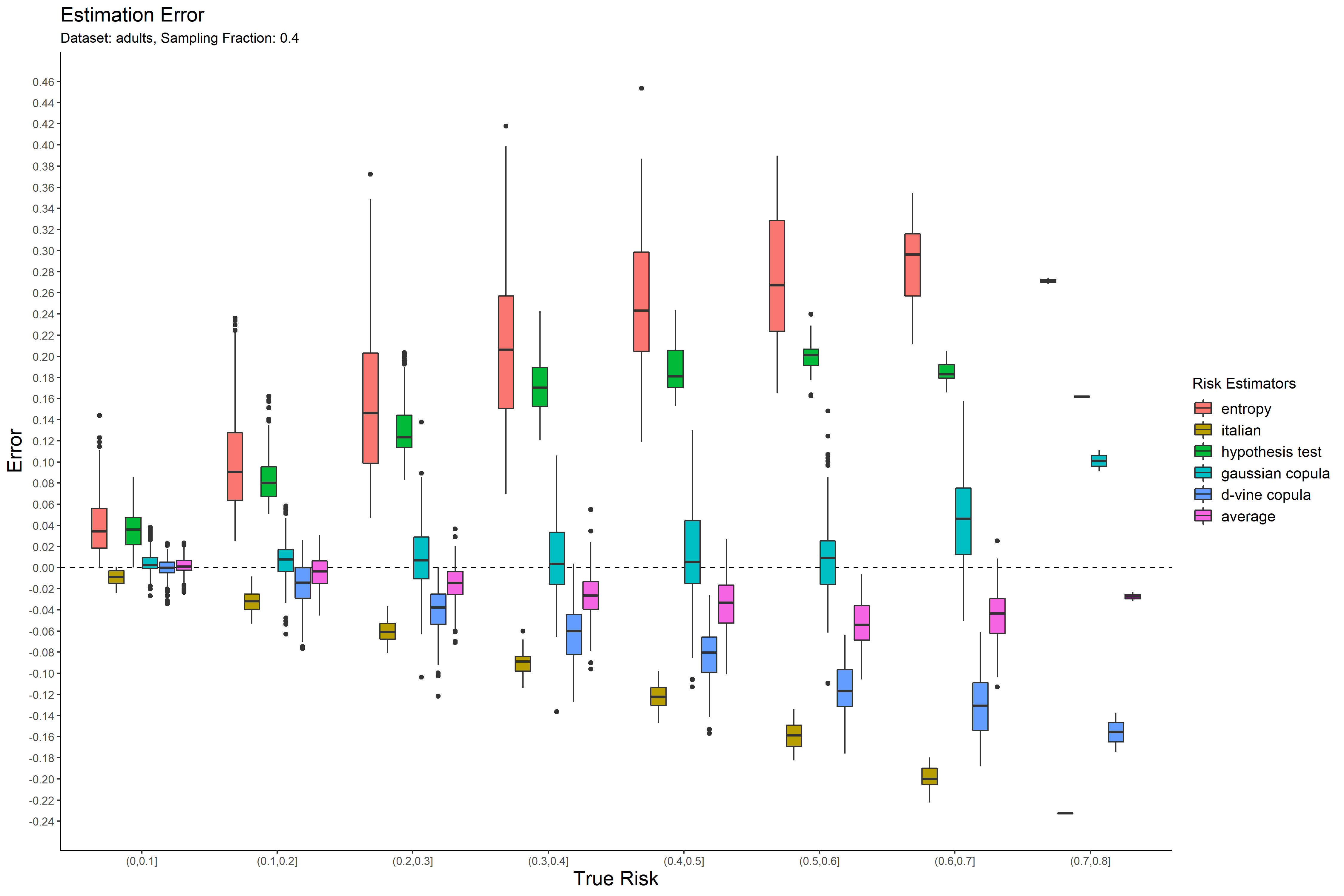

Supplement: S2 File — (ZIP) [file pone.0269097.s002.zip › adults/comparison.adults.8.png]

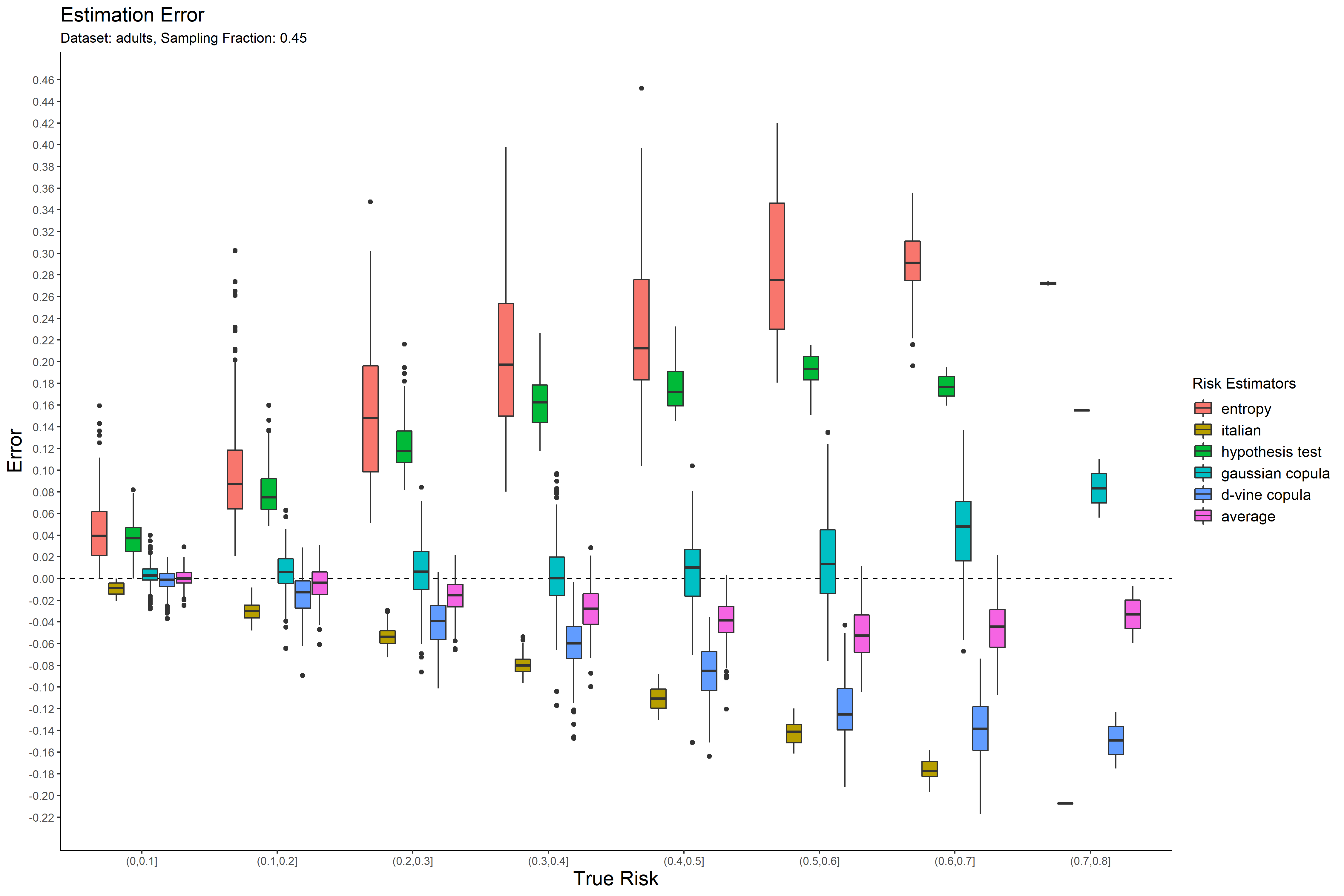

Supplement: S2 File — (ZIP) [file pone.0269097.s002.zip › adults/comparison.adults.9.png]

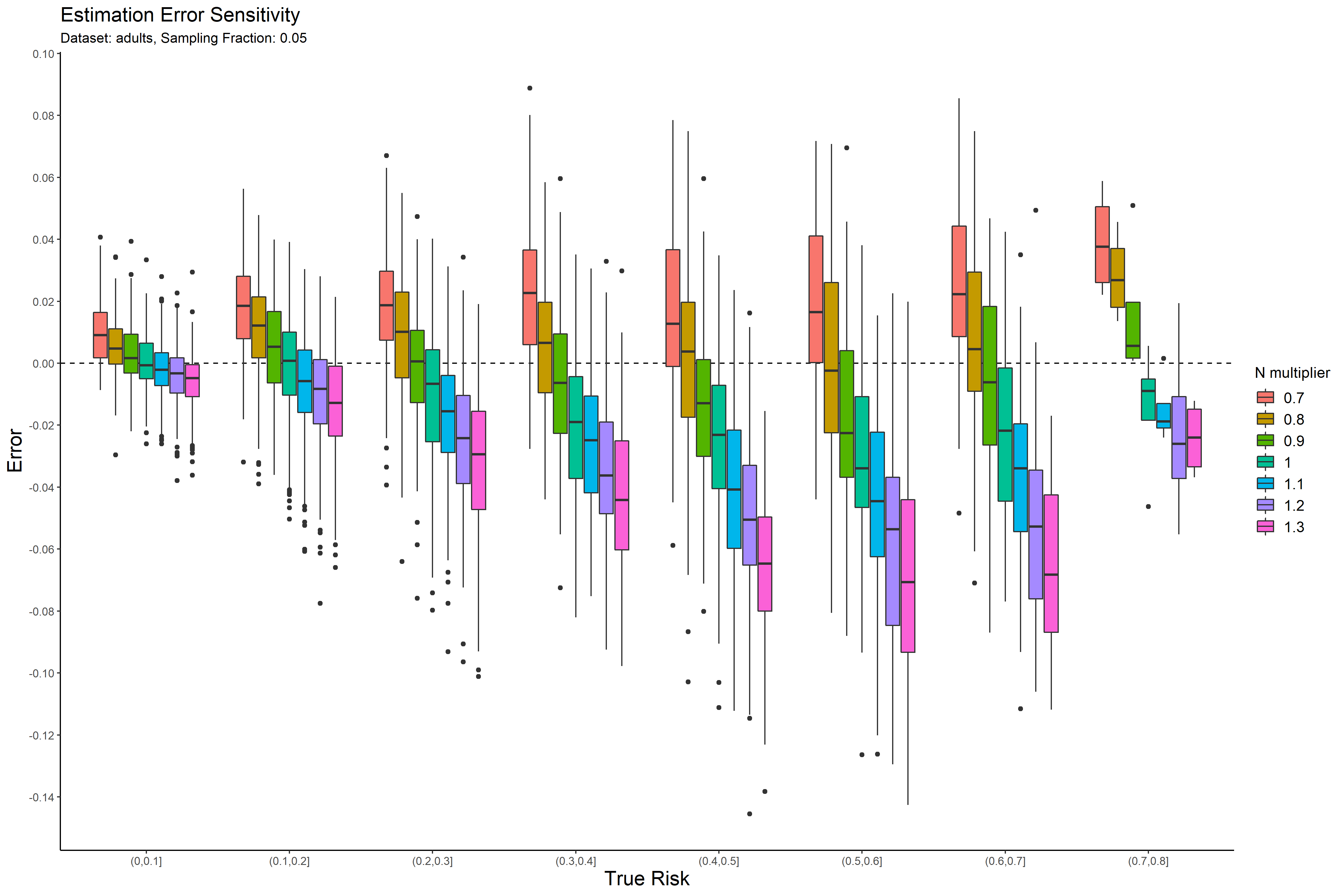

Supplement: S2 File — (ZIP) [file pone.0269097.s002.zip › adults/sensitivity.adults.1.png]

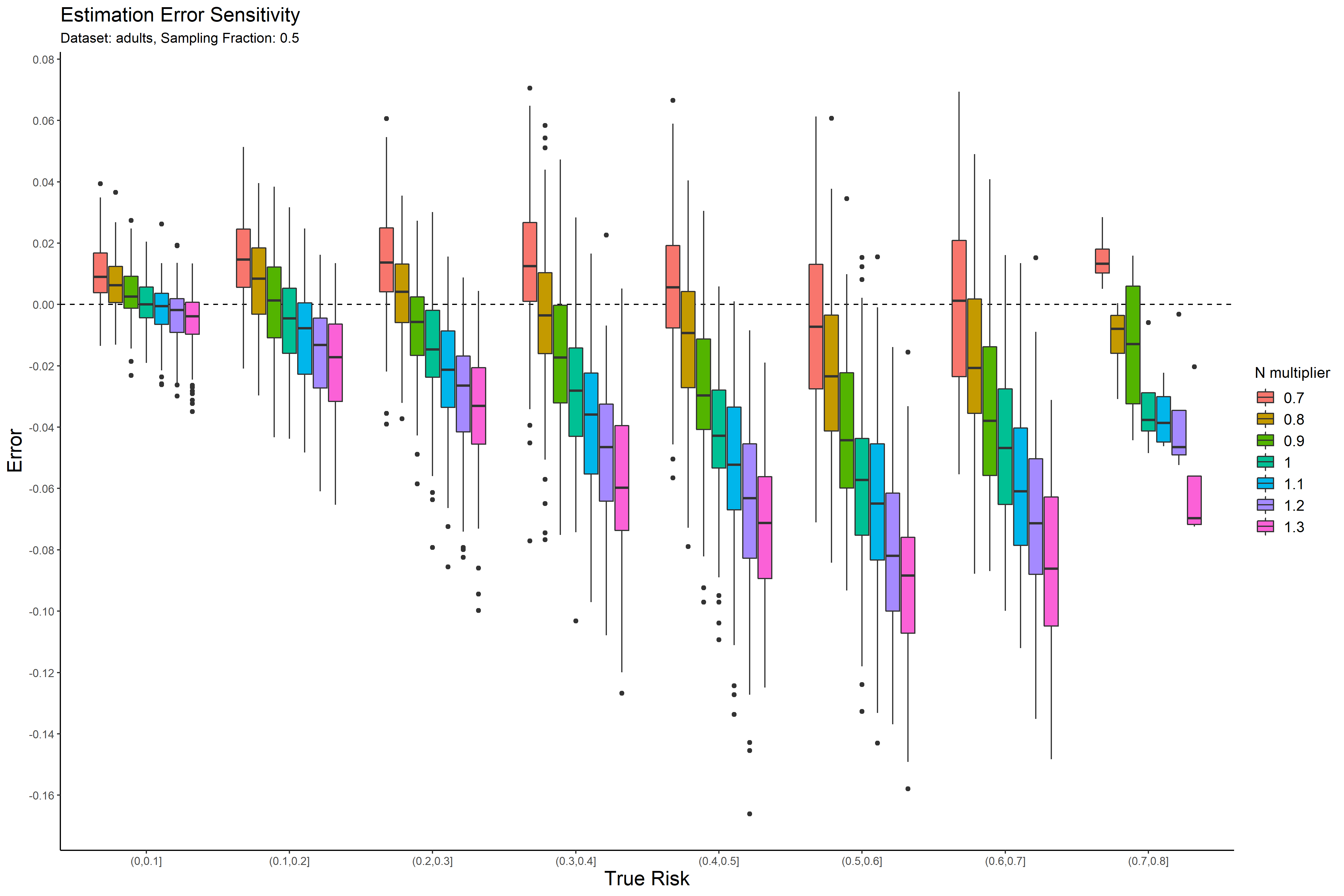

Supplement: S2 File — (ZIP) [file pone.0269097.s002.zip › adults/sensitivity.adults.10.png]

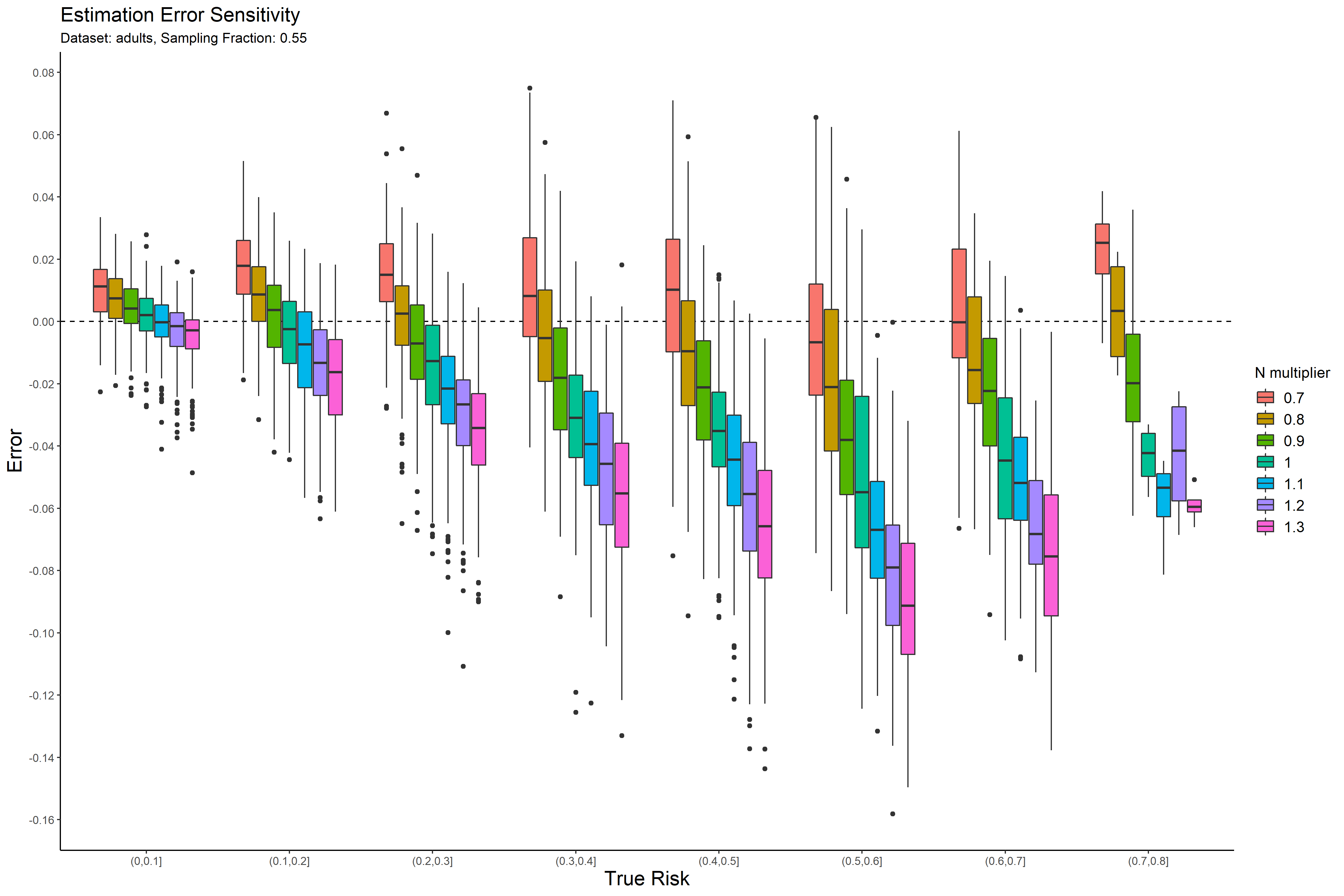

Supplement: S2 File — (ZIP) [file pone.0269097.s002.zip › adults/sensitivity.adults.11.png]

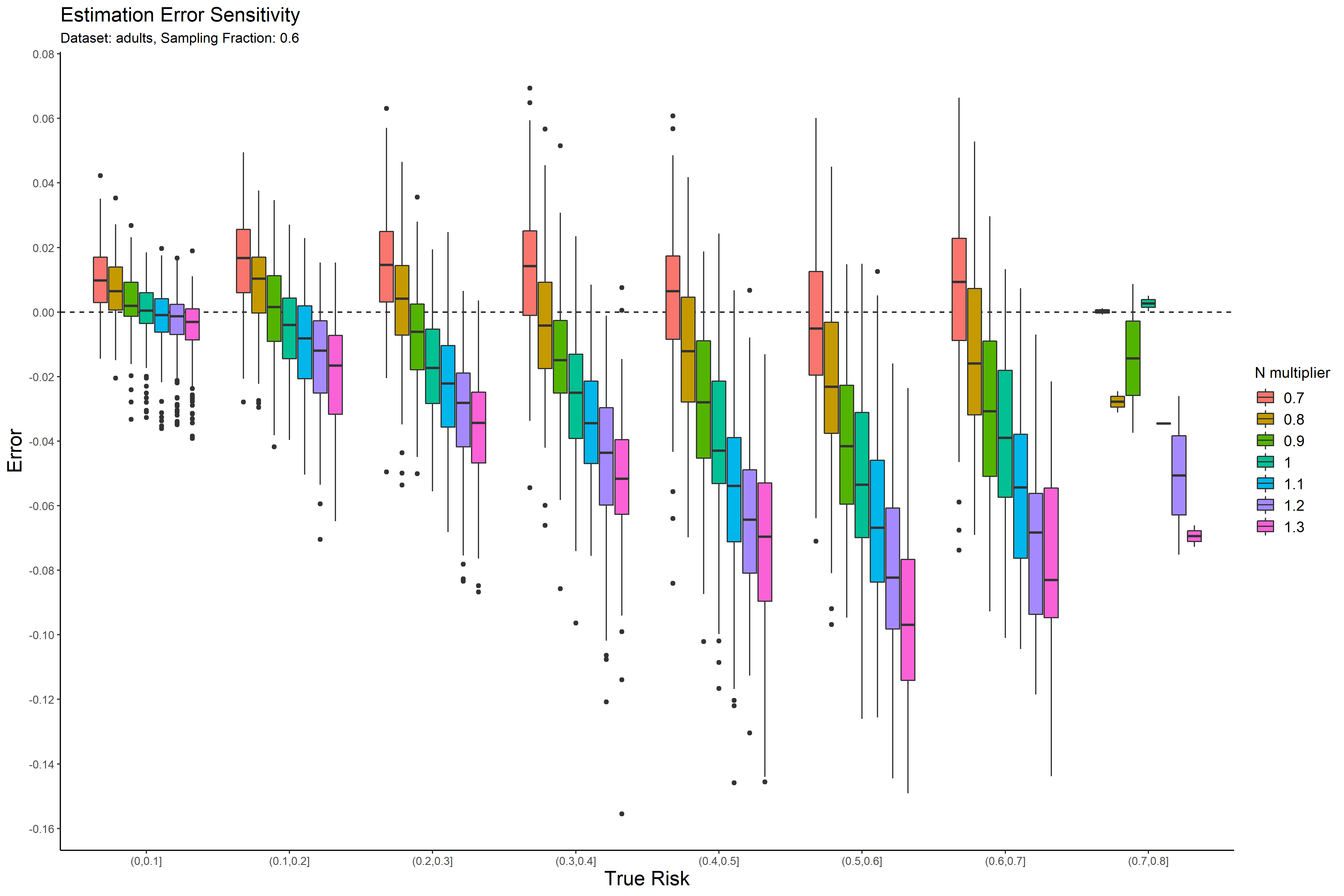

Supplement: S2 File — (ZIP) [file pone.0269097.s002.zip › adults/sensitivity.adults.12.png]

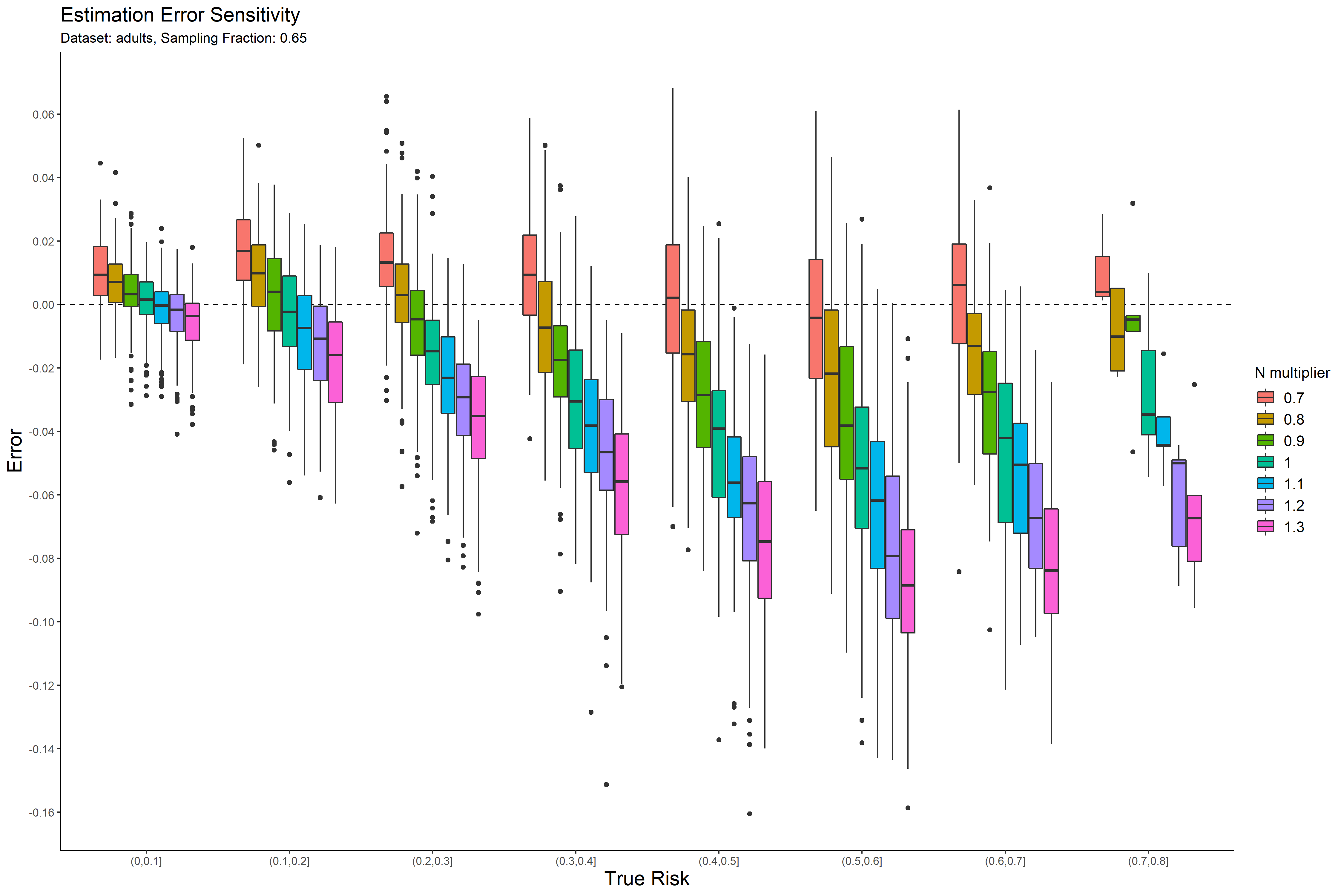

Supplement: S2 File — (ZIP) [file pone.0269097.s002.zip › adults/sensitivity.adults.13.png]

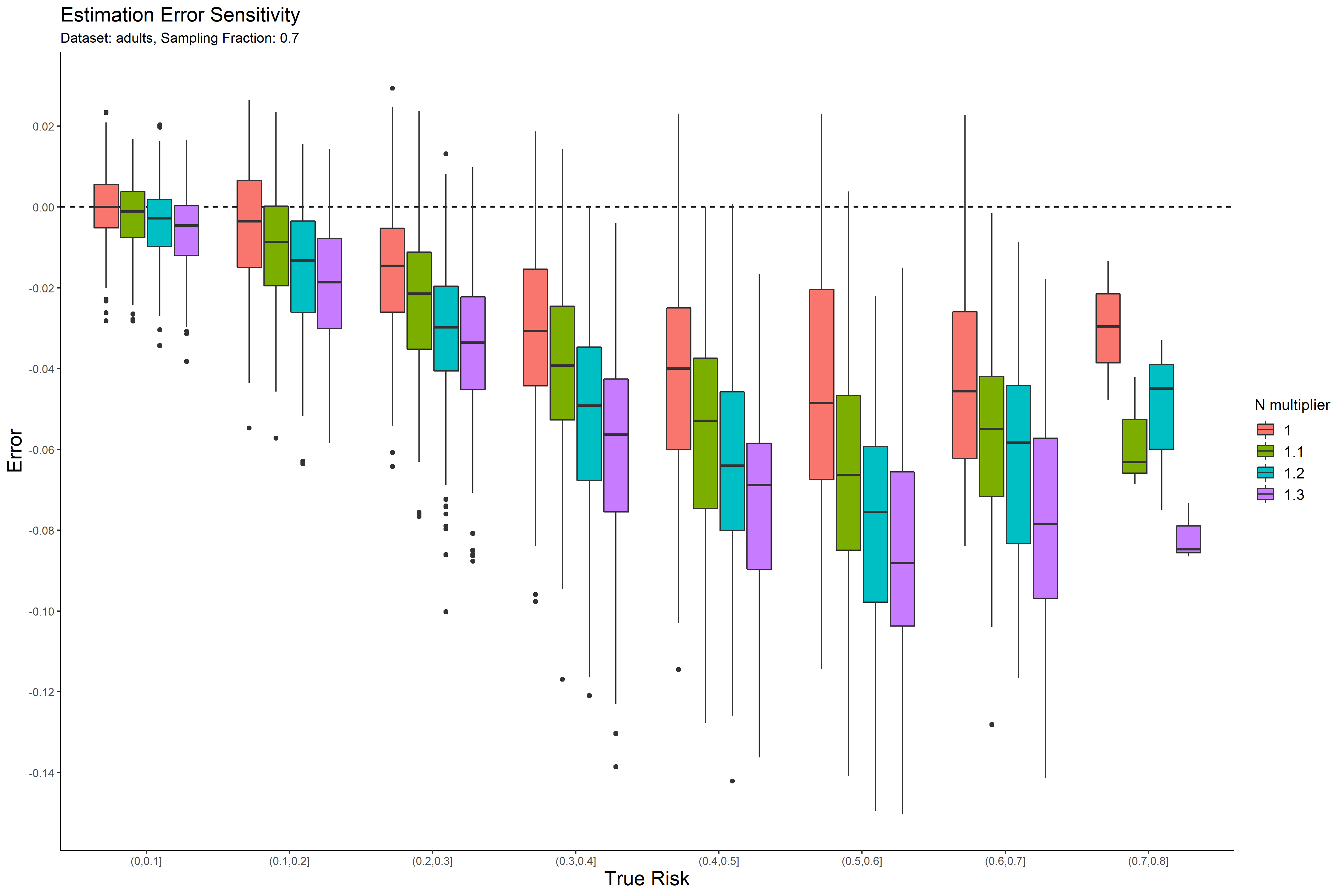

Supplement: S2 File — (ZIP) [file pone.0269097.s002.zip › adults/sensitivity.adults.14.png]

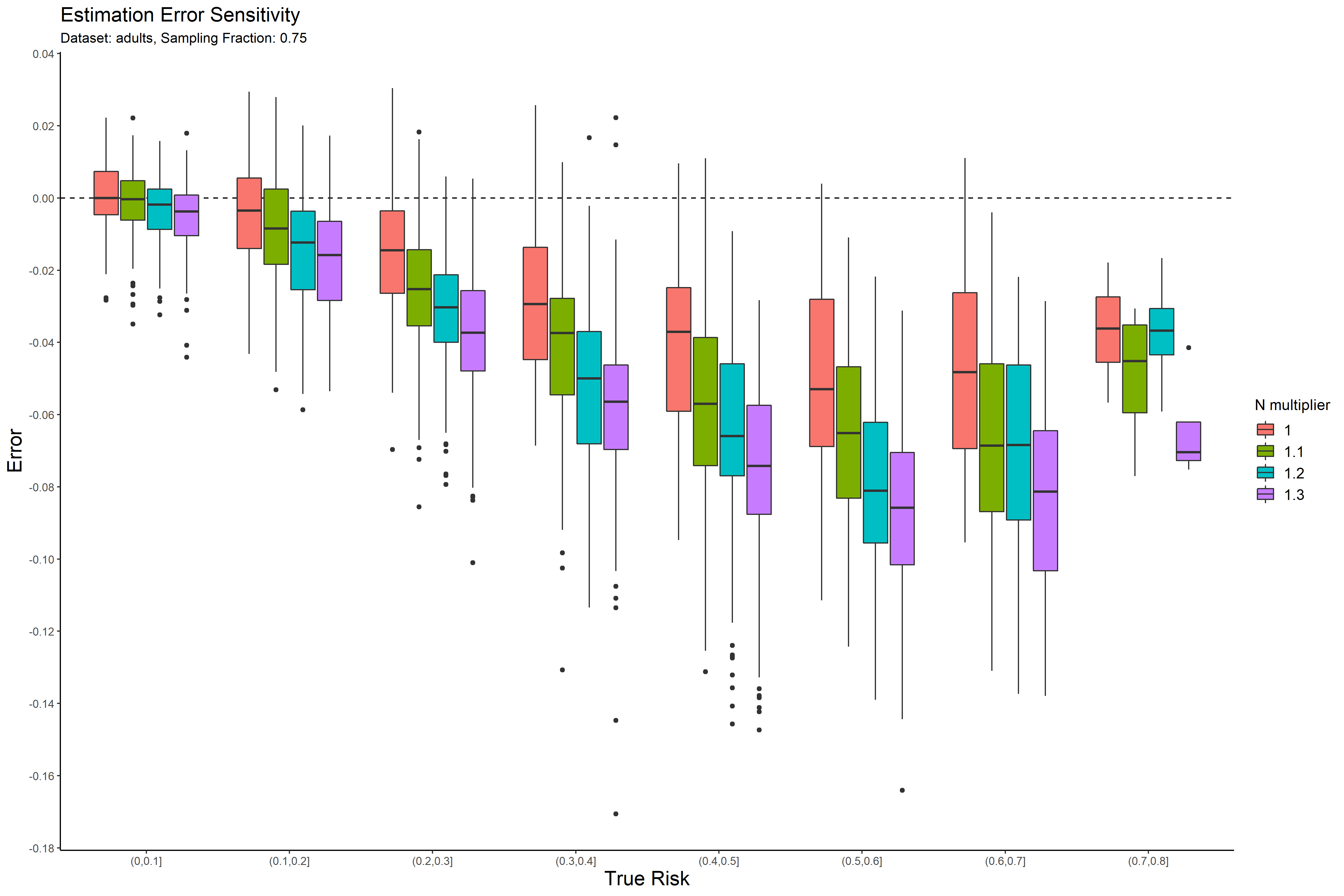

Supplement: S2 File — (ZIP) [file pone.0269097.s002.zip › adults/sensitivity.adults.15.png]

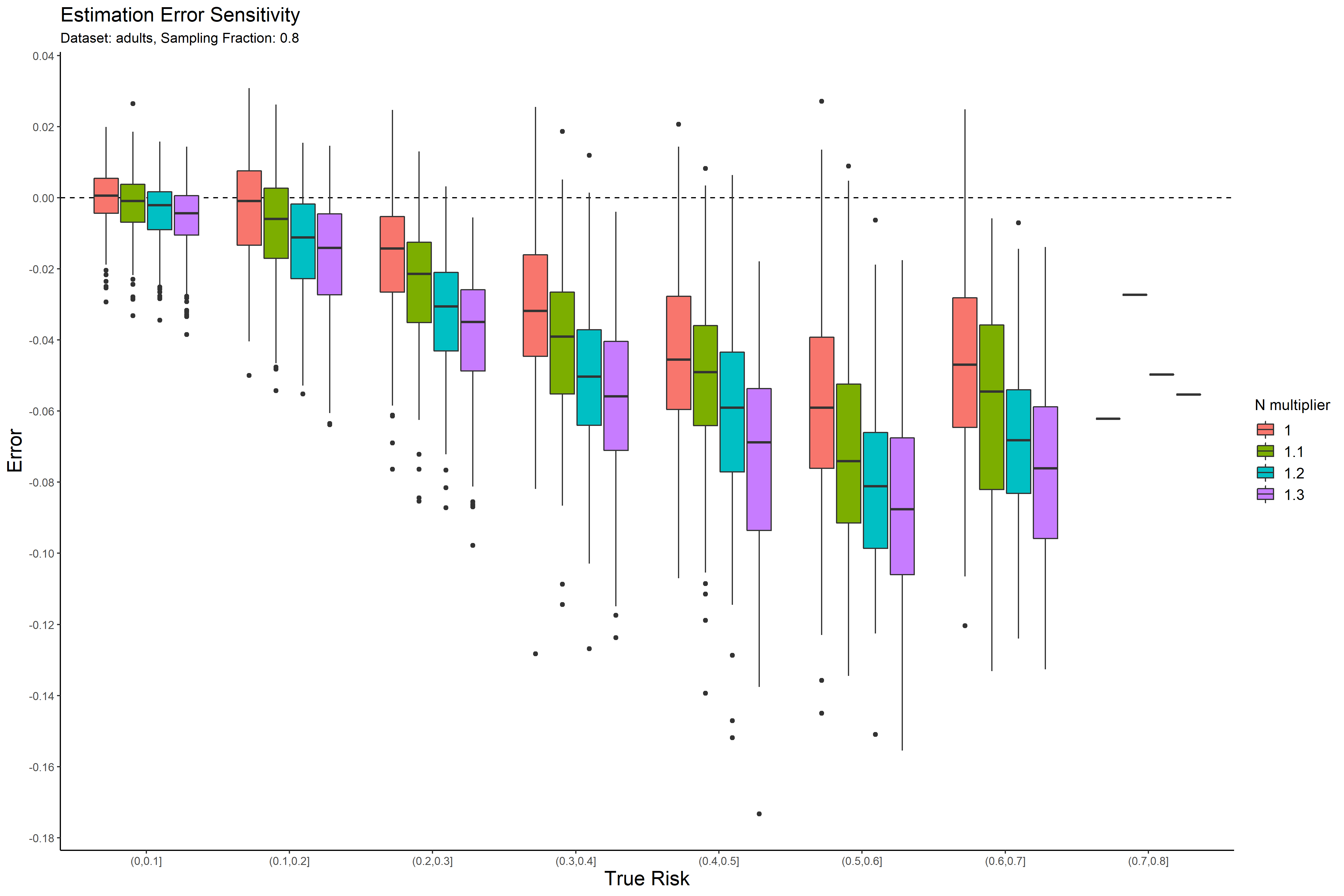

Supplement: S2 File — (ZIP) [file pone.0269097.s002.zip › adults/sensitivity.adults.16.png]

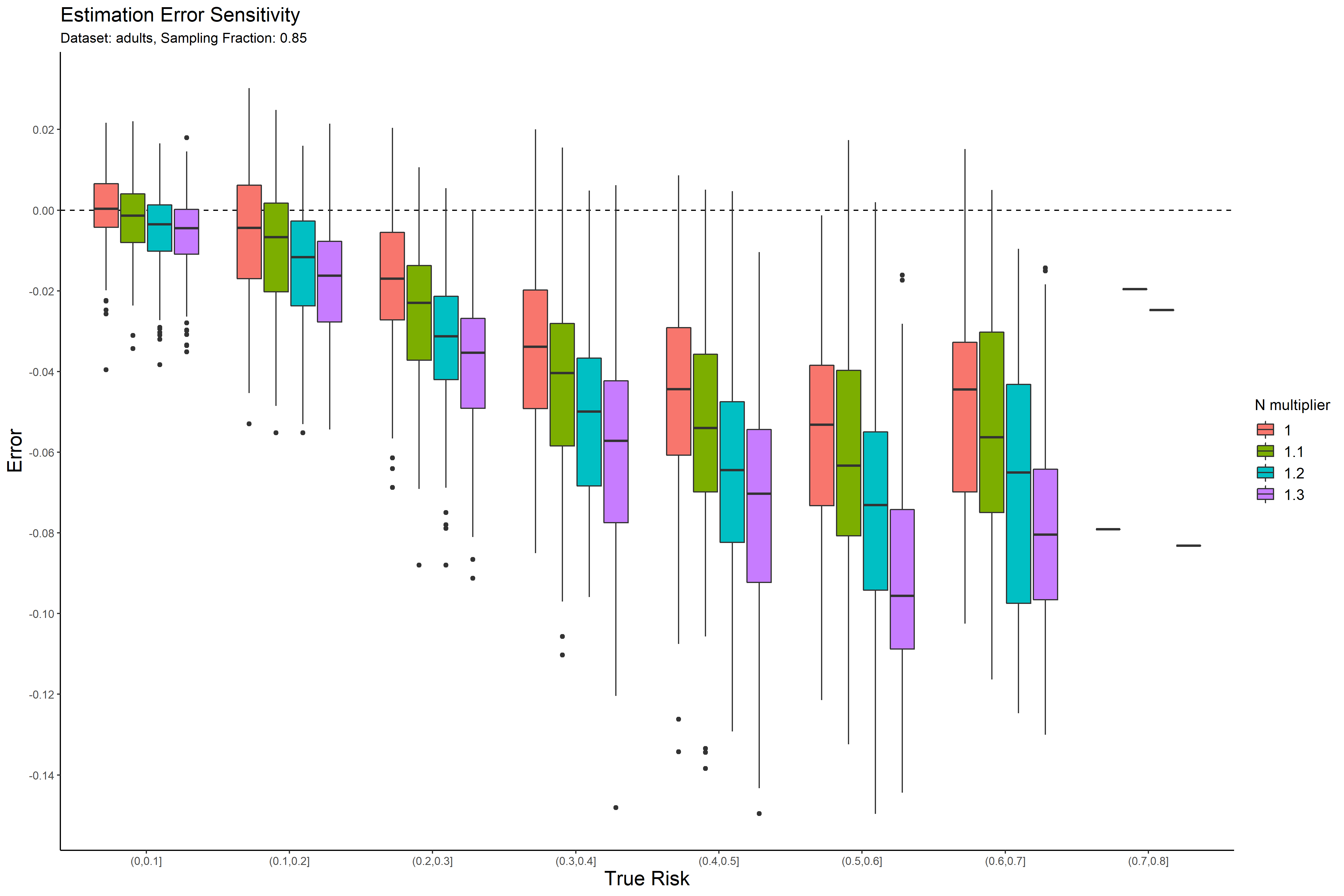

Supplement: S2 File — (ZIP) [file pone.0269097.s002.zip › adults/sensitivity.adults.17.png]

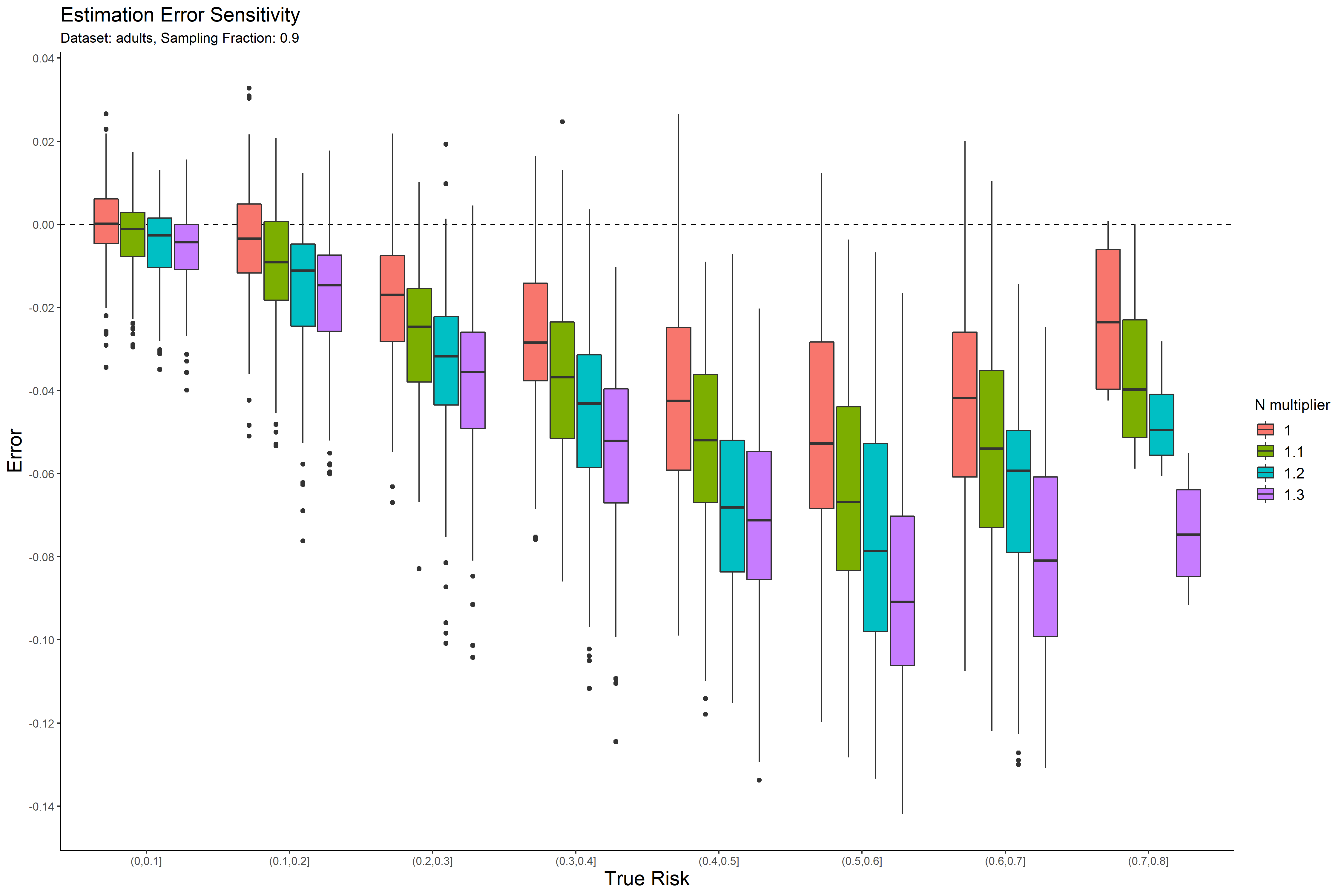

Supplement: S2 File — (ZIP) [file pone.0269097.s002.zip › adults/sensitivity.adults.18.png]

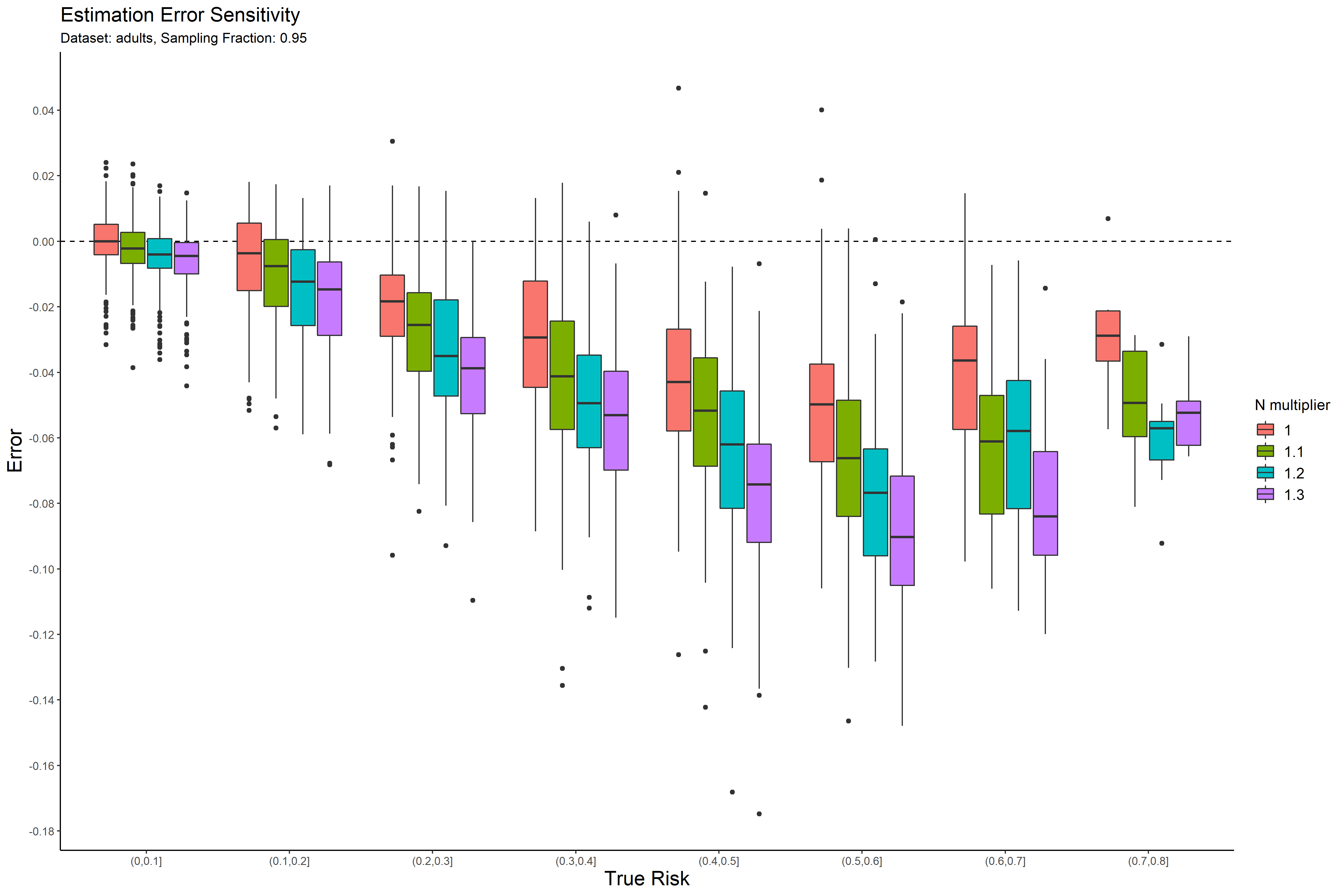

Supplement: S2 File — (ZIP) [file pone.0269097.s002.zip › adults/sensitivity.adults.19.png]

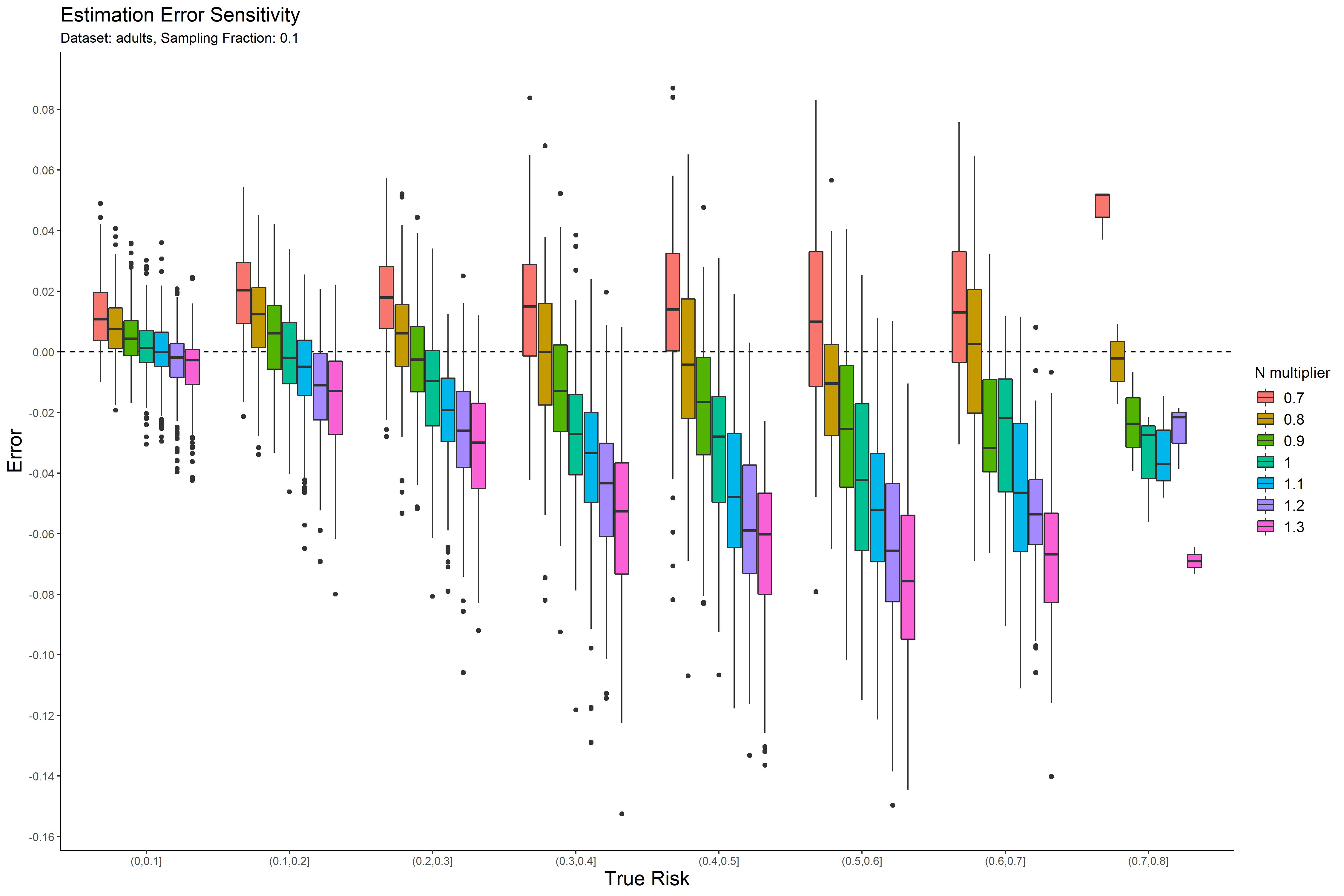

Supplement: S2 File — (ZIP) [file pone.0269097.s002.zip › adults/sensitivity.adults.2.png]

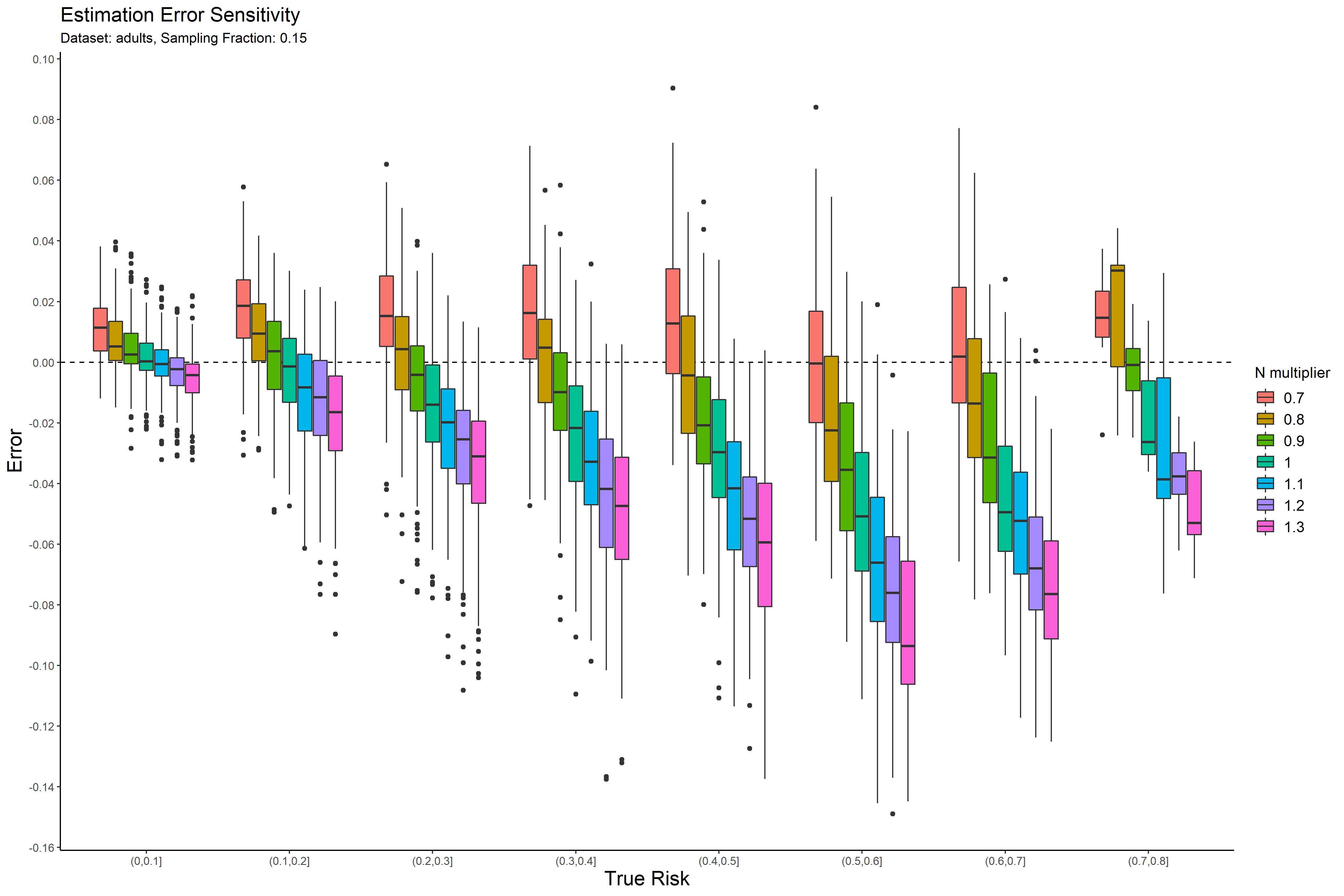

Supplement: S2 File — (ZIP) [file pone.0269097.s002.zip › adults/sensitivity.adults.3.png]

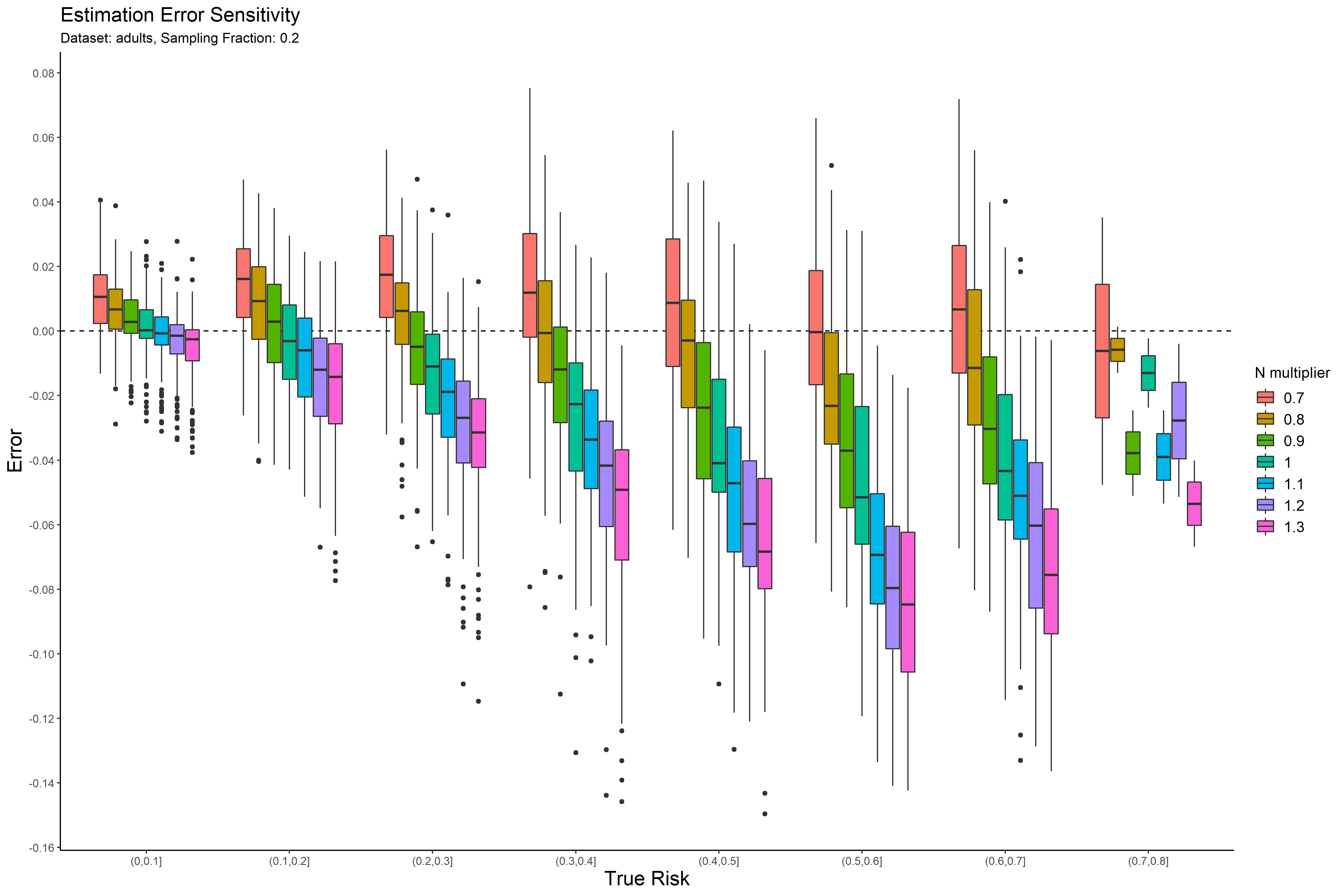

Supplement: S2 File — (ZIP) [file pone.0269097.s002.zip › adults/sensitivity.adults.4.png]

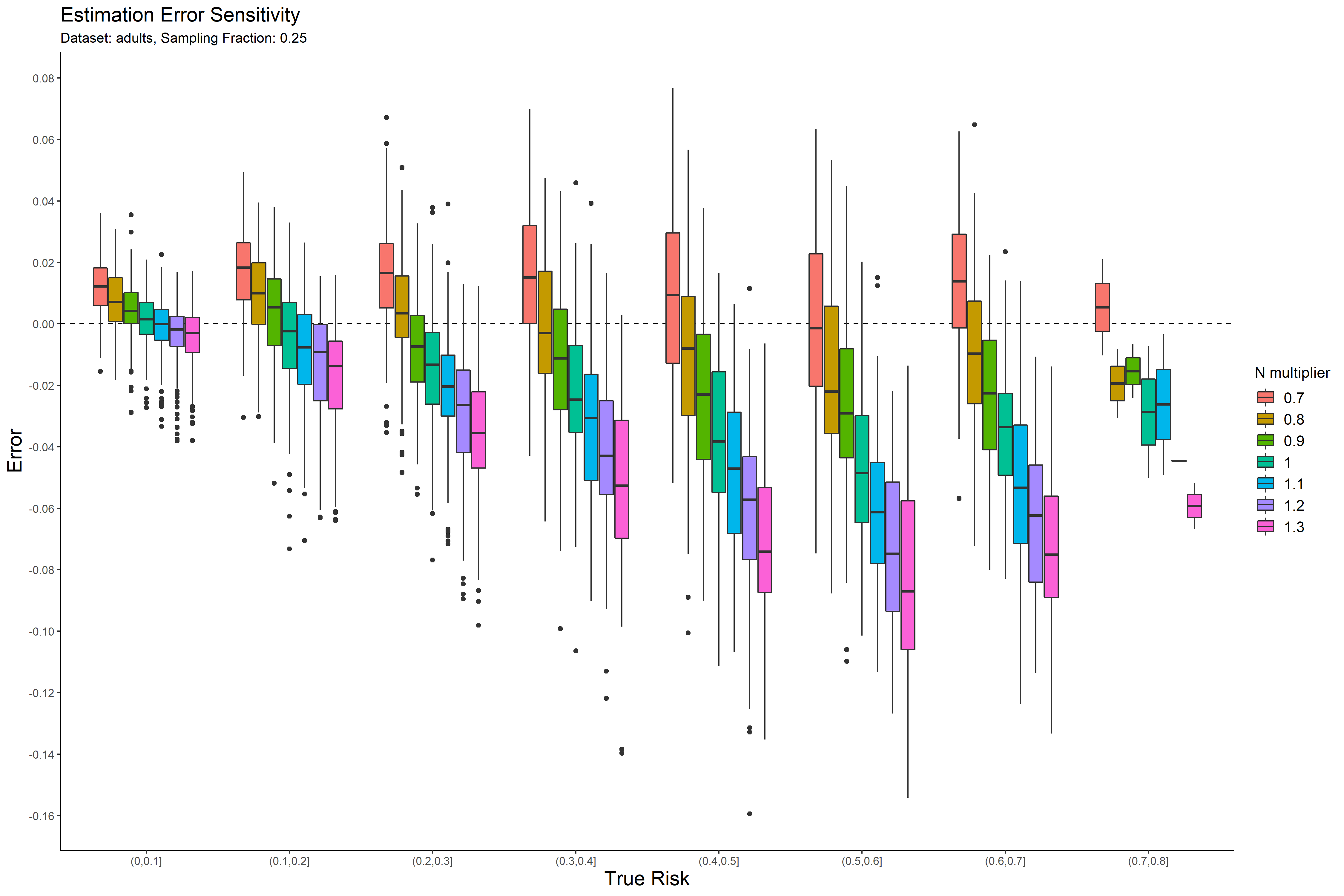

Supplement: S2 File — (ZIP) [file pone.0269097.s002.zip › adults/sensitivity.adults.5.png]

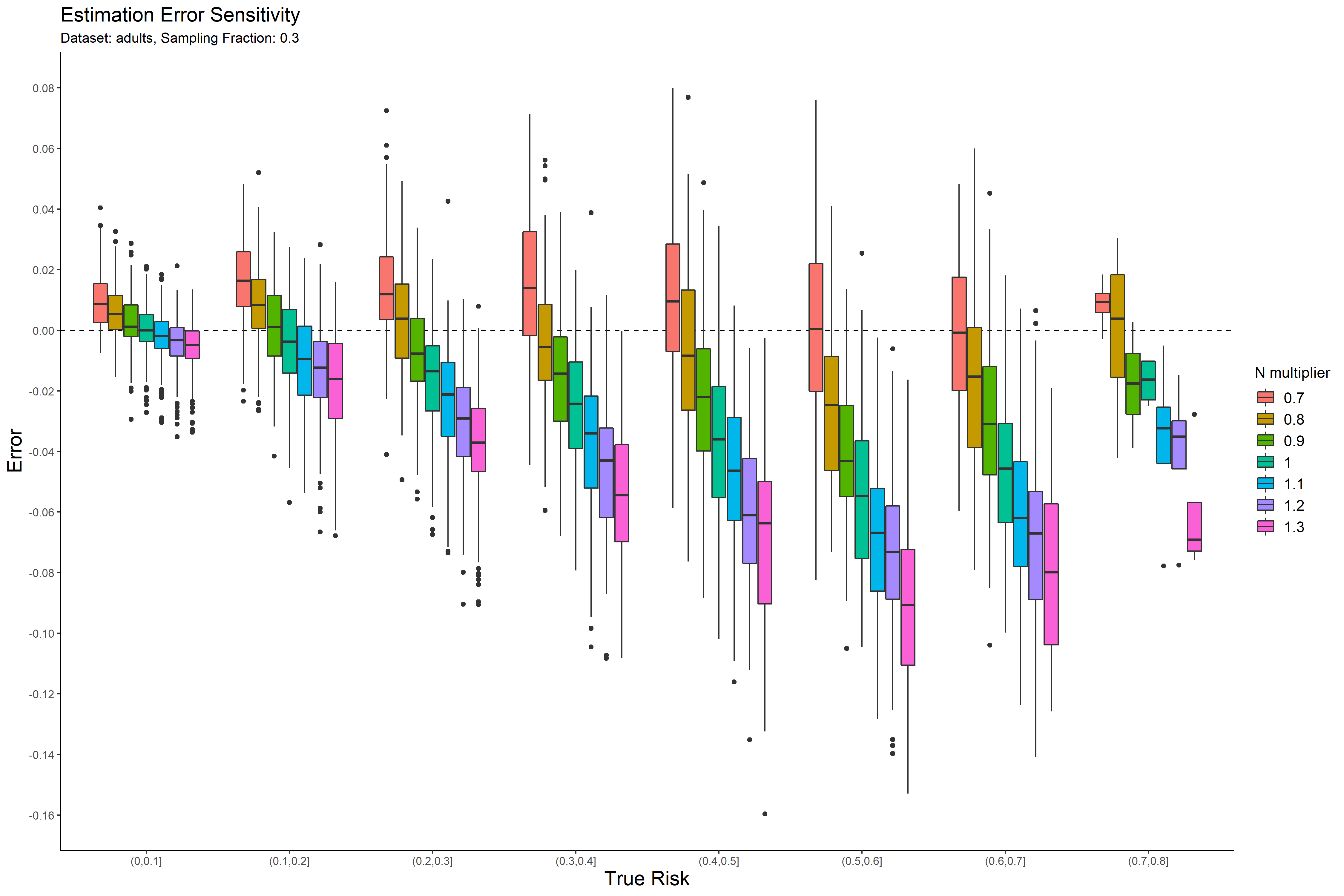

Supplement: S2 File — (ZIP) [file pone.0269097.s002.zip › adults/sensitivity.adults.6.png]

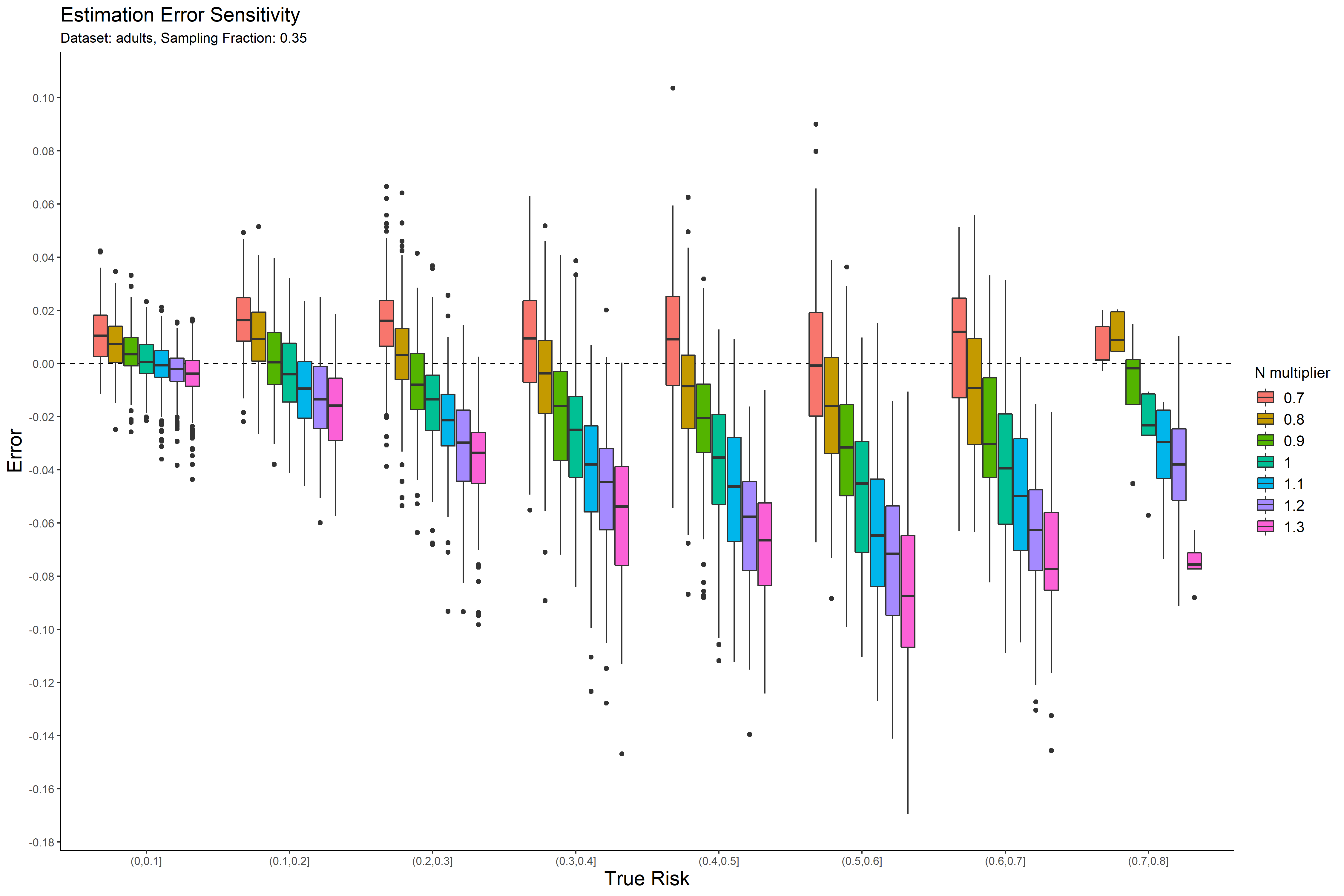

Supplement: S2 File — (ZIP) [file pone.0269097.s002.zip › adults/sensitivity.adults.7.png]

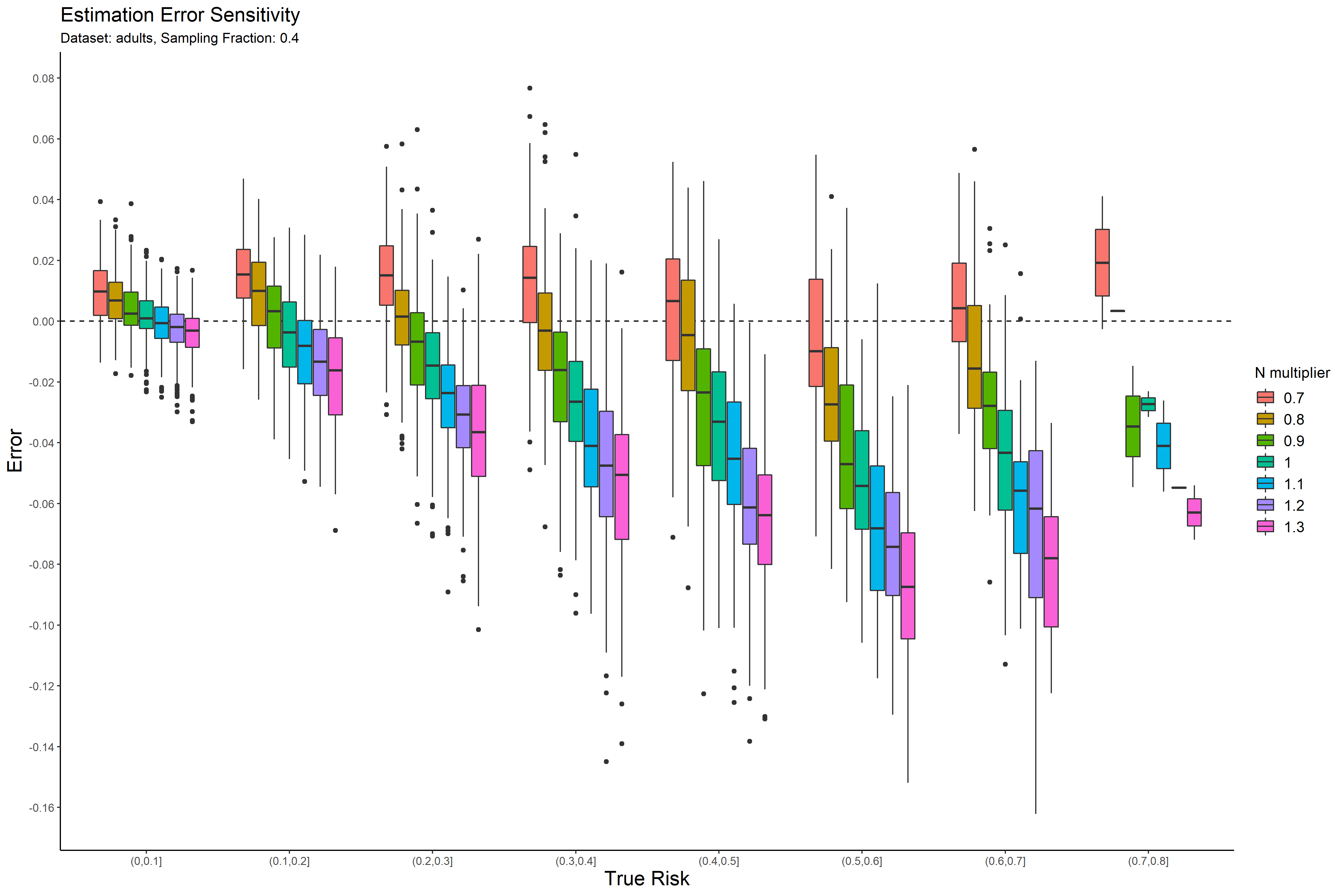

Supplement: S2 File — (ZIP) [file pone.0269097.s002.zip › adults/sensitivity.adults.8.png]

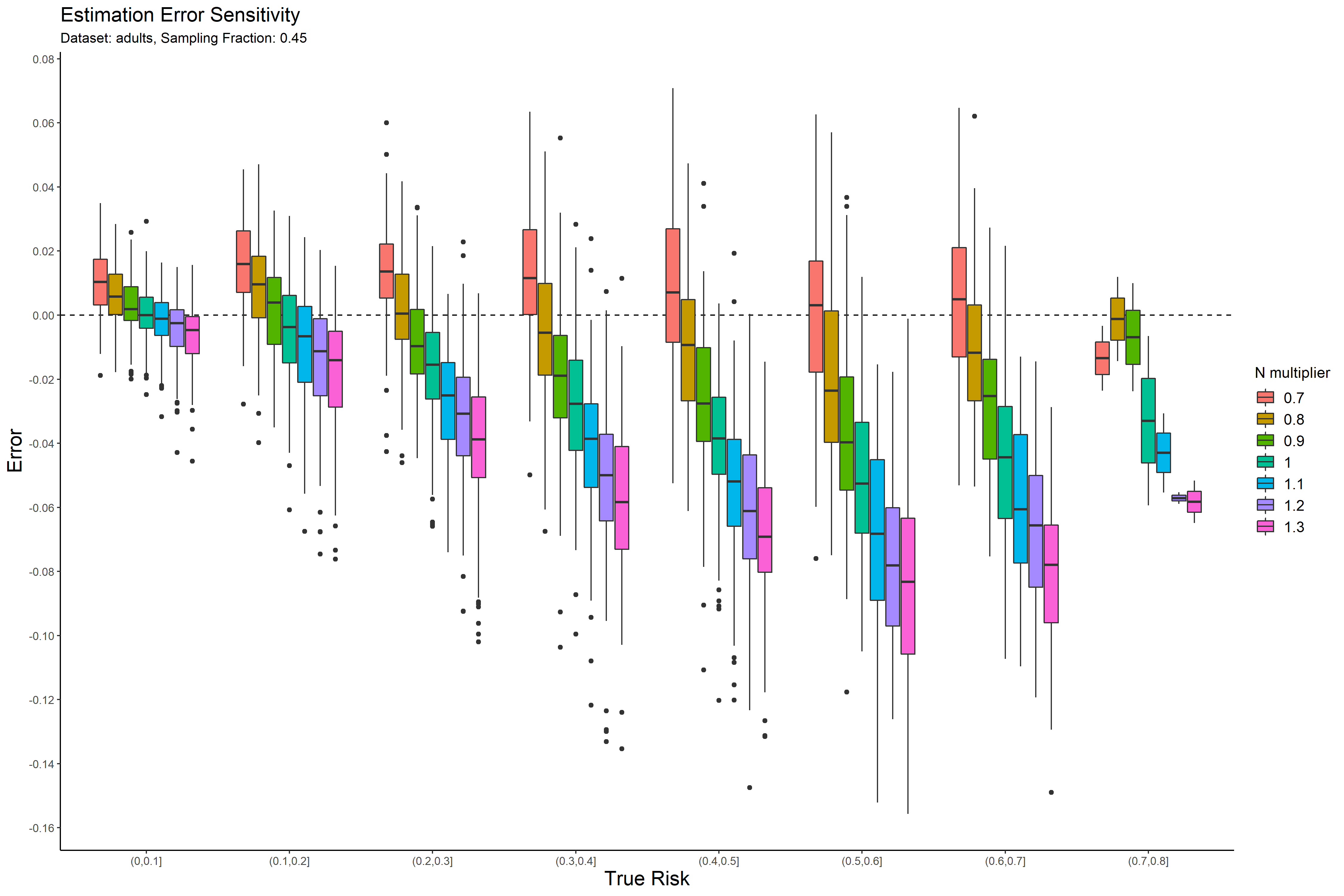

Supplement: S2 File — (ZIP) [file pone.0269097.s002.zip › adults/sensitivity.adults.9.png]

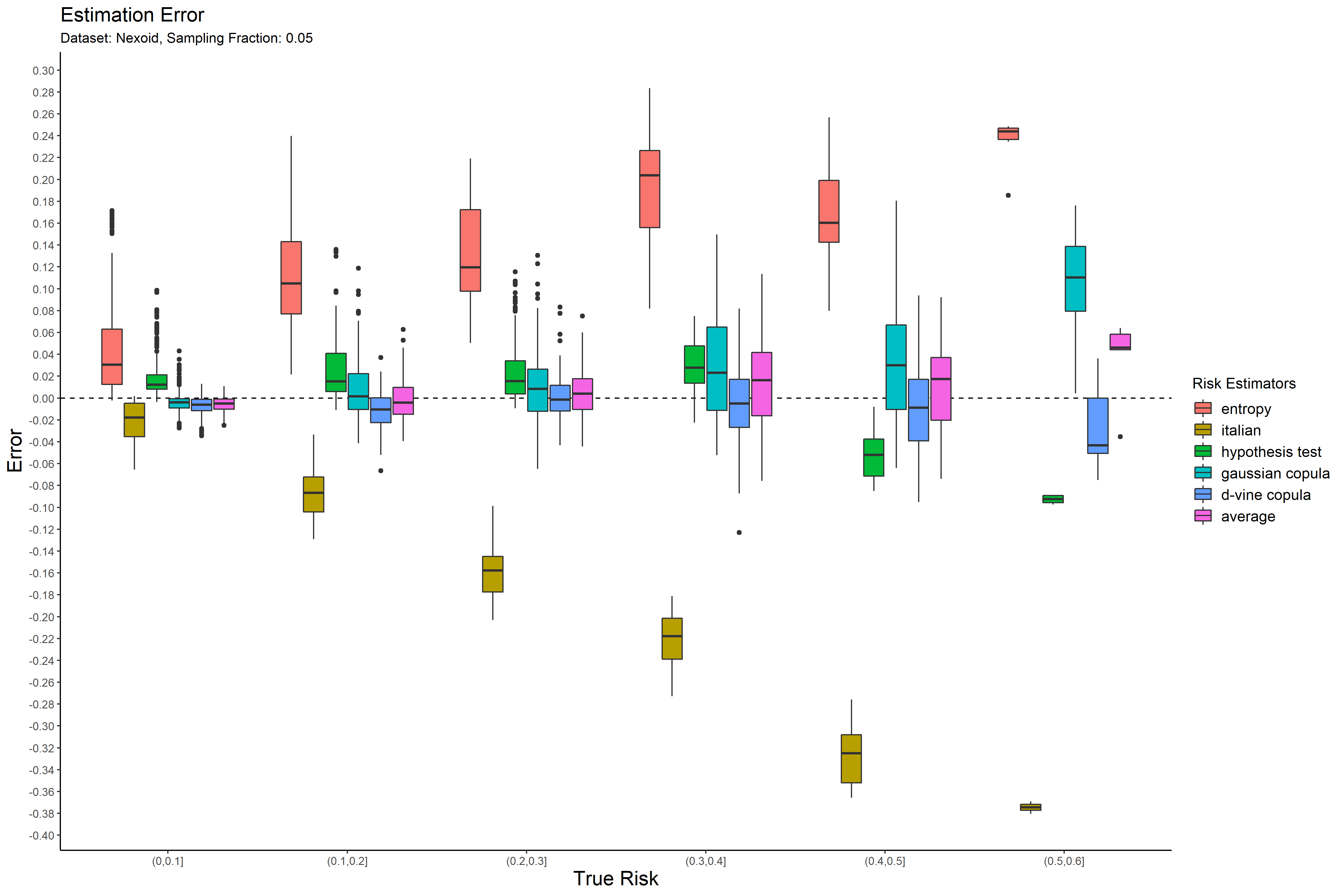

Supplement: S2 File — (ZIP) [file pone.0269097.s002.zip › nexoid/comparison.nexoid.1.png]

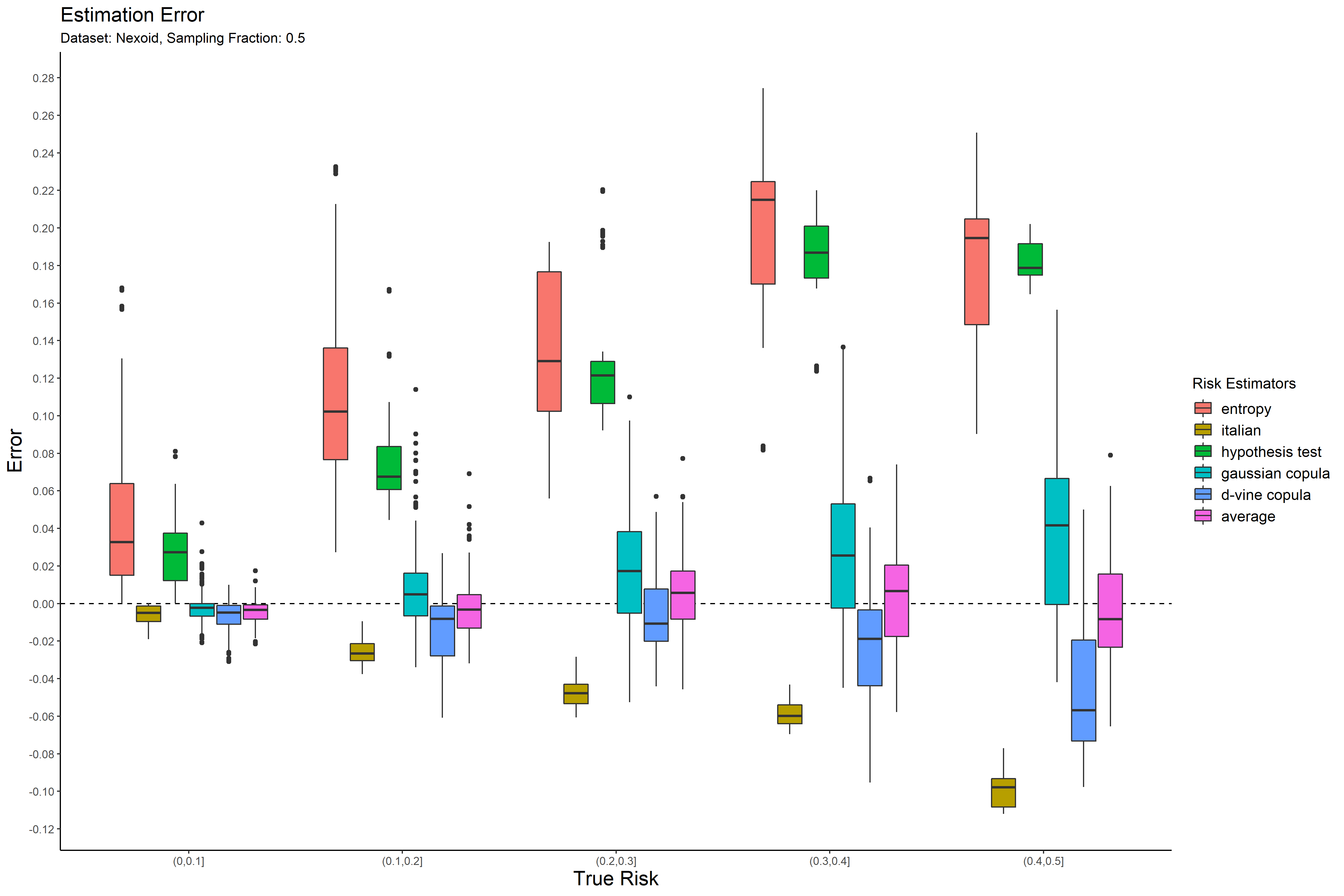

Supplement: S2 File — (ZIP) [file pone.0269097.s002.zip › nexoid/comparison.nexoid.10.png]

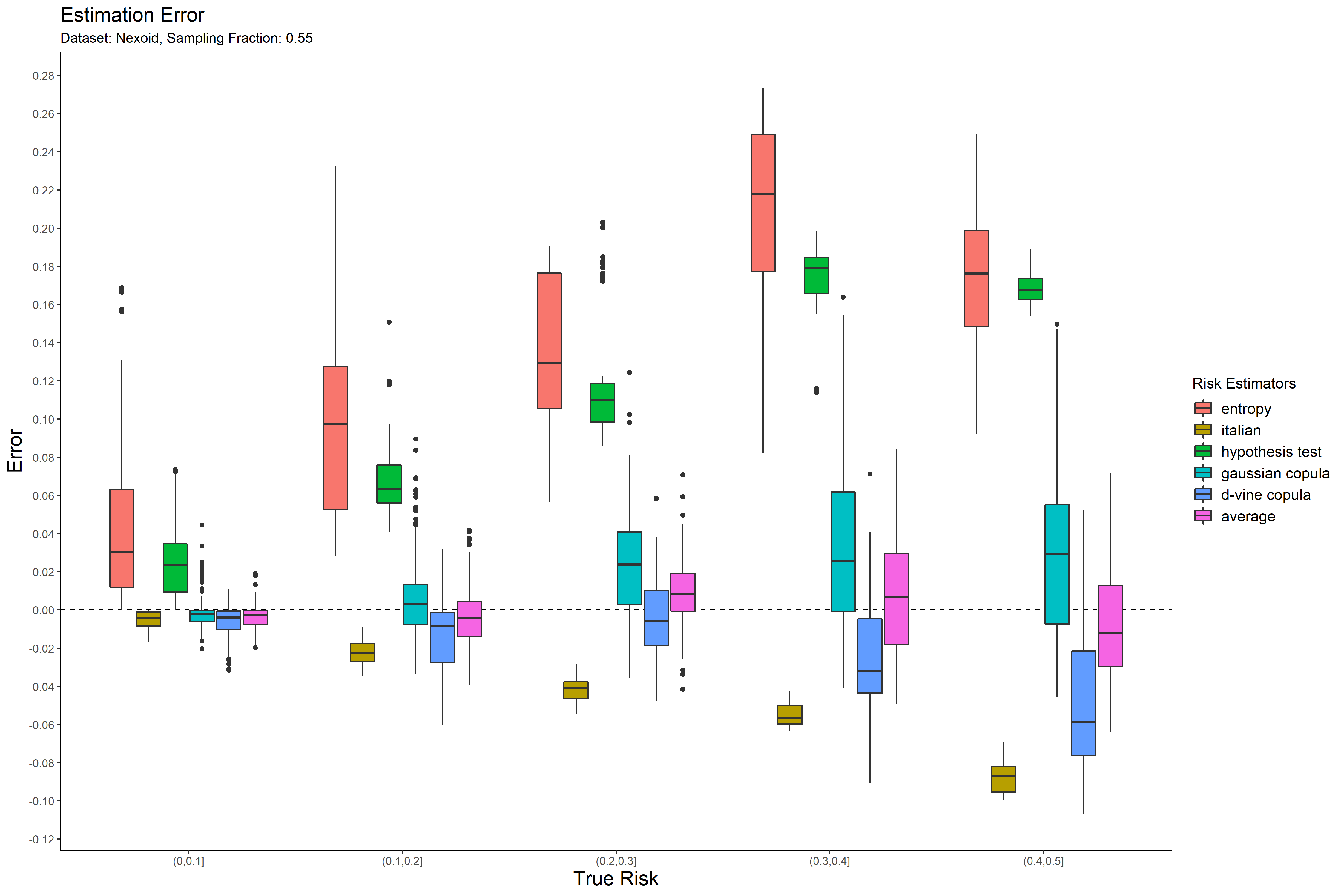

Supplement: S2 File — (ZIP) [file pone.0269097.s002.zip › nexoid/comparison.nexoid.11.png]

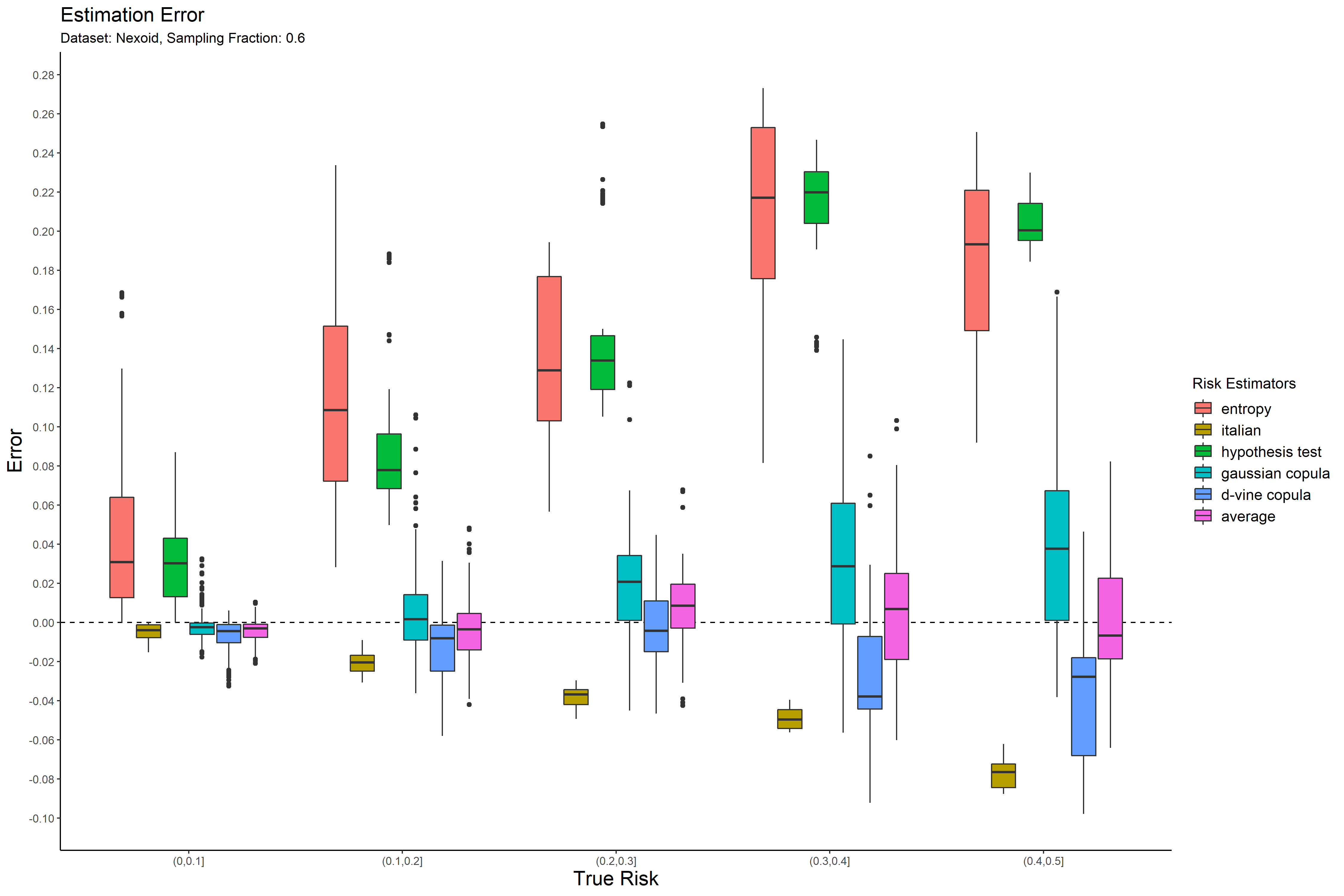

Supplement: S2 File — (ZIP) [file pone.0269097.s002.zip › nexoid/comparison.nexoid.12.png]

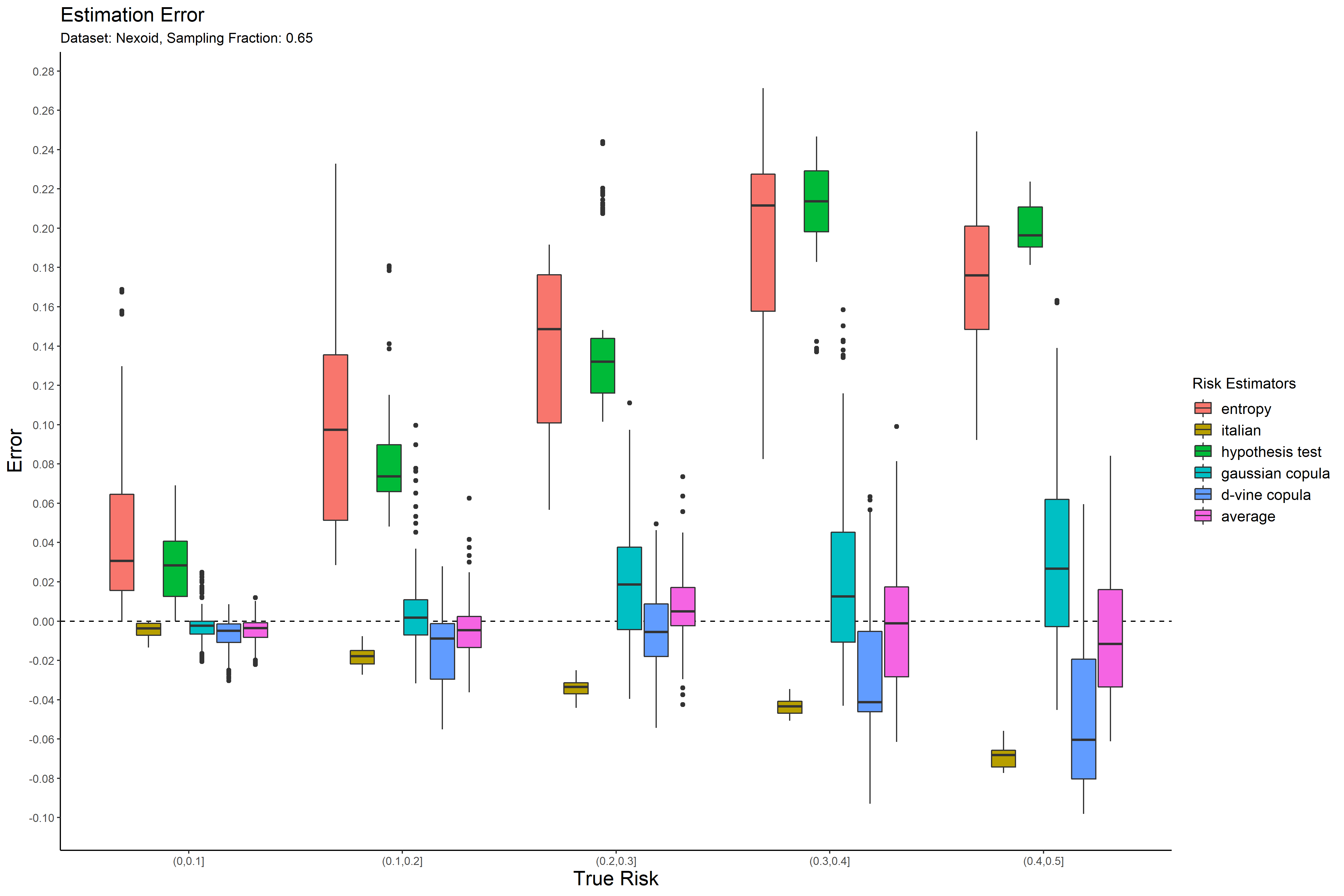

Supplement: S2 File — (ZIP) [file pone.0269097.s002.zip › nexoid/comparison.nexoid.13.png]

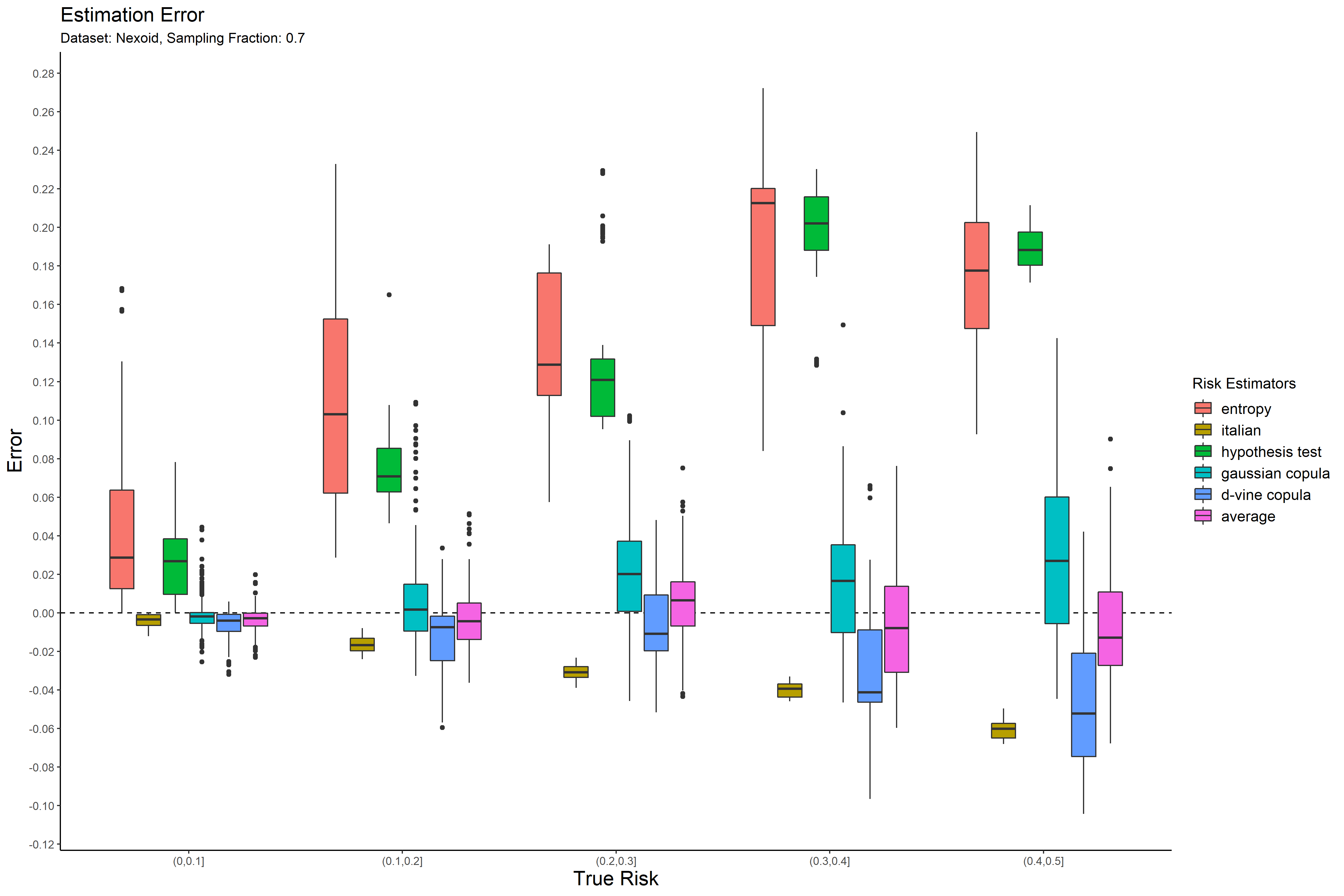

Supplement: S2 File — (ZIP) [file pone.0269097.s002.zip › nexoid/comparison.nexoid.14.png]

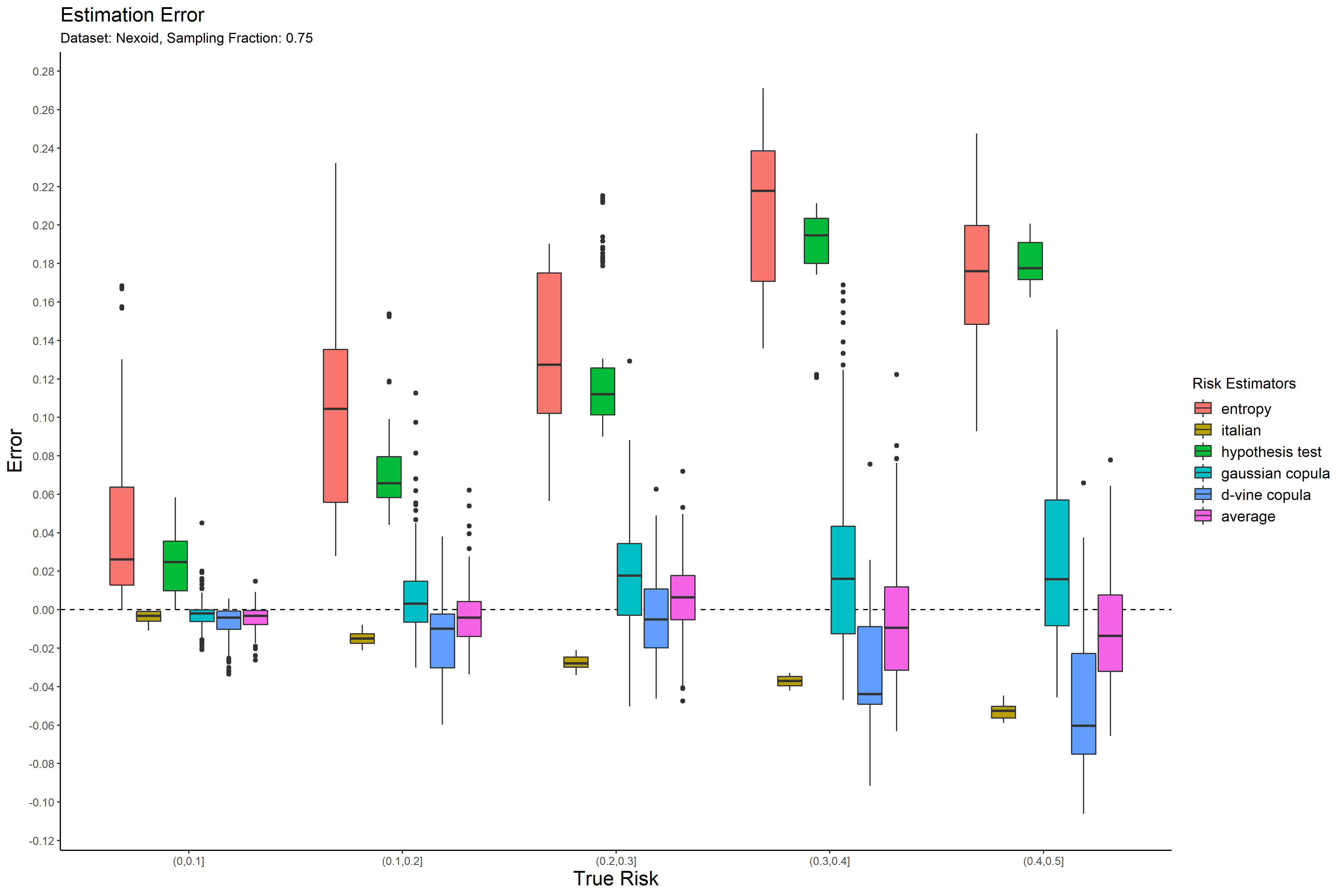

Supplement: S2 File — (ZIP) [file pone.0269097.s002.zip › nexoid/comparison.nexoid.15.png]

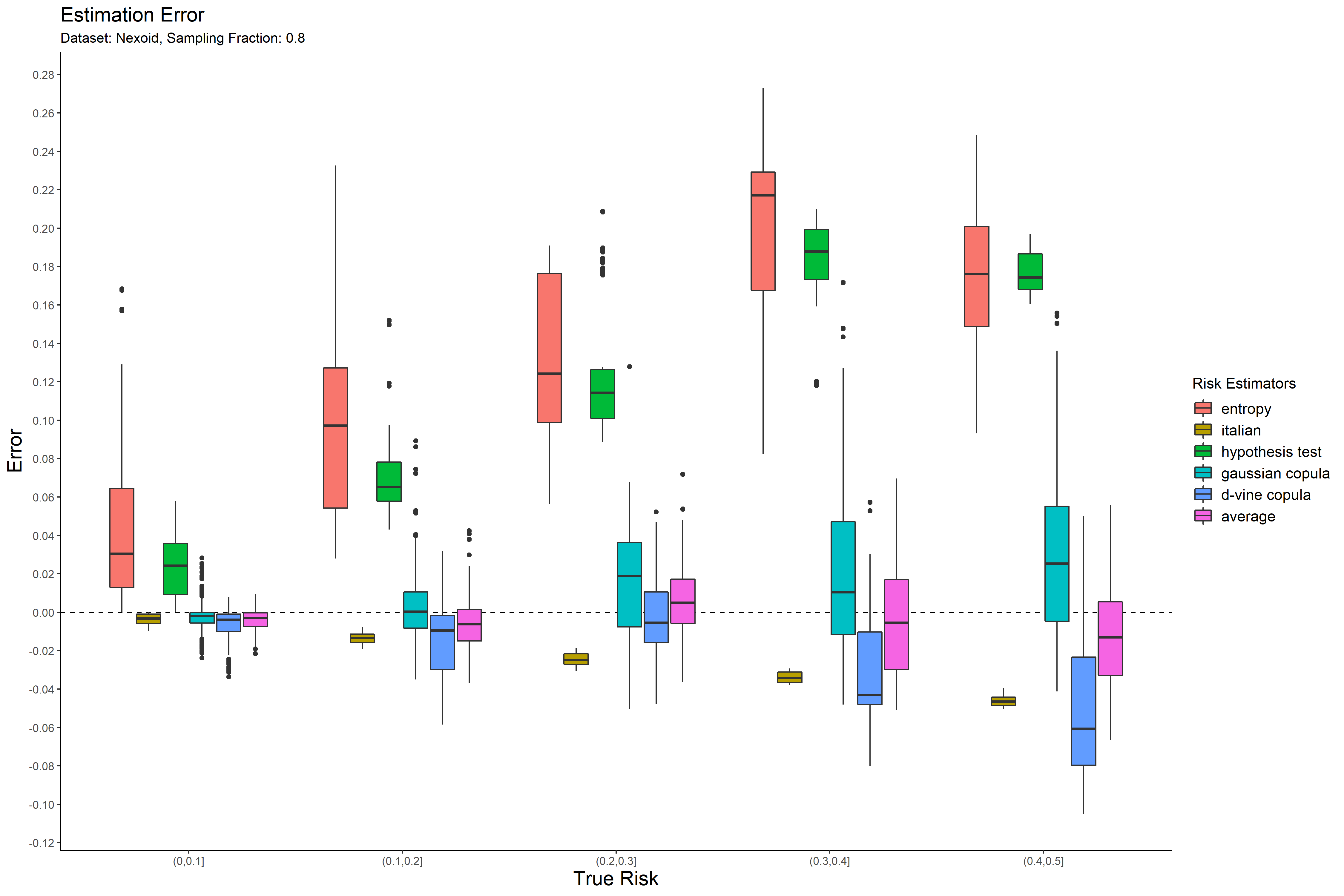

Supplement: S2 File — (ZIP) [file pone.0269097.s002.zip › nexoid/comparison.nexoid.16.png]

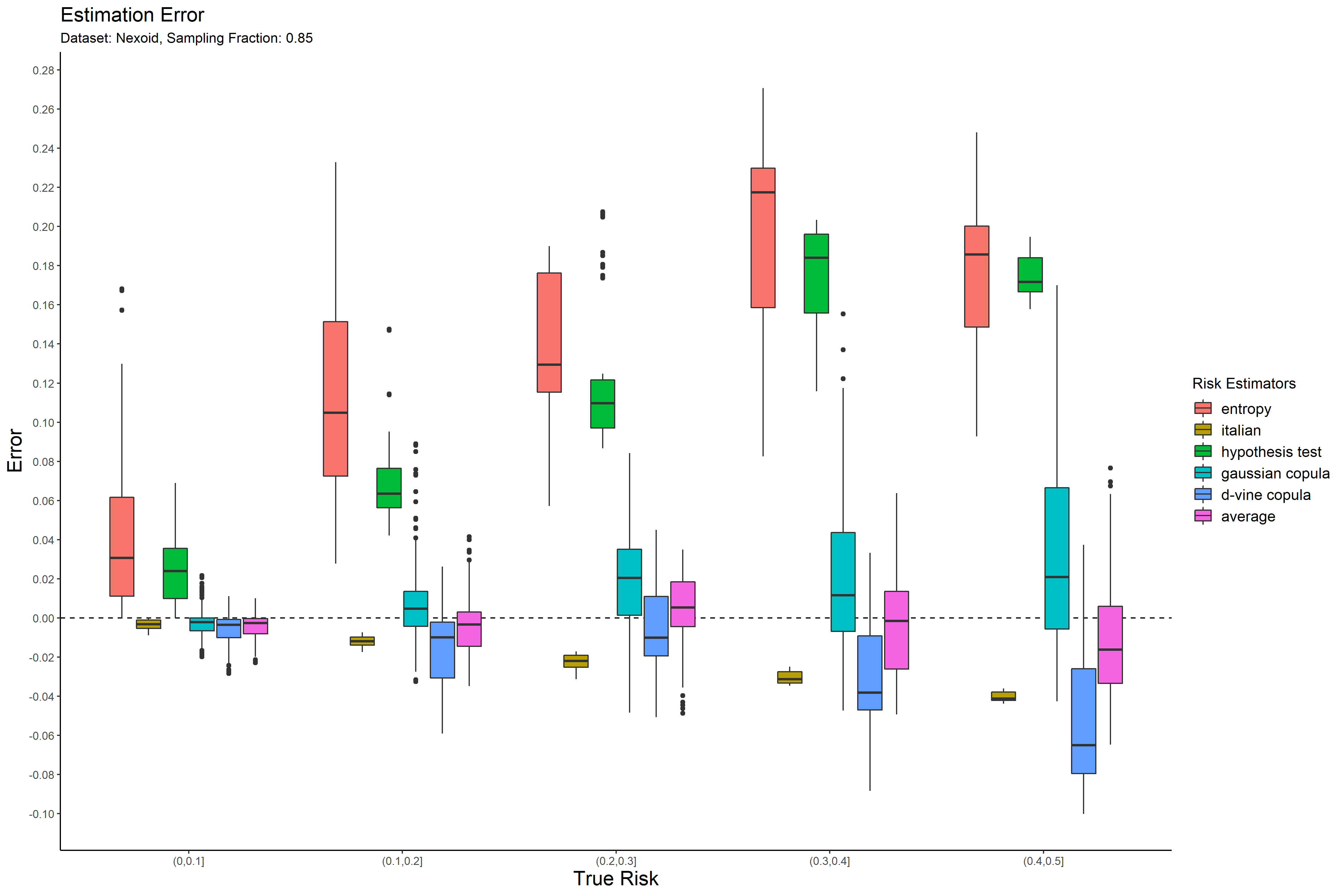

Supplement: S2 File — (ZIP) [file pone.0269097.s002.zip › nexoid/comparison.nexoid.17.png]

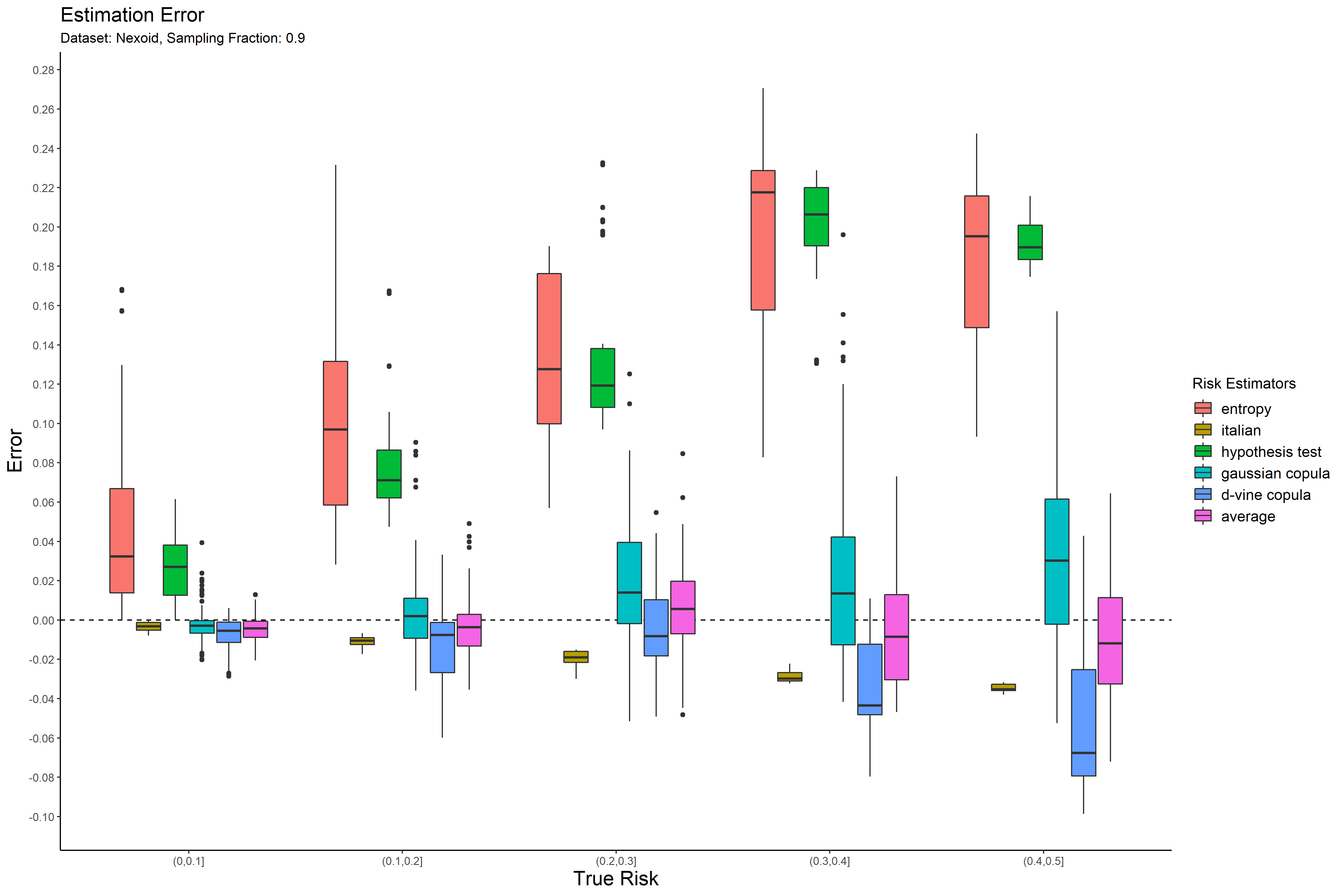

Supplement: S2 File — (ZIP) [file pone.0269097.s002.zip › nexoid/comparison.nexoid.18.png]

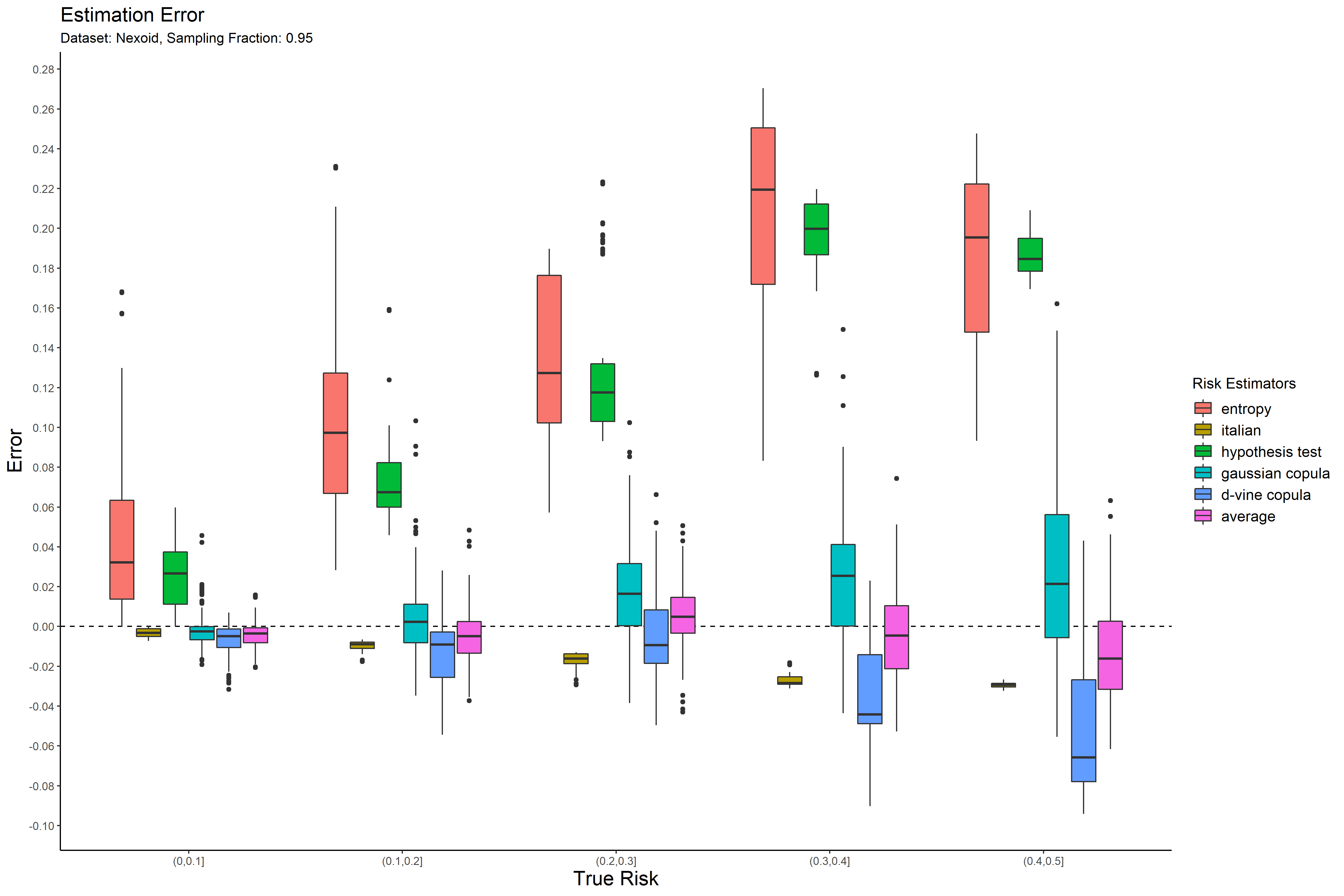

Supplement: S2 File — (ZIP) [file pone.0269097.s002.zip › nexoid/comparison.nexoid.19.png]

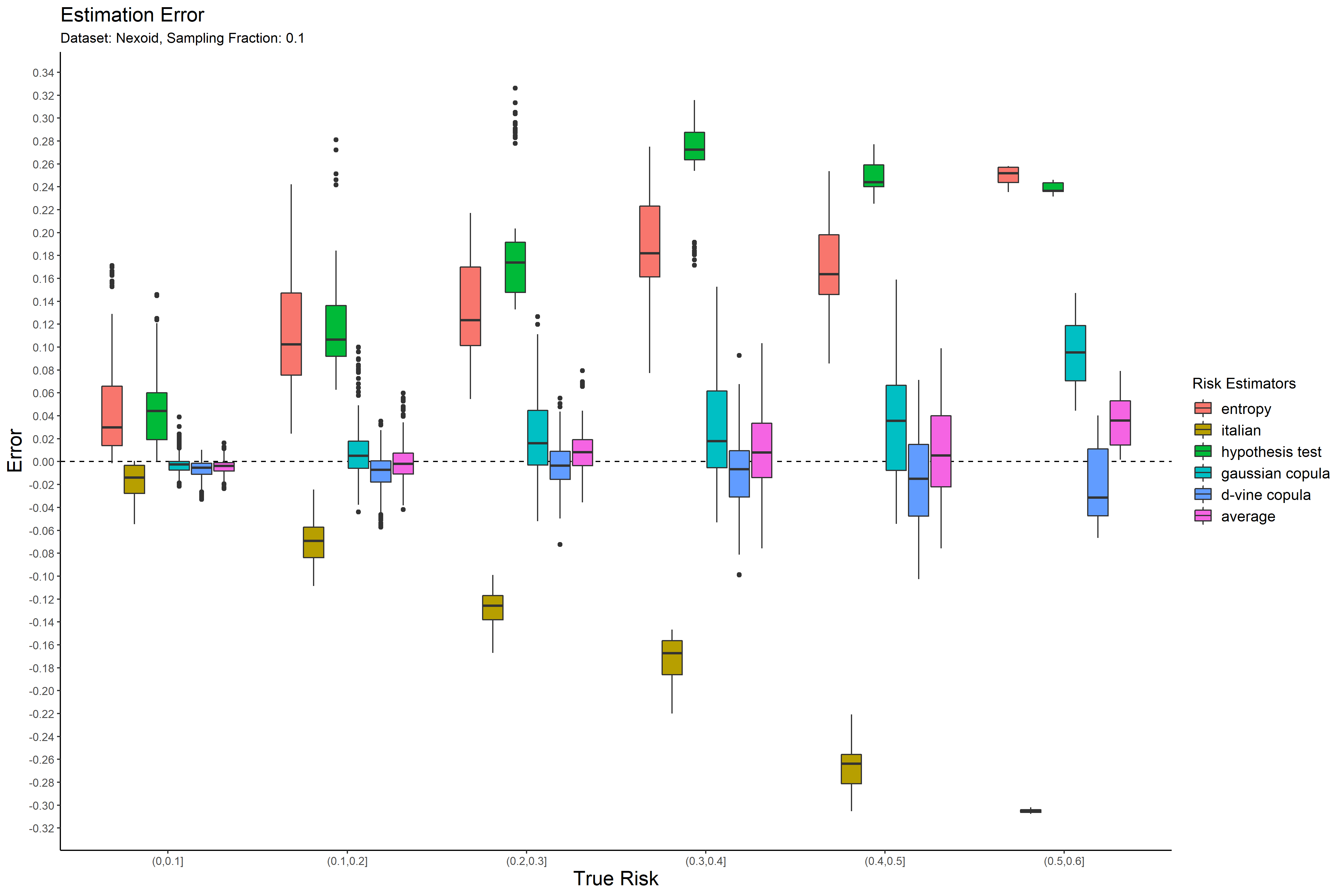

Supplement: S2 File — (ZIP) [file pone.0269097.s002.zip › nexoid/comparison.nexoid.2.png]

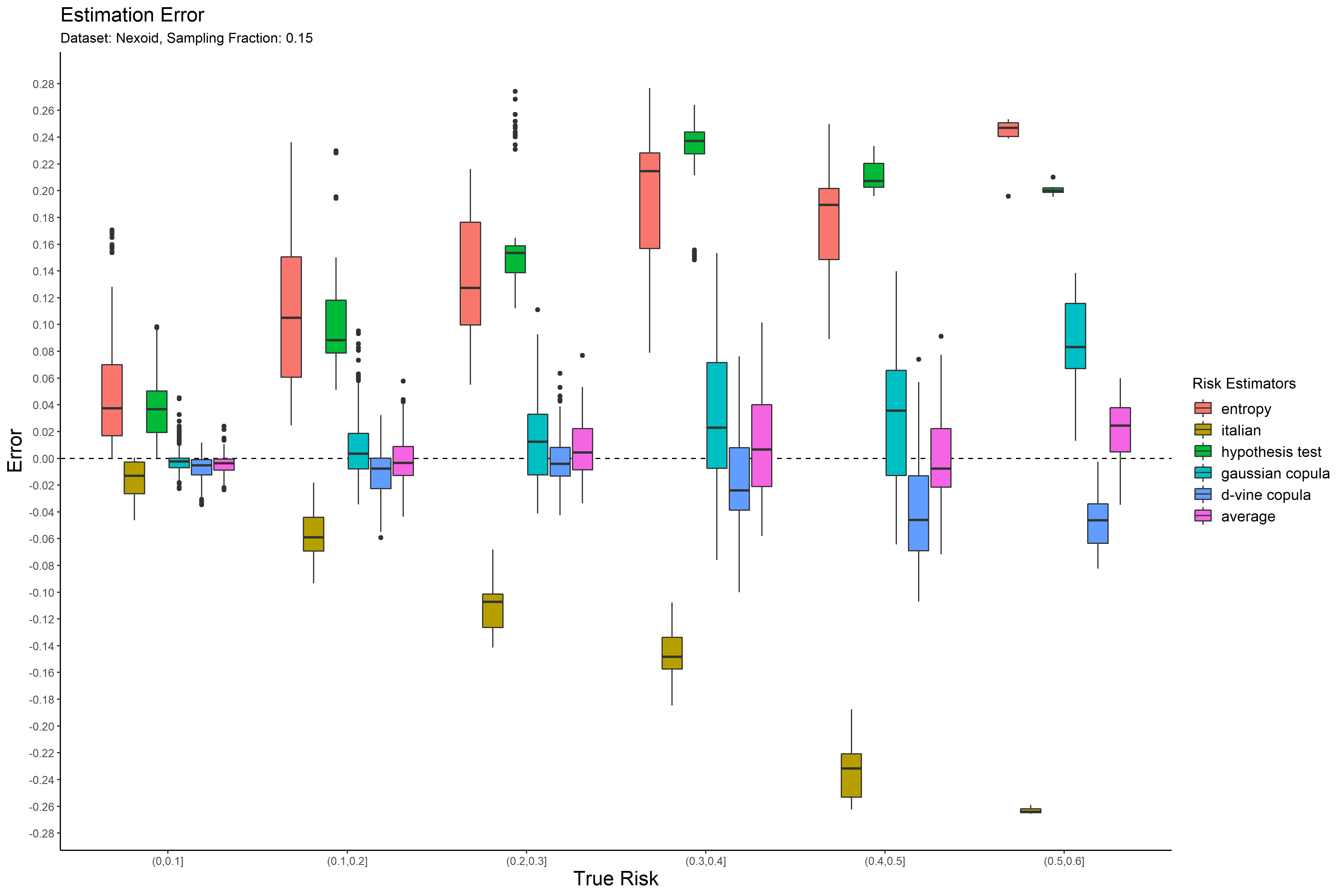

Supplement: S2 File — (ZIP) [file pone.0269097.s002.zip › nexoid/comparison.nexoid.3.png]

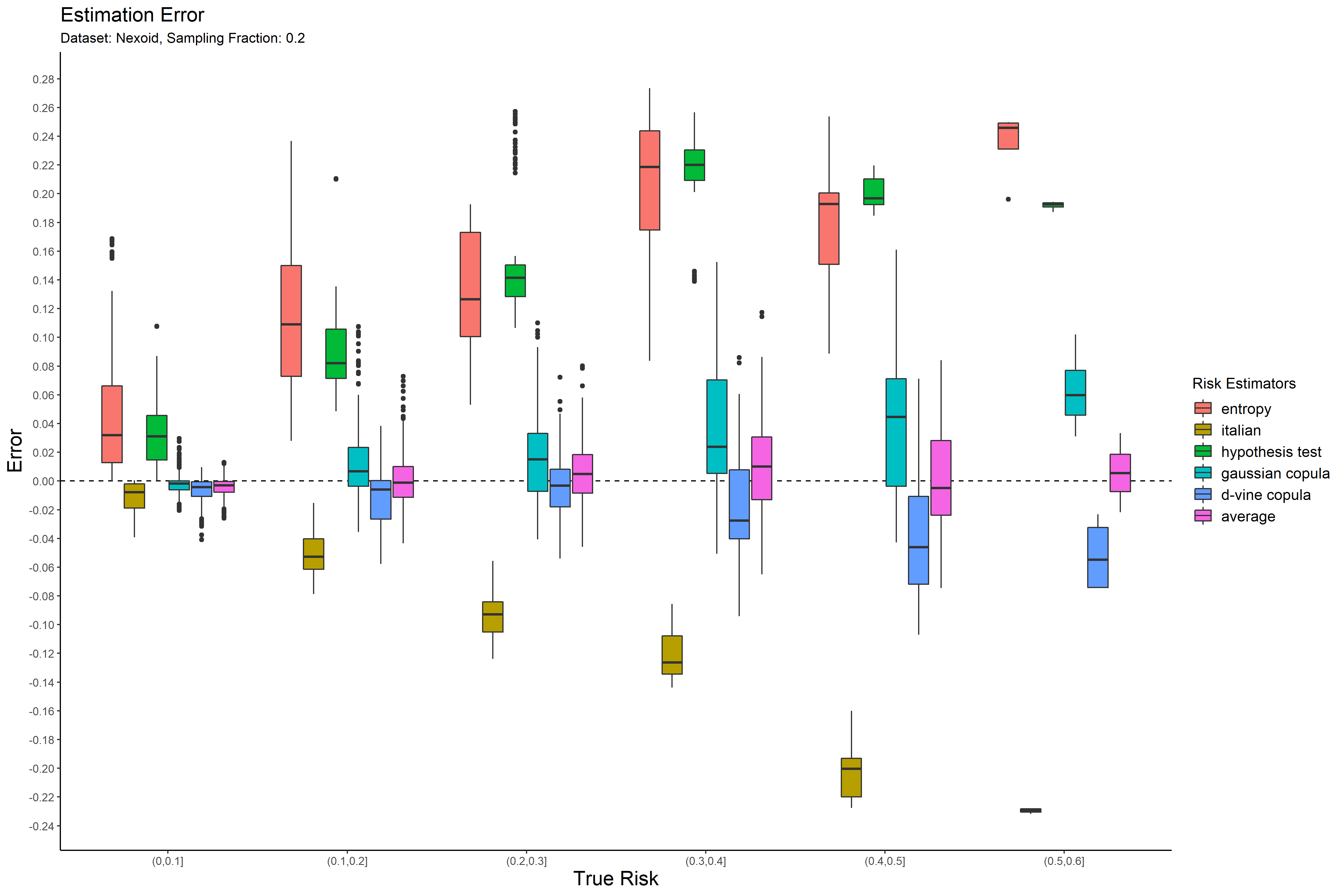

Supplement: S2 File — (ZIP) [file pone.0269097.s002.zip › nexoid/comparison.nexoid.4.png]

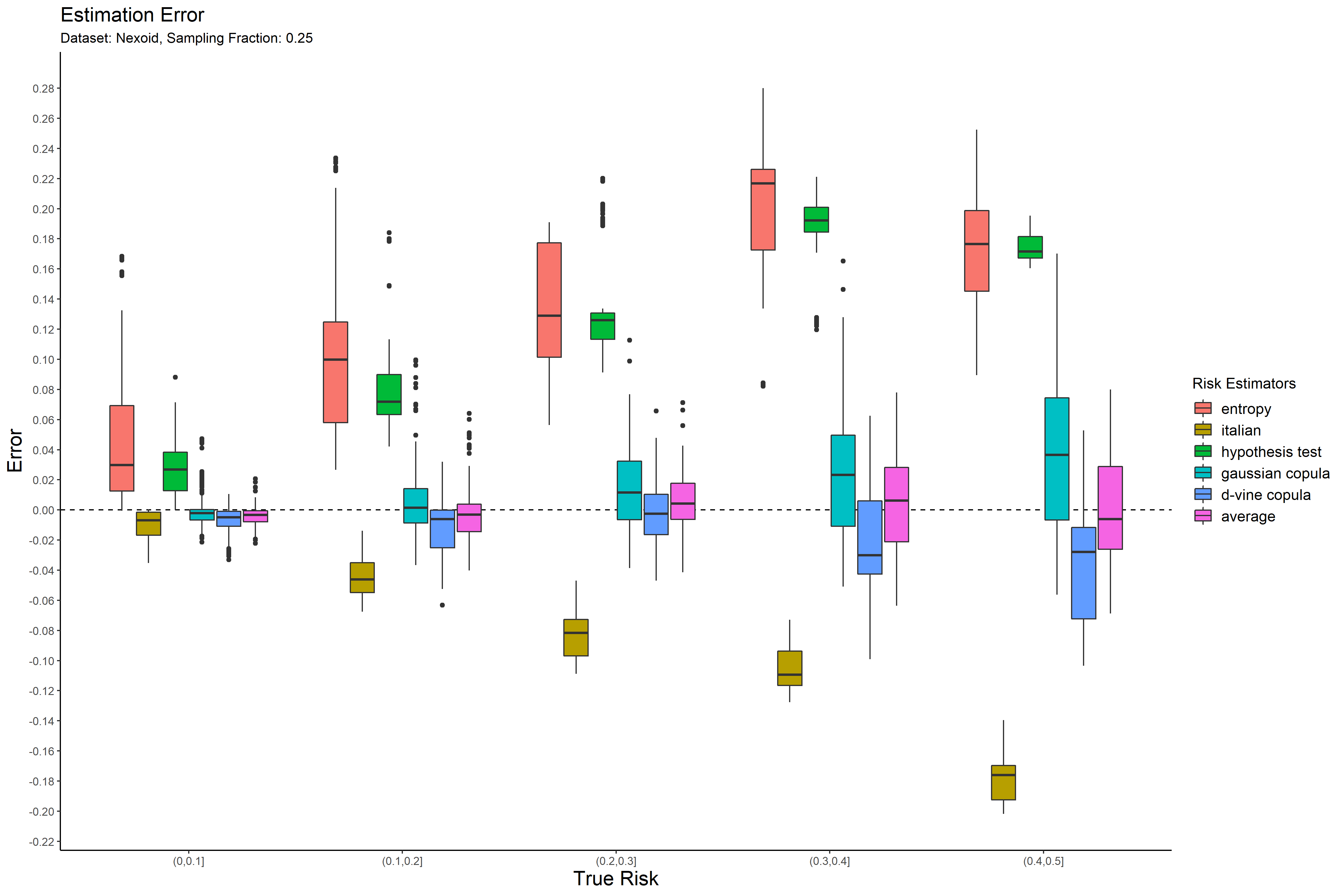

Supplement: S2 File — (ZIP) [file pone.0269097.s002.zip › nexoid/comparison.nexoid.5.png]

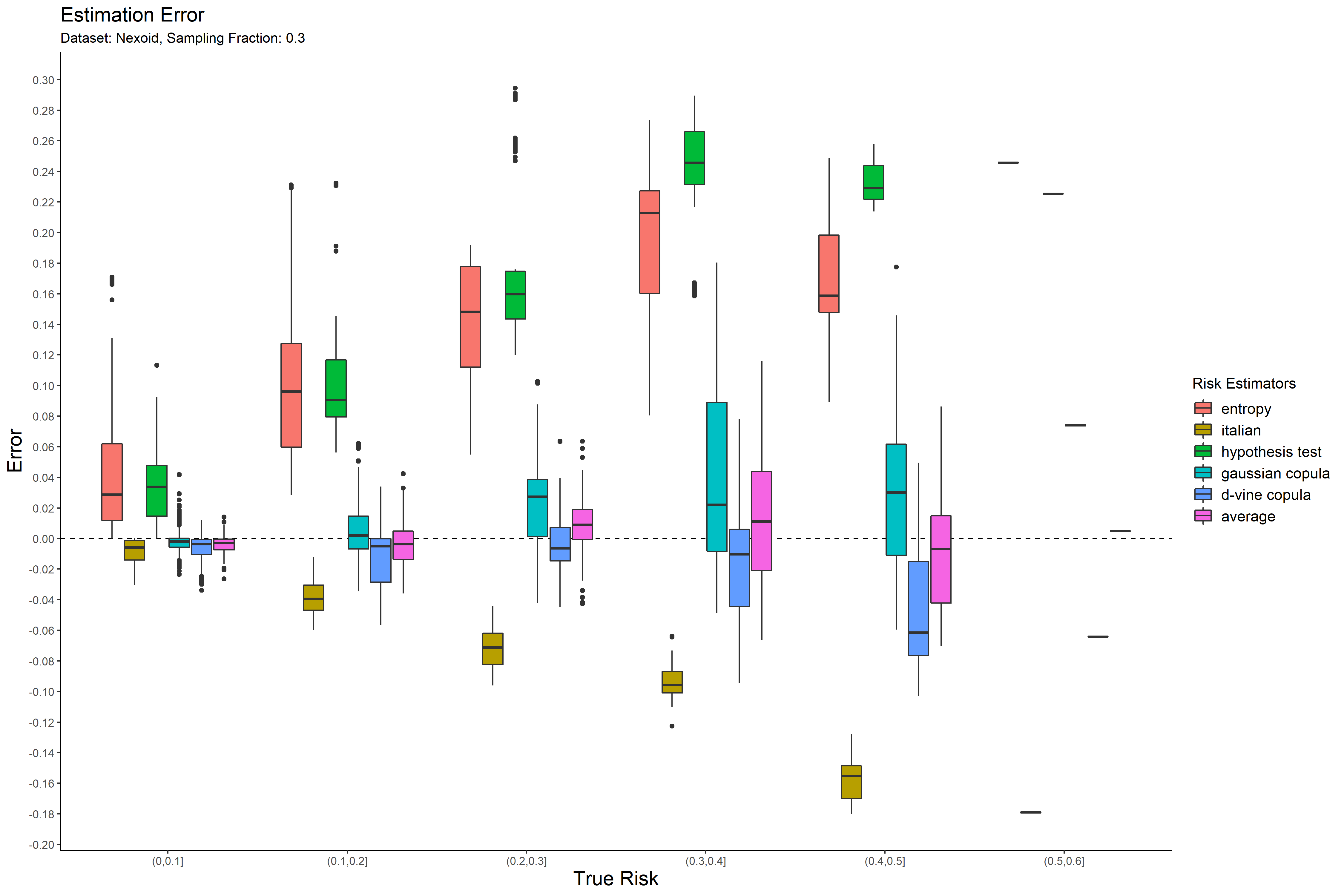

Supplement: S2 File — (ZIP) [file pone.0269097.s002.zip › nexoid/comparison.nexoid.6.png]

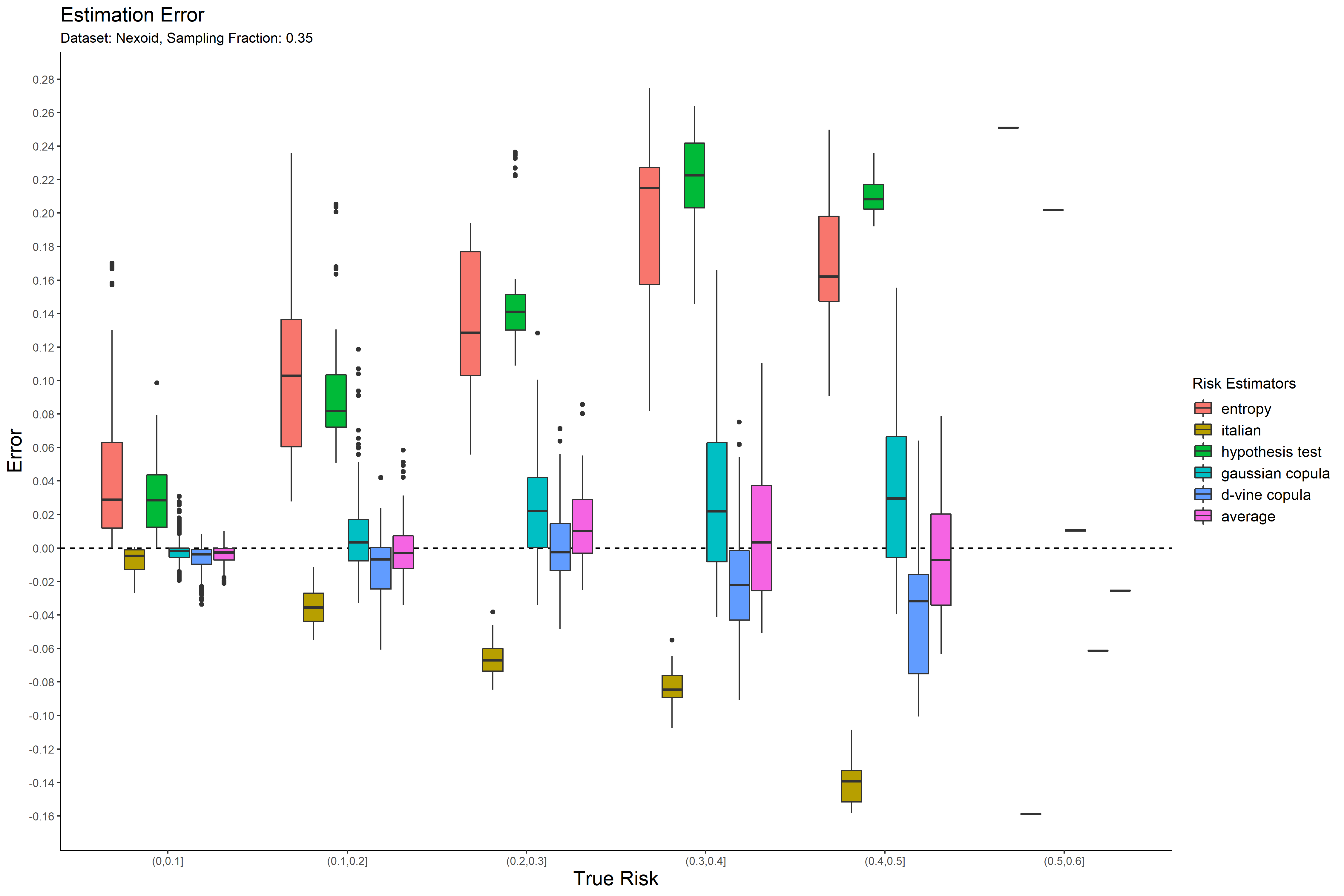

Supplement: S2 File — (ZIP) [file pone.0269097.s002.zip › nexoid/comparison.nexoid.7.png]

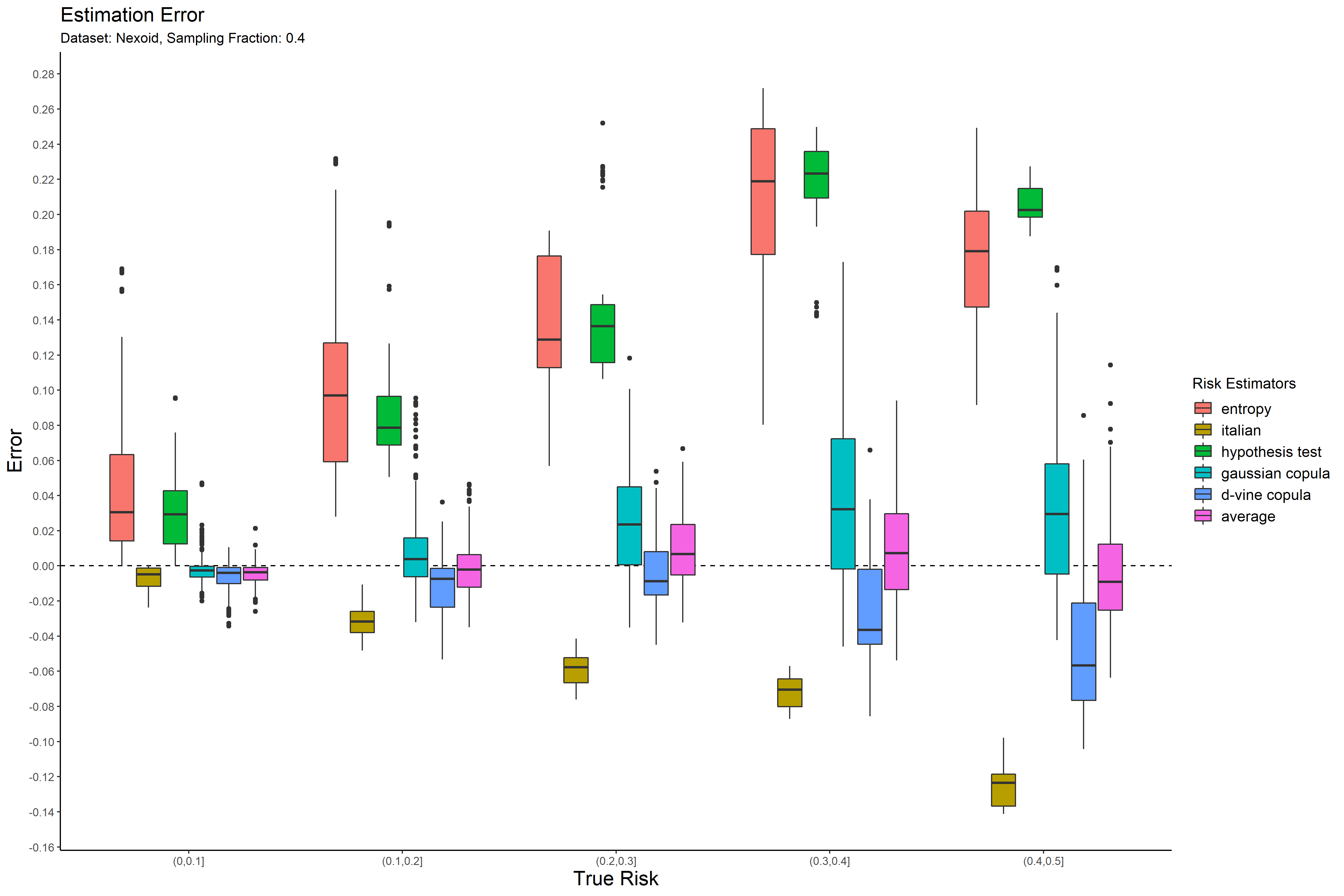

Supplement: S2 File — (ZIP) [file pone.0269097.s002.zip › nexoid/comparison.nexoid.8.png]

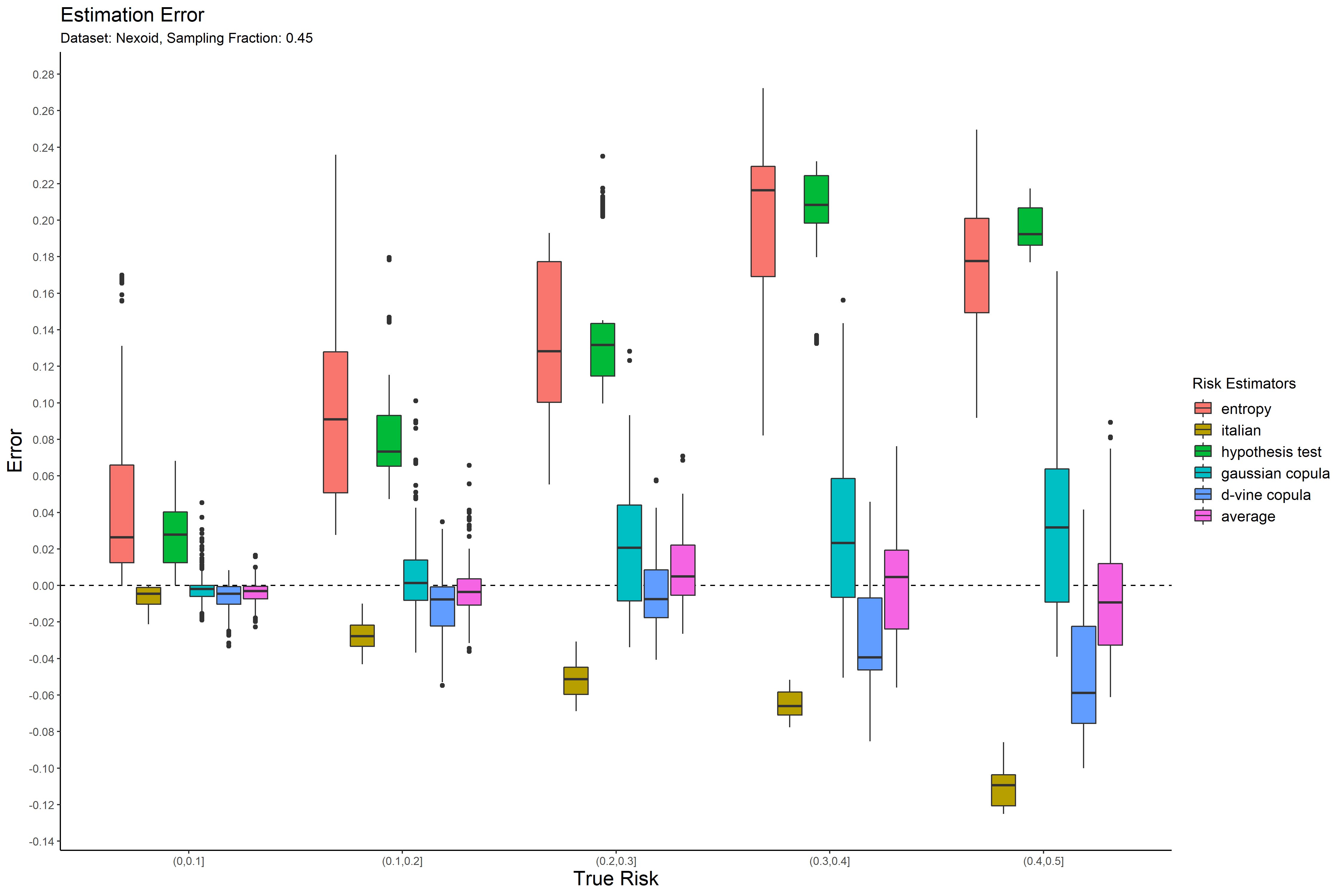

Supplement: S2 File — (ZIP) [file pone.0269097.s002.zip › nexoid/comparison.nexoid.9.png]

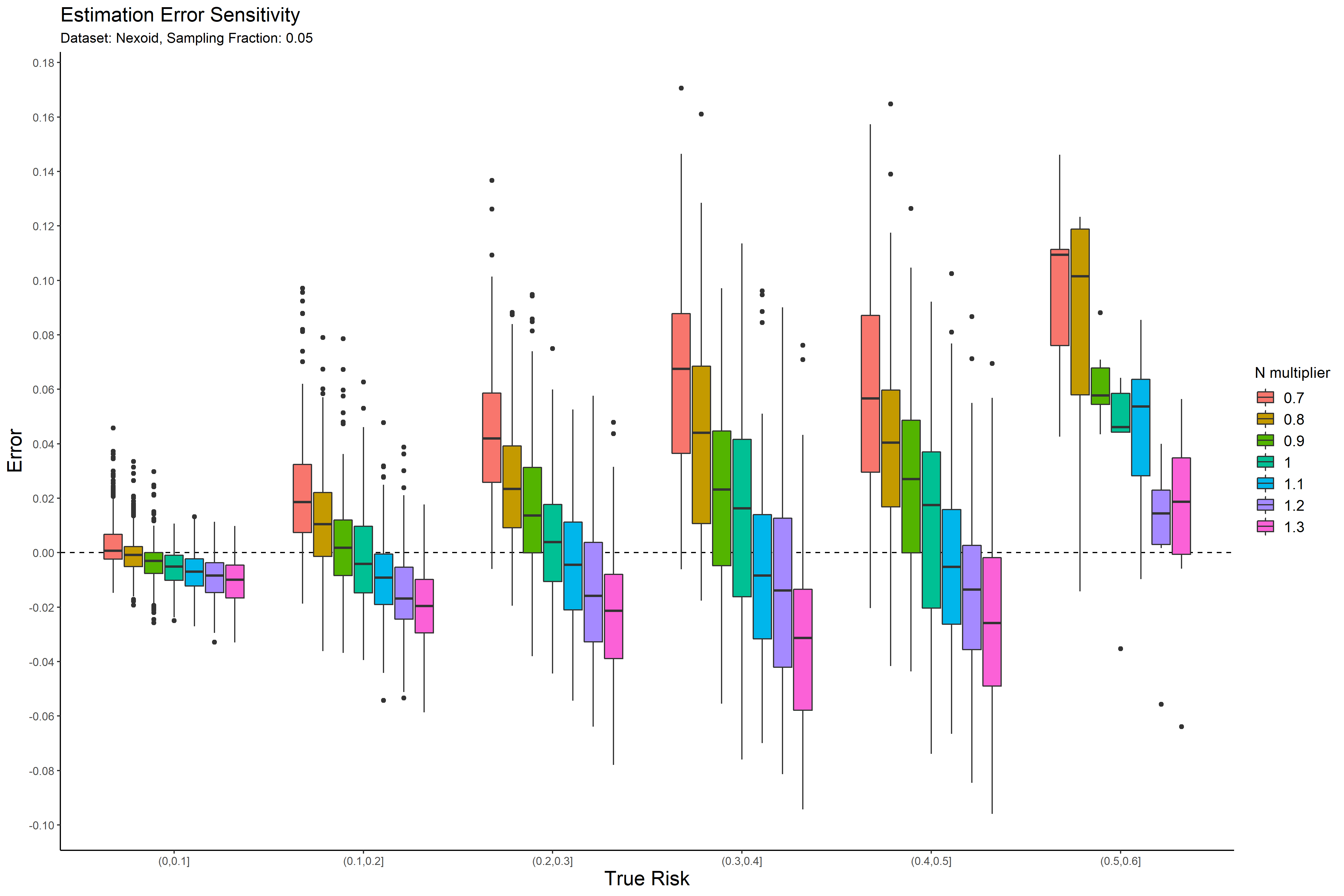

Supplement: S2 File — (ZIP) [file pone.0269097.s002.zip › nexoid/sensitivity.nexoid.1.png]

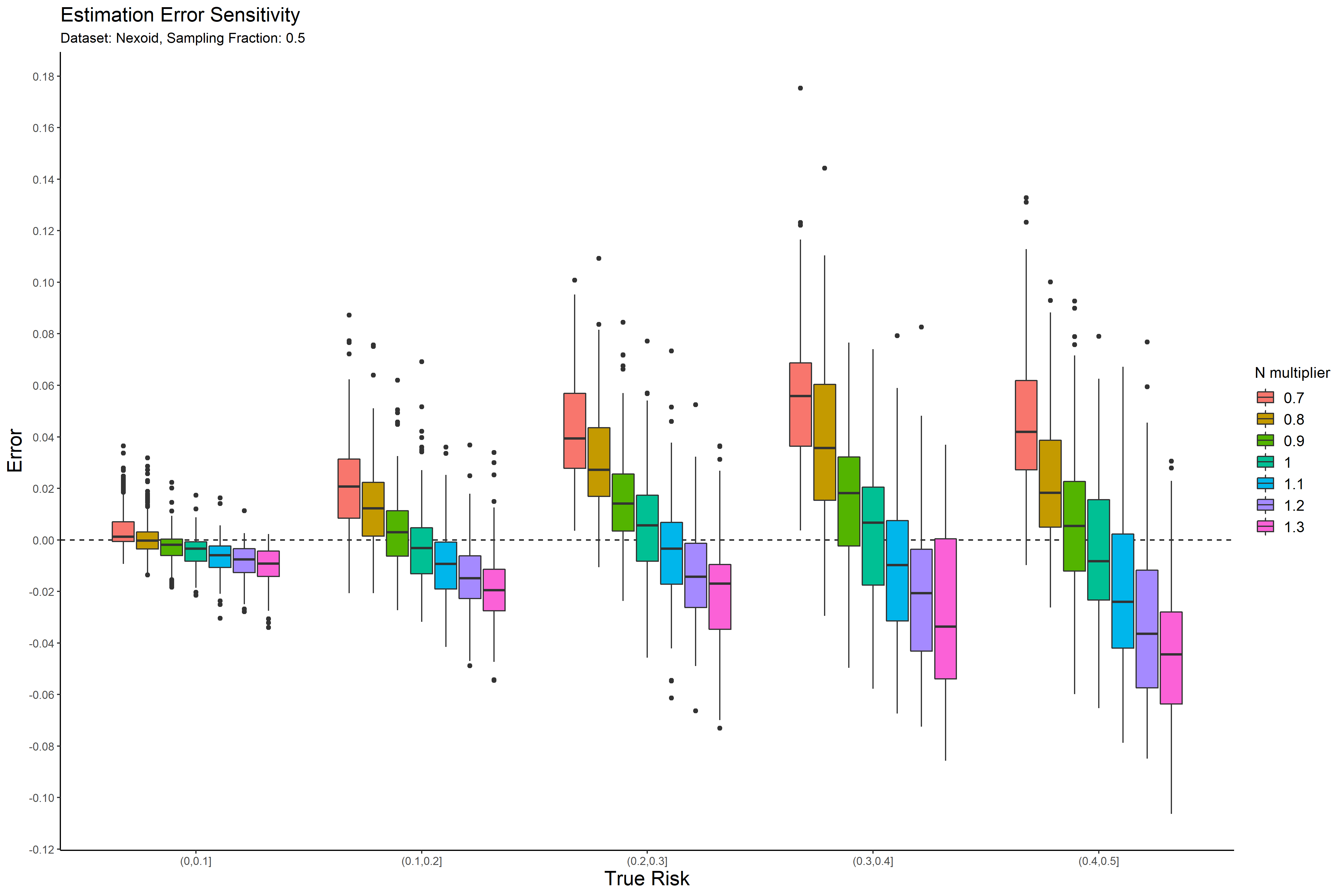

Supplement: S2 File — (ZIP) [file pone.0269097.s002.zip › nexoid/sensitivity.nexoid.10.png]

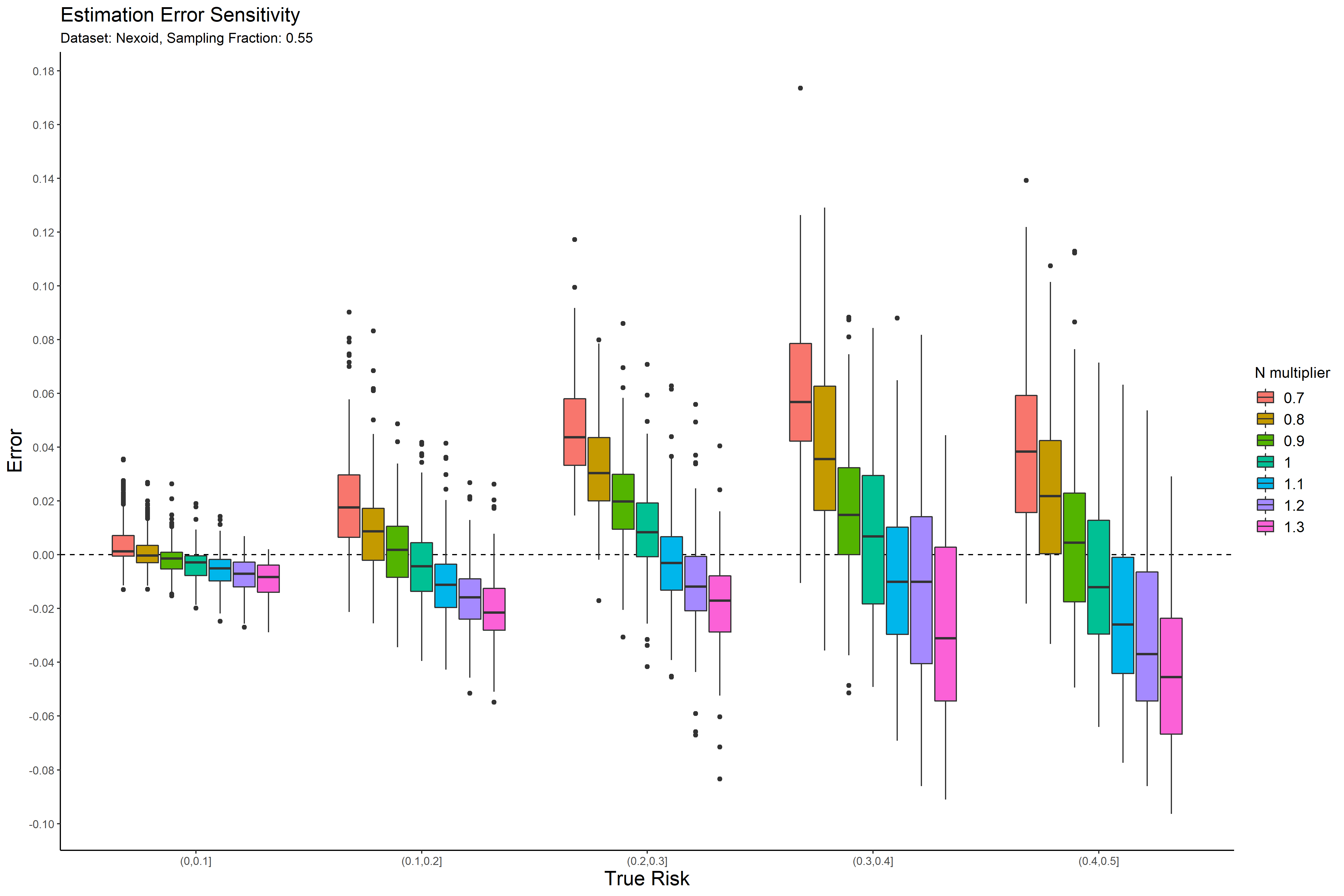

Supplement: S2 File — (ZIP) [file pone.0269097.s002.zip › nexoid/sensitivity.nexoid.11.png]

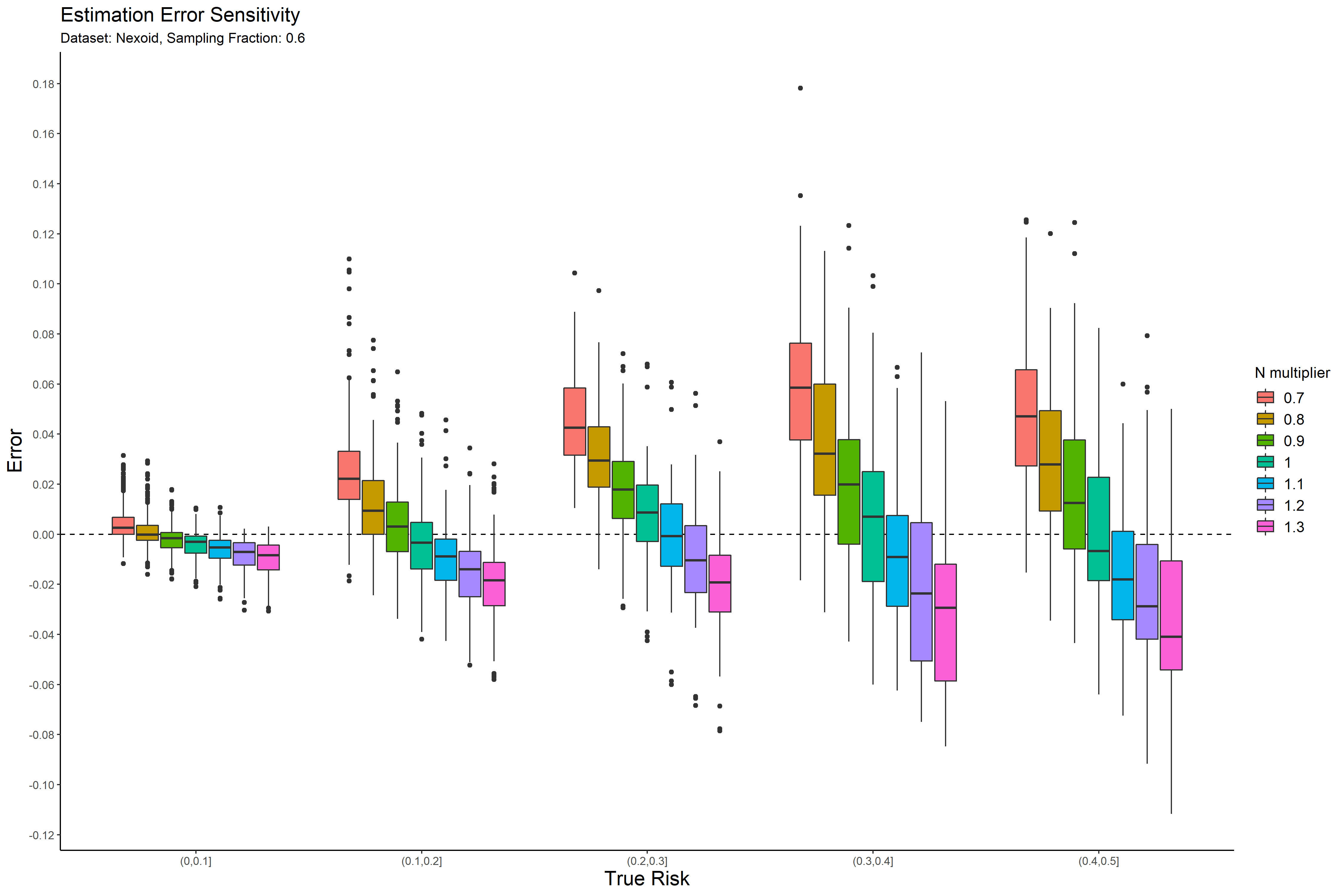

Supplement: S2 File — (ZIP) [file pone.0269097.s002.zip › nexoid/sensitivity.nexoid.12.png]

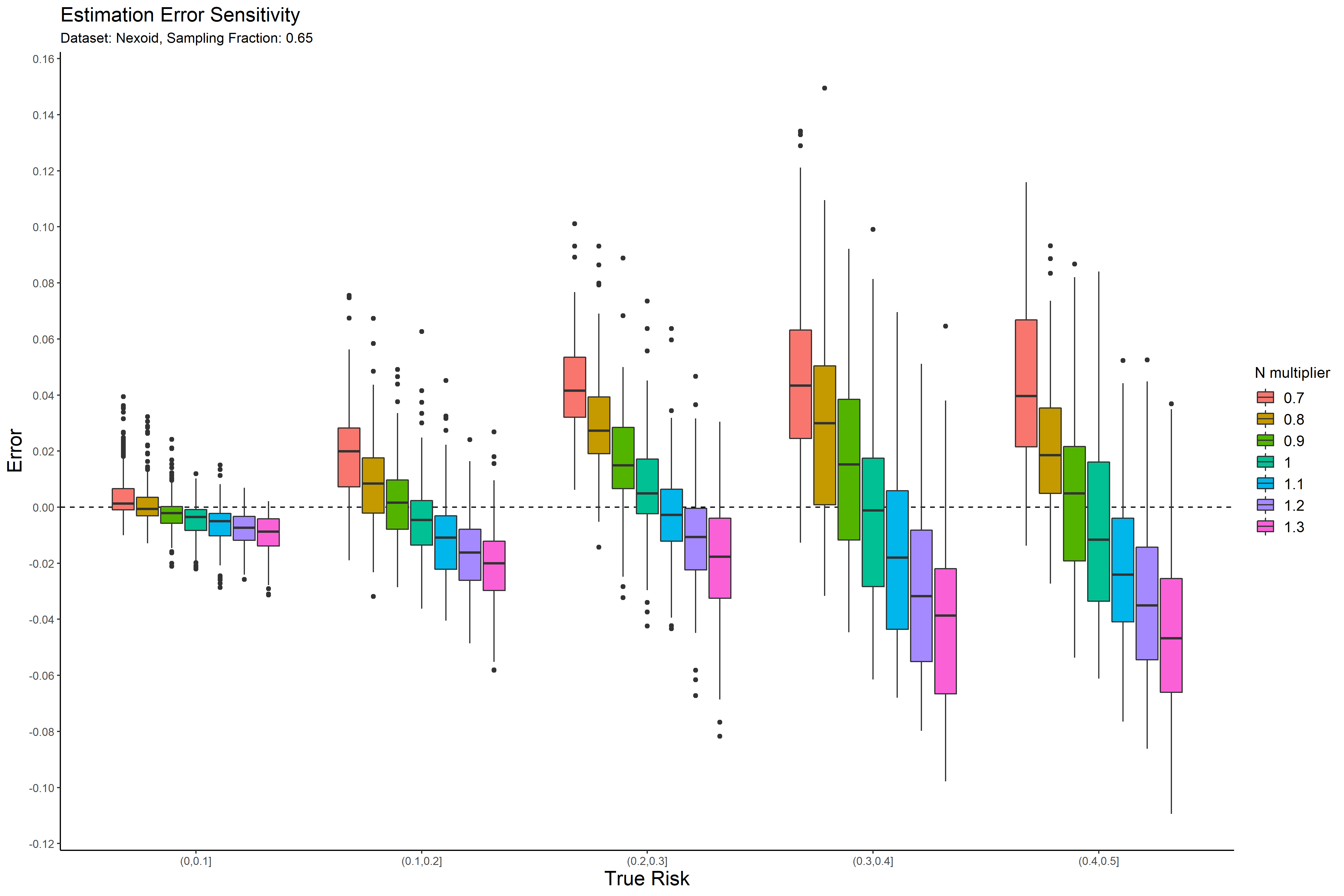

Supplement: S2 File — (ZIP) [file pone.0269097.s002.zip › nexoid/sensitivity.nexoid.13.png]

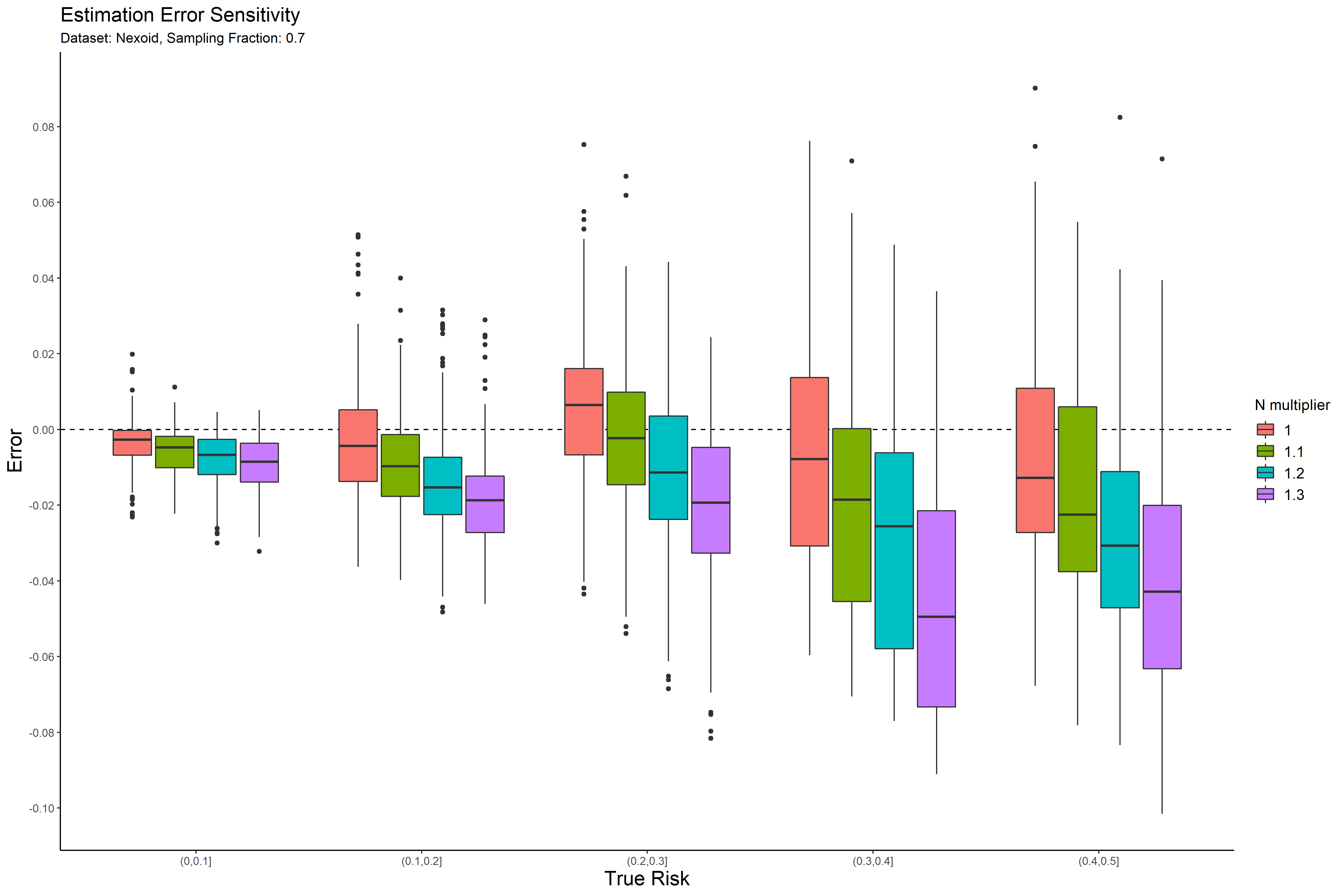

Supplement: S2 File — (ZIP) [file pone.0269097.s002.zip › nexoid/sensitivity.nexoid.14.png]

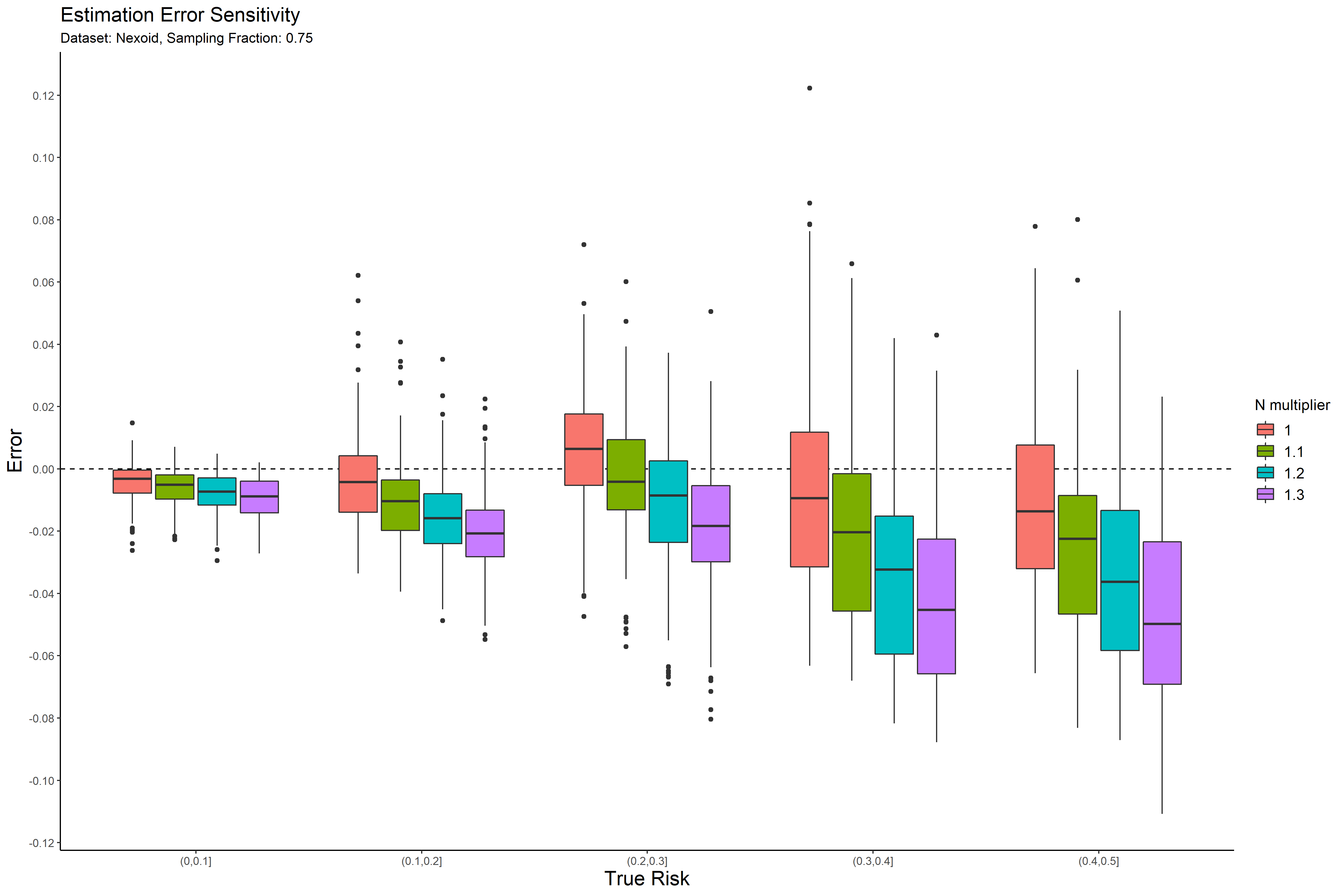

Supplement: S2 File — (ZIP) [file pone.0269097.s002.zip › nexoid/sensitivity.nexoid.15.png]

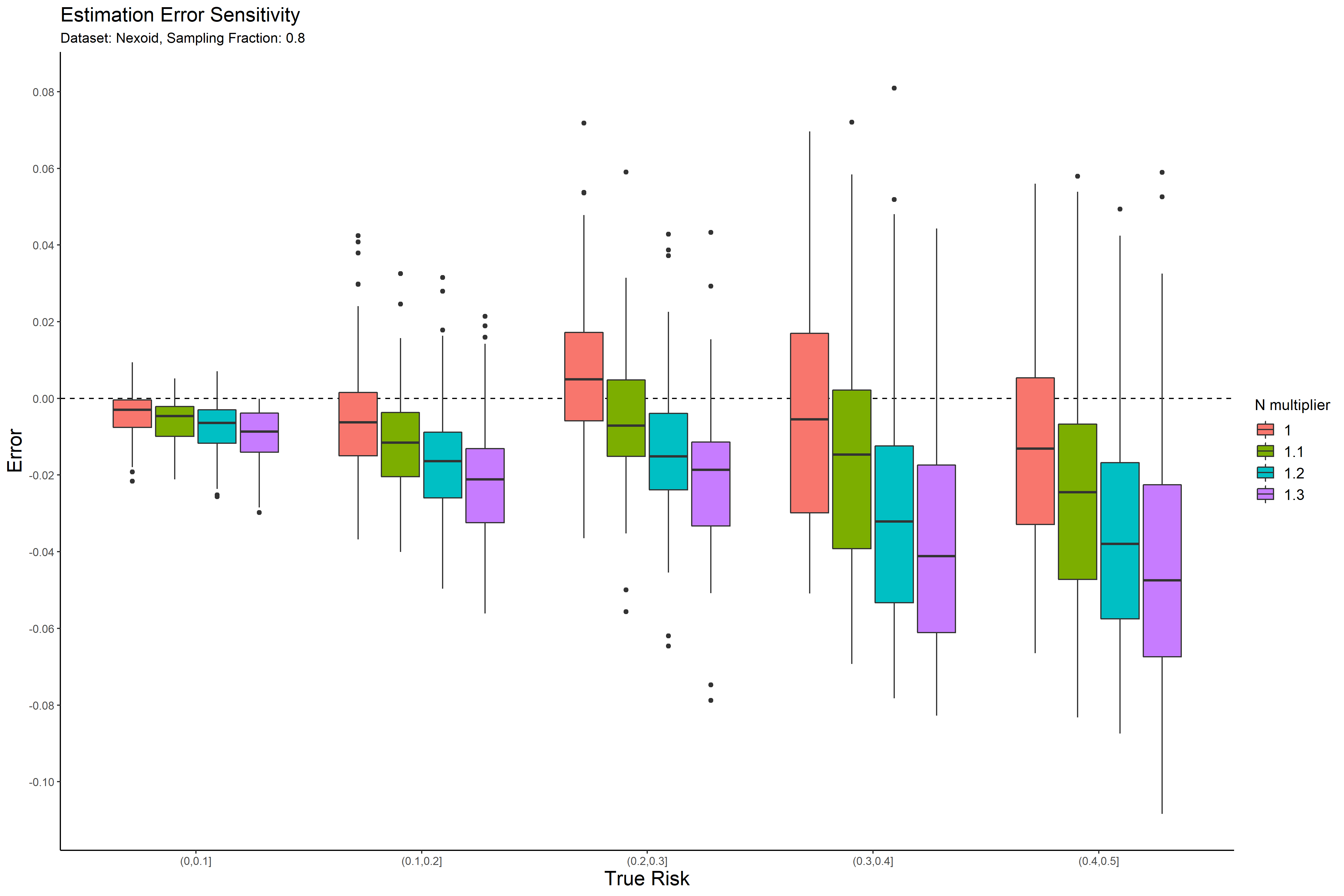

Supplement: S2 File — (ZIP) [file pone.0269097.s002.zip › nexoid/sensitivity.nexoid.16.png]

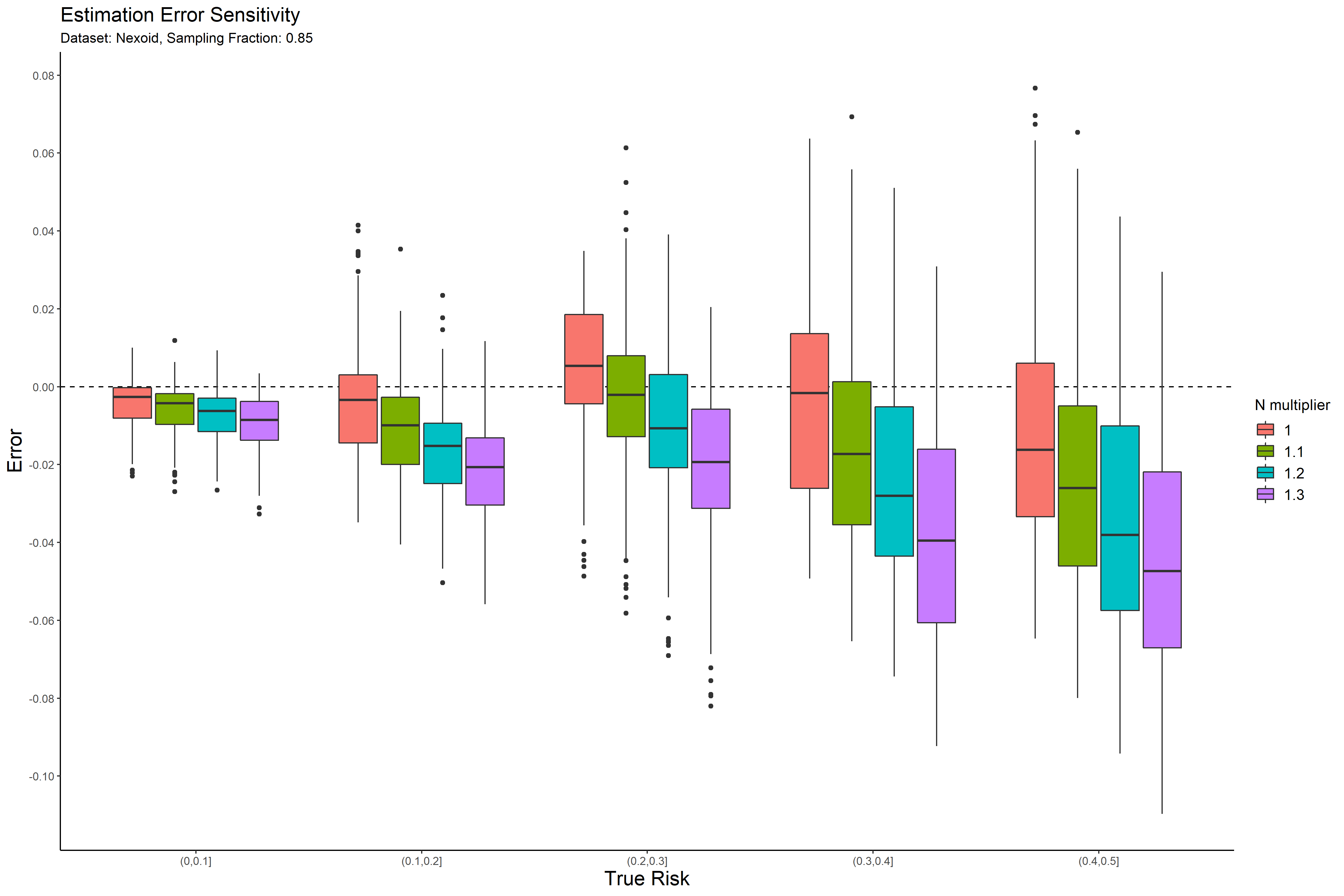

Supplement: S2 File — (ZIP) [file pone.0269097.s002.zip › nexoid/sensitivity.nexoid.17.png]

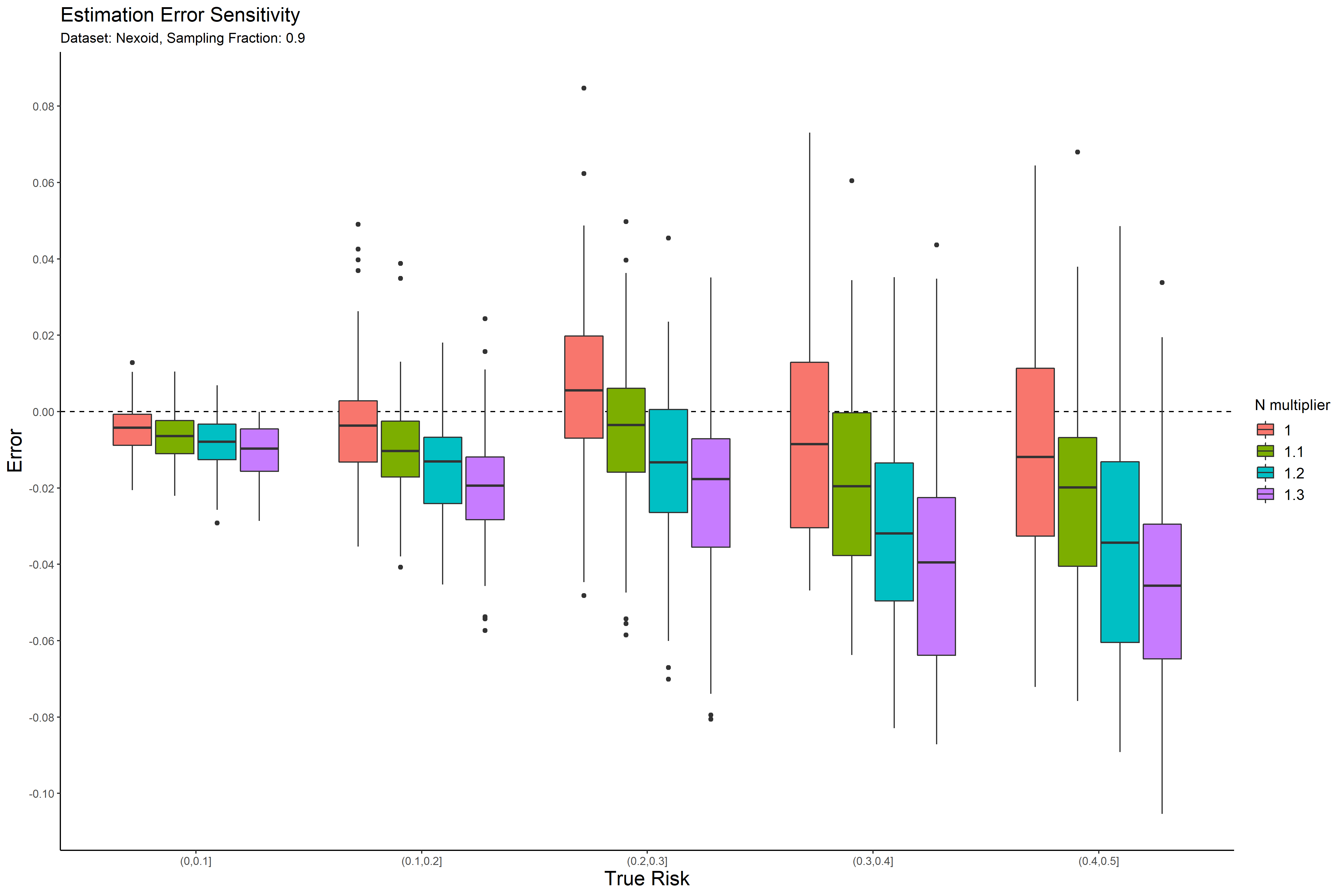

Supplement: S2 File — (ZIP) [file pone.0269097.s002.zip › nexoid/sensitivity.nexoid.18.png]

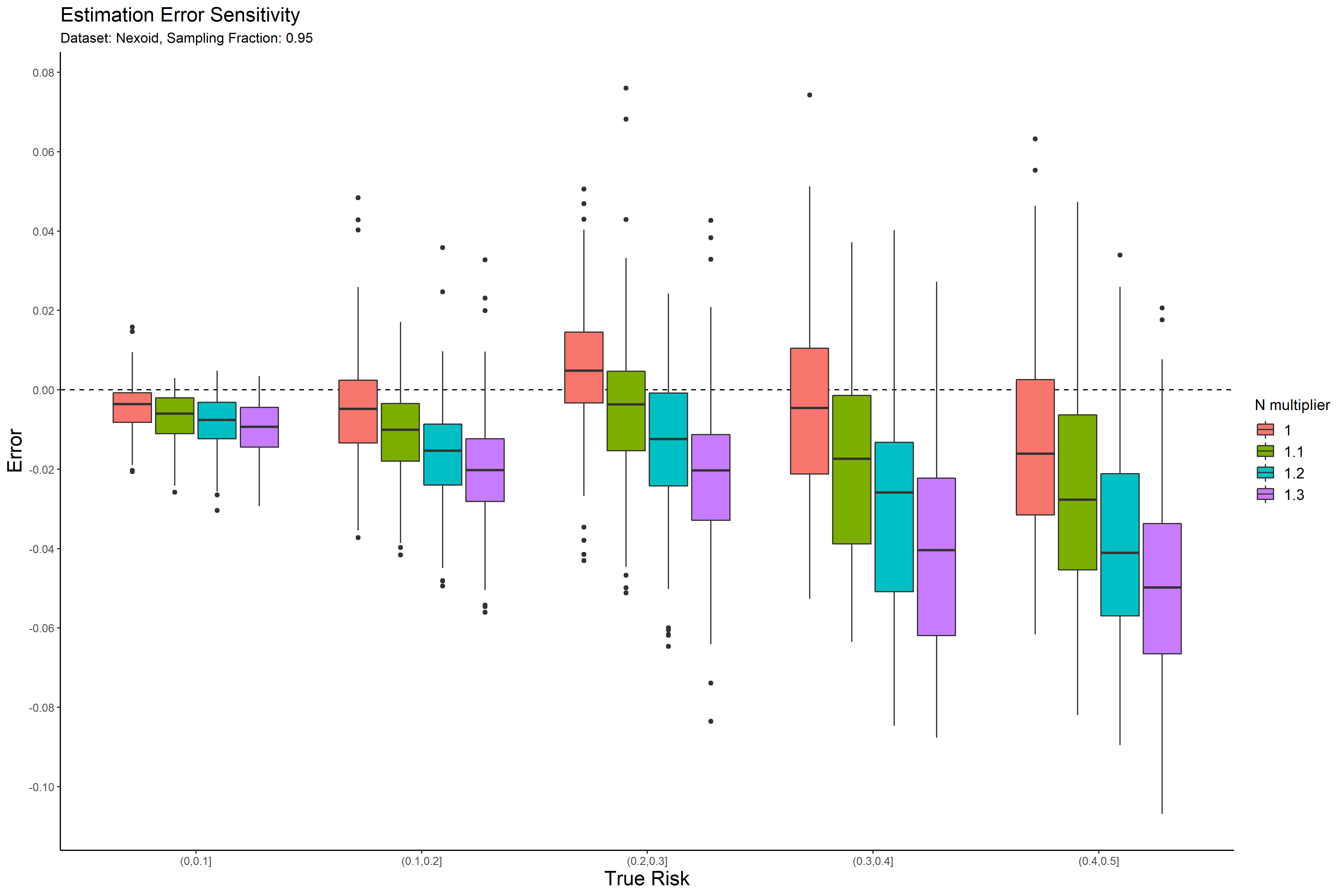

Supplement: S2 File — (ZIP) [file pone.0269097.s002.zip › nexoid/sensitivity.nexoid.19.png]

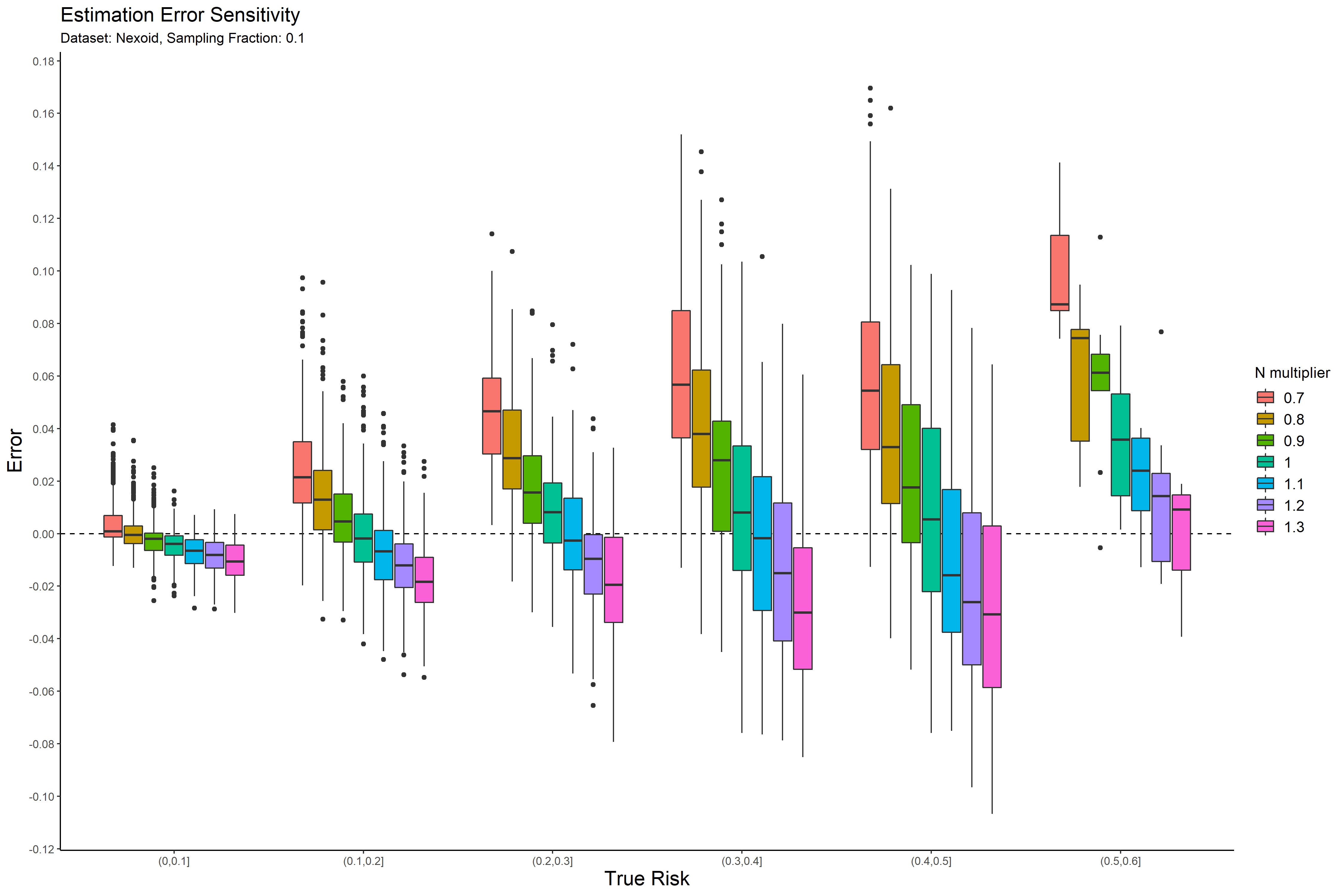

Supplement: S2 File — (ZIP) [file pone.0269097.s002.zip › nexoid/sensitivity.nexoid.2.png]

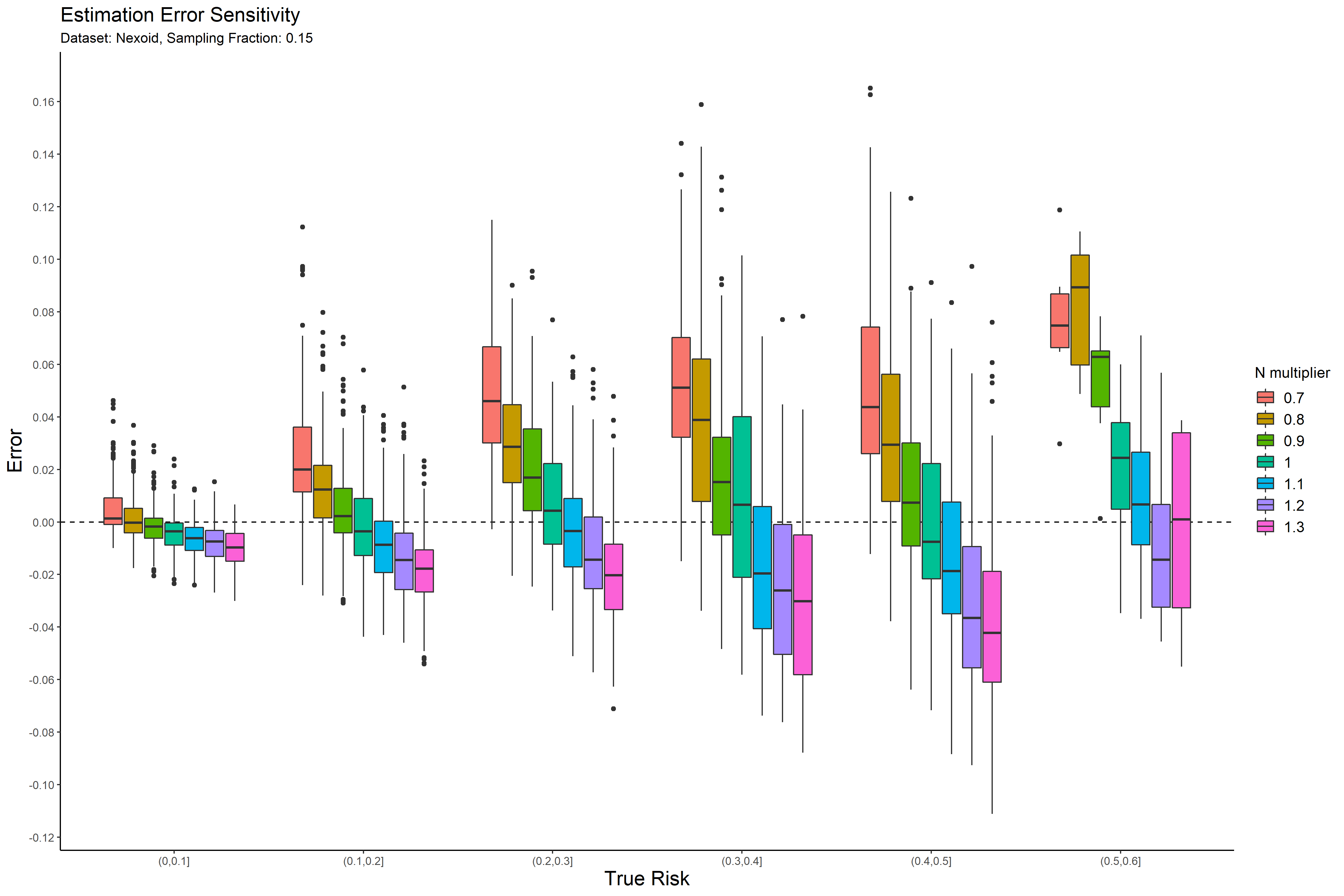

Supplement: S2 File — (ZIP) [file pone.0269097.s002.zip › nexoid/sensitivity.nexoid.3.png]

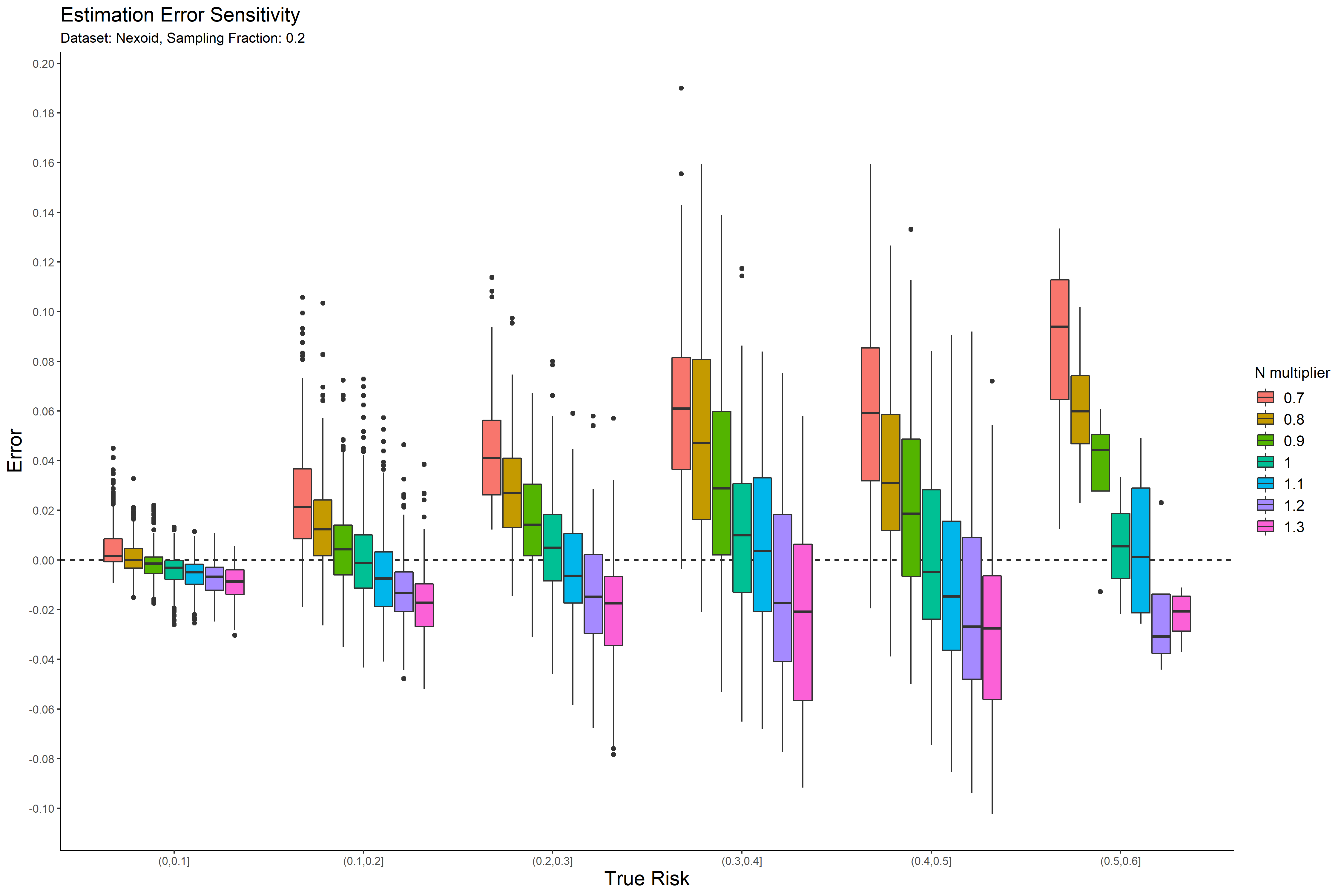

Supplement: S2 File — (ZIP) [file pone.0269097.s002.zip › nexoid/sensitivity.nexoid.4.png]

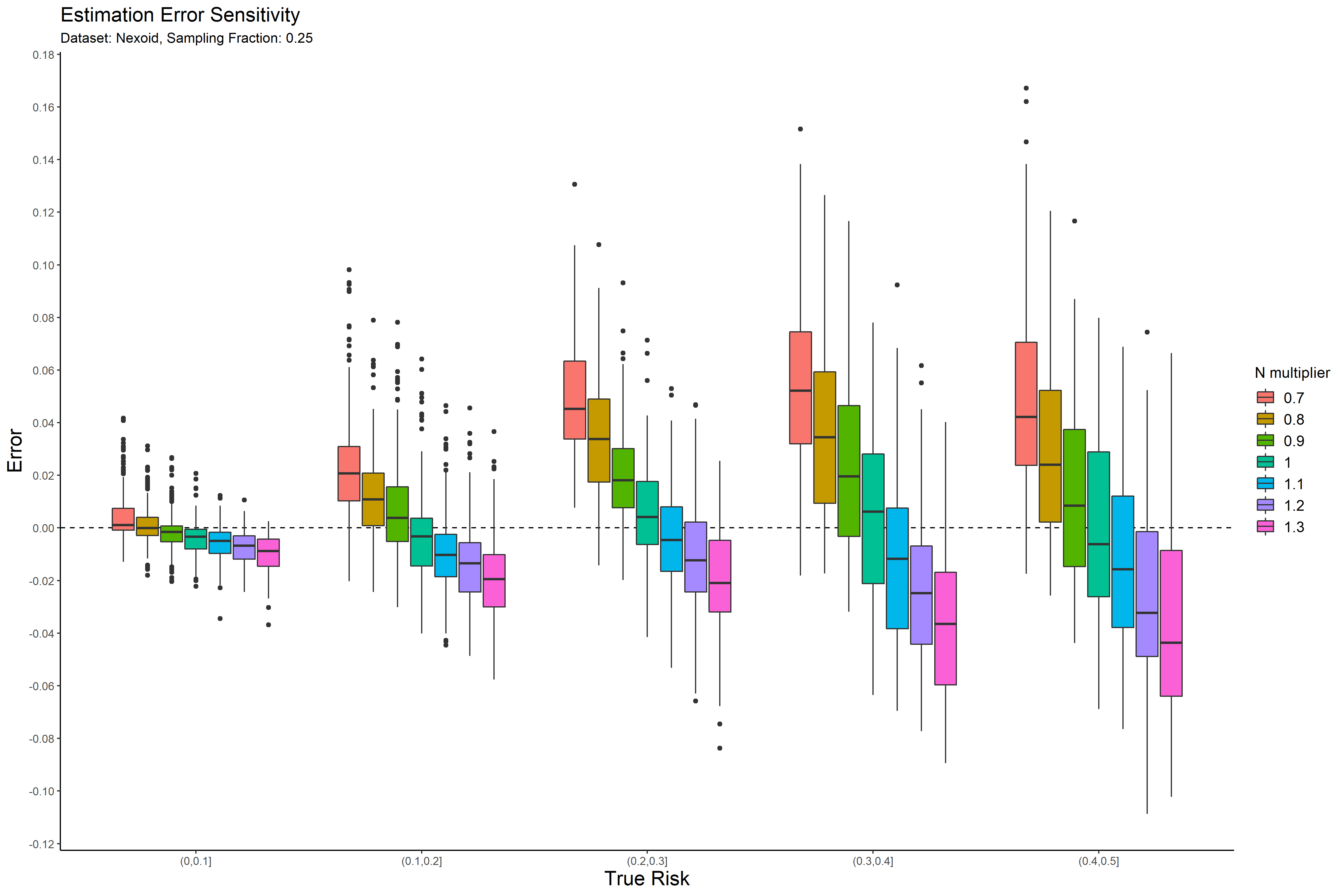

Supplement: S2 File — (ZIP) [file pone.0269097.s002.zip › nexoid/sensitivity.nexoid.5.png]

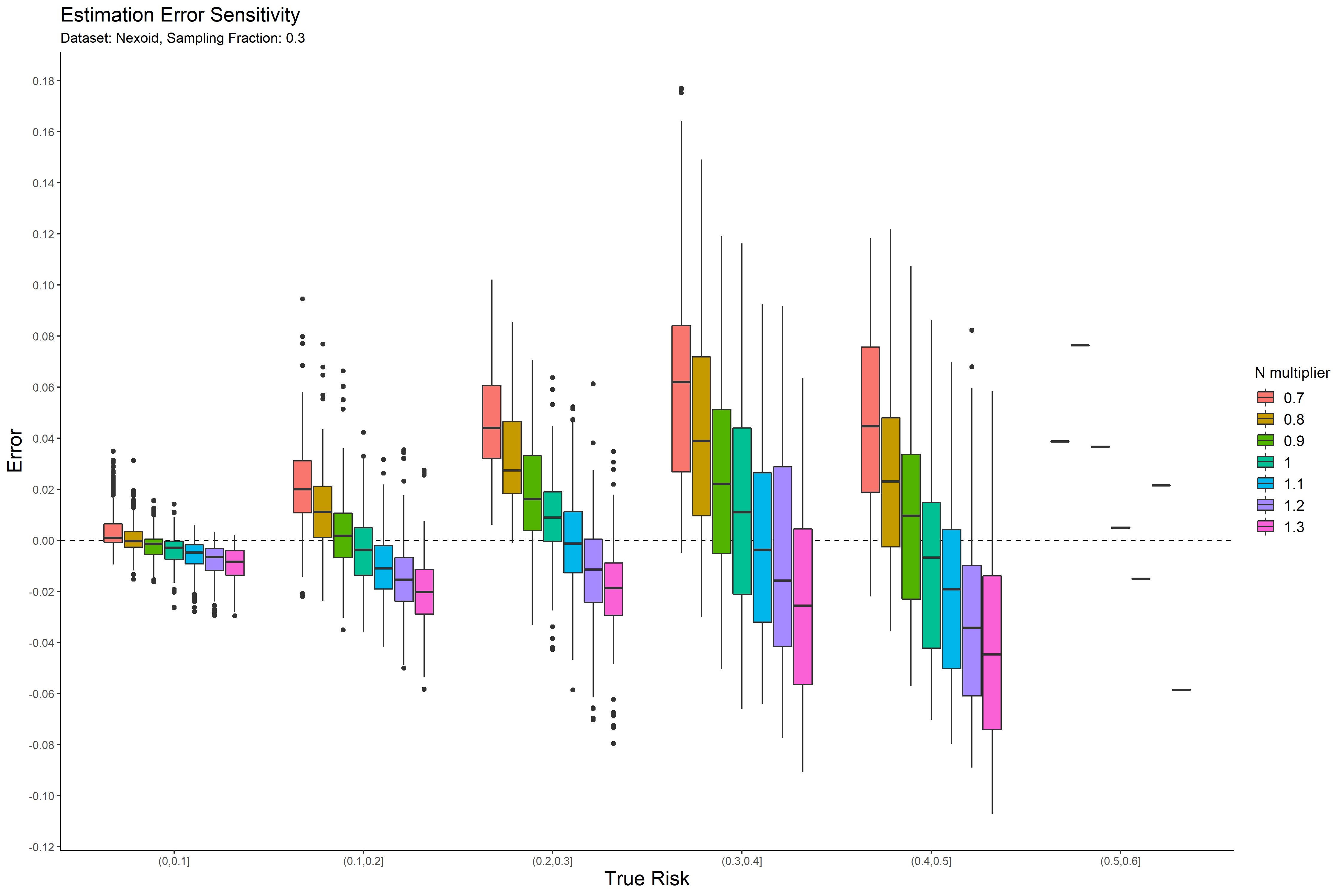

Supplement: S2 File — (ZIP) [file pone.0269097.s002.zip › nexoid/sensitivity.nexoid.6.png]

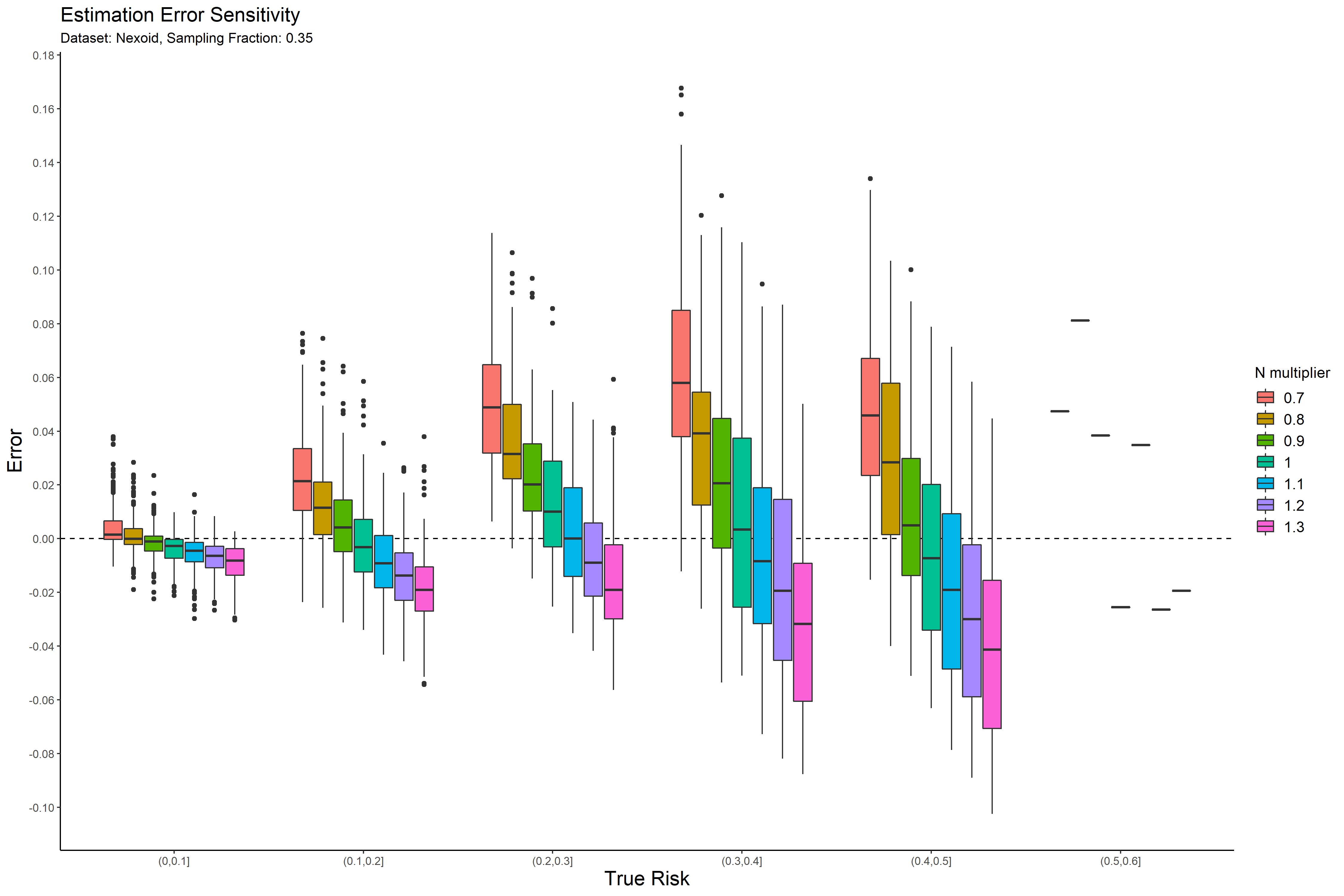

Supplement: S2 File — (ZIP) [file pone.0269097.s002.zip › nexoid/sensitivity.nexoid.7.png]

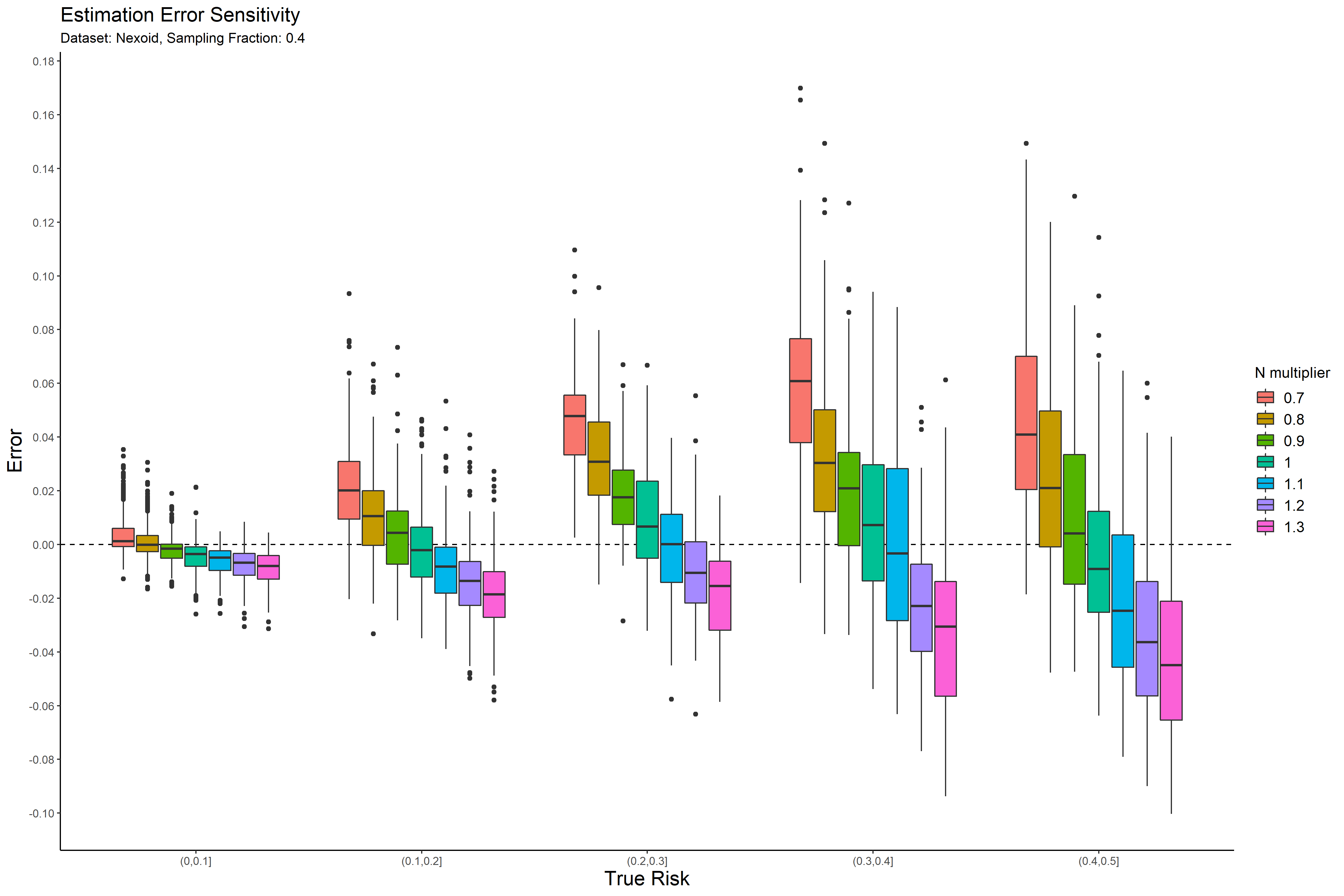

Supplement: S2 File — (ZIP) [file pone.0269097.s002.zip › nexoid/sensitivity.nexoid.8.png]

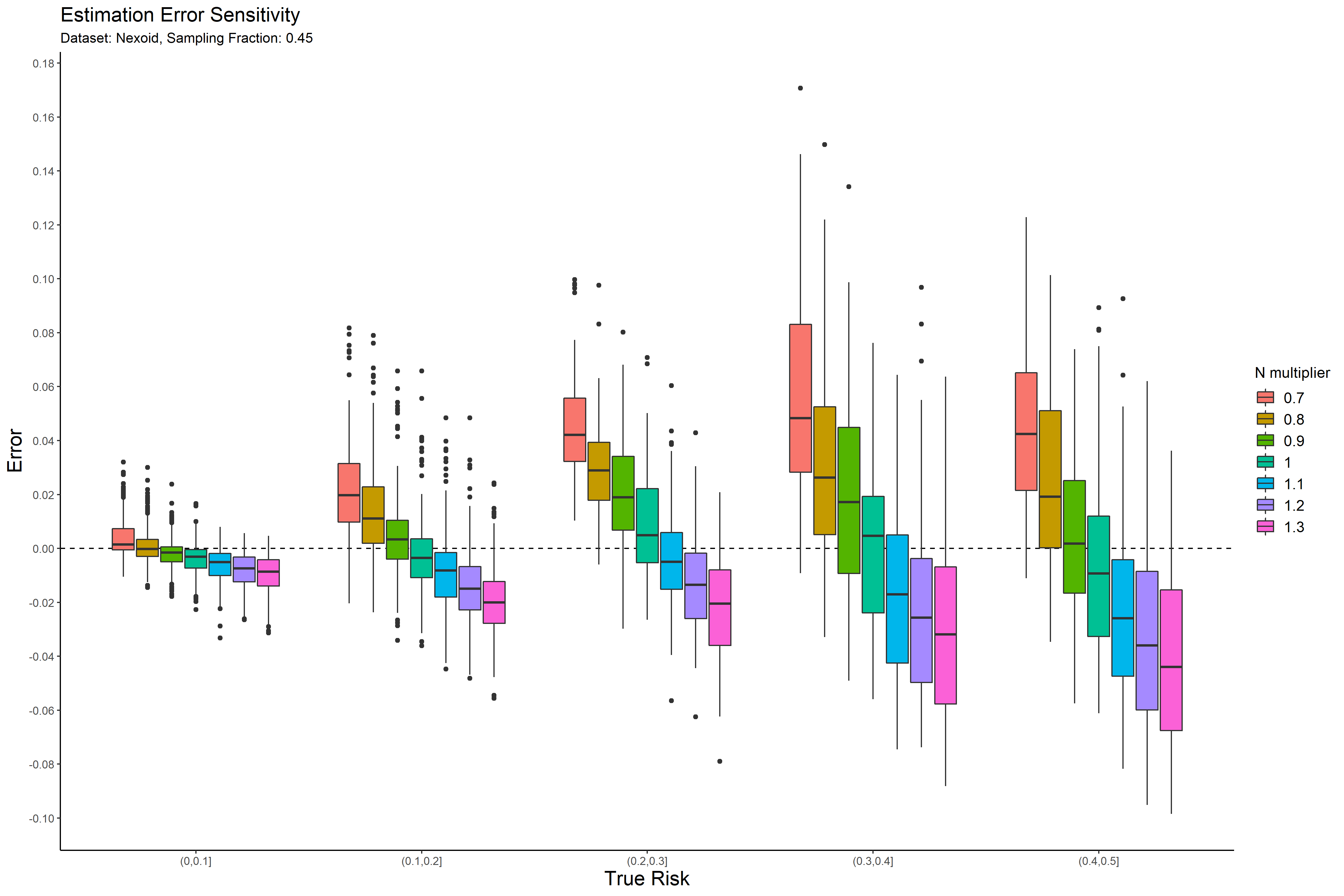

Supplement: S2 File — (ZIP) [file pone.0269097.s002.zip › nexoid/sensitivity.nexoid.9.png]

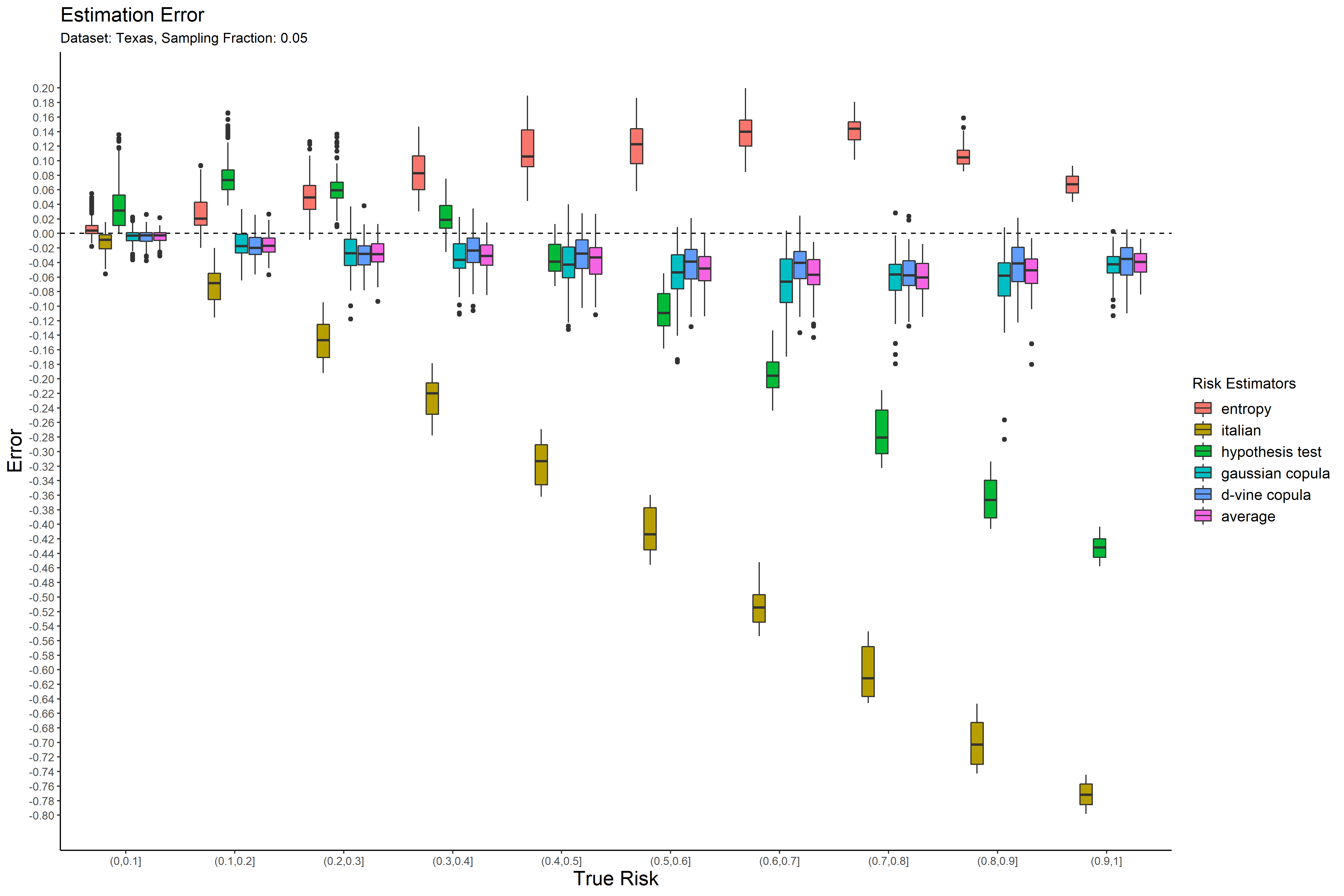

Supplement: S2 File — (ZIP) [file pone.0269097.s002.zip › tx/comparison.tx.1.png]

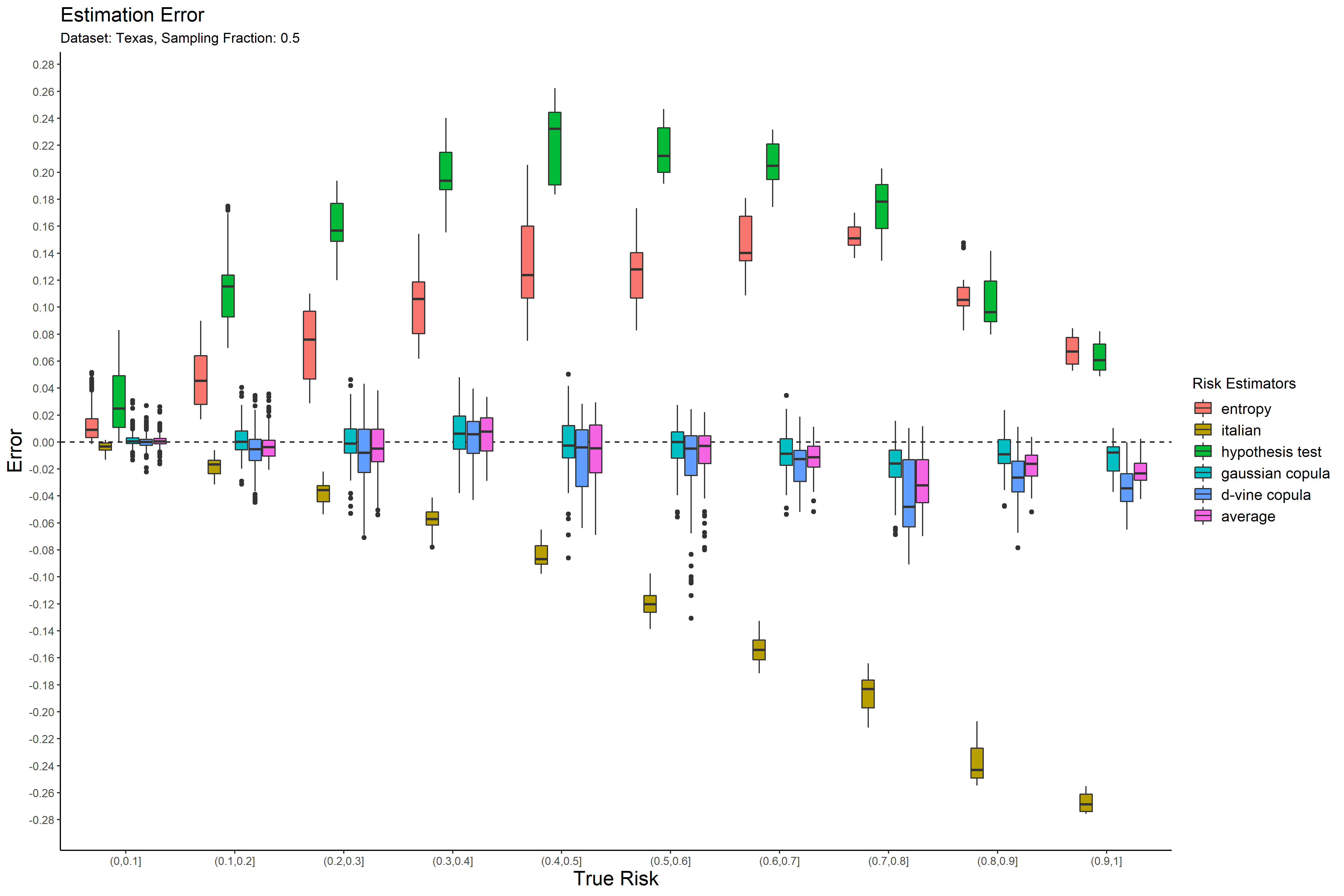

Supplement: S2 File — (ZIP) [file pone.0269097.s002.zip › tx/comparison.tx.10.png]

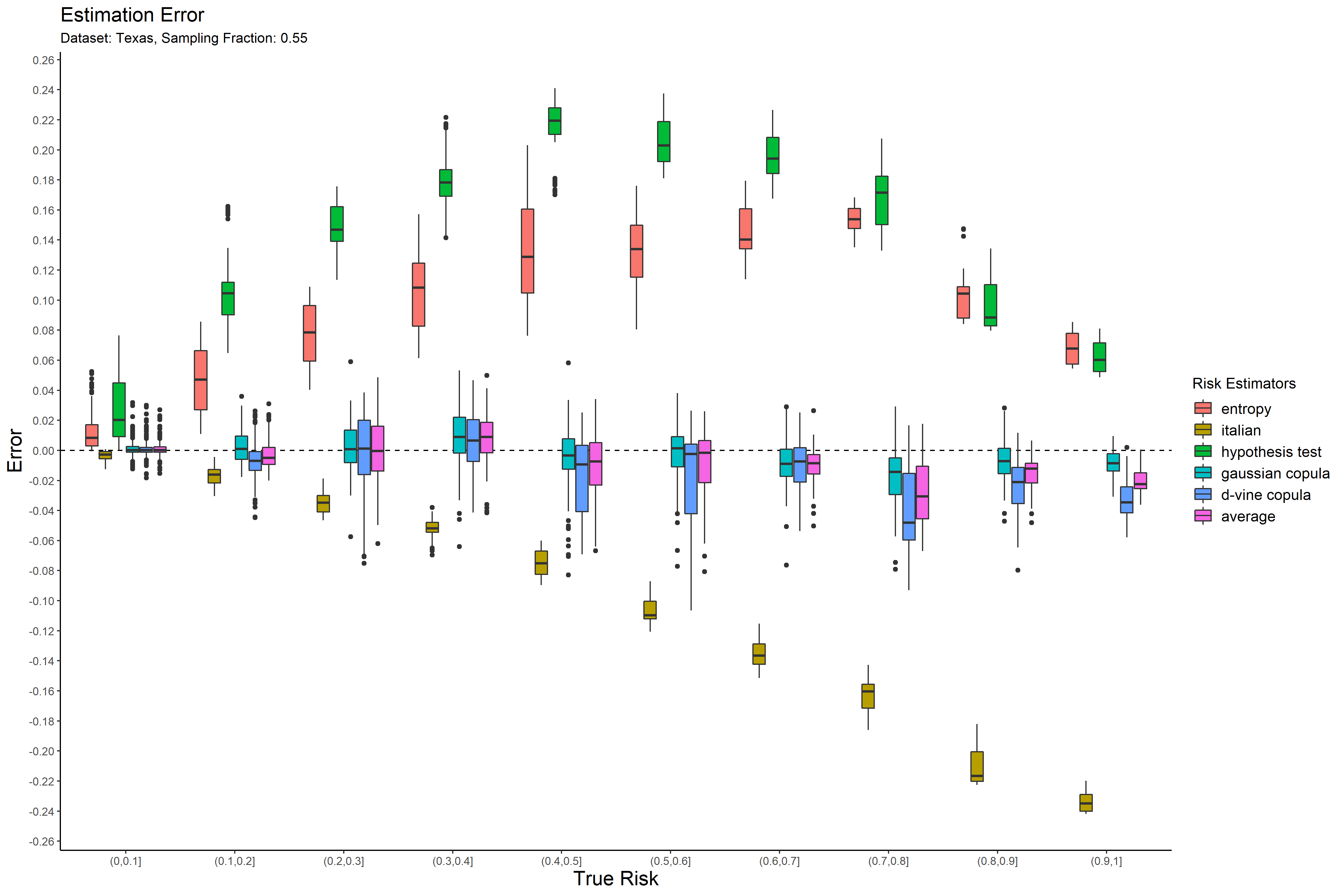

Supplement: S2 File — (ZIP) [file pone.0269097.s002.zip › tx/comparison.tx.11.png]

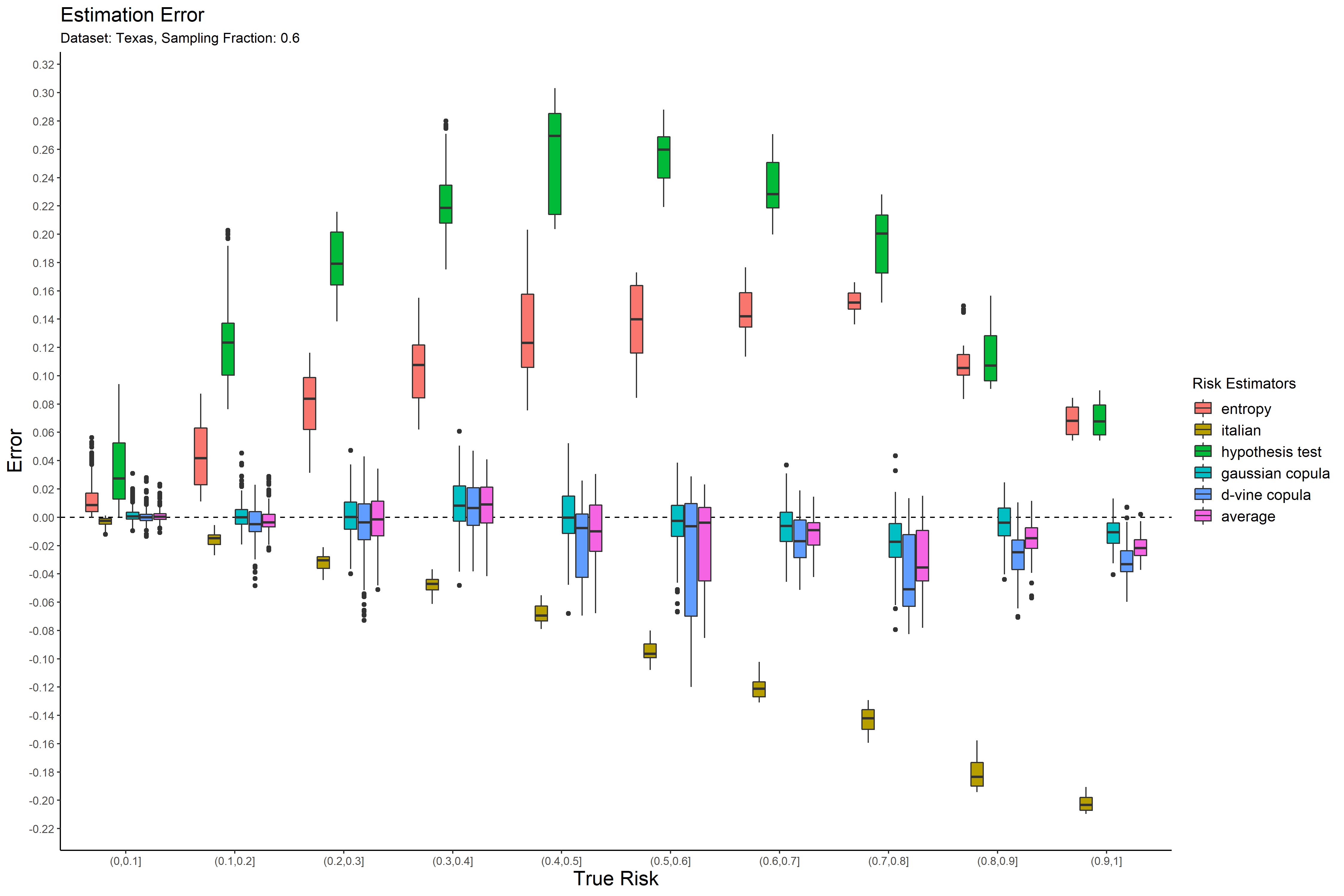

Supplement: S2 File — (ZIP) [file pone.0269097.s002.zip › tx/comparison.tx.12.png]

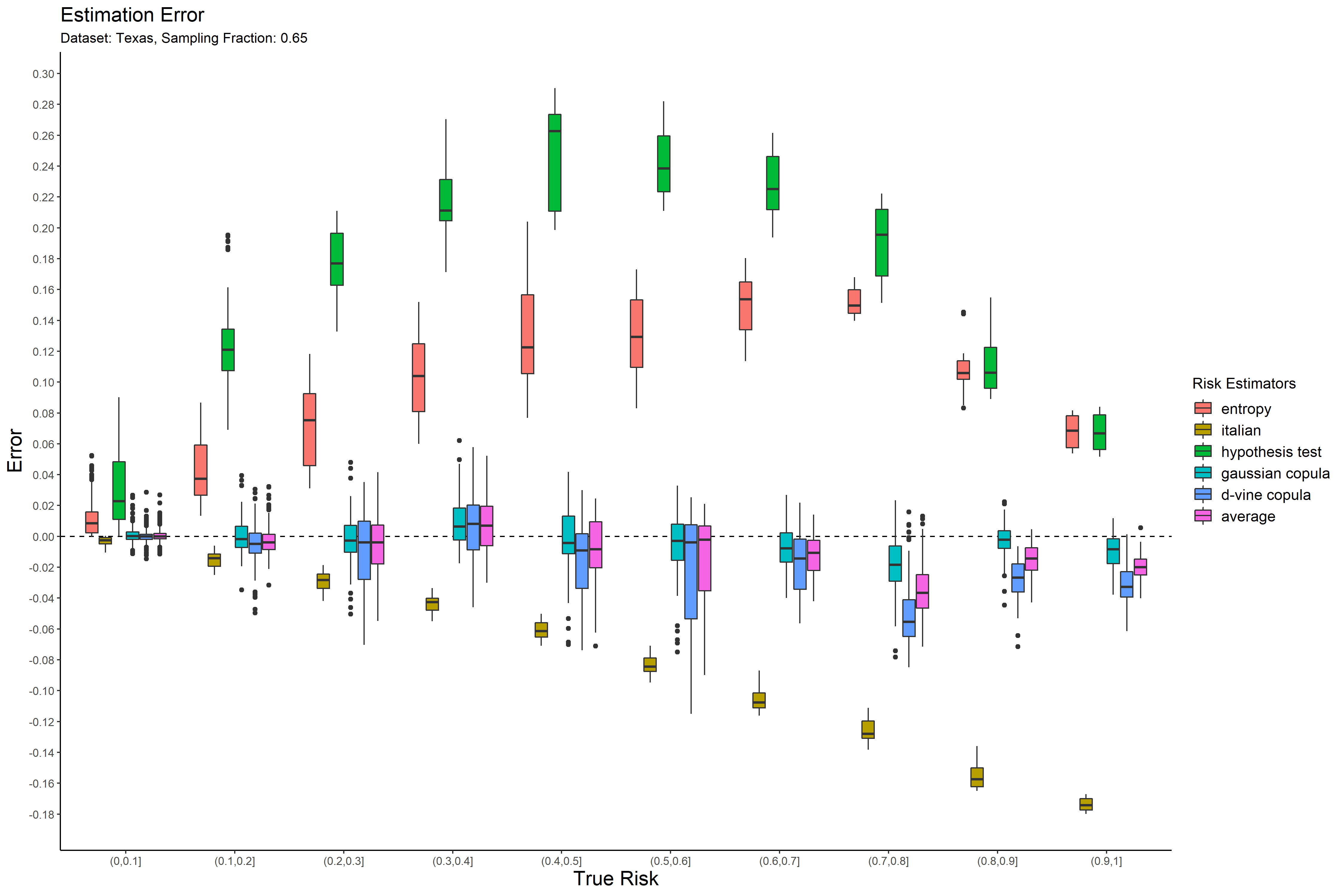

Supplement: S2 File — (ZIP) [file pone.0269097.s002.zip › tx/comparison.tx.13.png]

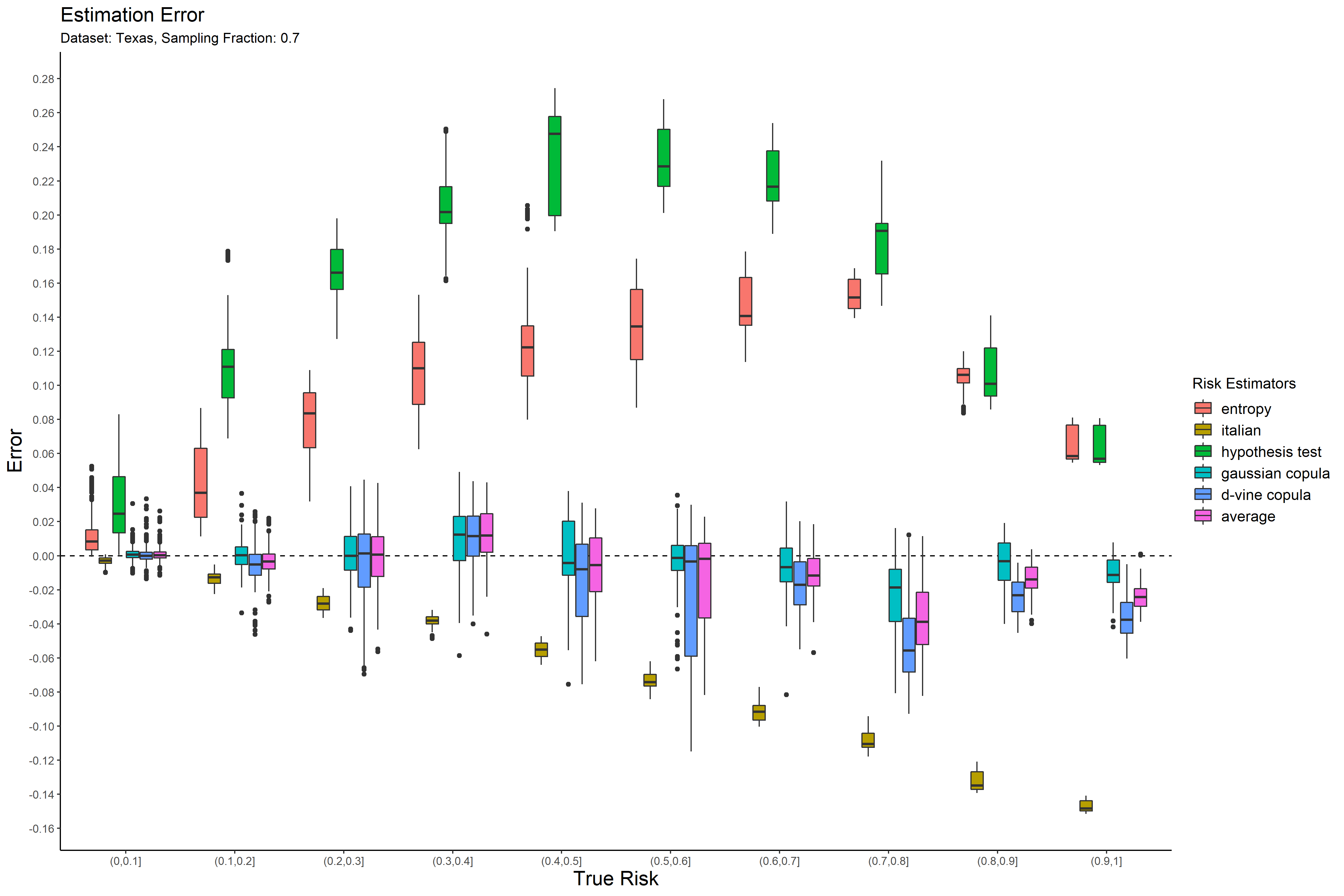

Supplement: S2 File — (ZIP) [file pone.0269097.s002.zip › tx/comparison.tx.14.png]

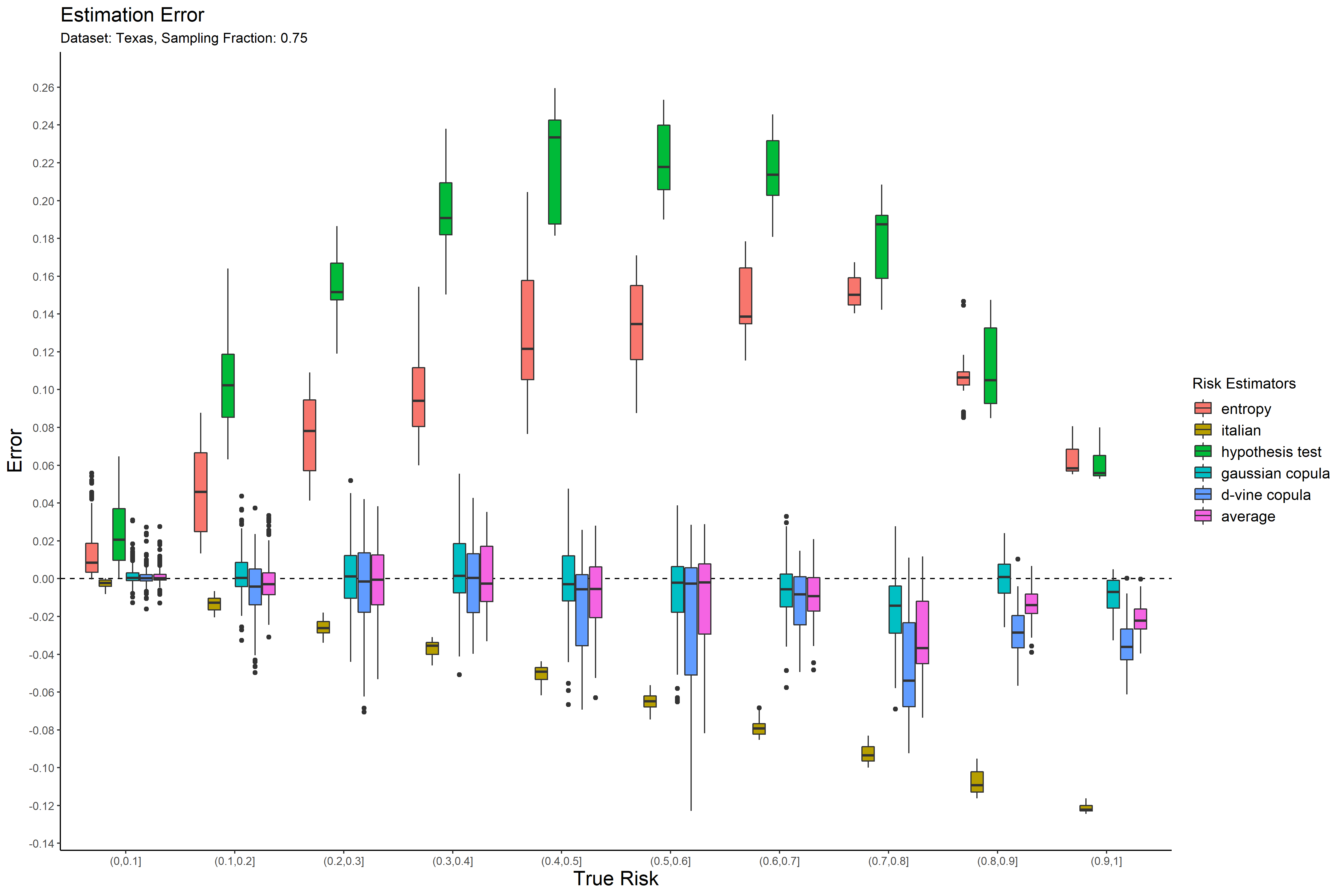

Supplement: S2 File — (ZIP) [file pone.0269097.s002.zip › tx/comparison.tx.15.png]

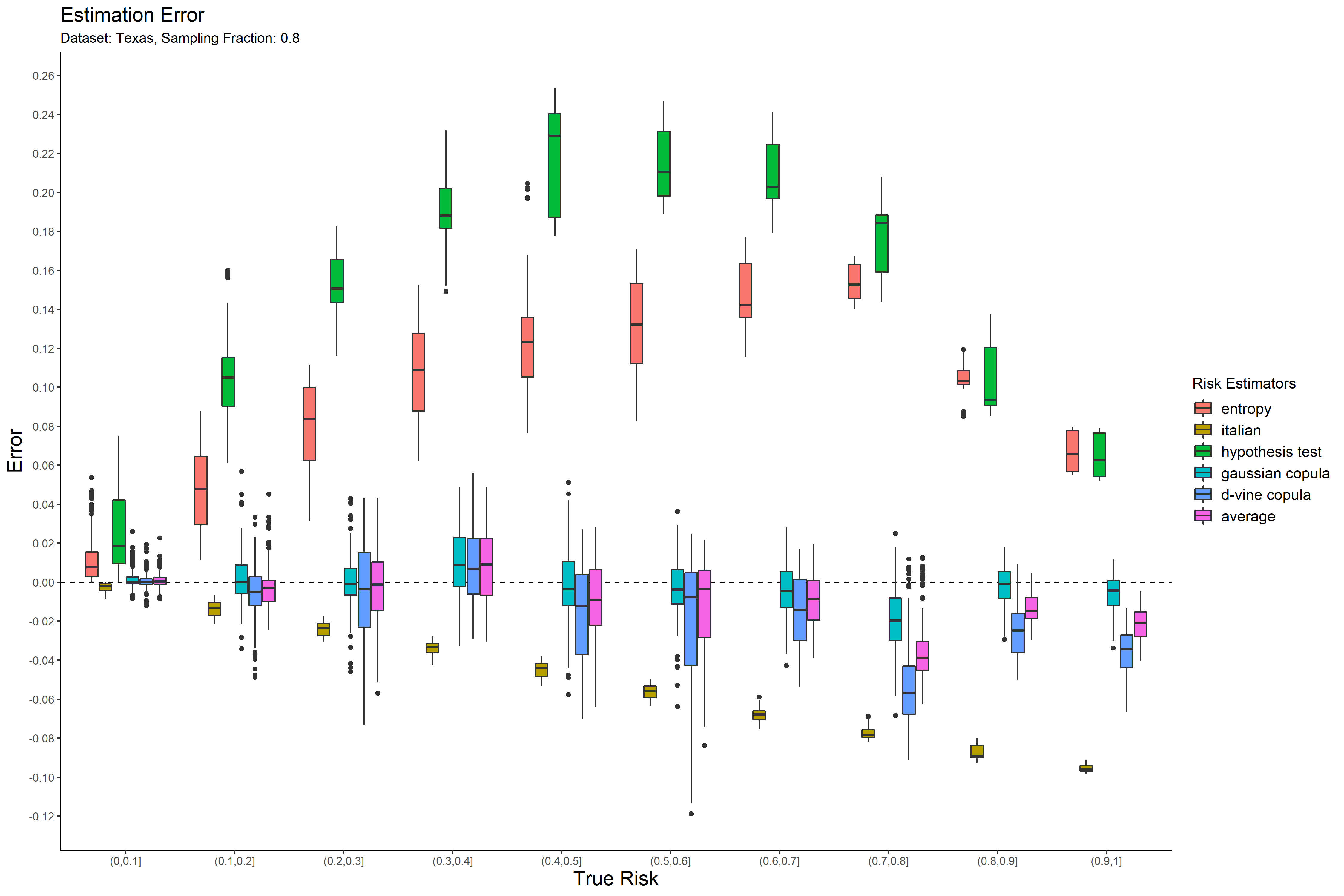

Supplement: S2 File — (ZIP) [file pone.0269097.s002.zip › tx/comparison.tx.16.png]

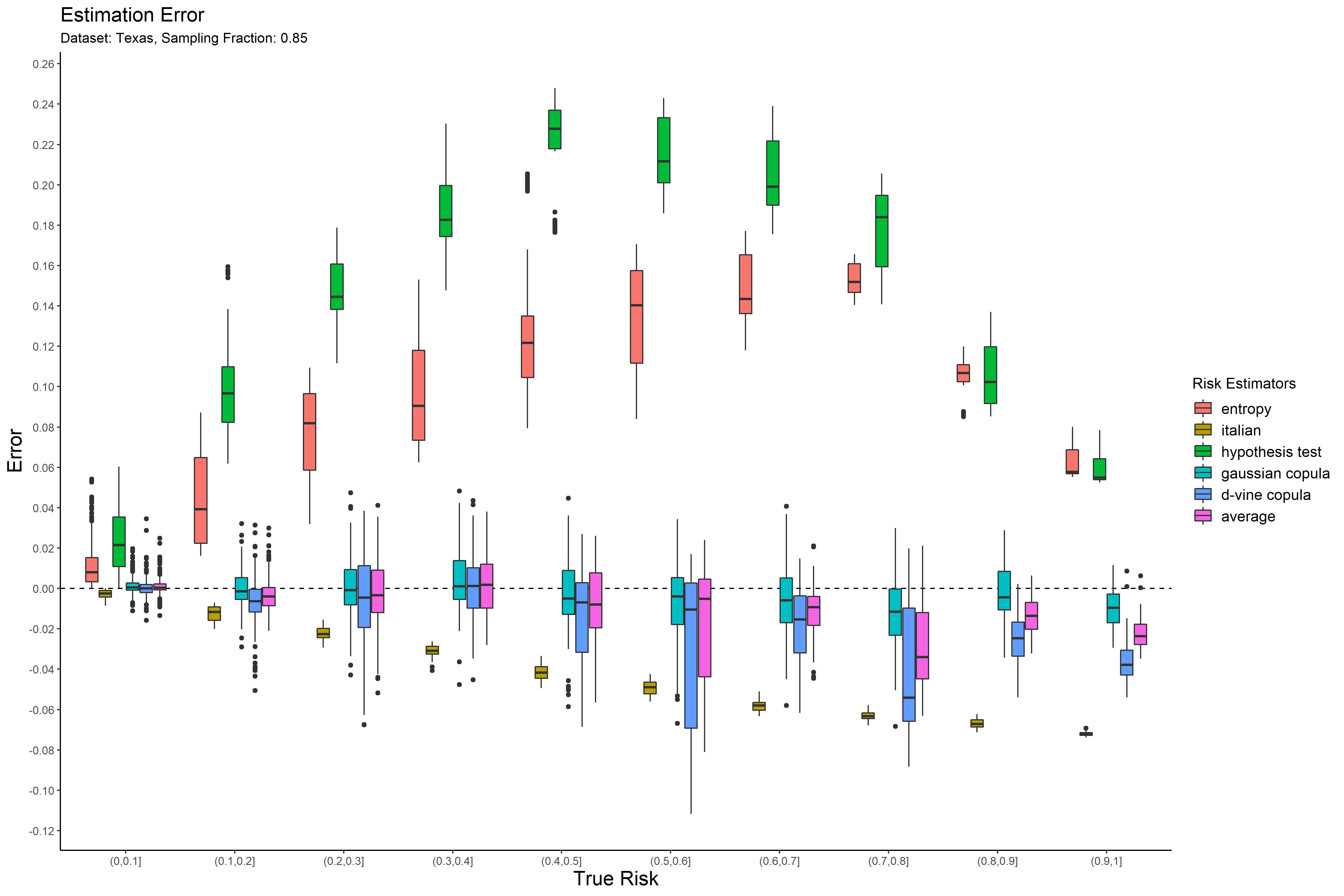

Supplement: S2 File — (ZIP) [file pone.0269097.s002.zip › tx/comparison.tx.17.png]

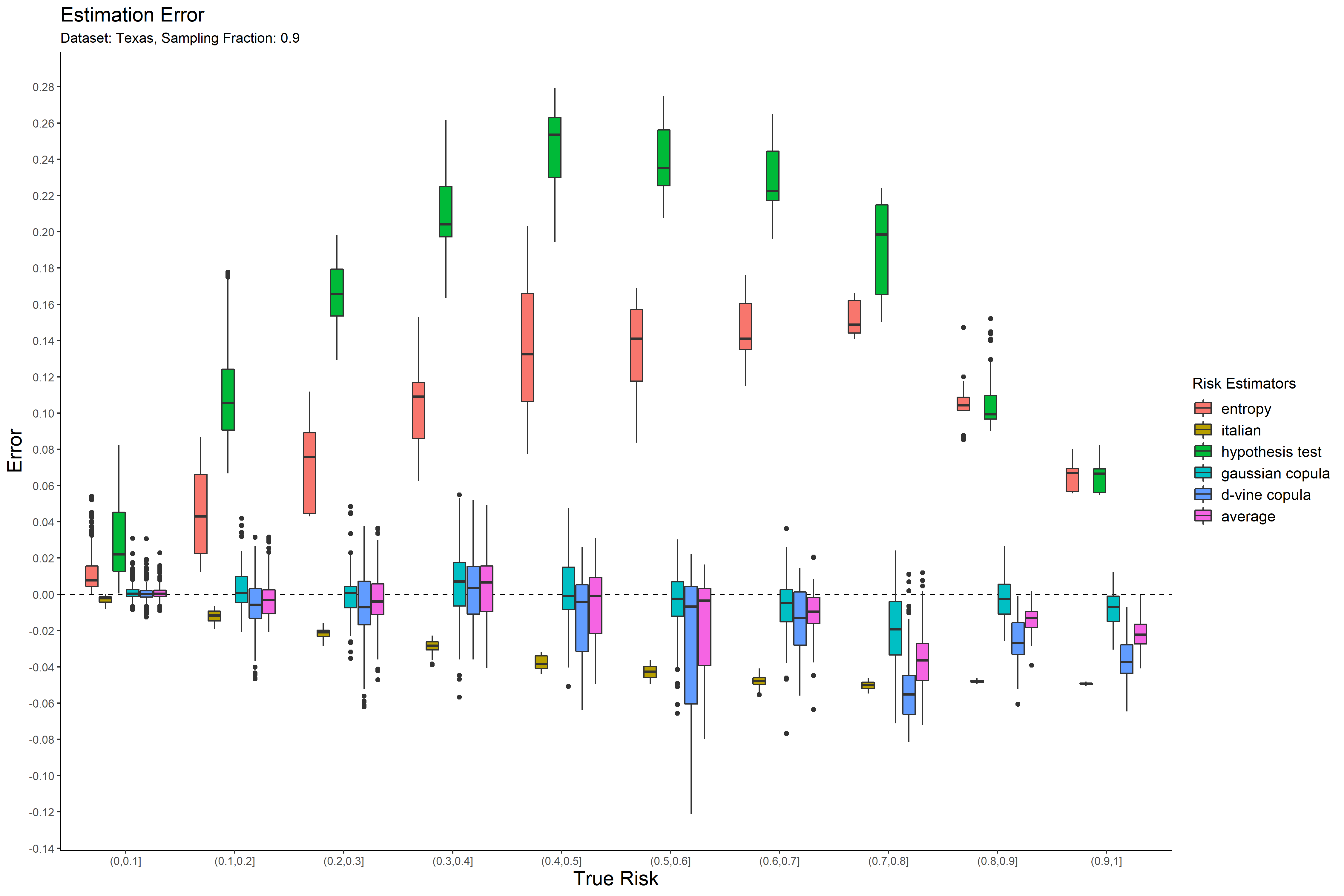

Supplement: S2 File — (ZIP) [file pone.0269097.s002.zip › tx/comparison.tx.18.png]

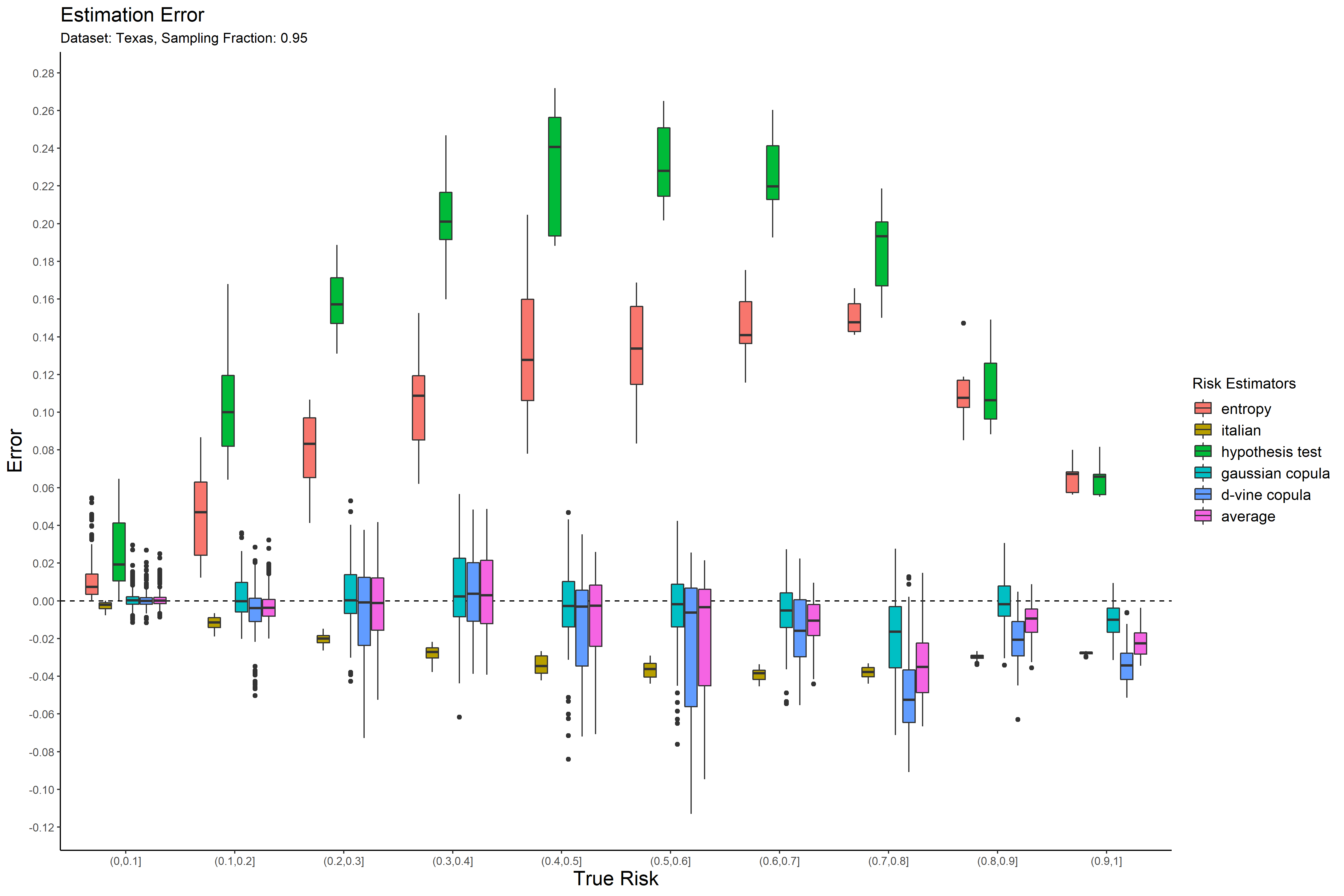

Supplement: S2 File — (ZIP) [file pone.0269097.s002.zip › tx/comparison.tx.19.png]

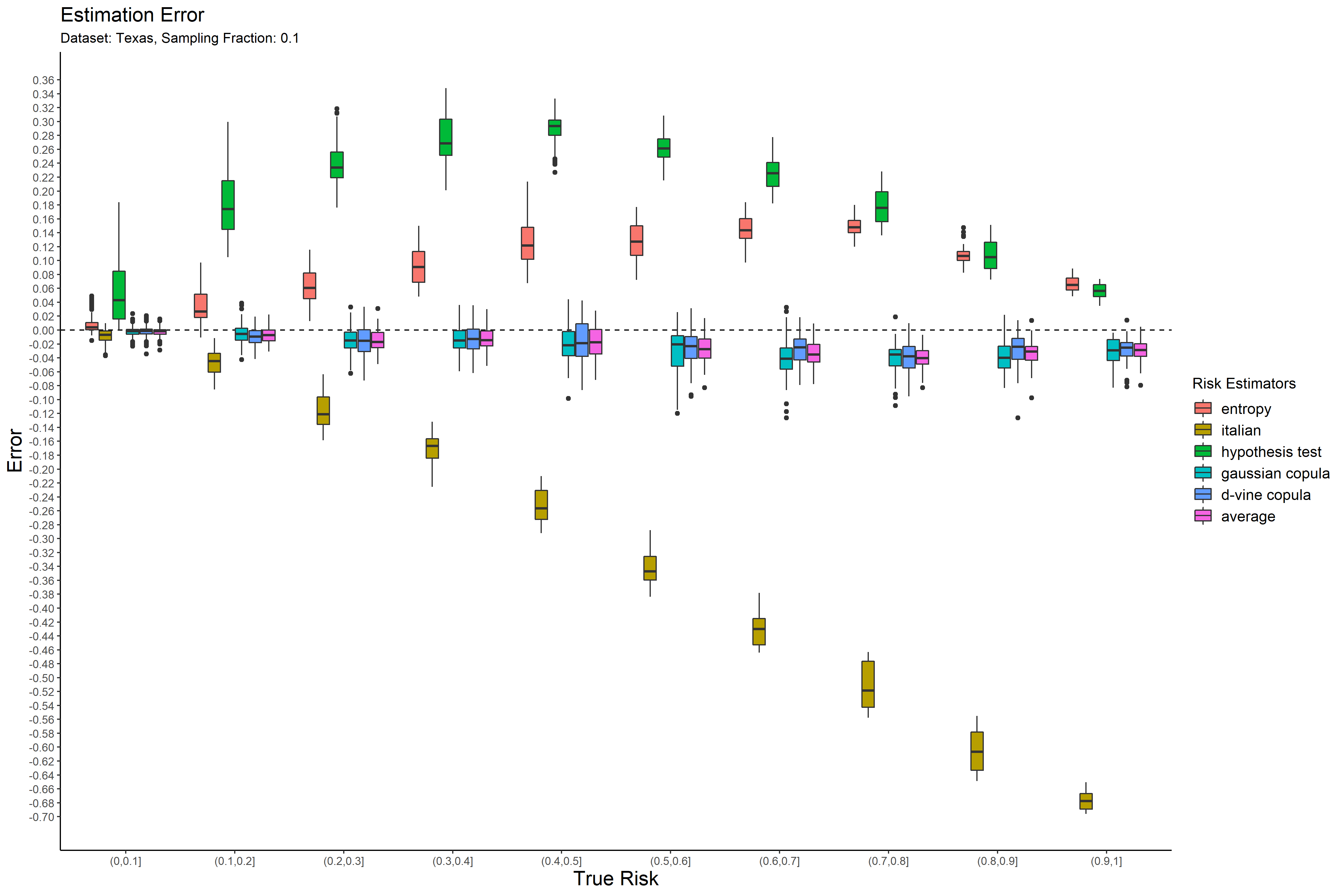

Supplement: S2 File — (ZIP) [file pone.0269097.s002.zip › tx/comparison.tx.2.png]

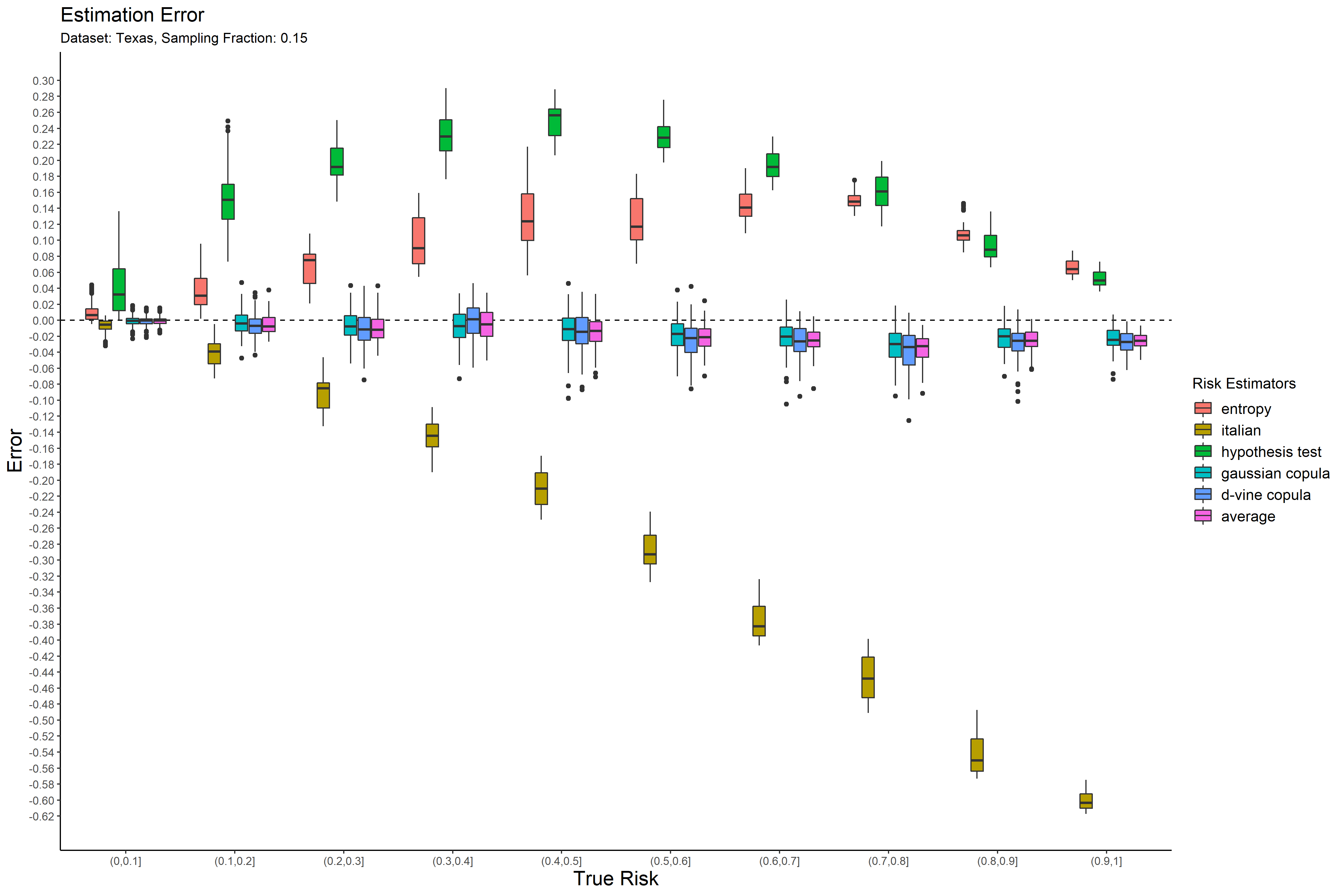

Supplement: S2 File — (ZIP) [file pone.0269097.s002.zip › tx/comparison.tx.3.png]

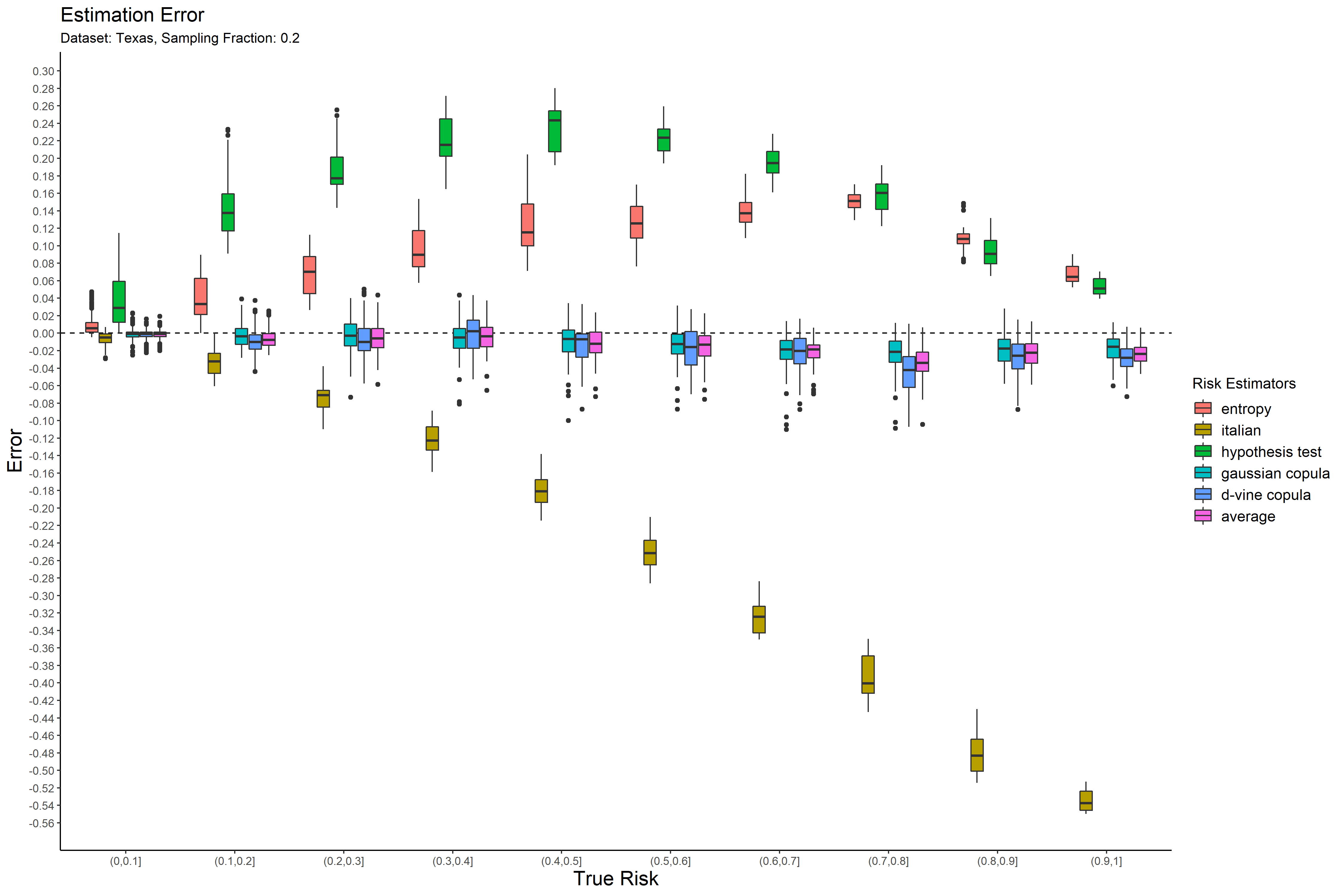

Supplement: S2 File — (ZIP) [file pone.0269097.s002.zip › tx/comparison.tx.4.png]

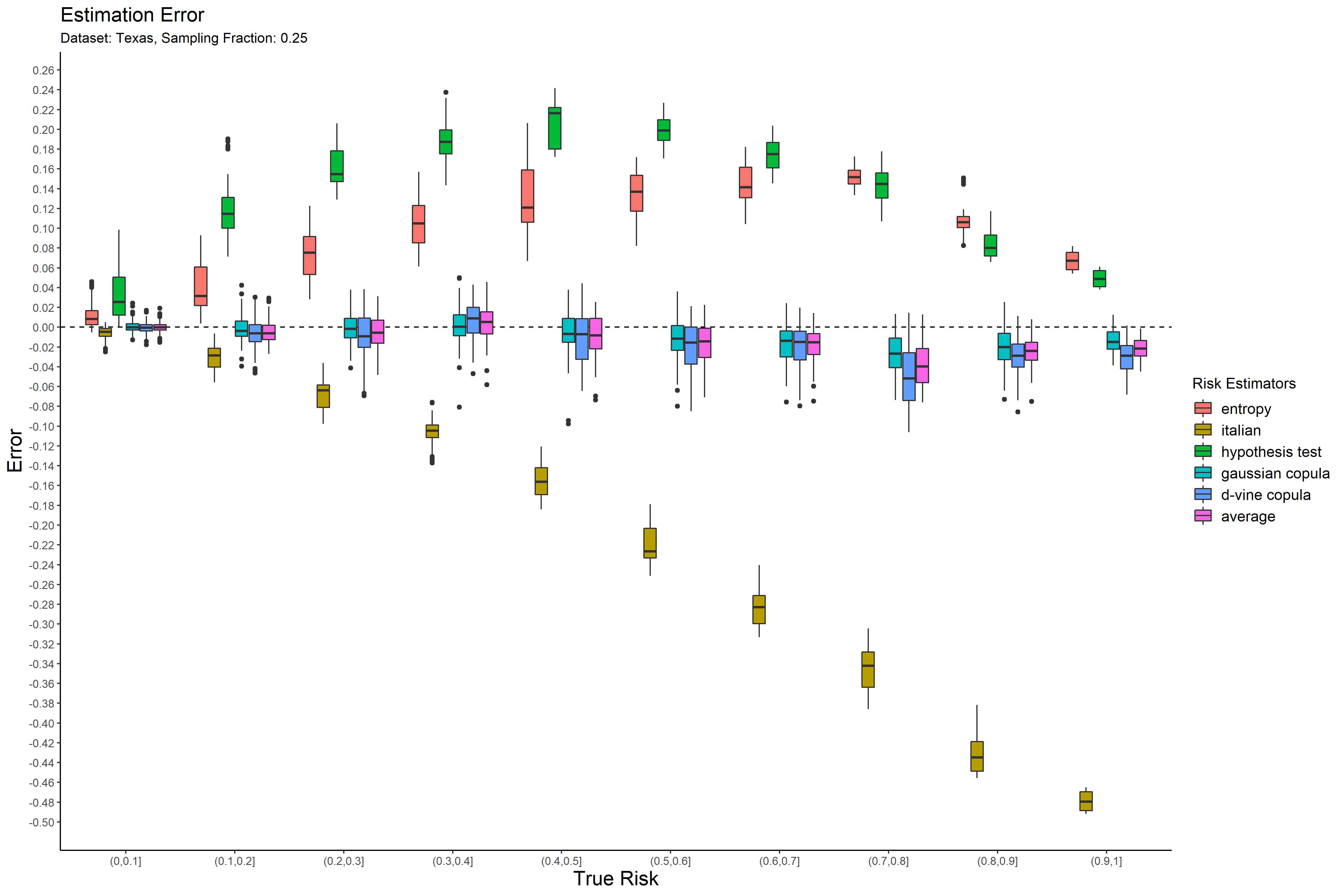

Supplement: S2 File — (ZIP) [file pone.0269097.s002.zip › tx/comparison.tx.5.png]

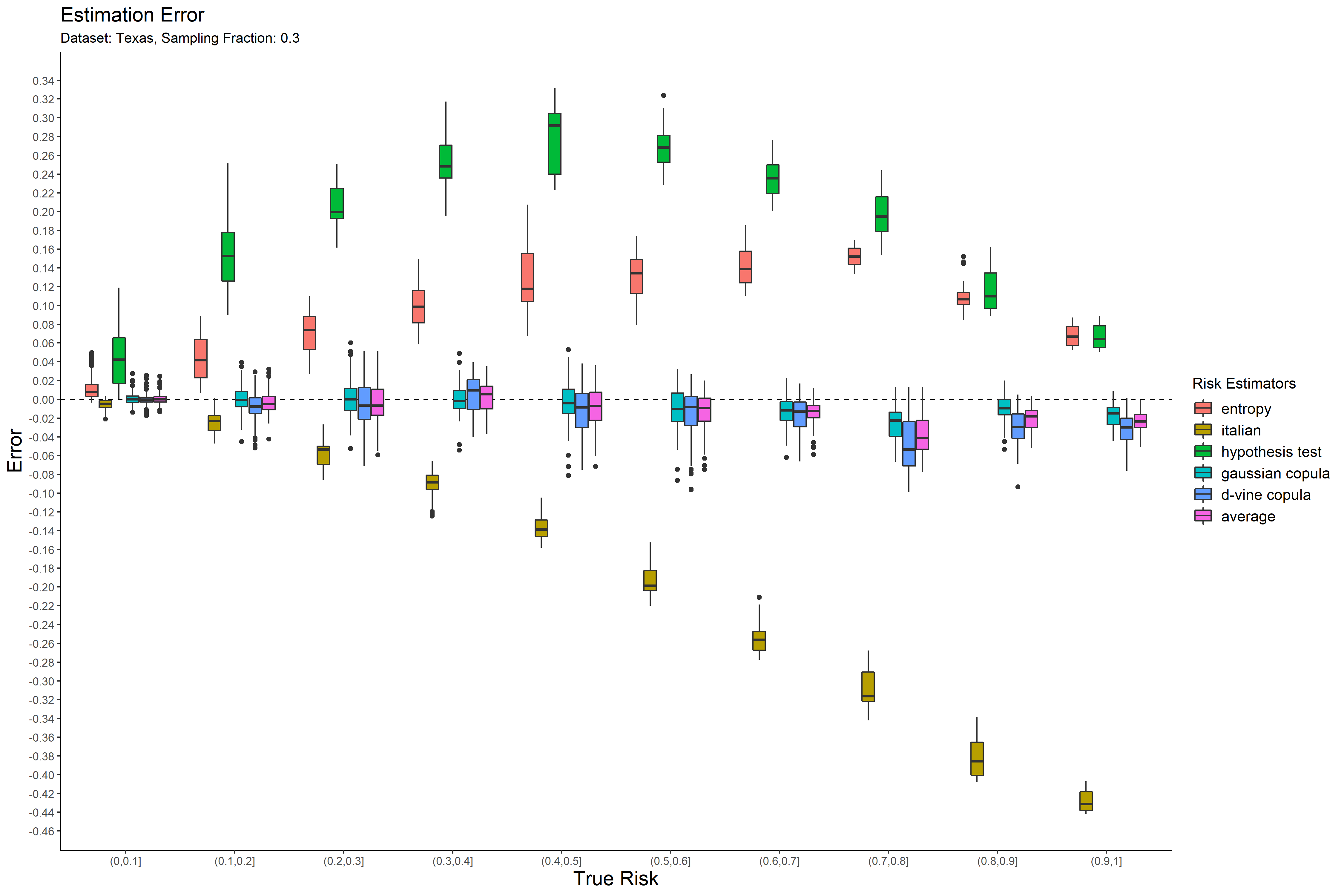

Supplement: S2 File — (ZIP) [file pone.0269097.s002.zip › tx/comparison.tx.6.png]

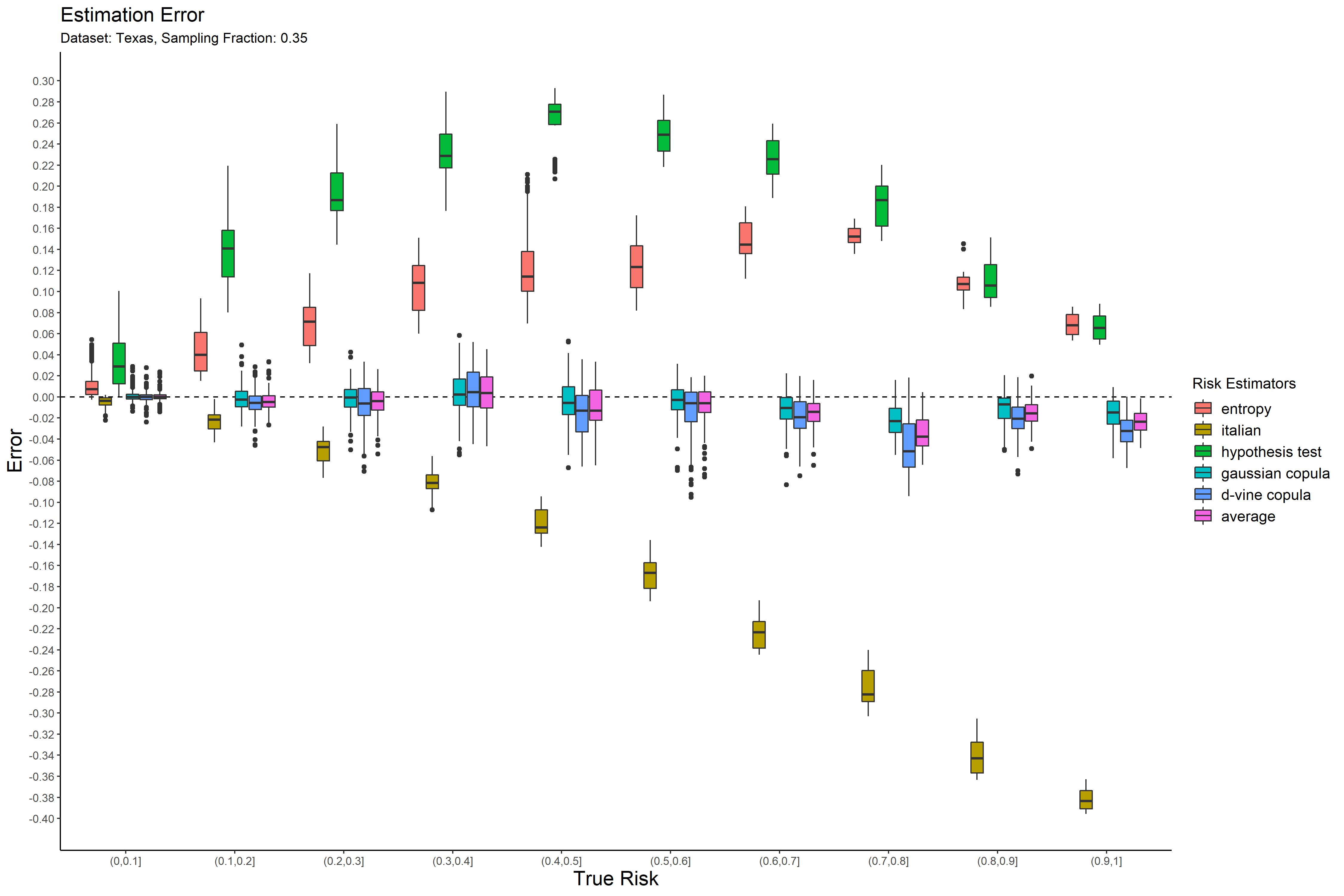

Supplement: S2 File — (ZIP) [file pone.0269097.s002.zip › tx/comparison.tx.7.png]

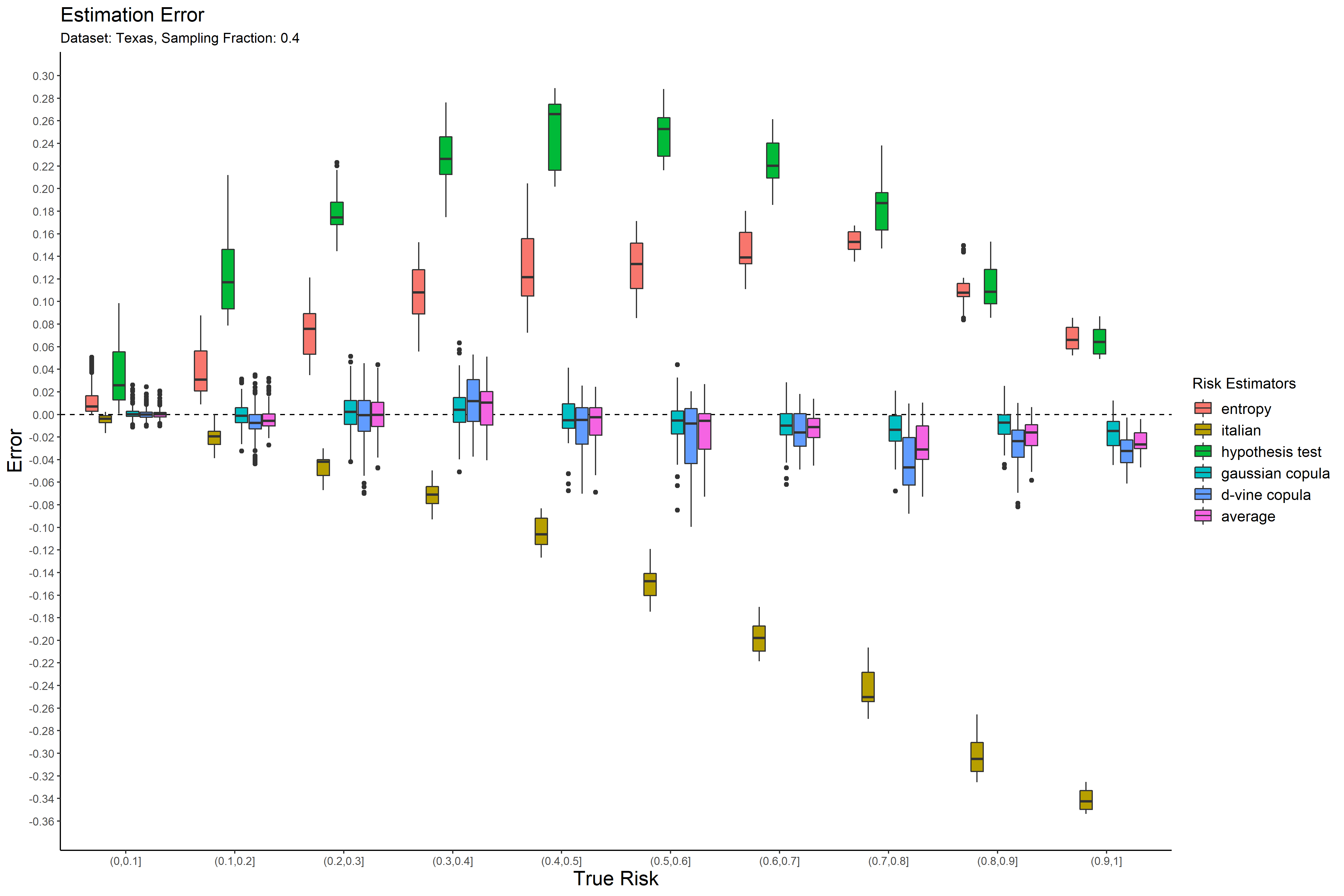

Supplement: S2 File — (ZIP) [file pone.0269097.s002.zip › tx/comparison.tx.8.png]

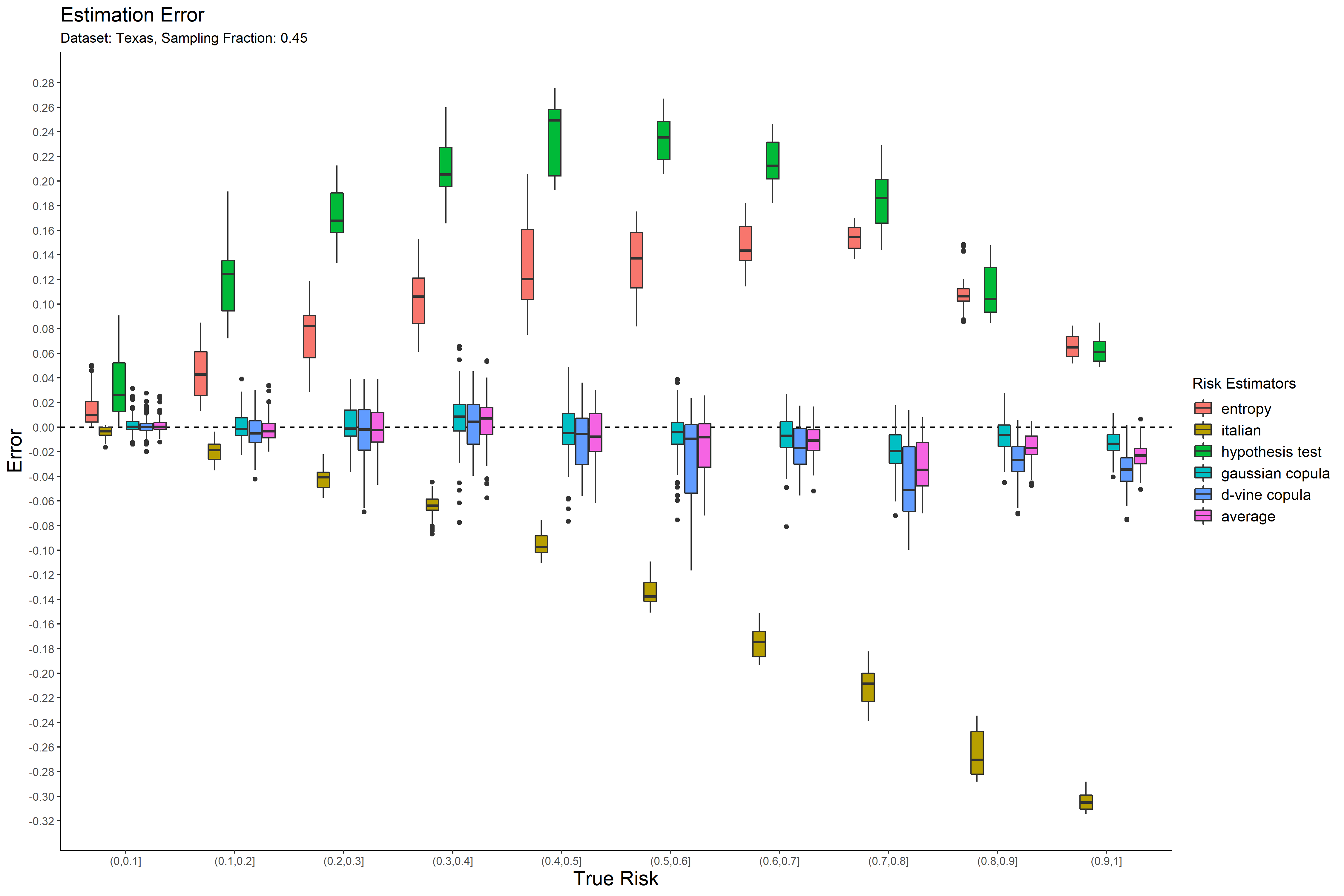

Supplement: S2 File — (ZIP) [file pone.0269097.s002.zip › tx/comparison.tx.9.png]

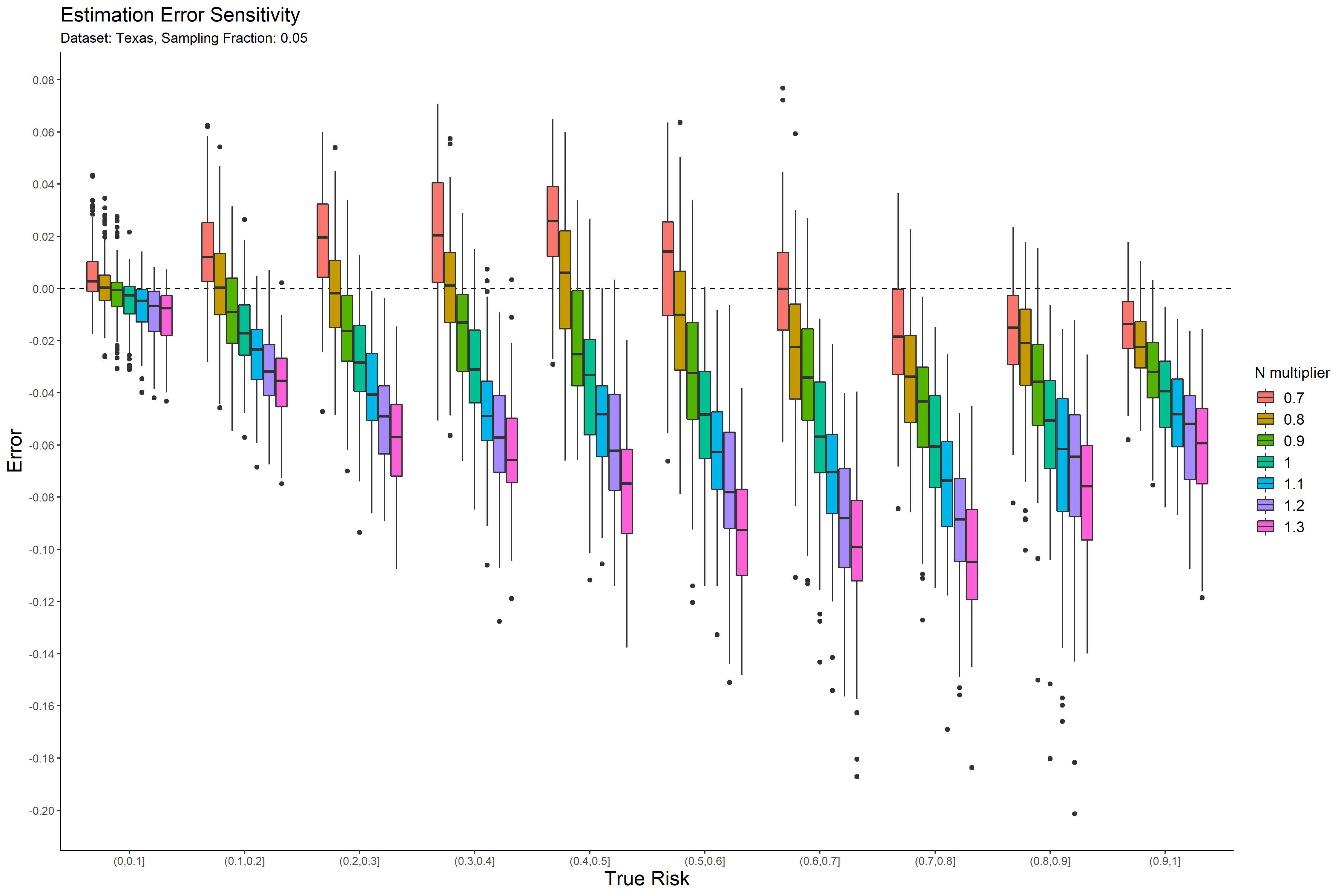

Supplement: S2 File — (ZIP) [file pone.0269097.s002.zip › tx/sensitivity.tx.1.png]

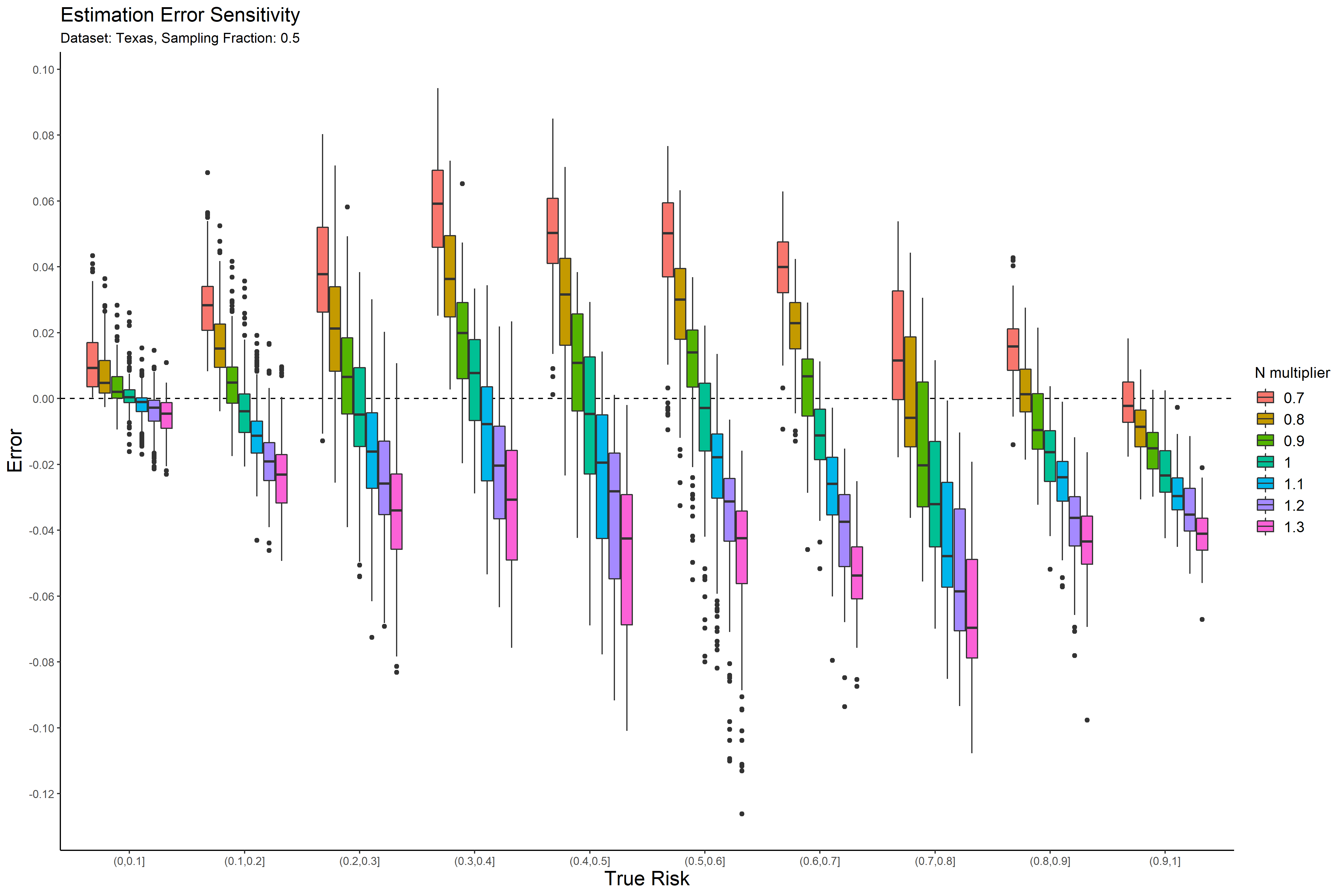

Supplement: S2 File — (ZIP) [file pone.0269097.s002.zip › tx/sensitivity.tx.10.png]

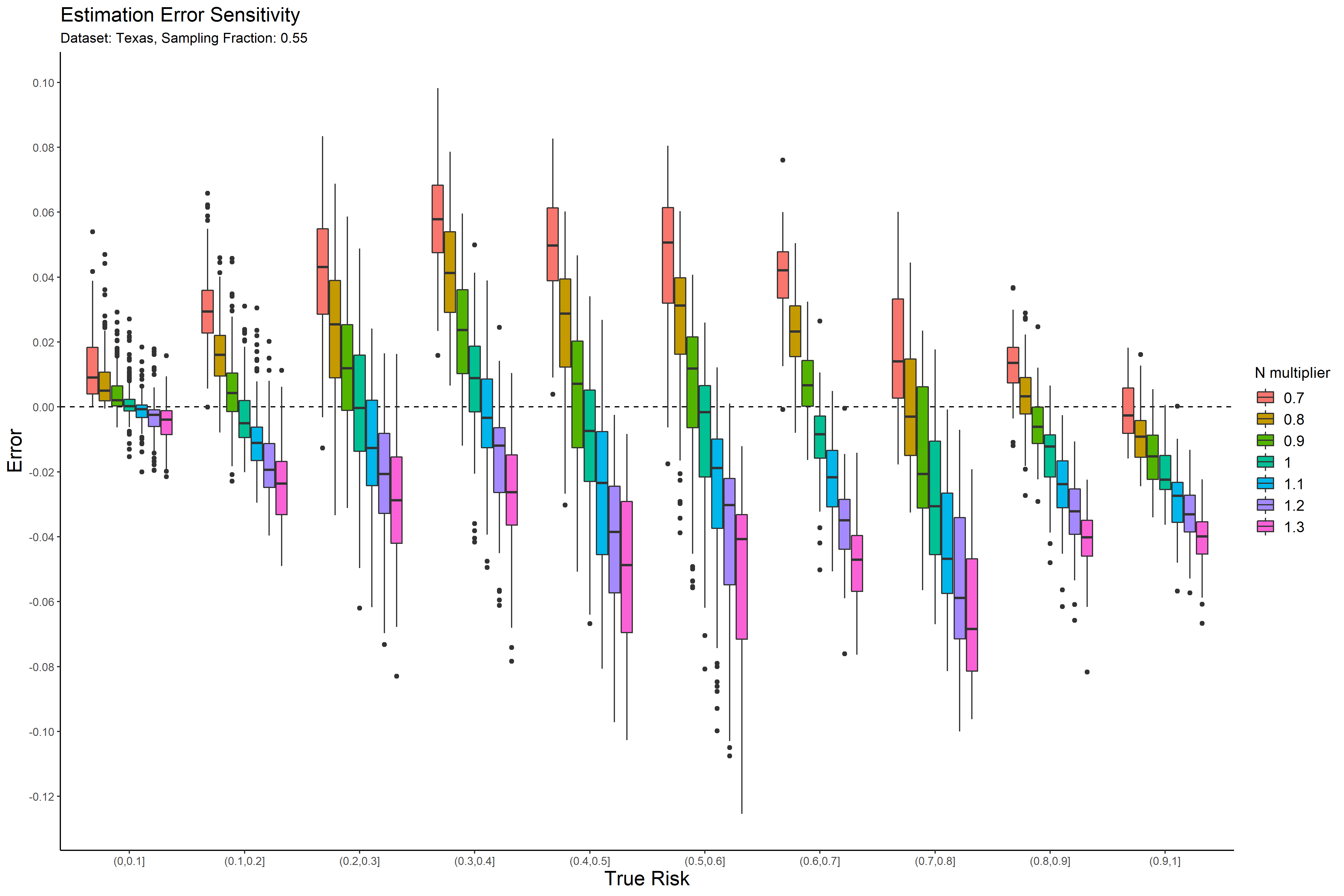

Supplement: S2 File — (ZIP) [file pone.0269097.s002.zip › tx/sensitivity.tx.11.png]

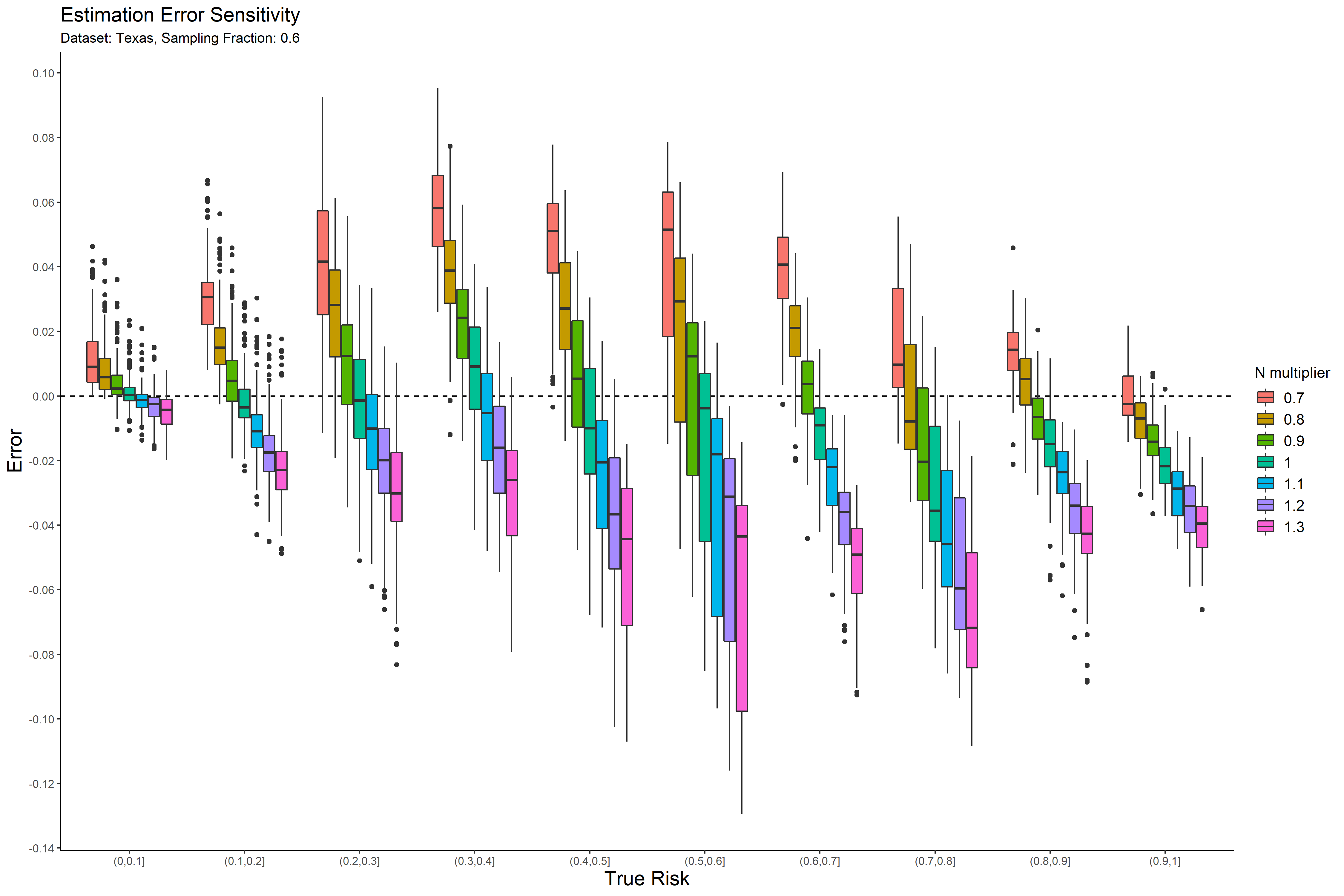

Supplement: S2 File — (ZIP) [file pone.0269097.s002.zip › tx/sensitivity.tx.12.png]

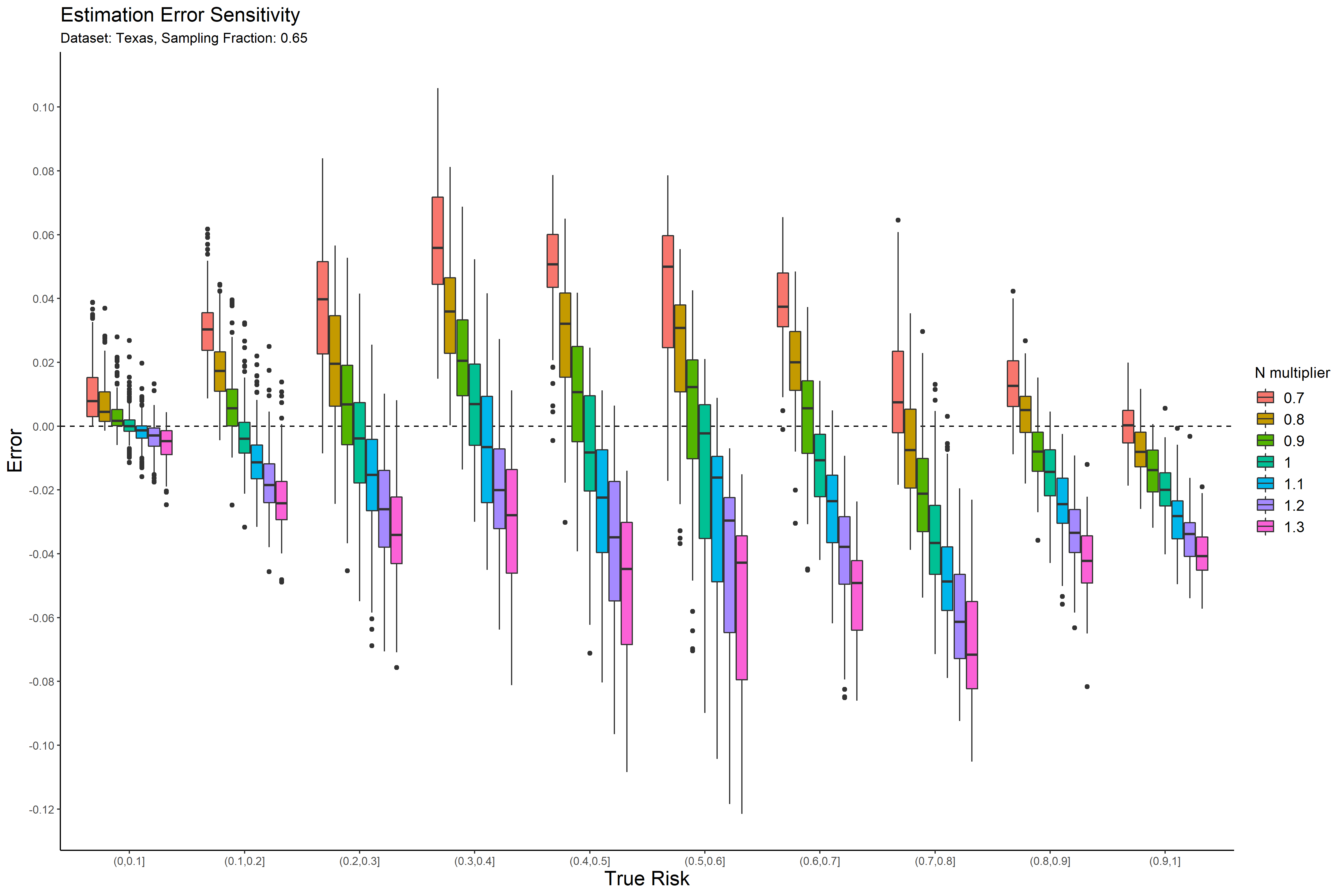

Supplement: S2 File — (ZIP) [file pone.0269097.s002.zip › tx/sensitivity.tx.13.png]
